# Curvibacter soli sp. nov., Extensimonas soli sp. nov., Pseudarthrobacter naphthalenicus sp. nov. and Terripilifer ovatus gen. nov., sp. nov., four new species isolated from polluted soil

**DOI:** 10.1099/ijsem.0.006698

**Published:** 2025-03-05

**Authors:** Ze-Shen Liu, Ke-Huan Wang, Xiao-Kang Wang, Man Cai, Mei-Ling Yang, Wen-Ke Yang, De-Feng Li, Shuang-Jiang Liu

**Affiliations:** 1 State Key Laboratory of Microbial Resources, Institute of Microbiology, Chinese Academy of Sciences, Beijing 100101, PR China; 2 State Key Laboratory of Microbial Biotechnology, Shandong University, Qingdao 266237, PR China

**Keywords:** *Curvibacter soli*, *Extensimonas soli*, *Pseudarthrobacter naphthalenicus*, *Terripilifer ovatus*, polyphasic characterization, pollutant degradation

## Abstract

A taxonomic study was conducted on four bacterial strains isolated from the soil of a coking plant. Phylogenetic analysis showed that the four strains belonged to three families: *Comamonadaceae*, *Micrococcaceae* and *Roseiarcaceae*. Identification of the 16S rRNA gene exhibited that their sequence similarities were between 94.96 and 98.98% when compared to known and validly nominated species. Their genomes ranged from 3.4 to 7.2 Mb, with DNA G+C molar contents varying from 62.3 to 67.2%. The average nucleotide identities ranged from 71.4 to 92.3%, and digital DNA–DNA hybridization values were 19.7–47.0% when comparing them with closely related type species, supporting the classification of these four strains. Functional analysis demonstrated that strain H3Y2-7^T^ was robustly resistant to chromate (VI) and arsenite (III) and was able to grow on aromatic compounds including naphthalene as carbon sources even in the presence of chromate (VI). These features reflect its ability to treat combined pollutants and adapt to a polluted environment. Based on the analysis of polyphasic taxonomy, we propose the four bacterial strains representing novel species, namely *Curvibacter soli* sp. nov. (type strain H39-3-26^T^=JCM 36178^T^=CGMCC 1.61344^T^), *Extensimonas soli* sp. nov. (type strain H3M7-6^T^=JCM 36176^T^=CGMCC 1.61336^T^), *Pseudarthrobacter naphthalenicus* sp. nov. (type strain H3Y2-7^T^=JCM 36482^T^=CGMCC 1.61323^T^) and *Terripilifer ovatus* gen. nov., sp. nov. (type strain H3SJ34-1^T^=JCM 36465^T^=CGMCC 1.61333^T^).

## Data Availability

The datasets of this study can be found in online repositories. The accession numbers in the repositories can be found in the article.

## Introduction

The genus *Pseudarthrobacter*, belonging to the family *Micrococcaceae*, was established through the reclassification of the genus *Arthrobacter*. Based on phylogenetic groupings, the species of *Arthrobacter* were arranged into different groups. The internal ‘*A. oxydans* group’ was reclassified as the genus *Pseudarthrobacter*, with its members sharing high 16S rRNA gene sequence similarities (>97.5%) [1,2]. The threshold of 16S rRNA gene similarity used to delineate a new species (98.7%) is not sufficiently resolutive for this genus. Polyphasic taxonomy methods are necessary for the classification of *Pseudarthrobacter* species [2]. Fourteen species have been validly nominated and published, with *Pseudarthrobacter polychromogenes* designated as the type species (https://lpsn.dsmz.de/genus/pseudarthrobacter) [3]. Species within the genus *Pseudarthrobacter* are characterized as Gram-stain-positive, aerobic microbes with genomic DNA G+C contents ranging from 62 to 71 mol%. The major cellular fatty acid components consist of anteiso-C_15 : 0_ and anteiso-C_17 : 0_ [4,5].

*Pseudarthrobacter* species inhabit various environments and have been frequently detected in soil, including polluted soils containing polycyclic aromatic hydrocarbons (PAHs, e.g. phenanthrene (Phe) and pyrene) and heavy metals (e.g. cadmium, lead and chromium) [6,8]. Extensive studies have been conducted on *Pseudarthrobacter* regarding the degradation of diverse pollutants. For example, a crude oil- and multi-substituted benzene compound-degrading strain, *Pseudarthrobacter sulfonivorans* Ar51, was engineered for further degradation of hydroquinone by expressing two subunits of hydroquinone dioxygenase [9]. Another strain *Pseudarthrobacter defluvii* E5, isolated from agricultural soils, demonstrated efficient 1,2-benzenedicarboxylic acid (PA) ester degradation and mineralization abilities, showing broad application prospects in the remediation of microplastic-contaminated environments [10]. Species of *Pseudarthrobacter* have also shown abilities to degrade phenol and 2,4,6-trinitrotoluene [11,12].

*Extensimonas* and *Curvibacter* are two genera within the family *Comamonadaceae*. *Extensimonas* was first described by Zhang *et al*., with *Extensimonas vulgaris* JCM 17803^T^ as the type strain [13]. *E. vulgaris*, isolated from industrial wastewater, and '*Extensimonas perlucida*', isolated from activated sludge treating pesticide manufacturing, are the only two known species of this genus (https://lpsn.dsmz.de/genus/extensimonas) [3]. Species of this genus share major fatty acids, including C_16 : 0_, cyclo-C_17 : 0_ and C_16 : 1_ ω7c/C_16 : 1_ ω6c. Their genomic DNA G+C molar contents are around 65%. The optimum pH and salinity for growth are pH 7.0 and 0.5% NaCl (w/v), respectively [13,14]. The genus *Curvibacter* was originally described in 2004, with *Curvibacter gracilis* as the type species [15]. It has since expanded to include four validly nominated species (isolated from well water and distilled water) and two not validly published species (isolated from a freshwater reservoir) (https://lpsn.dsmz.de/genus/curvibacter) [3]. Species of the genus *Curvibacter* are described as Gram-negative, heterotrophic, aerobic and curved rod-shaped bacteria, with DNA G+C molar contents ranging from 62.2 to 66.7%. Their optimum growth conditions are neutral pH and low salinity. *Curvibacter* species contain C_16 : 0_, C_18 : 1_ ω7c, C_16 : 1_ ω7c/C_16 : 1_ ω6c and C_8 : 0_ 3-OH as their major fatty acids. Cyclo-C_17 : 0_ occurs in individual species, such as *Curvibacter delicatus* NBRC 14919^T^ [16] and *Curvibacter fontanus* AQ9^T^ [17]. Currently, no species of the genera *Extensimonas* and *Curvibacter* have been isolated from soil.

The family *Roseiarcaceae* was established based on a light-pink-pigmented, microaerophilic bacterium, proposing *Roseiarcus fermentans* as the type species [18]. The genus *Rhodoblastus* was proposed following the reclassification of *Rhodopseudomonas*, with *Rhodoblastus acidophilus* as the type species, which was then transferred to the *Roseiarcaceae* family [19]. Currently, this family includes only the two genera members, *Rhodoblastus* and *Roseiarcus* (https://lpsn.dsmz.de/family/roseiarcaceae) [3], along with close relatives distributed in the families *Methylocystaceae* and *Beijerinckiaceae*. The type strain *R. fermentans* (with type strain Pf56^T^) was isolated from a methanotrophic consortium enriched from acidic *Sphagnum* peat and described as Gram-negative, non-motile, thick curved rod-shaped alphaproteobacteria that contained a vesicular intracytoplasmic membrane system characteristic of some purple non-sulphur bacteria. It can produce bacteriochlorophyll and carotenoids but is unable to grow phototrophically under anoxic conditions in the light [18]. The genus *Rhodoblastus* includes two species, namely *R. acidophilus* (with type strain DSM 137^T^) and *Rhodoblastus sphagnicola* (with type strain DSM 16996^T^), which were isolated from acidic *Sphagnum* peat bog [20]. These species were described as red- or purplish red-pigmented, motile by means of polar flagella and rod-shaped purple non-sulphur bacteria capable of growing photolithoautotrophically or photoheterotrophically under anaerobic or microaerobic conditions. The cells contain lamellar intracytoplasmic membranes and reproduce by budding. The major cellular fatty acids observed in the *Roseiarcaceae* family typically include C_16 : 0_, C_16 : 1_ ω7c and C_18 : 1_ ω7c, and sometimes cyclo-C_19 : 0_ ω8c. The cells are pigmented with the production of bacteriochlorophyll and carotenoids, and the DNA G+C content ranges from 62.2 to 70.0 mol% [18,20]. The current species of the family *Roseiarcaceae* are derived from acidic *Sphagnum* peat bog samples, and none have been isolated from other environments.

The vast diversity of microbes residing in soils remains largely unexplored, with many yet to be cultivated or characterized [21,22]. In a prior study, we investigated the microbial diversity within polluted soils from an abandoned coking plant [23]. This current study presents the isolation and genotypic and phenotypic characterization of four novel bacterial strains, namely H3M7-6^T^, H39-3-26^T^, H3Y2-7^T^ and H3SJ34-1^T^, derived from soil samples contaminated with the PAHs, including naphthalene (Nap), fluoranthene and pyrene, as well as heavy metals, including chromium and arsenic. Using polyphasic taxonomy methods, these strains were identified and classified as four novel species affiliated with the genera *Extensimonas* and *Curvibacter* of the family *Comamonadaceae*, the genus *Pseudarthrobacter* of the family *Micrococcaceae* and a newly proposed genus, *Terripilifer*, of the family *Roseiarcaceae*.

## Methods

### Sample collection

Soil samples were collected from the central region of an abandoned coking plant field where coal and iron were stacked, as described by Yang *et al*. [23]. The coking plant, which operated for nearly 60 years before closing in 2015, is located in Hangzhou, Zhejiang Province, China, at a latitude of 30° 21′ 16″ N and a longitude of 120° 09′ 31″ E. Sampling was carried out in December 2020, with local temperatures ranging from 5 to 9 °C. These soil specimens exhibited visible adherence to a mass of black, sticky contaminants. Preliminary analysis of the pollutants revealed the presence of heavy metals along with excess PAHs, including 10.6 mg kg^−1^ Nap, 183.1 mg kg^−1^ pyrene, 83.0 mg kg^−1^ fluoranthene, 66.9 mg kg^−1^ chromium and 23.0 mg kg^−1^ arsenic, among others [23]. Following collection, the samples were stored in sterile PE bags at 4 °C and transported to the laboratory within 24 h.

### Cultural media, isolation and cultivation conditions

Soil samples were suspended in 200 ml sterile phosphate buffer (PBS, pH 7.5) with a 1% inoculum and shaken for 2 h in a 500 ml conical flask using a rotary shaker (Zhichu, ZQLY180, China) at 150 r.p.m. After natural settlement, the supernatant was diluted to 10^−5^ and 10^−6^ using PBS, spread onto agar plates with each containing 20 ml of media and incubated at either 30 °C or room temperature (20–25 °C). Three kinds of media were used to isolate bacterial strains from the soil samples in the investigation of culturable micro-organisms. The composition of the M1 medium (per 1 l) included yeast extract 0.1 g, proteose peptone 0.1 g, casamino acids 0.1 g, glucose 0.1 g, soluble starch 0.1 g, sodium pyruvate 0.06 g, K_2_HPO_4_ 0.3 g, MgSO_4_.7H_2_O 0.05 g and agar 15.0 g. The medium was adjusted to pH 7.2 and autoclaved for 30 min at 105 °C. M2 medium (per 1 l) comprised K_2_HPO_4_ 1.6 g, KH_2_PO_4_ 0.4 g, MgSO_4_.7H_2_O 0.2 g, CaCl_2_.2H_2_O 0.1 g, NaCl 0.1 g, (NH_4_)_2_SO_4_ 0.5 g, NaNO_3_ 0.5 g, benz(a)anthracene 0.01 g, benzo(a)pyrene 0.01 g, agar 15.0 g and trace metal solution 1.0 ml. The trace metal solution contained (per 1 l) FeSO_4_.7H_2_O 2.0 g, ZnSO_4_.7H_2_O 0.1 g, MnCl_2_.4H_2_O 0.03 g, H_3_BO_3_ 0.3 g, CoCl_2_.6H_2_O 0.2 g, CuCl_2_.2H_2_O 0.01 g, NiCl_2_.6H_2_O 0.02 g and Na_2_MoO_4_.2H_2_O 0.03 g. Benz(a)anthracene and benzo(a)pyrene were dissolved in acetone and spread on the plates after agar solidification. Before spreading the cell suspension, the acetone was volatilized in a clean bench (AirTech, China) under ventilation. To create an M3 medium, a soil extract solution was added to M1 at a 10% dosage. For the soil extract solution, 5.0 g of soil was mixed with 150 ml distilled water and agitated at 150 r.p.m. for 30 min. The soil–water mixture was then centrifuged to obtain the supernatant as the soil extract solution. Colonies were picked upon appearance after cultivation for 3–14 days and re-streaked on Reasoner's 2A (R2A) [24] agar plates. H3Y2-7^T^ was isolated from the M2 agar plate. H3M7-6^T^ was isolated from the M1 agar plate. H39-3-26^T^ and H3SJ34-1^T^ were isolated from M3 agar plates.

### Heavy metal resistance and aromatic compound metabolism

Bacterial strains were cultured in an R2A medium [24] to prepare seed cultures. The cells were collected after centrifugation at 4000 r.p.m. and washed twice with an equal volume of PBS. For the heavy metal resistance test, 5 µl of washed seed cultures was inoculated into a 96-well microplate, with each well containing 150 µl of R2A medium supplemented with 0.5, 2.0, 5.0 or 10.0 mM sodium arsenite (Sigma, Germany) or 5, 20, 50 or 100 mg l^−1^ potassium dichromate. The 96-well microplates were cultured at 30 °C for 13 days. To assess aromatic compound metabolism, 5 µl of washed seed cultures was inoculated into another 96-well microplate, with each well containing 150 µl of minimal salt medium (MSM) [25], which was supplemented with 100 mg l^−1^ of 2,5-dihydroxybenzoic acid (DHB), 4-hydroxybenzoic acid (4HB), 3,4-dihydroxybenzoic acid (PCA), 2-hydroxybenzoic acid (SA), PA, benzoic acid (BA), Nap or Phe (Mreda, China) as the sole carbon sources. The 96-well microplates were cultured at 30 °C for 30 days. The OD_600_ of each well was monitored with a multimode plate reader (PerkinElmer, VICTOR Nivo) daily for the first 5 days and every 4 days thereafter. The maximum increase in OD_600_ from the initial values was selected to evaluate bacterial growth under the stress of different concentrations of heavy metals or by metabolizing different aromatic compounds. The tests were carried out in duplicates.

### Organic pollutant degradation

To assess PAH-degrading ability, 100 µl of washed seed cultures was inoculated into 5 ml of MSM, supplemented with 200 mg l^−1^ of Phe or 200 mg l^−1^ of Nap, with or without heavy metals (0.5 mM sodium arsenite or 5 mg l^−1^ of potassium dichromate). The inocula were then incubated at 30 °C and 160 r.p.m. for 3 or 10 days to assay the degradation of Nap and Phe, respectively. The residual PAHs were extracted using dichloromethane and analysed using the HPLC method [23,26]. The degradation of phenol (250 mg l^−1^), mono-methyl phthalate (MMP, 400 mg l^−1^), mono-butyl phthalate (MBP, 400 mg l^−1^) and n-hexadecane (C16, 5%) (Mreda, China) was tested in 20 ml of MSM at 30 °C and 160 r.p.m., using these chemicals as the sole carbon sources. After cultivation, the broth was diluted with deionized water and filtered through a 0.45 µm membrane. The residual phenol, MMP and MBP in the broth were monitored using the HPLC (Agilent 1260, USA) method under the following conditions: column, Eclipse Plus C18 (4.6 mm×150 mm, 5 µm); eluent, acetonitrile: water (50 : 50); flow rate, 1.0 ml min^−1^; and injection volume, 10 µl. The wavelength of the fluorescence detector was set at 224 nm. The C16 was extracted with n-hexane and detected using the GC (Agilent 7890A, USA) method under the following conditions: capillary column, HP-5 (60 m×250 μm×0.25 µm) composed of 5% phenyl-ethyl-siloxane; detector, flame ionization; carrier gas, nitrogen; temperature programme, initial temperature of 70 °C, followed by ramping at 10 °C min^−1^ to 310 °C; flow rate, 1 ml min^−1^; and injection volume, 1 µl. Tests without inoculation were served as controls. The degradation percentage of substrate content was calculated by comparing it with the control. The tests were repeated three times.

### Morphology observation and physiologic and chemotaxonomic determinations

The single-cell morphology of the bacterial strains was observed using transmission electron microscopy after cultivation on R2A agar plates at 30 °C for 2–3 days. Cell size was measured from the electron microscope photographs using ImageJ (version 1.53 t) software [27]. Motility was assessed using a light microscope (Olympus, CX31, China) at 1000-fold magnification.

Hydrolysis of starch, casein and Tween 60 was evaluated following the methods described by Luo *et al.* [28]. Anaerobic growth was tested using R2A liquid medium at 30 °C for 15 days in sealed bottles that filled with nitrogen. The Gram stain kit and Schaeffer–Fulton stain kit (Mreda, China) were used to assess Gram-staining and endospore-forming activities according to the manufacturer’s instructions. Fumarate metabolism was tested using MSM medium at 30 °C for 10 days with fumaric acid as the sole carbon source. Catalase activity was assessed by dribbling 10 µl of 3% (w/v) hydrogen peroxide onto bacterial colonies that cultured on R2A agar plates [5]. The occurrence of bubbles indicated a positive reaction for catalase. H_2_S production was examined in the R2A agar medium supplemented with 0.25% sodium thiosulphate and 0.01 % lead acetate [29]. The appearance of black colonies indicated positive results for H_2_S formation. Methyl red and Voges–Proskauer (V–P) reactions were tested with the cultured bacterial broth in the R2A medium containing 5 g l^−1^ glucose. The chromogenic reaction of the cultured broth was conducted by adding methyl red or creatine, following the method described by Huang *et al.* [30]. The API ZYM and API 20NE kits (bioMérieux, France) and the Biolog^™^ GEN III microplate system (BIOLOG, USA) were used to determine additional physiologic characteristics and enzyme activities of the bacterial strains according to the manufacturer’s protocol [5].

The cells of H39-3-26^T^ and H3M7-6^T^ for fatty acid identification were cultured in the R2A agar medium under the same conditions as their respective reference type strains. The cells of strain H3Y2-7^T^ and its reference type strain were cultured in the R2A liquid medium at 25 °C following the method described by Shin *et al.* [5]. Strain H3SJ34-1^T^ was cultured according to the medium described by Kulichevskaya *et al.* [20]. Cellular fatty acids were extracted and detected according to the standard MIDI protocol (Sherlock Microbial Identification System, version 6.0) [31]. Polar lipids were separated by two-dimensional TLC on silica TLC plates. Total lipids, aminolipids, phospholipids and glycolipids were then stained following the methods described by Minnikin *et al.* [32].

### 16S rRNA gene and genome sequencing

The total DNA of the cultured bacteria was extracted using TIANamp Bacteria DNA Kits (TIANGEN, China) and used for 16S rRNA gene and genome sequencing. The universal primers 27F (5′-AGAGTTTGATCCTGGCTCAG-3′) and 1492R (5′-GGTTACCTTGTTACGACTT-3′) were employed to amplify the full-length 16S rRNA genes [33]. The amplification of the 16S rRNA gene was conducted following the PCR procedure described by Abdugheni *et al.* [34]. The PCR products were sequenced by Tianyihuiyuan Biotechnology Co., Ltd. (Beijing, China). Genome sequencing, assembly and annotation were conducted on the Global Catalogue of Type Strain (gcType) Platform [35,36]. In brief, the total extracted DNA was sequenced on a next-generation sequencing platform (Illumina HiSeq X Ten, USA). Assemblies were performed using multiple assemblers, including SPAdes [37], IDBA [38], SOAPdenovo2 [39] and Platanus-b [40]. The assembled genomes were annotated with DIAMOND software [41]. The detailed protocol, parameters and versions of the software are described in the gcType Platform Manual v2 (https://gctype.wdcm.org/manual.jsp#detail) [35]. The RAST engine was also employed to annotate the genomes (http://rast.nmpdr.org/).

### Phylogenetic and genomic analysis

The similarities of the 16S rRNA gene sequences with the previously reported type strains were determined using the EzBioCloud server [42]. The 16S rRNA gene sequences of type strains were retrieved from the EzBioCloud server and aligned using clustalw [43]. Three distinct strategies were employed to construct phylogenetic trees using mega software (version 11) [44]. These strategies included the neighbour-joining (NJ) algorithm based on Kimura’s two-parameter model [45], the maximum-parsimony (MP) algorithm using the Subtree-Pruning-Regrafting search method [46] and the maximum-likelihood (ML) algorithm based on the Tamura–Nei model [47], with a site coverage cutoff value of 95%. Bootstrap analysis with 1000 replications was performed to assess the statistical reliability of the trees [48].

Average nucleotide identity (ANI) values were calculated using the OrthoANI calculator (https://www.ezbiocloud.net/tools/orthoani), and a UPGMA dendrogram was constructed using OAT (version 0.93.1) software [49,50]. Digital DNA–DNA hybridization (dDDH) and DNA G+C contents were determined using the Genome-to-Genome Distance Calculator (version 3.0; https://ggdc.dsmz.de/ggdc.php#) [51]. Whole genome-based phylogenomic trees were constructed using the Up-to-Date Bacterial Core Gene pipeline (https://www.ezbiocloud.net/tools/ubcg) [52] and the Integrated Prokaryotes Genome and pan-genome Analysis (IPGA, version 1.09) pipeline (https://nmdc.cn/ipga/) [53]. Plasmid annotation was carried out using the online tool of the Majorbio Cloud Platform (https://cloud.majorbio.com/page/tools/) [54]. Virus sequences were predicted and annotated based on the bacterial genomes using the PHAST programme [55].

## Results and discussion

### Four bacterial strains representing novel species

Four bacterial strains, namely H3Y2-7^T^, H3SJ34-1^T^, H3M7-6^T^ and H39-3-26^T^, were obtained from polluted soil and were phylogenetically related to previously known and validly nominated species in the families *Comamonadaceae*, *Micrococcaceae* and *Roseiarcaceae*. Based on the analysis of the phylogenetic relationships of each bacterial strain with their closely related species, as well as results from DNA molecular analysis and phenotypic and chemotaxonomic characterization, we propose that these four bacterial isolates represent novel species.

#### Strain H3Y2-7^T^

**Fig. 1.**
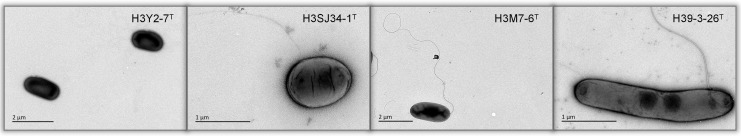
Transmission electron microscopy of the four bacterial strains illustrated their cellular morphology. Bacterial names and cellular size scale bars are shown in the images.

Strain H3Y2-7^T^ was obtained from M2 agar plates. Growth was observed after it was re-streaked onto R2A agar plates, and it formed visible colonies within 24 h. The colonies were white, smooth, convex and non-transparent. Cells of strain H3Y2-7^T^ were Gram-positive, short rods (0.93–1.70 µm in length and 0.63–0.85 µm in width) and aerobic and did not grow under anaerobic conditions. Endospores were not formed, and no flagella were observed on the cell surfaces (Table 1, Fig. 1).

**Table 1.** Comparison of phenotypic and chemotaxonomic features of the four novel species and their closely related species

Strain	Gram stain	Morphology	Appendage	Size (μm)	Colony	Major fatty acid	Major polar lipid
1	P	Short rod-shaped	No	0.93–1.70 L; 0.63–0.85 W	White, smooth, convex and non-transparent	Anteiso-C_15 : 0_, anteiso-C_17 : 0_ and iso-C_15 : 0_	DPG, PI, GLs and L
2	P	Rod-shaped	No	2.0–5.1 L; 0.7–0.9 W	/	Anteiso-C_15 : 0_, anteiso-C_17 : 0_, iso-C_15 : 0_, C_16 : 0_ and iso-C_16 : 0_	DPG, PG and GL
3	P	Rod-shaped	No	2–5.1 L; 0.7–0.9 W	/	Anteiso-C_15 : 0_, anteiso-C_17 : 0_, iso-C_15 : 0_, C_16 : 0_ and iso-C_16 : 0_	DPG, PG and GL
4	P	Rod-shaped	No	/	White or greyish, smooth, convex and non-transparent	Anteiso-C_15 : 0_, anteiso-C_17 : 0_, iso-C_15 : 0_, C_16 : 0_ and iso-C_16 : 0_	DPG, PG, PI, GLs and L
5	N	Ovoid-shaped	Polar flagellum	0.88–1.98 L; 0.67–0.97 W	White, smooth, convex and circular	C_16 : 0_, C_18 : 0_, C_15 : 0_ 3-OH, C_18 : 1_ ω7c and C_19 : 1_ ω7c/C_19 : 1_ ω6c	DPG, PE, PC, PG, PI, GLs and PL
6	N	Rod or ovoid-shaped	Polar flagellum	2.0–5.0 L; 1.0–1.3W	Red to orange-red	C_16 : 0_, C_16 : 1_ ω7c and C_18 : 1_ ω7c	/
7	N	Rod-shaped; straight or slightly curved	Polar flagella	2.0–6.0 L; 0.8–1.0 W	Purplish red	C_16 : 0_, C_18 : 1_ ω7c and C_16 : 1_ ω7c	/
8	N	Thick curved rod-shaped	No	2.7–4.0 L; 0.6–1.2W	Pink	Cyclo-C_19 : 0_ ω8c and C_18 : 1_ ω7c	DPG, PE, PC, PG and SL
9	N	Short rod-shaped	Polar flagellum	1.72–2.29 L; 0.96–1.06 W	Circular, smooth, convex and transparent	C_16 : 0_, C_16 : 1_ ω7c/C_16 : 1_ ω6c, cyclo-C_17 : 0_ and C_18 : 1_ ω7c	DPG, PE, PG, APL, PLs, GLs and AL
10	N	Short rod-shaped	Polar flagellum	1.3–1.9 L; 0.8–0.9 W	Circular, convex and transparent	C_16 : 0_, C_16 : 1_ ω7c/C_16 : 1_ ω6c, cyclo-C_17 : 0_ and C_18 : 1_ ω7c	DPG, PE, PG, APL and PLs
11	N	Rod-shaped	Polar flagellum	/	Circular, transparent and colourless	C_16 : 0_, C_16 : 1_ ω7c/C_16 : 1_ ω6c and cyclo-C_17 : 0_	DPG, PE, PG, APL, GPL and AGL
12	N	Rod-shaped	Polar flagellum	1.52–3.02 L; 0.46–0.60 W	Circular, smooth, convex and transparent	C_16 : 0_, cyclo-C_17 : 0_, C_18 : 1_ ω7c, C_16 : 1_ ω7c/C_16 : 1_ ω6c and C_12 : 0_ 2-OH	DPG, PE, PG, APLs and Ls
13	N	Concave-shaped	No	1.1–2.8 L; 0.4–0.5 W	Yellow to brown, circular, smooth and convex	C_16 : 0_, cyclo-C_17 : 0_, C_18 : 1_ ω7c and C_16 : 1_ ω7c/C_16 : 1_ ω6c	/
14	N	Short rod-shaped	Polar flagellum	1.2–1.8 L; 0.6–0.9 W	Colourless, smooth and non-transparent	C_16 : 0_, C_18 : 1_ ω7c and C_16 : 1_ ω7c/C_16 : 1_ ω6c	/

1, H3Y2-7T; 2, *P. sulfonivorans* ALLT [4]; 3, *P. psychrotolerans* YJ56T [5]; 4, *P. polychromogenes* JCM 2523T [1]; 5, H3SJ34-1T; 6, *R. acidophilus* DSM 137T [19,20]; 7, *R. sphagnicola* DSM 16996T [20]; 8, *R. fermentans* Pf56T [18]; 9, H3M7-6T; 10, *E. vulgaris* JCM 17803T [13,14]; 11, '*Extensimonas perlucida*' HX2-24 [14]; 12, H39-3-26T; 13, *C. gracilis* JCM 21496T [15,17]; 14, *Curvibacter lanceolatus* ATCC 14669T [15,17]. Data of the four studied strains are derived from this study. Data for their closely related species are referenced from previous studies as indicated. /, not reported.DPG, diphosphatidylglycerol; PE, phosphatidylethanolamine; PC, phosphatidylcholine; PG, phosphatidylglycerol; SL, sphingolipid; PI, phosphatidylinositol; PL, unknown phospholipid; GL, unknown glycolipid; APL, unknown aminophospholipid; AGL, unknown aminoglycolipid; AL, unknown aminolipid; GPL, unknown glycophospholipid;L, unknown lipid.

AGLunknown aminoglycolipidALunknown aminolipidAPLunknown aminophospholipidDPGdiphosphatidylglycerolGLunknown glycolipidGPLunknown glycophospholipidLunknown lipidPCphosphatidylcholinePEphosphatidylethanolaminePGphosphatidylglycerolPIphosphatidylinositolPLunknown phospholipidSLsphingolipid

**Fig. 2.**
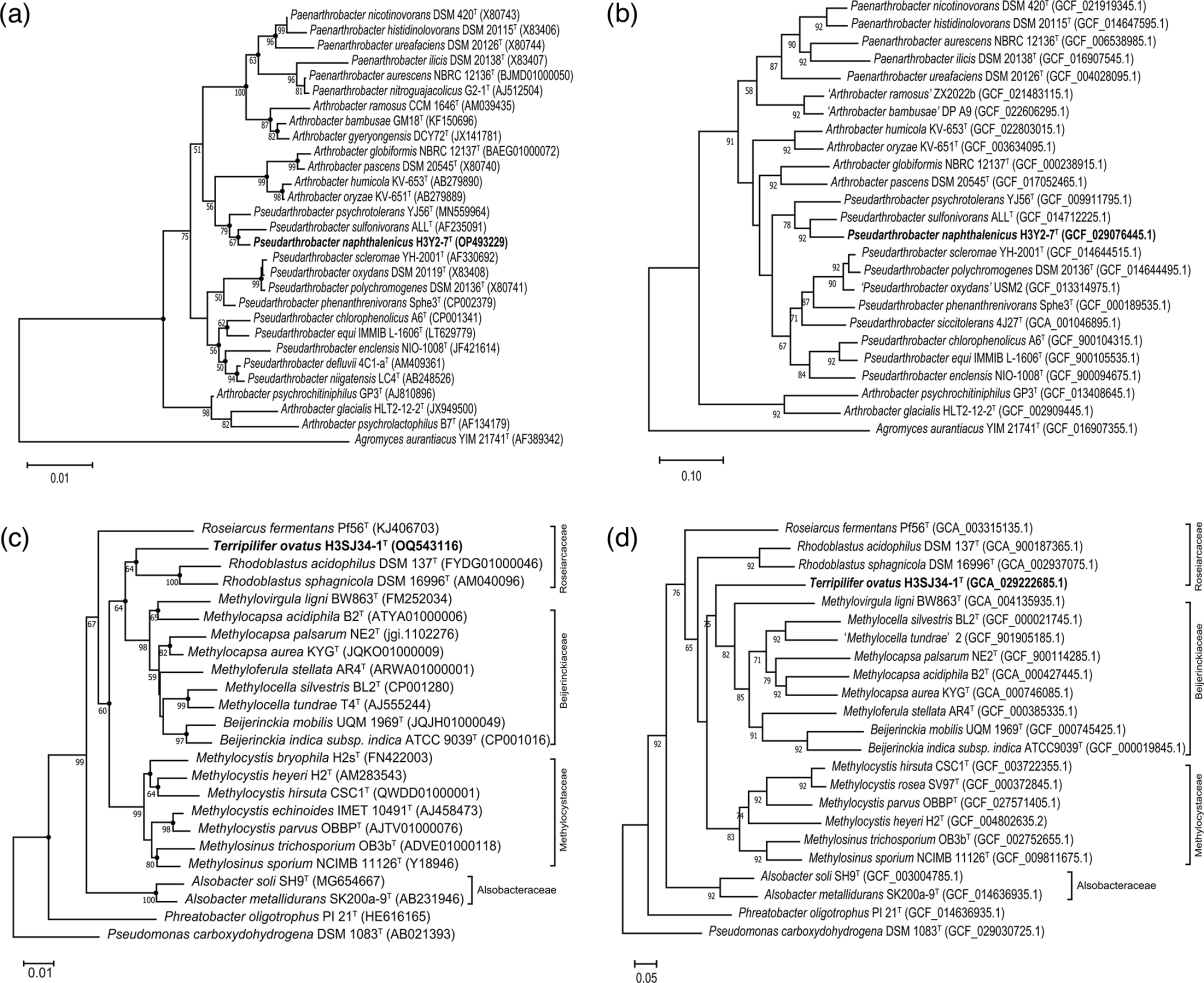
Phylogenetic and phylogenomic trees of strains H3Y2-7^T^ and H3SJ34-1^T^ showed the relationships with their closely related type strains. Phylogenetic (**a**) and phylogenomic (**b**) trees of strain H3Y2-7^T^ and its closely related species in the family *Micrococcaceae*, with *Agromyces aurantiacus* YIM 21741^T^ (AF389342) as the outgroup; phylogenetic (**c**) and phylogenomic (**d**) trees of strain H3SJ34-1^T^ and its closely related species in the families *Roseiarcaceae*, *Beijerinckiaceae*, *Methylocystaceae* and *Alsobacteraceae*, using *Pseudomonas carboxydohydrogena* DSM 1083^T^ (AB021393) and *Phreatobacter oligotrophus* PI 21^T^ (HE616165) as outgroups. The trees were constructed using the NJ algorithm. Phylogenetic trees were also constructed using the ML and the MP methods with 1000 bootstraps (see Fig. **S2**). Phylogenomic trees were also constructed using the IPGA pipeline (see Fig. **S4**). Filled circles indicate nodes supported by all three methods regardless of bootstrap percentages. GenBank accession numbers are provided in parentheses. Bootstrap percentages (>50%) based on 1000 replicates are shown at the nodes. Bar, 0.01 substitutions per nucleotide (nt) position for Fig. 4(a, c); bar, 0.1 substitutions per nt position for (**b)**; bar, 0.05 substitutions per nt position for (**d)**.

**Fig. 3.**
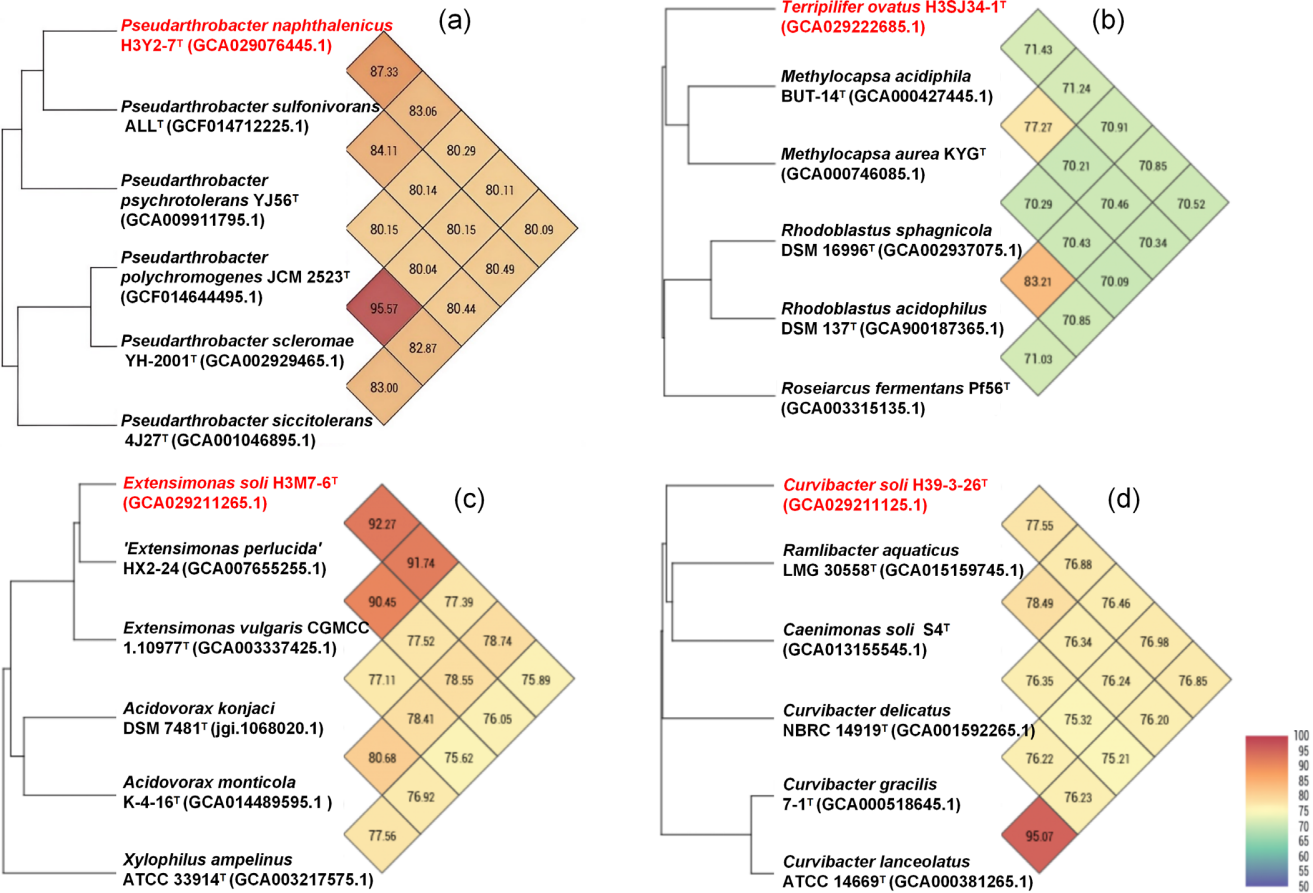
UPGMA phylogenetic trees and ANI heat maps illustrated different genomic characteristics between the four species and their close relatives. (a) H3Y2-7^T^; (**b**) H3SJ34-1^T^; (**c**) H3M7-6^T^; (**d)** H39-3-26^T^. GenBank accession numbers for the genomes are shown in parentheses. Proposed novel taxon names are highlighted in red.

Phylogenetic analysis was carried out to determine the classification of strain H3Y2-7^T^. Both the phylogenetic and phylogenomic trees revealed that H3Y2-7^T^ clustered with members of the genus *Pseudarthrobacter* (Fig. 2a, b). DNA molecular characterization exhibited a G+C content of 65.2 mol%, which falls within the range (62–71 mol%) for the genus *Pseudarthrobacter* [1]. The close relatives to H3Y2-7^T^ were *P. psychrotolerans* YJ56^T^ (98.98%, 16S rRNA gene identity) and *P. sulfonivorans* ALL^T^ (98.32%). It also showed a 97.70% 16S rRNA gene identity with the type species of the genus, *P. polychromogenes*. Due to members of *Pseudarthrobacter* typically sharing high 16S rRNA gene similarities (>97.5%), the 16S rRNA similarity threshold used to delineate a new species is not sufficiently resolutive for this genus [2]. In this study, although H3Y2-7^T^ shared the highest 16S rRNA gene identity of 98.98% with *P. psychrotolerans* YJ56^T^, the ANI and dDDH values based on genomic comparison were all below the threshold for differentiating two species (ANI, 95% [56], and dDDH, 70% [57]). The ANI and dDDH values between H3Y2-7^T^ and *P. psychrotolerans* YJ56^T^ were 83.1 and 26.3%, respectively, while the two values were 87.3 and 33.2% for *P. sulfonivorans* ALL^T^ and 80.3 and 26.4% for *P. polychromogenes* JCM 2523^T^ (Table 2, Fig. 3a). Therefore, these data support the conclusion that H3Y2-7^T^ represents a new species of *Pseudarthrobacter*.

**Table 2.** Comparison of genomic features of the four novel species and their closely related species

Strain	Genome size (Mbp)	Gene no.	DNA G+C content (mol%)	Identity of 16S rRNA gene (%)	dDDH (%)
1	4.6	4287	65.2	–	–
2	4.9	4701	65.1	98.32	33.2
3	5.2	5092	64.7	98.98	26.3
4	4.4	4085	65.8	97.70	26.4
5	7.2	6730	62.3	–	–
6	4.7	4377	65.3	94.96	19.4
7	5.1	4921	62.6	94.81	19.9
8	6.8	6173	70.0	93.03	19.4
9	3.4	3144	65.3	–	–
10	3.1	2777	68.8	96.89	45.3
11	3.6	3294	64.4	97.38	47.0
12	3.6	3126	67.2	–	–
13	6.8	6429	66.2	97.25	21.2
14	6.8	6640	66.0	97.31	21.2

1, H3Y2-7T; 2, *P. sulfonivorans* ALLT [4]; 3, *P. psychrotolerans* YJ56T [5]; 4, *P. polychromogenes* JCM 2523T [1]; 5, H3SJ34-1T; 6, *R. acidophilus* DSM 137T [19,20]; 7, *R. sphagnicola* DSM 16996T [20]; 8, *R. fermentans* Pf56T [18]; 9, H3M7-6T; 10, *E. vulgaris* JCM 17803T [13,14]; 11, '*Extensimonas perlucida*' HX2-24 [14]; 12, H39-3-26T; 13, *C. gracilis* JCM 21496T [15,17]; 14, *C. lanceolatus* ATCC 14669T [15,17]. Data of the four studied strains are derived from this study. Data for their closely related species are referenced from previous studies as indicated. The identities of the 16S rRNA gene and the dDDH refer to comparing the 16S rRNA gene sequences or genome sequences of the four strains with their respective closely related type strains. Specifically, H3Y2-7T was compared with strains in lines 1–4;, H3SJ34-1T with strains in lines 5–8;, H3M7-6T with strains in lines 9–11; and H39-3-26T with strains in lines 12–14.

A comparison of physiological properties was conducted between H3Y2-7^T^ and its close relatives. Strain H3Y2-7^T^ exhibited positive results for nitrate reduction to nitrite, urea hydrolysis and assimilation of d-glucose, d-mannitol, d-maltose, gluconic acid, malic acid and citric acid, along with enzyme activities of leucine arylamidase, valine arylamidase, acid phosphatase, *β*-galactosidase, *β*-glucuronidase, naphthol-AS-BI-phosphohydrolase and *α*-glucosidase (Table 3). These properties were consistent with those of its closest relative, *P. psychrotolerans* YJ56^T^. In addition to these similarities, they exhibited opposite characteristics in casein hydrolysis, assimilation of l-arabinose and d-maltose and enzyme activities of alkaline phosphatase, esterase (C4), esterase lipase (C8), cystine arylamidase, *α*-galactosidase, *β*-glucosidase, *α*-mannosidase and catalase. Strain H3Y2-7^T^ was also positive for H_2_S production and fumaric acid metabolism, but negative for indole production, Tween 80 hydrolysis, V–P and methyl red tests, which were similar to *P. polychromogenes*. However, they differed in characteristics such as nitrate reduction, starch and urea hydrolysis and assimilation of l-arabinose and d-maltose. These observations confirmed that H3Y2-7^T^ belongs to a novel species.

**Table 3.** Differential physiological properties of the four novel species and their closely related species

Strain	1	2	3	4	5	6	7	8
**Reduction of nitrate to nitrite**	+	+	−	+	+	+	−	+
**Catalase activity**	+	−	+	−	+	+	−	+
**H**_2_ **S production**	+	/	+	−	−	−	+	+
**V–P**	−	/	−	−	−	−	−	+
**Methyl red**	−	/	−	−	−	−	−	+
**Fumaric acid metabolization**	+	/	+	−	−	−	+	−
**Hydrolysis of:**								
Starch	−	−	+	−	−	−	−	−
Casein	+	−	+	−	−	−	−	−
Tween 80	−	/	−	−	+	+	+	+
Urea	+	+	−	+	−	−	+	+
**Assimilation (API 20NE) of:**								
d-Glucose	+	+	+	−	−	−	−	+
d-Mannitol	+	+	+	−	−	−	−	−
d-Maltose	w	+	+	−	−	−	−	−
Gluconic acid	+	+	+	−	−	+	−	+
Malic acid	+	+	+	−	−	−	−	−
Citric acid	+	+	+	−	−	−	−	−
l-Arabinose	−	+	+	−	−	−	−	−
d-Maltose	−	+	+	−	−	−	−	−
**Enzyme activity (API ZYM) of:**								
Alkaline phosphatase	w	−	+	+	+	+	+	+
Esterase (C4)	+	−	w	+	w	+	+	+
Esterase lipase (C8)	−	+	w	+	w	+	w	+
Leucine arylamidase	+	+	+	+	−	+	+	+
Valine arylamidase	w	+	w	+	−	+	w	w
Cystine arylamidase	w	−	−	w	−	w	−	w
Acid phosphatase	+	+	+	+	+	+	+	+
Naphthol-AS-BI-phosphohydrolase	+	+	w	+	+	+	+	+
*α*-Galactosidase	−	w	w	−	−	−	−	w
*β*-Galactosidase	+	+	+	−	−	−	−	−
*β*-Glucuronidase	w	w	−	−	−	−	−	−
*α*-Glucosidase	+	+	+	−	−	−	−	−
*β*-Glucosidase	−	+	+	−	−	−	−	−
*α*-Mannosidase	+	−	+	−	−	−	−	−

1, H3Y2-7T; 2, *P. psychrotolerans* YJ56T; 3 *P. polychromogenes* JCM 2523T; 4, H3SJ34-1T; 5, H3M7-6T; 6, *E. vulgaris* JCM 17803T; 7, H39-3-26T; 8, *C. gracilis* JCM 21496T. Data for *P. psychrotolerans* YJ56T in column 2 wasere obtained from Shin *et al.* [5]. Other data were derived from this study.

Chemotaxonomically, strain H3Y2-7^T^ exhibited the major cellular fatty acids of anteiso-C_15 : 0_ (59.5%), anteiso-C_17 : 0_ (9.0%) and iso-C_15 : 0_ (15.3%) (Table S1 available in the online Supplementary Material), which was consistent with the description of the genus *Pseudarthrobacter* [1]. However, it demonstrated a lower content in the component of C_16 : 0_ and iso-C_16 : 0_ compared to its close relatives. Strain H3Y2-7^T^ contained polar lipids, including DPG, PI, GLs and other Ls (Fig. S1). Among these polar lipids, DPG was shared by all species of *Pseudarthrobacter*, while PI was absent in *P. psychrotolerans* YJ56^T^. Additionally, * P. psychrotolerans* YJ56^T^ and *P. sulfonivorans* ALL^T^ contained PG, which was not observed in strain H3Y2-7^T^. GLs were present in these species, along with other Ls found in strains H3Y2-7^T^ and *P. polychromogenes* JCM 2523^T^. The distinctions in the composition of major cellular fatty acids and polar lipids further differentiate H3Y2-7^T^ from its close relatives.

Based on the phenotypic, phylogenetic and phylogenomic evidence presented here, we conclude that strain H3Y2-7^T^ represents a novel species affiliated with the genus *Pseudarthrobacter*, and we propose the name *Pseudarthrobacter naphthalenicus* sp. nov.

#### Strain H3SJ34-1^T^

Strain H3SJ34-1^T^ was obtained from M3 agar plates. Growth was observed after it was re-streaked onto the R2A agar plate. It demonstrated a low growth rate and formed visible colonies after 3 days of cultivation. The colonies were tiny, white, smooth, convex and circular. Cells of strain H3SJ34-1^T^ were Gram-negative, ovoid-shaped (0.88–1.98 µm in length and 0.67–0.97 µm in width) and aerobic and did not grow under anaerobic conditions. Endospores were not formed, and polar flagella were observed on the cell surfaces (Table 1, Fig. 1).

Phylogenetic analysis was conducted to determine the classification of strain H3SJ34-1^T^. The phylogenetic tree revealed that H3SJ34-1^T^ clustered with members of the family *Roseiarcaceae* (Fig. 2c). DNA molecular characterization exhibited a G+C content of 62.3 mol%, which is consistent with that of the family *Roseiarcaceae* (62.6–70.0 mol%) (Table 2). The close relatives to H3SJ34-1^T^ were *R. acidophilus* DSM 137^T^ (94.96%, 16S rRNA gene identity) and *R. sphagnicola* DSM 16996^T^ (94.81%). The 16S rRNA gene identities were below the recommended threshold (95%) for differentiating two genera [58]. The ANI and dDDH values between H3SJ34-1^T^ and *R. acidophilus* DSM 137^T^ were 70.9 and 19.4%, respectively, and they were 70.9 and 19.9% for *R. sphagnicola* DSM 16996^T^ (Table 2, Fig. 3b), all of which were below the threshold for differentiating two species. The ANI values were much lower than those between the two species, *R. acidophilus* DSM 137^T^ and *R. sphagnicola* DSM 16996^T^ (83.2%). A total of two genera and three species have been described for the family *Roseiarcaceae*. Strain H3SJ34-1^T^ had a greater distance from the third species, *R. fermentans* Pf56^T^ (93.03%, 16S rRNA gene identity). In conclusion, these data support the classification of H3SJ34-1^T^ as representing a novel genus within the family *Roseiarcaceae*.

Chemotaxonomically, the major cellular fatty acids of H3SJ34-1^T^ were C_16 : 0_ (27.9%), C_18 : 0_ (9.1%), C_18 : 1_ ω7c (8.9%), C_15 : 0_ 3-OH (6.1%) and summed feature 7 (C_19 : 1_ ω7c/C_19 : 1_ ω6c, 14.4%) (Table S1). In contrast, the species of *Rhodoblastus* exhibited a significantly higher composition of C_16 : 1_ ω7c (45.4 –46.8%), while *R. fermentans* Pf56^T^ had an additional major fatty acid, cyclo-C_19 : 0_ ω8c (38.9%). This fatty acid composition distinguishes H3SJ34-1^T^ from both the genera *Rhodoblastus* and *Roseiarcus*. Strain H3SJ34-1^T^ contained polar lipids, including DPG, PE, PG, PC, PI, GLs and a PL (Fig. S1).

Physiological property analysis revealed that strain H3SJ34-1^T^ was positive for nitrate reduction to nitrite, urea hydrolysis and enzyme activities of alkaline phosphatase, esterase (C4), esterase lipase (C8), leucine arylamidase, valine arylamidase, cystine arylamidase, acid phosphatase and naphthol-AS-BI-phosphohydrolase (Table 3). It did not assimilate any carbon source from API (20NE). It was negative for catalase activity; H_2_S production; indole production; hydrolysis of starch, casein and Tween 80, as well as fumaric acid metabolism; and V–P and methyl red tests. Additionally, all the members of the family *Roseiarcaceae* form purplish red or pink-pigmented colonies due to carotenoid production [18]. In contrast, H3SJ34-1^T^ formed distinct white colonies without pigment production.

Based on the results of phenotypic characterization and phylogenetic and phylogenomic analyses, we conclude that strain H3SJ34-1^T^ represents a new species of a novel genus affiliated with the family *Roseiarcaceae*, and we propose the name *Terripilifer ovatus* gen. nov., sp. nov.

#### Strain H3M7-6^T^

Strain H3M7-6^T^ was obtained from M1 agar plates. Growth was observed after it was re-streaked onto R2A agar plates, and it formed visible colonies in 2 days. The colonies were circular, smooth, convex and transparent. Cells of strain H3M7-6^T^ were Gram-negative, short rod-shaped (1.72–2.29 µm in length and 0.96–1.06 µm in width) and aerobic and did not grow under anaerobic conditions. Endospores were not formed, and polar flagella were observed on the cell surfaces (Table 1, Fig. 1).

**Fig. 4.**
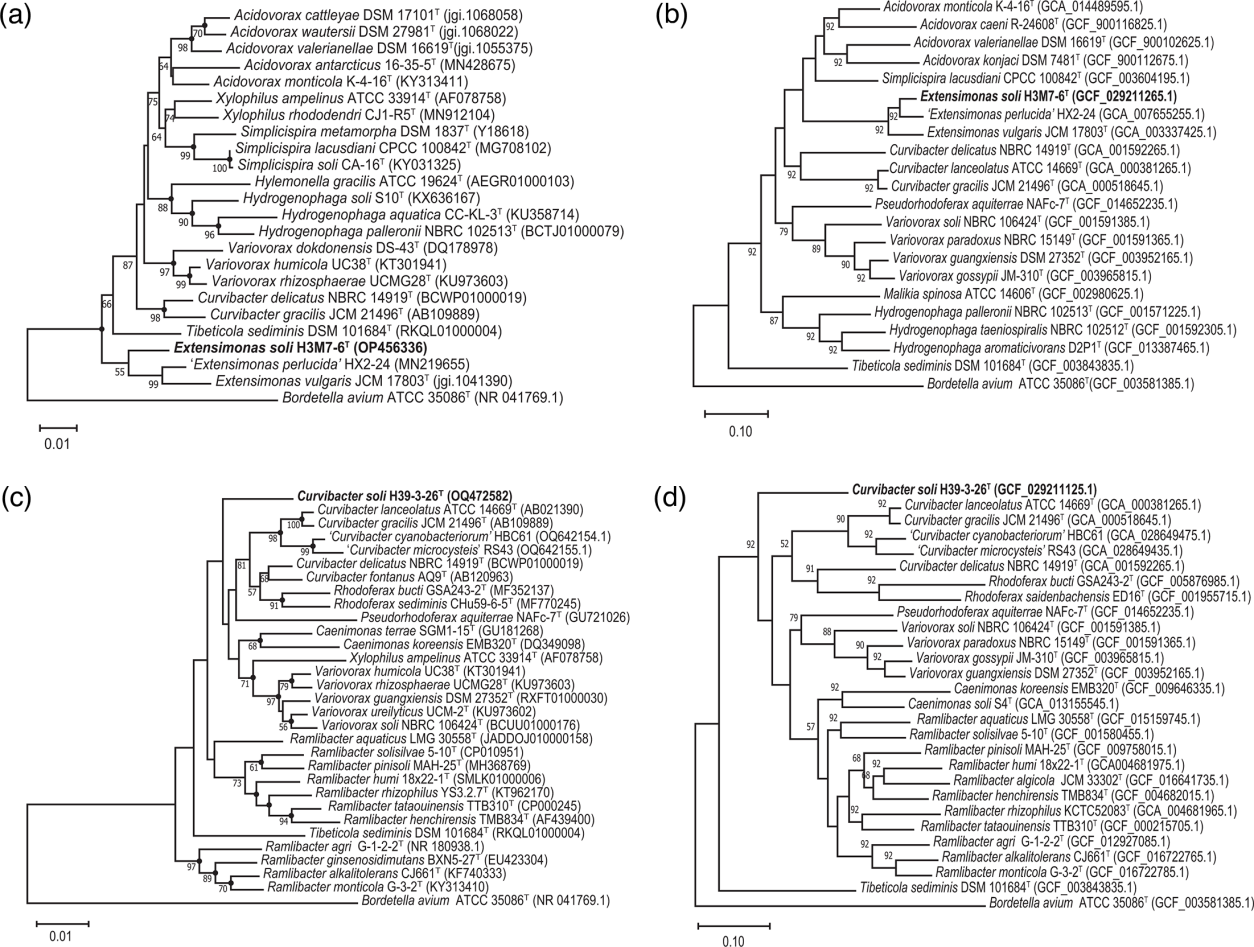
Phylogenetic and phylogenomic trees of strains H3M7-6^T^ and H39-3-26^T^ showed the relationships with their closely related type strains. Phylogenetic (**a**) and phylogenomic (**b**) trees of strain H3M7-6^T^ and its closely related species in the family *Comamonadaceae*; phylogenetic (**c**) and phylogenomic (**d**) trees of strain H39-3-26^T^ and its closely related species in the family *Comamonadaceae*. The DNA sequence of *Bordetella avium* ATCC 35086^T^ was used as the outgroup. The trees were constructed using the NJ algorithm. Phylogenetic trees were also constructed using ML and MP methods with 1000 bootstraps (see Fig. **S3**). Phylogenomic trees were also constructed using the IPGA pipeline (see Fig. **S4**). Filled circles indicate the nodes supported by all three methods regardless of bootstrap percentages. GenBank accession numbers are given in parentheses. Bootstrap percentages (>50%) based on 1000 replicates are shown at the nodes. Bar, 0.01 substitutions per nt position for (**a) and (c)**; bar, 0.1 substitutions per nt position for (**b) and (d)**.

Phylogenetic analysis revealed that strain H3M7-6^T^ clustered with members of the genus *Extensimonas* (Fig. 4a, b). DNA molecular characterization exhibited a G+C content of 65.3 mol%, which lies within the range (64.4–68.8 mol%) for the genus *Extensimonas*. This genus includes only two species. The 16S rRNA gene identity between H3M7-6^T^ and the validly named *E. vulgaris* JCM 17803^T^ was 96.89%, and it was 97.38% for the not validly published '*Extensimonas perlucida*' HX2-24. The ANI and dDDH values between H3M7-6^T^ and *E. vulgaris* JCM 17803^T^ were 91.7 and 45.3%, respectively, and they were 92.3 and 47.0% for '*E. perlucida*' HX2-24 (Table 2, Fig. 3c), all falling below the threshold for differentiating two species. These values support the classification of strain H3M7-6^T^ as a novel species within the genus *Extensimonas*.

Chemotaxonomically, the major cellular fatty acids of H3M7-6^T^ were C_16 : 0_ (34.8%), cyclo-C_17 : 0_ (13.1%), C_18 : 1_ ω7c (11.4%) and summed feature 3 (C_16 : 1_ ω7c/C_16 : 1_ ω6c, 20.2%). This composition was similar to that of *E. vulgaris* JCM 17803^T^ (Table S2). The polar lipids observed in strain H3M7-6^T^, including DPG, PE and PG (Fig. S1), were consistent with those of *E. vulgaris* JCM 17803^T^. However, H3M7-6^T^ also contained some unknown components, including an APL, an AL, PLs and GLs, which were not observed in *E. vulgaris* JCM 17803^T^. These additional polar lipids differentiate strain H3M7-6^T^ from *E. vulgaris* JCM 17803^T^.

The analysis of physiological properties revealed that strain H3M7-6^T^ was positive for nitrate reduction to nitrite, Tween 80 hydrolysis and enzyme activities of alkaline phosphatase, esterase (C4), esterase lipase (C8), acid phosphatase, naphthol-AS-BI-phosphohydrolase and catalase (Table 3). These features were also observed in *E. vulgaris* JCM 17803^T^. However, *E. vulgaris* JCM 17803^T^ exhibited positive reactions for the enzyme activities of leucine arylamidase, valine arylamidase and cystine arylamidase and hydrolysis of gluconic acid, which were different from those of H3M7-6^T^. Strain H3M7-6^T^ did not assimilate any carbon sources from the API (20NE). It was negative for H_2_S production, indole production and hydrolysis of starch, casein and urea, as well as fumaric acid metabolism, V–P and methyl red tests.

Based on the results of phenotypic characterization, phylogenetic and phylogenomic analyses, we conclude that strain H3M7-6^T^ represents a novel species affiliated with the genus *Extensimonas*, and we propose the name *Extensimonas soli* sp. nov.

#### Strain H39-3-26^T^

Strain H39-3-26^T^ was obtained from M3 agar plates. Growth was observed after it was re-streaked onto the R2A agar plate, and it formed visible colonies in 2 days. The colonies are circular, smooth, convex and transparent. Cells of strain H39-3-26^T^ were Gram-negative, rod-shaped (1.52–3.02 µm in length and 0.46–0.60 µm in width) and aerobic and did not grow under anaerobic conditions. Endospores were not formed, and polar flagella were observed on the cell surfaces (Table 1, Fig. 1).

Based on 16S rRNA gene sequence alignment, strain H39-3-26^T^ demonstrated the highest similarity with species of the genus *Curvibacter*, including *C. gracilis* JCM 21496^T^ (97.25%), *C. lanceolatus* ATCC 14669^T^ (97.31%) and *C. delicatus* NBRC 14919^T^ (97.04%). Phylogenetic analysis was further carried out to determine its relationship with closely related species. The closely related species constructed in the phylogenetic tree were all from the family *Comamonadaceae*, and they showed close 16S rRNA gene similarities with H39-3-26^T^, ranging from 96.16 to 97.31%. Both the phylogenetic and phylogenomic trees revealed that H39-3-26^T^ had a close relationship with the genus *Curvibacter*, though they did not cluster in a clade (Fig. 4c, d). Additionally, its DNA G+C content (67.2 mol%) is similar to other members of the genus *Curvibacter* (Table 2). Comparisons of their genome sequences showed that the ANI and dDDH values between H39-3-26^T^ and *C. gracilis* JCM 21496^T^ were 77.0 and 21.2%, respectively, and they were 76.9 and 21.2% for *C. lanceolatus* ATCC 14669^T^ (Table 2, Fig. 3d). These values support the classification of H39-3-26^T^ as a novel species of the genus *Curvibacter*.

Chemotaxonomically, both H39-3-26^T^ and *C. gracilis* JCM 21496^T^ had C_16 : 0_, cyclo-C_17 : 0_, C_18 : 1_ ω7c and summed feature 3 (C_16 : 1_ ω7c/C_16 : 1_ ω6c) as major cellular fatty acids (Table S2). However, strain H39-3-26^T^ also contained 5.3% of C_12 : 0_ 2-OH, which was not detected in *C. gracilis* JCM 21496^T^. The results indicate individual differences in the fatty acid composition, although the content of most components is similar. Regarding polar lipids, strain H39-3-26^T^ contained DPG, PE, PG, APLs and Ls (Fig. S1).

Physiologically, strains H39-3-26^T^ and *C. gracilis* JCM 21496^T^ displayed different properties. Strain H39-3-26^T^ was able to grow on fumaric acid, while *C. gracilis* JCM 21496^T^ was not. Conversely, *C. gracilis* JCM 21496^T^ showed positive results for nitrate reduction to nitrite, V–P and methyl red tests, assimilation of d-glucose and gluconic acid and enzyme activities of cystine arylamidase, *α*-galactosidase and catalase, whereas strain H39-3-26^T^ was negative for these tests (Table 3). Additionally, strain H39-3-26^T^ also showed positive results for H_2_S production, Tween 80 and urea hydrolysis and enzyme activities of alkaline phosphatase, esterase (C4), esterase lipase (C8), leucine arylamidase, valine arylamidase, acid phosphatase and naphthol-AS-BI-phosphohydrolase but was negative for indole production. It did not assimilate any carbon sources from the API 20NE.

Based on the results of phenotypic characterization and phylogenetic and phylogenomic analyses, we propose that strain H39-3-26^T^ represents a novel species affiliated with the genus *Curvibacter*, and we propose the name *Curvibacter soli* sp. nov.

### Genome features and genomic annotation of the four bacterial strains

The genomes of the four bacterial strains were sequenced and annotated. Genome examination using CheckM v1.1 software [59] indicated that the assembled genomes were free from contamination, with genome completeness approaching 99%. All strains possessed 1 5S rRNA gene, 1 16S rRNA gene, 1 23S rRNA gene and ~50 tRNA genes, except for strain H3Y2-7^T^, which had three 5S rRNA genes (Table S3). The genome sizes of strains H3Y2-7^T^, H3SJ34-1^T^, H3M7-6^T^ and H39-3-26^T^ were 4.6, 7.2, 3.4 and 3.4 Mbp, respectively. Their DNA G+C molar contents were 65.2, 62.3, 65.3 and 67.2%, and a total of 4287, 6730, 3144 and 3126 genes were annotated from their respective genomes. The lengths of the 16S rRNA gene sequences obtained by Sanger sequencing were 1309, 1472, 1413 and 1408 bp, respectively. The similarity between 16S rRNA gene sequences obtained from the genome and those from Sanger sequencing was greater than 99.7%, indicating that the sequences determined by the two methods were consistent. All strains contained plasmids. An interpretation of genome annotation related to their physiology, such as carbon source assimilation, resistance to heavy metals and antibacterial agents and biodegradation of aromatic compounds, is described below.

### Assimilation of carbon sources and response to chemical sensitivity assays

**Fig. 5.**
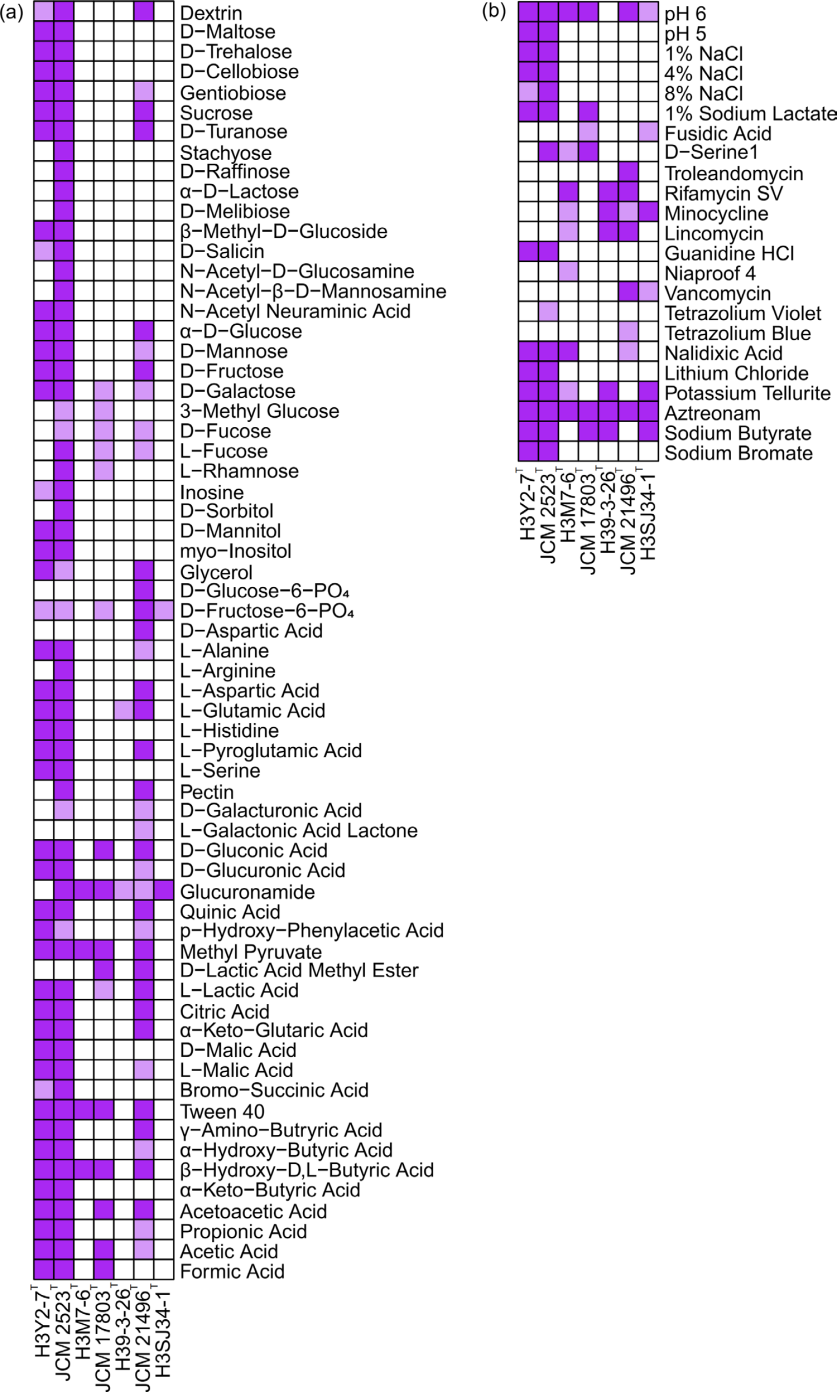
Profile of carbon source assimilation (**a**) and responses to chemical sensitivity assays (**b**) for the four bacterial species and closely related species illustrated their ability to metabolize various carbon sources and resist antibacterial substance. Deep purple indicates strong positive, light purple indicates weak positive and white indicates negative.

The carbon source metabolism profiles of the four strains were further explored using Biolog^™^ GEN III microplates. The results of 71 carbon source assimilations and 23 chemical sensitivity assays are presented in Fig. 5. The four bacterial strains exhibited varying abilities to metabolize carbon sources and resist antibacterial agents. Strain H3Y2-7^T^ demonstrated a more robust capacity in metabolizing carbon sources compared to H3SJ34-1^T^, H3M7-6^T^ and H39-3-26^T^. It utilized a total of 45 carbon sources, including 4 monosaccharides, 6 disaccharides, 1 oligosaccharide, 6 amino acids and 14 organic acids. In contrast, H3SJ34-1^T^, H3M7-6^T^ and H39-3-26^T^ only utilized two (d-fructose-6-PO4 and glucuronamide), four (glucuronamide, methyl pyruvate, Tween 40 and *β*-hydroxy-d, l-butyric acid) and two (l-glutamic acid and glucuronamide) kinds of carbon sources, respectively (Fig. 5a). These findings align with the genome annotation results, showing that strain H3Y2-7^T^ possesses 59, 35 and 28 genes for metabolizing monosaccharides, di-/oligosaccharides and organic acids, respectively, while the other strains only have 2–10 related genes (Table S4). Notably, H3SJ34-1^T^ was annotated with 24 genes for metabolizing disaccharides and oligosaccharides and 9 for monosaccharides, yet it did not utilize saccharides on the Biolog^™^ GEN III microplate, except for the two derivatives of saccharides, d-fructose-6-PO_4_ and glucuronamide. This discrepancy may be due to the nutritional components of the medium influencing carbon source metabolism [60]. Additionally, viruses could also affect host cell metabolism by affecting carbon, nitrogen, phosphorus and sulphur cycles [61]. Based on predicted virus sequences, 17, 32 and 20 homologues of prophages were annotated for H3SJ34-1^T^, H3M7-6^T^ and H39-3-26^T^, respectively (Table S4). In contrast, no prophage was predicted in strain H3Y2-7^T^. These observations are consistent with the metabolic properties described above, with H3Y2-7^T^ metabolizing significantly more carbon sources than the others. Further studies are needed to explore suitable growth conditions for H3SJ34-1^T^, H3M7-6^T^ and H39-3-26^T^.

Comparing the metabolism profiles with close relatives revealed different carbon source utilizations. Several carbon sources that were negative for H3Y2-7^T^, including stachyose, d-raffinose, *α*-d-lactose, d-melibiose, *N*-acetyl-d-glucosamine, *N*-acetyl-*β*-d-mannosamine, 3-methyl glucose, d-fucose, l-fucose, l-rhamnose, d-sorbitol, l-arginine, d-galacturonic acid and glucuronamide, were positive for *P. polychromogenes* JCM 2523^T^. Additionally, strain H3Y2-7^T^ also displayed opposite phenotypes compared to its closest relative, *P. psychrotolerans* YJ56^T^, in 15 kinds of carbon source utilization, including sucrose, stachyose, d-raffinose, *α*-d-lactose, d-melibiose, d-salicin, *N*-acetyl-d-glucosamine, d-sorbitol, d-fructose-6-PO4, d-serine, *p*-hydroxy-phenylacetic acid, methyl pyruvate, Tween 40, *α*-keto-butyric acid and formic acid. For H3M7-6^T^, 11 carbon sources were less metabolized than those of its closest relative, *E. vulgaris* JCM 17803^T^. Strain H39-3-26^T^ metabolized 23 fewer carbon sources than its closest relative, *C. gracilis* JCM 21496^T^. These distinct phenotypic characteristics also support the phylogenetic classifications of these strains described above.

The chemical sensitivity assay revealed that the four bacterial strains resisted six to eight antibacterial substances, including potassium tellurite and aztreonam, but did not tolerate troleandomycin, tetrazolium violet and tetrazolium blue (Fig. 5b). Strain H3Y2-7^T^ grew at pH 5.0 and 1–8% NaCl, while strains H3M7-6^T^, H3SJ34-1^T^ and H39-3-26^T^ could not grow under these conditions, indicating that H3Y2-7^T^ was more tolerant to acidity and salinity than the others. Both H3Y2-7^T^ and *P. polychromogenes* JCM 2523^T^ showed growth while resisting 1% sodium lactate, guanidine HCl, lithium chloride, sodium butyrate and sodium bromate; however, *P. psychrotolerans* YJ56^T^ did not tolerate these chemicals. Strain H3SJ34-1^T^ tolerated fusidic acid, minocycline, vancomycin, potassium tellurite, aztreonam and sodium butyrate. Strain H3M7-6^T^ tolerated d-serine, rifamycin SV, minocycline, lincomycin, niaproof 4, nalidixic acid, potassium tellurites and aztreonam, while strain H39-3-26^T^ tolerated rifamycin SV, minocycline, lincomycin, potassium tellurite, aztreonam and sodium butyrate. Strains H3M7-6^T^ and H39-3-26^T^ also exhibited different resistance properties compared to their respective closest relatives, *E. vulgaris* JCM 17803^T^ and *C. gracilis* JCM 21496^T^, respectively. According to the genome annotations, all four strains were annotated with two or three genes for resisting fluoroquinolones [62] (Table S4), but only H3Y2-7^T^ and H3M7-6^T^ showed growth in the presence of nalidixic acid [63]. Genes encoding for *β*-lactamase [64] were annotated in the genomes of H3Y2-7^T^, H3SJ34-1^T^ and H39-3-26^T^, explaining their resistance to aztreonam.

### Heavy metal resistance and aromatic compound metabolism

**Fig. 6.**
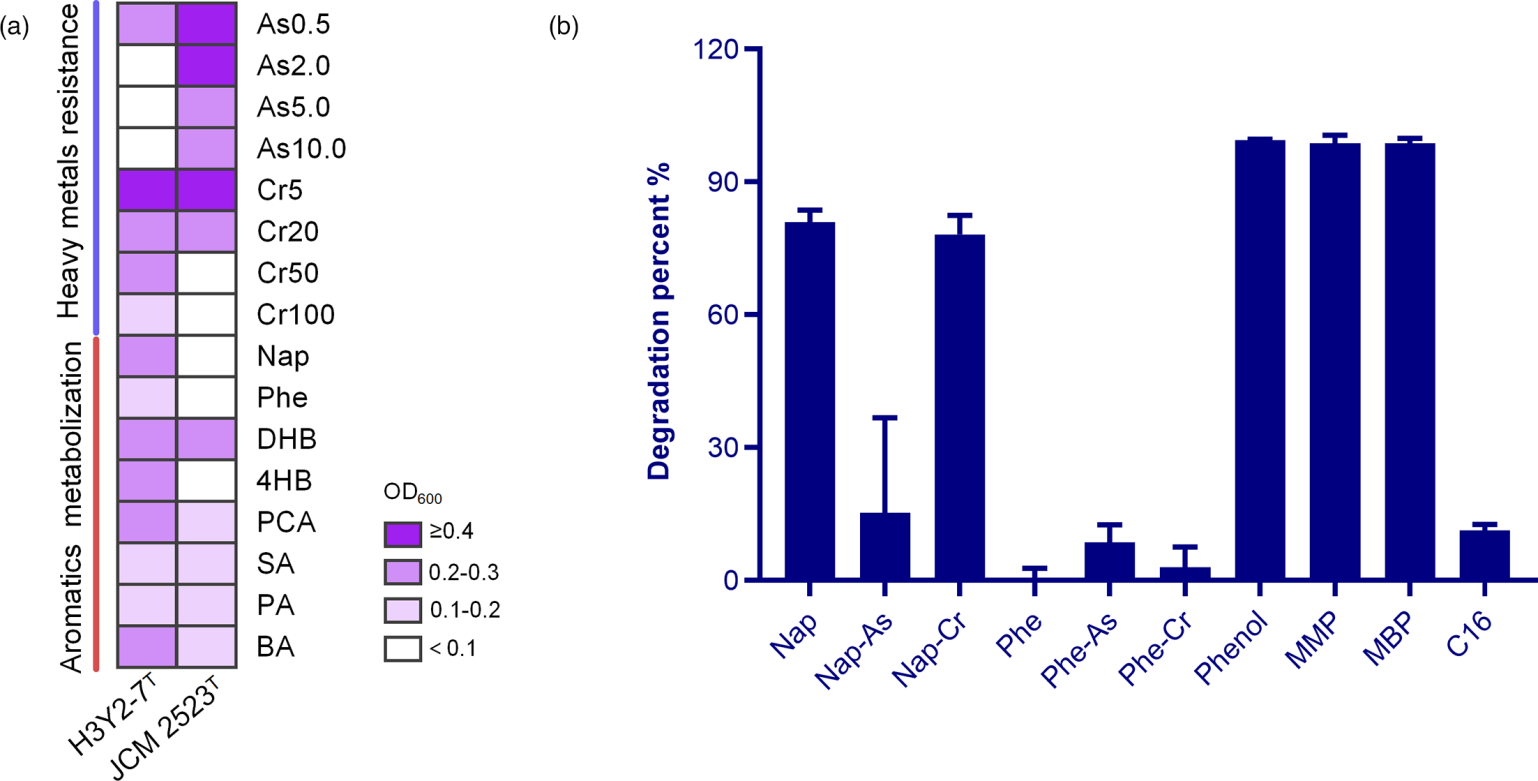
Growth of strain H3Y2-7^T^ under heavy metal resistance and aromatic compound metabolism (**a**) and degradation of PAHs (**b**) demonstrated the strain’s capacity to resist and degrade pollutants. For panel (**a)**, As0.5, As2.0, As5.0 and As10.0 indicate the R2A medium containing 0.5, 2.0, 5.0 and 10.0 mM sodium arsenite, respectively; Cr5, Cr20, Cr50 and Cr100 indicate the R2A medium containing 5, 20, 50 and 100 mg l^−1^ potassium dichromate, respectively. Growth was considered significant if the OD_600_ value increased by at least 0.1 [25]. Deep purple indicates the growth OD_600_≥0.4, purple indicates OD_600_ 0.2–0.3, light purple indicates OD_600_ 0.1–0.2 and white indicates OD_600_<0.1. For panel (**b)**, Nap-As and Phe-As indicate MSM medium for PAH degradation containing 0.5 mM sodium arsenite; Nap-Cr and Phe-Cr indicate MSM medium for PAH degradation containing 5 mg l^−1^ potassium dichromate.

As the four bacterial strains were isolated from polluted soil with PAHs and heavy metals, we tested their abilities to resist heavy metals and degrade PAHs. Results indicated that strain H3Y2-7^T^ could resist arsenite and dichromate and metabolize aromatic compounds, including Nap. In contrast, strains H3SJ34-1^T^, H3M7-6^T^ and H39-3-26^T^ showed no growth in either test (data not shown). Strain H3Y2-7^T^ tolerated 0.5 mM arsenite and 100 mg l^−1^ of potassium dichromate and metabolized Nap, Phe, DHB, 4HB, PCA, SA, PA and BA (Fig. 6a). In contrast, *P. polychromogenes* JCM 2523^T^ had a greater resistance to arsenite than H3Y2-7^T^ but less tolerance to dichromate. It tolerated as high as 10 mM arsenite but did not tolerate above 50 mg l^−1^ dichromate. It could not utilize Nap, Phe and 4HB for growth. Given H3Y2-7^T^’s ability to metabolize PAHs and resist heavy metals, we further tested its degradation of Nap and Phe in the presence of arsenite or dichromate. The results showed that H3Y2-7^T^ degraded 80.9 and 78.1% of Nap in the presence and absence of dichromate, respectively, and weakly degraded Phe (Fig. 6b). Nevertheless, we observed that arsenite significantly impeded the Nap degradation (only 15.2%).

Members of the *Pseudarthrobacter* genus are known to degrade various organic pollutants, including phenol, phthalate esters and alkane [9,11]. In view of these functions possessed by this species, we further examined the degradation of phenol, MMP, MBP and C16 with strain H3Y2-7^T^. The results showed that H3Y2-7^T^ nearly completely degraded 250 mg l^−1^ phenol (99.5%), 400 mg l^−1^ of MMP (98.7%) and MBP (98.8%) after 4 days of cultivation and degraded 11.3% of C16 after 10 days of cultivation (Fig. 6b). According to these examinations, this study highlights the species of *Pseudarthrobacter* represented by strain H3Y2-7^T^ as a potent degrader for complex pollutants.

Moreover, an interpretation of functional genes was carried out to facilitate the understanding of the phenotypic characteristics on the basis of the genomic annotation. The genomic annotations revealed that strain H3Y2-7^T^ possesses the gene homologues for *ars*A and *ars*B, which are involved in arsenite resistance [65] (Table S5), as well as *chr*R, which is responsible for dichromium resistance [66]. These gene homologues probably contribute to its heavy metal resistance phenotype. Although Nap-1,2-dioxygenase, a critical enzyme for the first catalytic reaction step of PAH degradation [67], was not annotated, H3Y2-7^T^ displayed significant growth on all tested aromatic compounds and a well-established ability to degrade Nap. It is speculated that strain H3Y2-7^T^ probably degrades Nap using cytochrome P450, as four gene homologues encoding for this enzyme were annotated (Table S5). Cytochrome P450 is a kind of catalase targeting to oxidize a broad spectrum of substrate [68]. The oxidation of PAHs by CYP450 enzymes has been extensively investigated. This degradation approach was employed by many bacterial species, such as *Bacillus thuringiensis* [69], *Mycobacterium vanbaalenii* [70] and *Rhodococcus* sp. [71]. However, more molecular biology experiments were needed to confirm the PAH degradation mechanism of H3Y2-7^T^ in the future work. Overall, these findings reflect H3Y2-7^T^’s adaptation to a polluted soil environment.

## Description of four novel species

### *Pseudarthrobacter naphthalenicus* sp. nov.

*Pseudarthrobacter naphthalenicus* (naph.tha.le’ ni.cus. N.L. masc. adj. *naphthalenicus*, pertaining to Nap).

Cells are Gram-positive, aerobic and short rods with a size of ~0.93–1.70×0.63–0.85 µm. Colonies grown on R2A agar plates are white, smooth, convex and non-transparent. Cells metabolize d-maltose, d-trehalose, d-cellobiose, gentiobiose, sucrose, d-turanose, *β*-methyl-d-glucoside, *N*-acetyl neuraminic acid, *α*-d-glucose, d-mannose, d-fructose, d-galactose, d-mannitol, myo-inositol, glycerol, l-alanine, l-aspartic acid, l-glutamic acid, l-histidine, l-pyroglutamic acid, l-serine, d-gluconic acid, d-glucuronic acid, quinic acid, *p*-hydroxy-phenylacetic acid, methyl pyruvate, l-lactic acid, citric acid, *α*-keto-glutaric acid, d-malic acid, l-malic acid, Tween 40, *γ*-amino-butryric acid, *α*-hydroxy-butyric acid, *β*-hydroxy-d, l-butyric acid, *α*-keto-butyric acid, acetoacetic acid, propionic acid, acetic acid, formic acid, dextrin, d-salicin, inosine, d-fructose-6-PO_4_, bromo-succinic acid, DHB, 4HB, fumaric acid, PCA, SA, PA and BA. Cells grow at pH 5.0–6.0 and tolerate 1–8% NaCl. Hydrolysis of urea and casein, reduction of nitrate to nitrite and H_2_S production are detected. They have alkaline phosphatase, esterase (C4), leucine arylamidase, valine arylamidase, cystine arylamidase, acid phosphatase, naphthol-AS-BI-phosphohydrolase, *β*-galactosidase, *β*-glucuronidase, *α*-glucosidase, *α*-mannosidase and catalase activities. The major cellular fatty acids are anteiso-C_15 : 0_, iso-C_15 : 0_ and anteiso-C_17 : 0_. Major polar lipids are DPG, PI, GLs and L. The genome size is 4.6 Mbp, and the G+C content is 65.2 mol%.

The type strain is H3Y2-7^T^ (=JCM 36482^T^=CGMCC 1.61323^T^), isolated from polluted soil. The GenBank accession numbers for the 16S rRNA gene sequence and genome sequence of the type strain are OP493229 and JARESG000000000, respectively.

### *Terripilifer* gen. nov.

*Terripilifer* (Ter.ri.pi’li.fer. L. fem. n. *terra*, earth; L. masc. n. *pilus*, a hair; L. suff. *-fer*, carrying, bearing; N.L. masc. n. *Terripilifer*, a bearer of pili from soil).

Cells are Gram-negative, aerobic, ovoid-shaped and motile via a polar flagellum. The major cellular fatty acids include C_16 : 0_ and summed feature 7 (C_19 : 1_ ω7c/C_19 : 1_ ω6c). The major polar lipids are DPG, PE, PG, PC, PI, GLs and PL. The genome size is ~7.2 Mbp, and the DNA G+C content is 62.3 mol%. This genus belongs to the family *Roseiarcaceae*. The type species of the genus is *T. ovatus*.

### *Terripilifer ovatus* sp. nov.

*Terripilifer ovatus* (o.va’tus. L. masc. adj. *ovatus*, egg-shaped).

Cells are Gram-negative, aerobic, ovoid-shaped with a size of ~0.88–1.98×0.67–0.97 µm and motile by means of a polar flagellum. Colonies grown on R2A agar plates are white, smooth, convex and circular. Cells metabolize d-fructose-6-PO_4_ and glucuronamide. They grow at pH 6.0 but do not exhibit growth when the pH is lower than pH 5.0. The strain does not tolerate 1–8% NaCl. Urea hydrolysis and nitrate-to-nitrite reduction are detected. They have alkaline phosphatase, esterase (C4), esterase lipase (C8), leucine arylamidase, valine arylamidase, cystine arylamidase, acid phosphatase and naphthol-AS-BI-phosphohydrolase activities. The dominant fatty acids are C_16 : 0_, C_18 : 0_, C_15 : 0_ 3-OH, C_18 : 1_ ω7c and summed feature 7 (C_19 : 1_ ω7c/C_19 : 1_ ω6c). Major polar lipids include DPG, PG, PE, PI, PC, GLs and PL. The genome size is 7.2 Mbp, and the DNA G+C content is 62.3 mol%.

The type strain is H3SJ34-1^T^ (=JCM 36465^T^=CGMCC 1.61333^T^), isolated from polluted soil. The GenBank accession numbers for the 16S rRNA gene sequence and genome sequence of the type strain are OQ543116 and JARHVB000000000, respectively.

### *Extensimonas soli* sp. nov.

*Extensimonas soli* (so'li. L. gen. n. *soli*, of soil, the source of the type strain).

Cells are Gram-negative, aerobic and motile via a polar flagellum, presenting as short rods with a size of ~1.72–2.29×0.96–1.06 µm. Colonies grown on R2A agar plates are circular, smooth, convex and transparent. Cells metabolize glucuronamide, methyl pyruvate, Tween 40 and *β*-hydroxy-d, l-butyric acid. It grows at pH 6.0 but does not show growth when the pH is lower than pH 5.0. It does not tolerate 1–8% NaCl. Tween 80 hydrolysis and nitrate-to-nitrite reduction are detected. They have alkaline phosphatase, esterase (C4), esterase lipase (C8), acid phosphatase, naphthol-AS-BI-phosphohydrolase and catalase activities. The major fatty acids include C_16 : 0_, cyclo-C_17 : 0_, summed feature 3 (C_16 : 1_ ω7c/C_16 : 1_ ω6c) and C_18 : 1_ ω7c. The major polar lipids comprise DPG, PE, PG, APL, AL, PLs and GLs. The genome size is 3.4 Mbp, and the DNA G+C content is 65.3 mol%.

The type strain is H3M7-6^T^ (=JCM 36176^T^=CGMCC 1.61336^T^), isolated from polluted soil. The GenBank accession numbers for the 16S rRNA gene sequence and genome sequence of the type strain are OP456336 and JARGEL000000000, respectively.

### *Curvibacter soli* sp. nov.

*Curvibacter soli* (so'li. L. gen. n. *soli*, of soil, the source of the type strain).

Cells are Gram-negative, aerobic, rod-shaped with a size of ~1.52–3.02×0.46–0.60 µm and motile by means of polar flagellum. Colonies grown on R2A agar plates are circular, smooth, convex and transparent. Cells metabolize l-glutamic acid, glucuronamide and fumaric acid. They do not grow at pH 5.0–6.0. The strain does not tolerate 1–8% NaCl. H_2_S production and hydrolysis of Tween 80 and urea are detected. They have alkaline phosphatase, esterase (C4), esterase lipase (C8), leucine arylamidase, valine arylamidase, acid phosphatase and naphthol-AS-BI-phosphohydrolase activities. Dominant fatty acids include C_16 : 0_, cyclo-C_17 : 0_, C_18 : 1_ ω7c, summed feature 3 (C_16 : 1_ ω7c/C_16 : 1_ ω6c) and C_12 : 0_ 2-OH. The major polar lipids are PE, DPG, PG, APLs and Ls. The genome size is 3.6 Mbp, and the G+C content is 67.2 mol%.

The type strain is H39-3-26^T^ (=JCM 36178^T^=CGMCC 1.61344^T^), isolated from polluted soil. The GenBank accession numbers for the 16S rRNA gene sequence and genome sequence of the type strain are OQ472582 and JARGEN000000000, respectively.

## supplementary material

10.1099/ijsem.0.006698 Uncited Supplementary Material 1.

## Abbreviations

AGL: unknown aminoglycolipid
AL: unknown aminolipid
ANI: average nucleotide identity
APL: unknown aminophospholipid
BA: benzoic acid
C16: n-hexadecane
dDDH: digital DNA–DNA hybridization
DHB: 2,5-dihydroxybenzoic acid
DPG: diphosphatidylglycerol
gcType: Global Catalogue of Type Strain
GL: unknown glycolipid
4HB: 4-hydroxybenzoic acid
IPGA: Integrated Prokaryotes Genome and pan-genome Analysis
L: unknown lipid
MBP: mono-butyl phthalate
ML: maximum-likelihood
MMP: mono-methyl phthalate
MP: maximum-parsimony
MSM: minimal salt medium
Nap: naphthalene
NJ: neighbour-joining
NT: nucleotide
PA: 1,2-benzenedicarboxylic acid
PAHs: polycyclic aromatic hydrocarbons
PBS: phosphate buffer
PC: phosphatidylcholine
PCA: 3,4-dihydroxybenzoic acid
PE: phosphatidylethanolamine
PG: phosphatidylglycerol
Phe: phenanthrene
PI: phosphatidylinositol
PL: unknown phospholipid
R2A: Reasoner's 2A
SA: 2-hydroxybenzoic acid
SL: sphingolipid
V–P: Voges–Proskauer
