# Supplementary material for: Curvibacter soli sp. nov., Extensimonas soli sp. nov., Pseudarthrobacter naphthalenicus sp. nov. and Terripilifer ovatus gen. nov., sp. nov., four new species isolated from polluted soil
Source: Int J Syst Evol Microbiol. 2025 Mar 5;75(3):006698. doi: 10.1099/ijsem.0.006698 (PMC11936349; doi:10.1099/ijsem.0.006698)
Supplement: Uncited Supplementary Material 1. [file ijsem-75-06698-s001.pdf]

Supplementary materials for:

Curvibacter soli **sp. nov.,** ***Extensimonas soli*** **sp. nov.,** ***Pseudarthrobacter napthalenicus*** **sp. nov., and** ***Terripilifer ovatus*** **gen. nov., sp. nov., four new species isolated from polluted soil**

Ze-Shen Liu[1], Ke-Huan Wang[1], Xiao-Kang Wang[1], Man Cai[1], Mei-Ling Yang[1], Wen-Ke Yang[1], De-Feng Li*[1], Shuang-Jiang Liu*[1,2]

1. State Key Laboratory of Microbial Resources, Institute of Microbiology, Chinese Academy of Sciences, Beijing 100101, China.

2. State Key Laboratory of Microbial Biotechnology, Shandong University, Qingdao 266237, China.

*Corresponding authors:

De-Feng Li (lidefeng@im.ac.cn) & Shuang-Jiang Liu (liusj@im.ac.cn)

Postal address: Institute of Microbiology, Chinese Academy of Sciences, No. 1 Beichen West Road, Chaoyang District, Beijing 100101, China

Telephone number: +86-010-64807423

Table S1. Cellular fatty acid compositions of strains H3Y2-7T, H3SJ34-1T, and their closely related species.

Strains	1	2	3	4	5	6	7	8
$C_{12:0}$	ND	/	/	ND	1.1	/	/	/
$C_{14:0}$	1.3	1.4	1.7	2.3	4.0	/	/	/
**$C_{16:0}$**	2.8	**6.1**	**8.1**	**9.2**	**27.9**	**8.7**	**11.4**	**9.3**
**$C_{18:0}$**	/	/	/	/	**9.1**	/	/	ND
**iso-$C_{15:0}$**	**15.3**	**10.6**	**7.1**	**9.0**	/	/	/	/
**iso-$C_{16:0}$**	4.0	**5.2**	**7.2**	**6.2**	ND	/	/	/
iso-$C_{17:0}$	1.0	/	/	2.7	ND	/	/	/
iso-$C_{19:0}$	/	/	/	/	1.3	/	/	/
**anteiso-$C_{15:0}$**	**59.5**	**52.2**	**51.5**	**46.1**	/	/	/	/
**anteiso-$C_{17:0}$**	**9.0**	**14.8**	**12.7**	**16.5**	/	/	/	/
anteiso-$C_{17:1}$ ω9c	2.4	/	/	2.4	ND	/	/	/
**$C_{15:0}$** 3-OH	ND	/	/	ND	**6.1**	/	/	/
$C_{16:0}$ 3-OH	ND	/	/	ND	3.7	/	3.1	/
cyclo-$C_{19:0}$ ω8c	ND	/	/	ND	ND	/	/	**38.9**
11-methyl-$C_{18:1}$ ω7c	ND	/	/	ND	4.2	/	/	/
**$C_{16:1}$ ω7c**	ND	/	/	ND	ND	**45.4**	**46.8**	/
**$C_{18:1}$ ω7c**	/	/	/	/	**8.9**	**42.2**	**35.0**	**26.3**
$C_{18:1}$ ω9c	ND	/	/	ND	2.2	/	/	/
$C_{20:1}$ ω9c	ND	/	/	ND	1.4	/	/	/
Summed feature 2	ND	/	/	ND	3.9	/	/	/
Summed feature 3	/	2.3	1.2	2.9	4.1	/	/	/
**Summed feature 7**	ND	/	/	ND	**14.4**	/	/	/

Strains: 1, H3Y2-7T; 2, *Pseudarthrobacter psychrotolerans* YJ56T [1]; 3, *Pseudarthrobacter sulfonivorans* ALLT [1, 2]; 4, *Pseudarthrobacter polychromogenes* JCM 2523T (this study); 5, H3SJ34-1T; 6, *Rhodoblastus sphagnicola* DSM 16996T [3]; 7, *Rhodoblastus acidophilus* DSM 137T [3, 4]; 8, *Roseiarcus fermentans* Pf56T [5]. Data for the strains in column 1, 4, and 5 are derived from this study, while data for other strains are referenced from previous studies as indicated. Fatty acids with content proportion ≥1.0% are shown, and those ≥5.0% are considered as major component and indicated in bold. /, not reported; ND, not detected. Summed features are fatty acids that cannot be resolved reliably from another fatty acid using the chromatographic conditions chosen. The MIDI system groups these fatty acids together as one feature with a single percentage of the total. Summed feature 2 represents $C_{14:0}$ 3-OH and/or iso-$C_{16:1}$ I; summed feature 3 represents $C_{16:1}$ ω7c and/or $C_{16:1}$ ω6c; summed feature 7 represents $C_{19:1}$ ω7c and/or $C_{19:1}$ ω6c.

Table S2. Cellular fatty acid compositions of strains H3M7-6^T, H39-3-26^T, and their closely related species.

Strains	1	2	3	4	5	6
$C_{12:0}$	2.9	2.6	4.0	2.1	3.2	3.6
**$C_{16:0}$**	**34.8**	**35.7**	**29.1**	**34.9**	**34.2**	**16.9**
$C_{17:0}$	2.0	2.1	/	/	/	ND
$C_{10:0}$ 3-OH	4.7	4.5	4.6	/	ND	ND
**$C_{12:0}$ 2-OH**	ND	ND	/	**5.3**	ND	/
$C_{14:0}$ 2-OH	1.9	/	ND	/	ND	ND
$C_{16:0}$ 2-OH	/	1.3	1.3	1.2	ND	/
$C_{16:1}$ 2-OH	1.5	/	2.5	3.7	ND	/
$C_{18:1}$ 2-OH	/	1.1	/	/	ND	/
**$C_{18:1}$ ω7c**	**11.4**	**13.3**	**/**	**6.6**	**12.9**	**35.5**
iso-$C_{17:0}$ 3-OH	ND	/	ND	1.0	ND	ND
anteiso-$C_{17:1}$ ω9c	1.5	1.2	ND	1.2	/	ND
**cyclo-$C_{17:0}$**	**13.1**	**11.9**	**24.8**	**14.3**	**9.7**	**ND**
cyclo-$C_{19:0}$ ω8c	/	3.3	2.9	/	ND	/
11-methyl-$C_{18:1}$ ω7c	ND	1.4	ND	ND	/	ND
**Summed feature 3**	**20.2**	**15.5**	**26.9**	**25.0**	**32.9**	**41.9**
Summed feature 7	ND	1.9	1.4	ND	3.2	/

Strains: 1, H3M7-6^T; 2, *Extensimonas vulgaris* JCM 17803^T (this study); 3, *'Extensimonas perlucida'* HX2-24 [6]; 4, H39-3-26^T; 5, *Curvibacter gracilis* JCM 21496^T (this study); 6, *Curvibacter lanceolatus* ATCC 14669^T [7, 8]. Data for the strains in column 1, 2, 4, and 5 are derived from this study, while data for other strains are referenced from previous studies as indicated. Fatty acids with content proportion ≥1.0% are shown, and those ≥5.0% are considered as major component and indicated in bold. /, not reported; ND, not detected. Summed features are fatty acids that cannot be resolved reliably from another fatty acid using the chromatographic conditions chosen. The MIDI system groups these fatty acids together as one feature with a single percentage of the total. Summed feature 3 represents $C_{16:1}$ ω7c and/or $C_{16:1}$ ω6c; summed feature 7 represents $C_{19:1}$ ω7c and/or $C_{19:1}$ ω6c.

Table S3. Genomic features of the four novel species isolated from polluted soil.

Genome features	H3Y2-7^T	H3SJ34-1^T	H3M7-6^T	H39-3-26^T
Genome Size (kbp)	4559.1	7197.9	3438.2	3359.1
G+C comtent (mol %)	65.2	62.3	65.3	67.2
Completeness (%)	99.1	99.7	99.3	98.9
Coverage of the genome (%)	99.9	99.8	99.6	96.3
Gene number	4287	6730	3144	3126
Contamination (%)	0.05	0.73	0	0.47
Number of contigs	90	86	50	99
N50 of contigs (kbp)	178.6	279.2	181.9	82.2
Largest contig (kbp)	452.1	568.8	529.4	177.6
Number of tRNA	52	49	46	49
Number of 5S rRNA	3	1	1	1
Number of 23S rRNA	1	1	1	1
Number of 16SrRNA	1	1	1	1
Number of sRNA	1	8	3	4
Plasimid size (Kbp)	299.7	1024.1	215.1	71
Identity between 16S rRNA gene sequences obtained from genome and Sanger sequencing	99.8	99.7	99.7	100.0

Table S4. Annotated gene number related to antibiotics resistance, carbohydrates and amino acids metabolism, and prophage based on the genomes of the four novel species.

Strains	H3Y2-7^T	H3SJ34-1^T	H3M7-6^T	H39-3-26^T
**Resistance to antibiotics**				
Anti fluoroquinolones	2	3	2	2
Beta-lactamase	1	1	-	1
**Carbohydrates metabolism**				
Central carbohydrate metabolism	139	113	81	73
Monosaccharides	59	9	2	2
Di- and oligo-saccharides	35	24	2	3
Aminosugars	6	-	-	-
**Organic acids**	**28**	**8**	**10**	**6**
**Amino acids and derivatives**	**309**	**357**	**284**	**256**
**Prophage**	**-**	**17**	**32**	**20**
Head protein	-	3	5	5
Tail protein	-	2	5	1
Portal protein	-	-	1	2
Fiber protein	-	1	-	-
Plate protein	-	3	-	-
Regulatory protein	-	-	1	-
Phage-like protein	-	3	6	4
Protease	-	-		1
Integrase	-	-	1	1
Terminase	-	-	1	1
Hypothetical protein	-	5	12	5

The genomes were annotated using RAST engine. -, no related genes were annotated. Prophage sequences were predicted and annotated with PHAEST program.

Table S5. Annotated gene number related to heavy metal resistance and aromatic compound metabolism based on the genomes of the four novel species.

Strains	H3Y2-7^T	H3SJ34-1^T	H3M7-6^T	H39-3-26^T
**Enymes for arsenic resistance**				
ArsA	1	-	-	1
ArsB	1	2	-	1
**Enymes for chromium resistance**				
ChrB	-	1	-	-
ChrR	1	3	1	-
Chromate transporter	-	7	2	1
**Enymes for aromatic compounds metabolism**				
Naphthalene 1,2-dioxygenase	-	2	5	4
Cytochrome P450	4	2	2	1
Peripheral pathways for aromatic compound catabolism	12	28	26	5
Metabolism of central aromatic intermediates	42	75	7	9

The genomes were annotated on the Global Catalogue of Type Strain (gcType) Platform. -, no related genes were annotated.

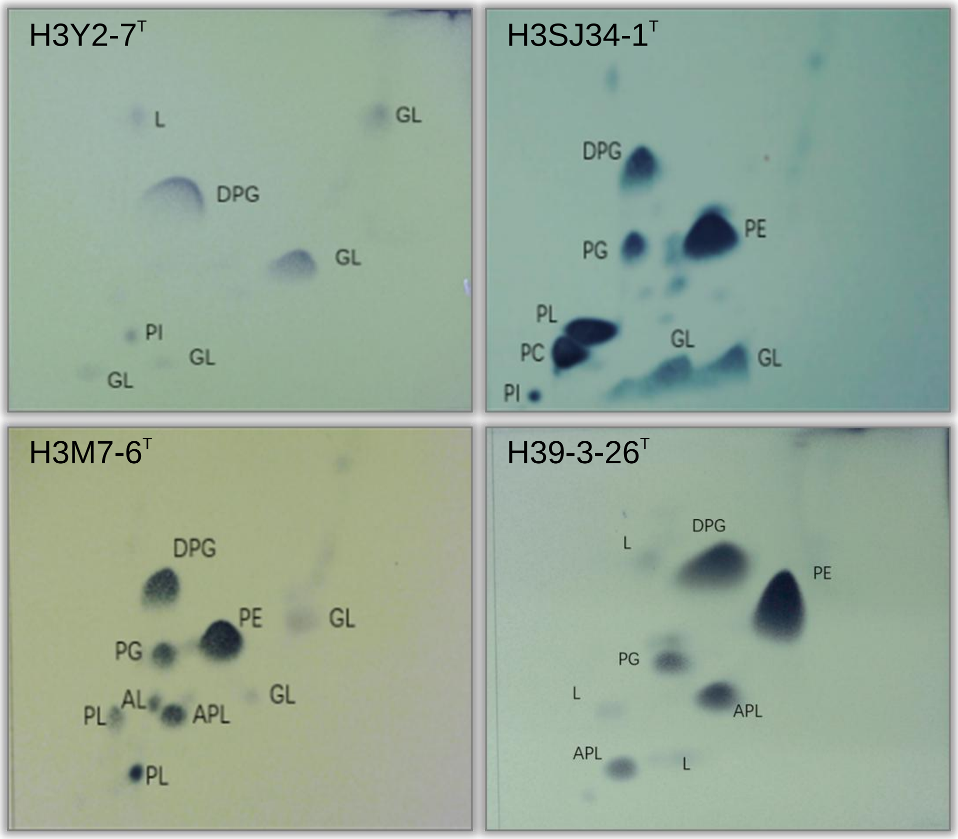

Fig. S1. Polar lipid profiles after separation by two-dimensional thin layer chromatography of the four strains. DPG, diphosphatidylglycerol; PG, phosphatidylglycerol; PE, phosphatidylethanolamine; APL, unknown aminophospholipid; PL, phospholipid; GL, glycolipid; AL, aminolipid; L, unknown lipid; PC , phosphatidylcholine; PI, phosphatidylinositols.

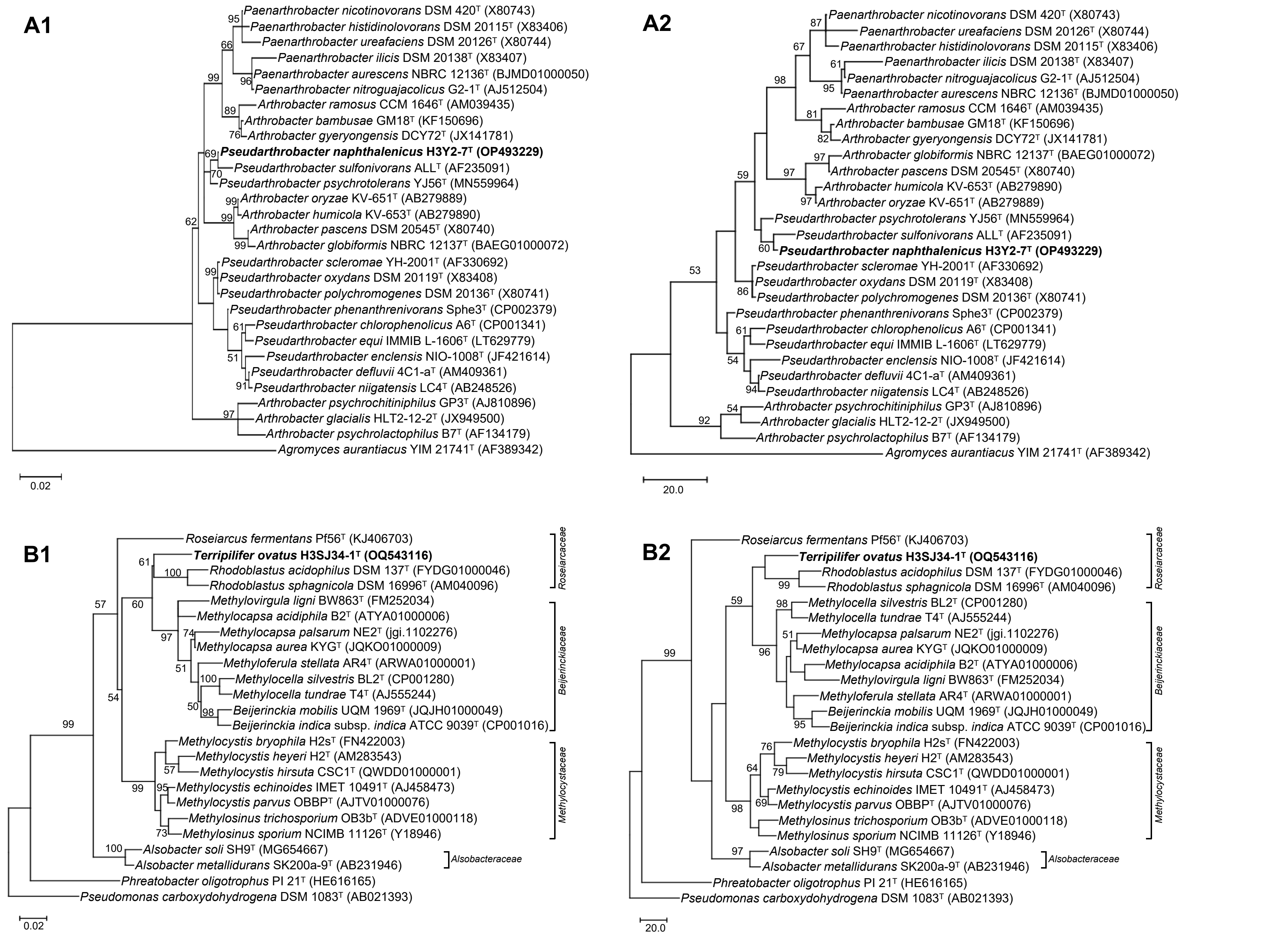

Fig. S2. Maximum-likelihood (**A1**, **B1**) and maximum parsimony (**A2**, **B2**) phylogenetic trees based on 16S rRNA gene sequences show the relationship between strains H3Y2-7^T and H3SJ34-1^T and their closely related microorganisms. **A**, strain H3Y2-7^T and its closely related species in the family *Micrococcaceae*. The sequence of *Agromyces aurantiacus* YIM 21741^T (AF389342) was used as an outgroup; **B**, strain H3SJ34-1^T and its closely related species in the families *Roseiarcaceae*, *Beijerinckiaceae*, *Methylocystaceae* and *Alsobacteraceae*. The sequence of *Pseudomonas carboxydohydrogena* DSM 1083^T (AB021393) and *Phreatobacter oligotrophus* PI 21^T (HE616165) were used as outgroup. GenBank accession numbers are given in parentheses. Bootstrap percentages (>50%) based on 1,000 replicates are shown at the nodes. Bar, 0.02 substitutions per nucleotide position for **A1**and **B1**; Bar, 20.0 substitutions per nucleotide position for **A2**, **B2**.

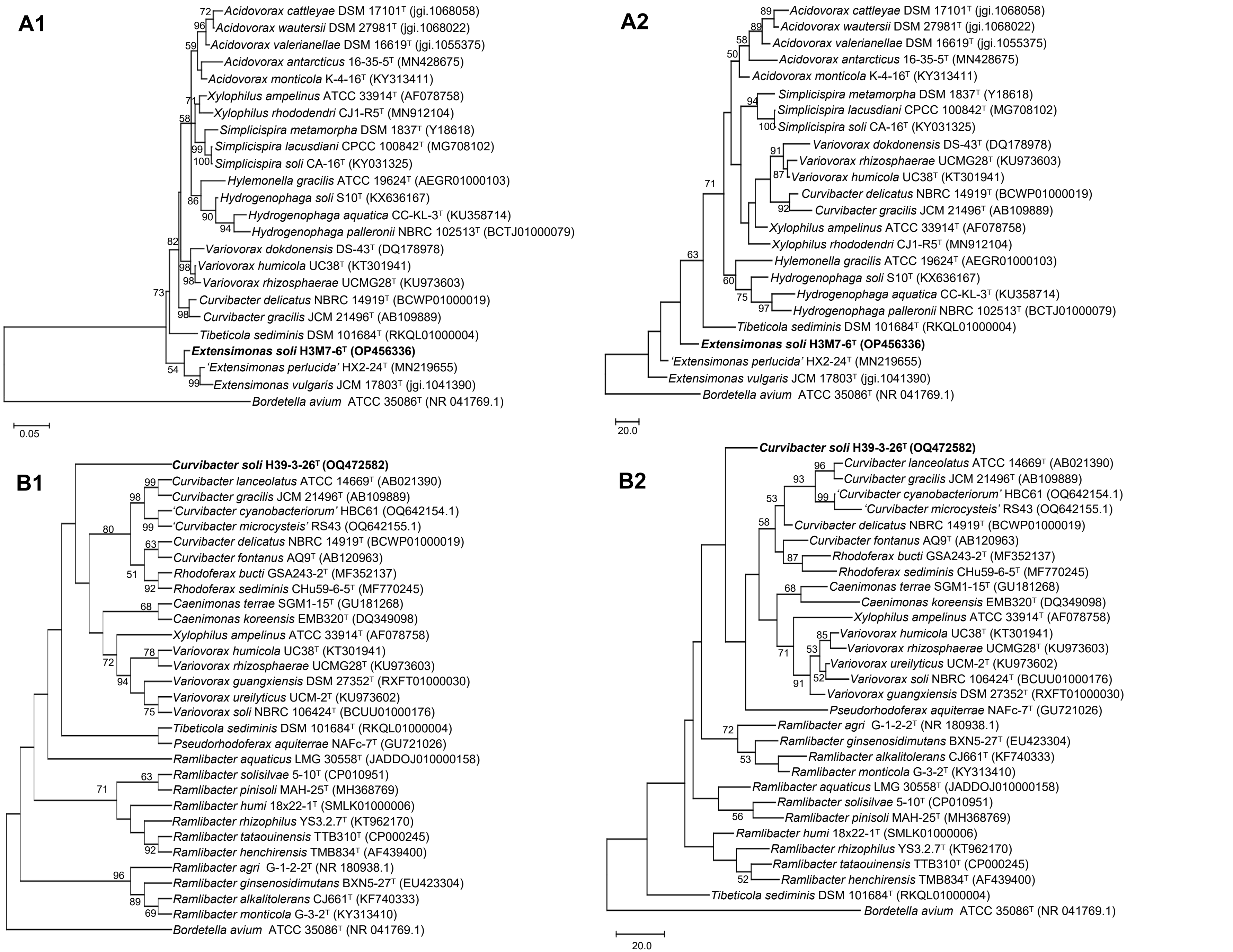

Fig. S3. Maximum-likelihood (**A1**, **B1**) and maximum parsimony (**A2**, **B2**) phylogenetic trees based on 16S rRNA gene sequences show the relationship between strains H3M7-6^T and H39-3-26^T and their closely related microorganisms. **A**, strain H3M7-6^T and its closely related species in the family *Comamonadaceae*. The sequence of *Bordetella avium* ATCC 35086^T (NR 041769.1) was used as outgroup; **B**, strain H39-3-26^T and its closely related species in the family *Comamonadaceae*. The sequence of *Bordetella avium* ATCC 35086^T (NR 041769.1) was used as outgroup. GenBank accession numbers are given in parentheses. Bootstrap percentages (>50%) based on 1,000 replicates are shown at the nodes. Bar, 0.05 substitutions per nucleotide position for **A1**; Bar, 20.0 substitutions per nucleotide position for **A2**, **B2**.

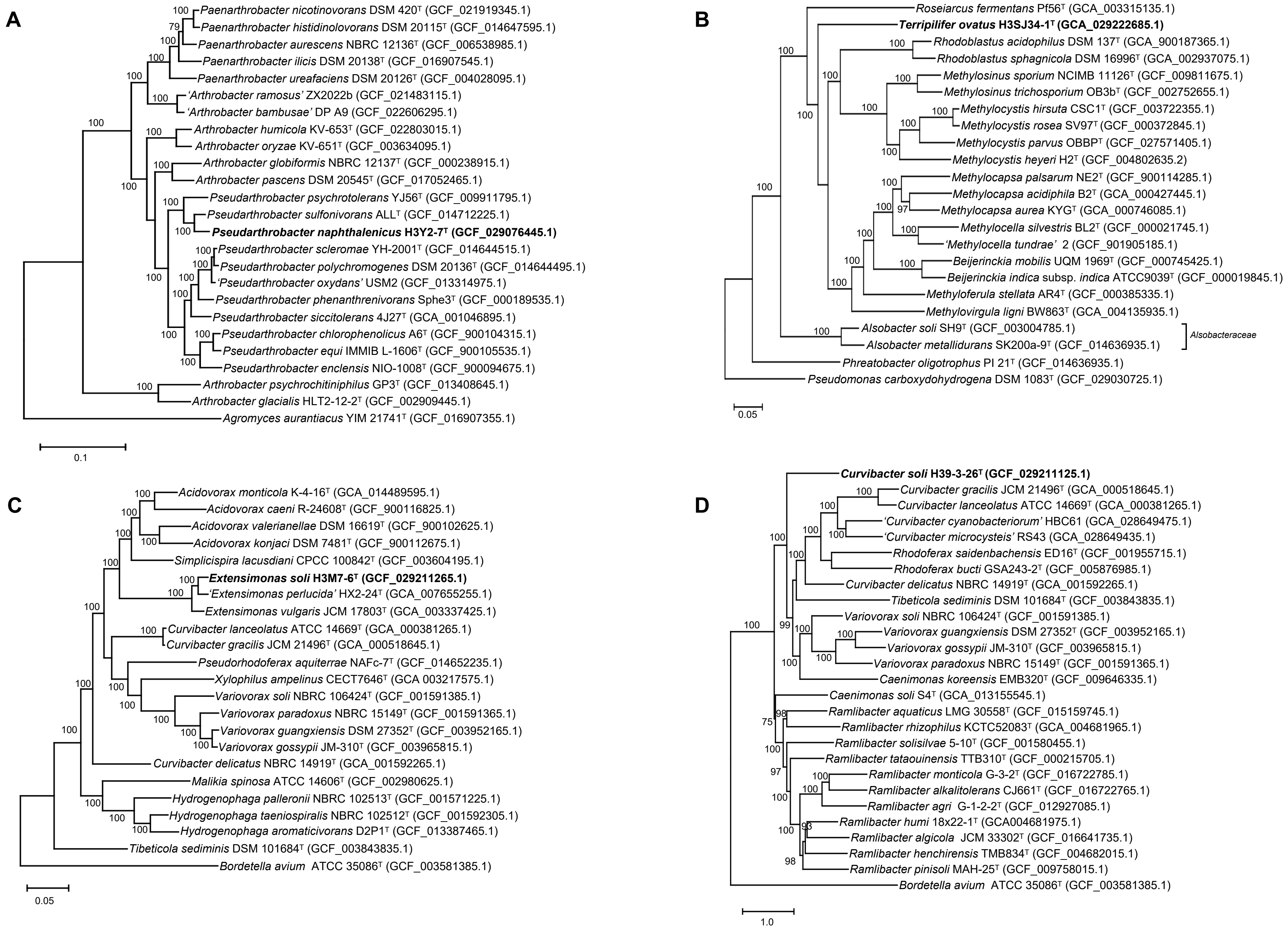

Fig. S4. Phylogenomic trees reconstructed with IPGA based on genome sequences. **A**, strain H3Y2-7^T and its closely related species; **B**, strain H3SJ34-1^T and its closely related species; **C**, strain H3M7-6^T and its closely related species; **D**, strain H339-3-26^T and its closely related species. Bootstrap percentages (>50%) based on 1,000 replicates are shown at the nodes. Bar, 0.1 substitutions per nucleotide position for **A** and **D**; Bar, 0.05 substitutions per nucleotide position for **B** and **C**. The IPGA program was developed by Liu *et al.*, 2022 [9].

References

1. **Shin Y, Lee BH, Lee KE, Park W.** *Pseudarthrobacter psychrotolerans* sp. nov., a cold-adapted bacterium isolated from Antarctic soil. *Int J Syst Evol Microbiol* 2020; 70:6106-6114.
2. **Borodina E, Kelly DP, Schumann P, Rainey FA, Ward-Rainey NL, *et al.*** Enzymes of dimethylsulfone metabolism and the phylogenetic characterization of the facultative methylotrophs *Arthrobacter sulfonivorans* sp. nov., *Arthrobacter methylotrophus* sp. nov., and *Hyphomicrobium sulfonivorans* sp. nov. *Arch Microbiol* 2002; 177:173-183.
3. **Kulichevskaya IS, Guzev VS, Gorlenko VM, Liesack W, Dedysh SN.** *Rhodoblastus sphagnicola* sp. nov., a novel acidophilic purple non-sulfur bacterium from *Sphagnum* peat bog. *Int J Syst Evol Microbiol* 2006; 56:1397-1402.
4. **Imhoff JF.** Transfer of *Rhodopseudomonas acidophila* to the new genus *Rhodoblastus* as *Rhodoblastus acidophilus* gen. nov., comb. nov. *Int J Syst Evol Microbiol* 2001; 51:1863-1866.
5. **Kulichevskaya IS, Danilova OV, Tereshina VM, Kevbrin VV, Dedysh SN.** Descriptions of *Roseiarcus fermentans* gen. nov., sp. nov., a bacteriochlorophyll a-containing fermentative bacterium related phylogenetically to alphaproteobacterial methanotrophs, and of the family *Roseiarcaceae* fam. nov. *Int J Syst Evol Microbiol* 2014; 64:2558-2565.
6. **Peng Q, Sheng M, Yang Z, Ni H, Li Q, *et al.*** *Extensimonas perlucida* sp. nov., a novel bacterium isolated from sludge. *Curr Microbiol* 2020; 77:1316-1320.
7. **Ding L, Yokota A.** *Curvibacter fontana* sp. nov., a microaerobic bacteria isolated from well water. *J Gen Appl Microbiol* 2010; 56:267-271.
8. **Ding L, Yokota A.** Proposals of *Curvibacter gracilis* gen. nov., sp. nov. and *Herbaspirillum putei* sp. nov. for bacterial strains isolated from well water and reclassification of [*Pseudomonas*] *huttiensis*, [*Pseudomonas*] *lanceolata*, [*Aquaspirillum*] *delicatum* and [*Aquaspirillum*] *autotrophicum* as *Herbaspirillum huttiense* comb. nov., *Curvibacter lanceolatus* comb. nov., *Curvibacter delicatus* comb. nov. and *Herbaspirillum autotrophicum* comb. nov. *Int J Syst Evol Microbiol* 2004; 54:2223-2230.
9. **Dongmei L, Yifei ZH, Guomei F, Dingzhong S, Xingjiao ZH, *et al.*** IPGA: a handy integrated prokaryotes genome and pan-genome analysis web service. iMeta 2022; 1:e55.

Supplementary information about the genomic annotation

The annotated genes from the genome of strain H3Y2-7^{T} involving in metabolizaton of carbohydrates, amino acids, and aromatic compounds and resistance to antibiotics and heavy metals.

Category	Subcategory	Subsystem	Annotated genes and its roles
Carbohydrates	Central carbohydrate metabolism	Pentose phosphate pathway	Glucose-6-phosphate 1-dehydrogenase (EC 1.1.1.49)
Carbohydrates	Central carbohydrate metabolism	Pentose phosphate pathway	Transketolase, C-terminal section (EC 2.2.1.1)
Carbohydrates	Central carbohydrate metabolism	Pentose phosphate pathway	Xylulose-5-phosphate phosphoketolase (EC 4.1.2.9)
Carbohydrates	Central carbohydrate metabolism	Pentose phosphate pathway	Fructose-6-phosphate phosphoketolase (EC 4.1.2.22)
Carbohydrates	Central carbohydrate metabolism	Pentose phosphate pathway	6-phosphogluconolactonase (EC 3.1.1.31), eukaryotic type
Carbohydrates	Central carbohydrate metabolism	Pentose phosphate pathway	Ribulose-phosphate 3-epimerase (EC 5.1.3.1)
Carbohydrates	Central carbohydrate metabolism	Pentose phosphate pathway	Transaldolase (EC 2.2.1.2)
Carbohydrates	Central carbohydrate metabolism	Pentose phosphate pathway	Transketolase (EC 2.2.1.1)
Carbohydrates	Central carbohydrate metabolism	Pentose phosphate pathway	6-phosphogluconate dehydrogenase, decarboxylating (EC 1.1.1.44)
Carbohydrates	Central carbohydrate metabolism	Pentose phosphate pathway	Ribose-phosphate pyrophosphokinase (EC 2.7.6.1)
Carbohydrates	Central carbohydrate metabolism	Pentose phosphate pathway	Transketolase, N-terminal section (EC 2.2.1.1)
Carbohydrates	Central carbohydrate metabolism	Dihydroxyacetone kinases	DHA-specific phosphocarrier protein HPr
Carbohydrates	Central carbohydrate metabolism	Dihydroxyacetone kinases	Putative dihydroxyacetone kinase (EC 2.7.1.29), ADP-binding subunit
Carbohydrates	Central carbohydrate metabolism	Dihydroxyacetone kinases	DHA-specific IIA component
Carbohydrates	Central carbohydrate metabolism	Dihydroxyacetone kinases	Phosphoenolpyruvate-dihydroxyacetone phosphotransferase (EC 2.7.1.121), subunit DhaM
Carbohydrates	Central carbohydrate metabolism	Dihydroxyacetone kinases	Phosphoenolpyruvate-dihydroxyacetone phosphotransferase (EC 2.7.1.121), ADP-binding subunit DhaL
Carbohydrates	Central carbohydrate metabolism	Dihydroxyacetone kinases	Dihydroxyacetone kinase, ATP-dependent (EC 2.7.1.29)
Carbohydrates	Central carbohydrate metabolism	Dihydroxyacetone kinases	Phosphoenolpyruvate-dihydroxyacetone phosphotransferase (EC 2.7.1.121), dihydroxyacetone binding subunit DhaK
Carbohydrates	Central carbohydrate metabolism	Pyruvate Alanine Serine Interconversions	L-serine dehydratase, beta subunit (EC 4.3.1.17)
Carbohydrates	Central carbohydrate metabolism	Pyruvate Alanine Serine Interconversions	Alanine dehydrogenase (EC 1.4.1.1)
Carbohydrates	Central carbohydrate metabolism	Pyruvate Alanine Serine Interconversions	Alanine racemase (EC 5.1.1.1)
Carbohydrates	Central carbohydrate metabolism	Pyruvate Alanine Serine Interconversions	L-serine dehydratase, alpha subunit (EC 4.3.1.17)
Carbohydrates	Central carbohydrate metabolism	Pyruvate Alanine Serine Interconversions	D-serine/D-alanine/glycine transporter
Carbohydrates	Central carbohydrate metabolism	Pyruvate Alanine Serine Interconversions	Branched-chain amino acid aminotransferase (EC 2.6.1.42)
Carbohydrates	Central carbohydrate metabolism	Pyruvate metabolism I: anaplerotic reactions, PEP	Phosphoenolpyruvate carboxykinase [GTP] (EC 4.1.1.32)
Carbohydrates	Central carbohydrate metabolism	Pyruvate metabolism I: anaplerotic reactions, PEP	Phosphoenolpyruvate carboxylase (EC 4.1.1.31)
Carbohydrates	Central carbohydrate metabolism	Pyruvate metabolism I: anaplerotic reactions, PEP	Phosphoenolpyruvate synthase (EC 2.7.9.2)
Carbohydrates	Central carbohydrate metabolism	Pyruvate metabolism I: anaplerotic reactions, PEP	NADP-dependent malic enzyme (EC 1.1.1.40)
Carbohydrates	Central carbohydrate metabolism	Pyruvate metabolism I: anaplerotic reactions, PEP	Pyruvate kinase (EC 2.7.1.40)
Carbohydrates	Central carbohydrate metabolism	Pyruvate metabolism I: anaplerotic reactions, PEP	NAD-dependent malic enzyme (EC 1.1.1.38)
Carbohydrates	Central carbohydrate metabolism	Glyoxylate bypass	Citrate synthase (si) (EC 2.3.3.1)
Carbohydrates	Central carbohydrate metabolism	Glyoxylate bypass	Isocitrate lyase (EC 4.1.3.1)
Carbohydrates	Central carbohydrate metabolism	Glyoxylate bypass	Malate synthase (EC 2.3.3.9)
Carbohydrates	Central carbohydrate metabolism	Glyoxylate bypass	Aconitate hydratase (EC 4.2.1.3)
Carbohydrates	Central carbohydrate metabolism	Dehydrogenase complexes	Branched-chain alpha-keto acid dehydrogenase, E1 component, beta subunit (EC 1.2.4.4)
Carbohydrates	Central carbohydrate metabolism	Dehydrogenase complexes	2-oxoglutarate dehydrogenase E1 component (EC 1.2.4.2)
Carbohydrates	Central carbohydrate metabolism	Dehydrogenase complexes	Pyruvate dehydrogenase E1 component (EC 1.2.4.1)
Carbohydrates	Central carbohydrate metabolism	Dehydrogenase complexes	Cytosol aminopeptidase PepA (EC 3.4.11.1)

Carbohydrates	Central carbohydrate metabolism	Dehydrogenase complexes	Dihydrolipoamide succinyltransferase component (E2) of 2-oxoglutarate dehydrogenase complex (EC 2.3.1.61)
Carbohydrates	Central carbohydrate metabolism	Dehydrogenase complexes	Branched-chain alpha-keto acid dehydrogenase, E1 component, alpha subunit (EC 1.2.4.4)
Carbohydrates	Central carbohydrate metabolism	Dehydrogenase complexes	Dihydrolipoamide acyltransferase component of branched-chain alpha-keto acid dehydrogenase complex (EC 2.3.1.168)
Carbohydrates	Central carbohydrate metabolism	Dehydrogenase complexes	Dihydrolipoamide acetyltransferase component of pyruvate dehydrogenase complex (EC 2.3.1.12)
Carbohydrates	Central carbohydrate metabolism	Glycolate, glyoxylate interconversions	Glycolate dehydrogenase (EC 1.1.99.14), subunit GlcD
Carbohydrates	Central carbohydrate metabolism	Glycolate, glyoxylate interconversions	Phosphoglycolate phosphatase (EC 3.1.3.18)
Carbohydrates	Central carbohydrate metabolism	Glycolysis and Gluconeogenesis	Glucose-6-phosphate isomerase (EC 5.3.1.9)
Carbohydrates	Central carbohydrate metabolism	Glycolysis and Gluconeogenesis	Fructose-1,6-bisphosphatase, GlpX type (EC 3.1.3.11)
Carbohydrates	Central carbohydrate metabolism	Glycolysis and Gluconeogenesis	Fructose-bisphosphate aldolase class II (EC 4.1.2.13)
Carbohydrates	Central carbohydrate metabolism	Glycolysis and Gluconeogenesis	Phosphoenolpyruvate synthase (EC 2.7.9.2)
Carbohydrates	Central carbohydrate metabolism	Glycolysis and Gluconeogenesis	Putative phosphoenolpyruvate synthase/pyruvate phosphate dikinase, N-terminal domain
Carbohydrates	Central carbohydrate metabolism	Glycolysis and Gluconeogenesis	Glucokinase (EC 2.7.1.2)
Carbohydrates	Central carbohydrate metabolism	Glycolysis and Gluconeogenesis	Phosphoglycerate kinase (EC 2.7.2.3)
Carbohydrates	Central carbohydrate metabolism	Glycolysis and Gluconeogenesis	Enolase (EC 4.2.1.11)
Carbohydrates	Central carbohydrate metabolism	Glycolysis and Gluconeogenesis	Pyruvate kinase (EC 2.7.1.40)
Carbohydrates	Central carbohydrate metabolism	Glycolysis and Gluconeogenesis	Putative phosphoenolpyruvate synthase/pyruvate phosphate dikinase, C-terminal domain
Carbohydrates	Central carbohydrate metabolism	Glycolysis and Gluconeogenesis	NAD-dependent glyceraldehyde-3-phosphate dehydrogenase (EC 1.2.1.12)
Carbohydrates	Central carbohydrate metabolism	Glycolysis and Gluconeogenesis	6-phosphofructokinase (EC 2.7.1.11)
Carbohydrates	Central carbohydrate metabolism	Glycolysis and Gluconeogenesis	Triosephosphate isomerase (EC 5.3.1.1)
Carbohydrates	Central carbohydrate metabolism	Glycolysis and Gluconeogenesis	Phosphoglycerate mutase (EC 5.4.2.1)
Carbohydrates	Central carbohydrate metabolism	Glycolysis and Gluconeogenesis	Polyphosphate glucokinase (EC 2.7.1.63)
Carbohydrates	Central carbohydrate metabolism	Entner-Doudoroff Pathway	Pyruvate kinase (EC 2.7.1.40)
Carbohydrates	Central carbohydrate metabolism	Entner-Doudoroff Pathway	Phosphoglycerate kinase (EC 2.7.2.3)
Carbohydrates	Central carbohydrate metabolism	Entner-Doudoroff Pathway	Enolase (EC 4.2.1.11)
Carbohydrates	Central carbohydrate metabolism	Entner-Doudoroff Pathway	Glucokinase (EC 2.7.1.2)
Carbohydrates	Central carbohydrate metabolism	Entner-Doudoroff Pathway	Gluconokinase (EC 2.7.1.12)
Carbohydrates	Central carbohydrate metabolism	Entner-Doudoroff Pathway	6-phosphogluconolactonase (EC 3.1.1.31), eukaryotic type
Carbohydrates	Central carbohydrate metabolism	Entner-Doudoroff Pathway	OpcA, an allosteric effector of glucose-6-phosphate dehydrogenase, actinobacterial
Carbohydrates	Central carbohydrate metabolism	Entner-Doudoroff Pathway	Glucose-6-phosphate 1-dehydrogenase (EC 1.1.1.49)
Carbohydrates	Central carbohydrate metabolism	Entner-Doudoroff Pathway	Glucose 1-dehydrogenase (EC 1.1.1.47)
Carbohydrates	Central carbohydrate metabolism	Entner-Doudoroff Pathway	Polyphosphate glucokinase (EC 2.7.1.63)
Carbohydrates	Central carbohydrate metabolism	Entner-Doudoroff Pathway	Gluconate dehydratase (EC 4.2.1.39)
Carbohydrates	Central carbohydrate metabolism	Entner-Doudoroff Pathway	Phosphoglycerate mutase (EC 5.4.2.1)
Carbohydrates	Central carbohydrate metabolism	Entner-Doudoroff Pathway	2-dehydro-3-deoxyphosphogluconate aldolase (EC 4.1.2.14)
Carbohydrates	Central carbohydrate metabolism	Entner-Doudoroff Pathway	NAD-dependent glyceraldehyde-3-phosphate dehydrogenase (EC 1.2.1.12)
Carbohydrates	Central carbohydrate metabolism	Pyruvate metabolism II: acetyl-CoA, acetogenesis from pyruvate	Dihydrolipoamide acetyltransferase component of pyruvate dehydrogenase complex (EC 2.3.1.12)
Carbohydrates	Central carbohydrate metabolism	Pyruvate metabolism II: acetyl-CoA, acetogenesis from pyruvate	Aldehyde dehydrogenase (EC 1.2.1.3)

Carbohydrates	Central carbohydrate metabolism	Pyruvate metabolism II: acetyl-CoA, acetogenesis from pyruvate	Acetate kinase (EC 2.7.2.1)
Carbohydrates	Central carbohydrate metabolism	Pyruvate metabolism II: acetyl-CoA, acetogenesis from pyruvate	Pyruvate dehydrogenase E1 component (EC 1.2.4.1)
Carbohydrates	Central carbohydrate metabolism	Pyruvate metabolism II: acetyl-CoA, acetogenesis from pyruvate	Dihydrolipoamide dehydrogenase of pyruvate dehydrogenase complex (EC 1.8.1.4)
Carbohydrates	Central carbohydrate metabolism	Pyruvate metabolism II: acetyl-CoA, acetogenesis from pyruvate	NAD-dependent protein deacetylase of SIR2 family
Carbohydrates	Central carbohydrate metabolism	Pyruvate metabolism II: acetyl-CoA, acetogenesis from pyruvate	Acetyl-CoA synthetase (ADP-forming) alpha and beta chains, putative
Carbohydrates	Central carbohydrate metabolism	Pyruvate metabolism II: acetyl-CoA, acetogenesis from pyruvate	Phosphate acetyltransferase (EC 2.3.1.8)
Carbohydrates	Central carbohydrate metabolism	TCA Cycle	Succinyl-CoA ligase [ADP-forming] alpha chain (EC 6.2.1.5)
Carbohydrates	Central carbohydrate metabolism	TCA Cycle	Fumarate hydratase class II (EC 4.2.1.2)
Carbohydrates	Central carbohydrate metabolism	TCA Cycle	Aconitate hydratase (EC 4.2.1.3)
Carbohydrates	Central carbohydrate metabolism	TCA Cycle	Dihydrolipoamide succinyltransferase component (E2) of 2-oxoglutarate dehydrogenase complex (EC 2.3.1.61)
Carbohydrates	Central carbohydrate metabolism	TCA Cycle	Citrate synthase (si) (EC 2.3.3.1)
Carbohydrates	Central carbohydrate metabolism	TCA Cycle	Isocitrate dehydrogenase [NADP] (EC 1.1.1.42)
Carbohydrates	Central carbohydrate metabolism	TCA Cycle	Succinyl-CoA ligase [ADP-forming] beta chain (EC 6.2.1.5)
Carbohydrates	Central carbohydrate metabolism	TCA Cycle	Malate:quinone oxidoreductase (EC 1.1.5.4)
Carbohydrates	Central carbohydrate metabolism	TCA Cycle	Dihydrolipoamide dehydrogenase of pyruvate dehydrogenase complex (EC 1.8.1.4)
Carbohydrates	Central carbohydrate metabolism	TCA Cycle	2-oxoglutarate dehydrogenase E1 component (EC 1.2.4.2)
Carbohydrates	Monosaccharides	D-galactarate, D-glucarate and D-glycerate catabolism - gjo	2-hydroxy-3-oxopropionate reductase (EC 1.1.1.60)
Carbohydrates	Monosaccharides	D-galactarate, D-glucarate and D-glycerate catabolism - gjo	Predicted D-glucarate or D-galactorate regulator, GntR family
Carbohydrates	Monosaccharides	D-galactarate, D-glucarate and D-glycerate catabolism - gjo	Glycerate kinase (EC 2.7.1.31)
Carbohydrates	Monosaccharides	D-galactarate, D-glucarate and D-glycerate catabolism - gjo	5-dehydro-4-deoxyglucarate dehydratase (EC 4.2.1.41)
Carbohydrates	Monosaccharides	D-galactarate, D-glucarate and D-glycerate catabolism - gjo	Glucarate dehydratase (EC 4.2.1.40)
Carbohydrates	Monosaccharides	D-Galacturonate and D-Glucuronate Utilization	Uronate isomerase (EC 5.3.1.12)
Carbohydrates	Monosaccharides	D-Galacturonate and D-Glucuronate Utilization	D-mannonate oxidoreductase (EC 1.1.1.57)
Carbohydrates	Monosaccharides	D-Galacturonate and D-Glucuronate Utilization	Glucuronide transporter UidB
Carbohydrates	Monosaccharides	D-Galacturonate and D-Glucuronate Utilization	5-dehydro-4-deoxyglucarate dehydratase (EC 4.2.1.41)
Carbohydrates	Monosaccharides	D-Galacturonate and D-Glucuronate Utilization	Mannonate dehydratase (EC 4.2.1.8)
Carbohydrates	Monosaccharides	D-Galacturonate and D-Glucuronate Utilization	Alpha-glucosidase (EC 3.2.1.20)
Carbohydrates	Monosaccharides	D-Galacturonate and D-Glucuronate Utilization	2-dehydro-3-deoxyphosphogluconate aldolase (EC 4.1.2.14)
Carbohydrates	Monosaccharides	Mannose Metabolism	Phosphomannomutase (EC 5.4.2.8)
Carbohydrates	Monosaccharides	Mannose Metabolism	Mannose-6-phosphate isomerase (EC 5.3.1.8)
Carbohydrates	Monosaccharides	D-gluconate and ketogluconates metabolism	Gluconokinase (EC 2.7.1.12)

Carbohydrates	Monosaccharides	D-gluconate and ketogluconates metabolism	5-keto-D-gluconate 5-reductase (EC 1.1.1.69)
Carbohydrates	Monosaccharides	D-gluconate and ketogluconates metabolism	Gluconate dehydratase (EC 4.2.1.39)
Carbohydrates	Monosaccharides	D-gluconate and ketogluconates metabolism	L-idonate 5-dehydrogenase (EC 1.1.1.264)
Carbohydrates	Monosaccharides	D-gluconate and ketogluconates metabolism	6-phosphogluconate dehydrogenase, decarboxylating (EC 1.1.1.44)
Carbohydrates	Monosaccharides	D-gluconate and ketogluconates metabolism	Glucose 1-dehydrogenase (EC 1.1.1.47)
Carbohydrates	Monosaccharides	D-gluconate and ketogluconates metabolism	Low-affinity gluconate/H+ symporter GntU
Carbohydrates	Monosaccharides	D-gluconate and ketogluconates metabolism	Gluconate transporter family protein
Carbohydrates	Monosaccharides	Deoxyribose and Deoxynucleoside Catabolism	Deoxyribose-phosphate aldolase (EC 4.1.2.4)
Carbohydrates	Monosaccharides	Deoxyribose and Deoxynucleoside Catabolism	Purine nucleoside phosphorylase (EC 2.4.2.1)
Carbohydrates	Monosaccharides	Deoxyribose and Deoxynucleoside Catabolism	Ribokinase (EC 2.7.1.15)
Carbohydrates	Monosaccharides	Deoxyribose and Deoxynucleoside Catabolism	Pyrimidine-nucleoside phosphorylase (EC 2.4.2.2)
Carbohydrates	Monosaccharides	D-ribose utilization	Ribokinase (EC 2.7.1.15)
Carbohydrates	Monosaccharides	D-galactarate, D-glucarate and D-glycerate catabolism	Predicted D-glucarate or D-galactorate regulator, GntR family
Carbohydrates	Monosaccharides	D-galactarate, D-glucarate and D-glycerate catabolism	2-hydroxy-3-oxopropionate reductase (EC 1.1.1.60)
Carbohydrates	Monosaccharides	D-galactarate, D-glucarate and D-glycerate catabolism	Glucarate dehydratase (EC 4.2.1.40)
Carbohydrates	Monosaccharides	D-galactarate, D-glucarate and D-glycerate catabolism	Glycerate kinase (EC 2.7.1.31)
Carbohydrates	Monosaccharides	D-galactarate, D-glucarate and D-glycerate catabolism	5-dehydro-4-deoxyglucarate dehydratase (EC 4.2.1.41)
Carbohydrates	Monosaccharides	Fructose utilization	1-phosphofructokinase (EC 2.7.1.56)
Carbohydrates	Monosaccharides	Fructose utilization	Phosphotransferase system, phosphocarrier protein HPr
Carbohydrates	Monosaccharides	Fructose utilization	Transcriptional repressor of the fructose operon, DeoR family
Carbohydrates	Monosaccharides	Fructose utilization	Fructokinase (EC 2.7.1.4)
Carbohydrates	Monosaccharides	Fructose utilization	Phosphoenolpyruvate-protein phosphotransferase of PTS system (EC 2.7.3.9)
Carbohydrates	Monosaccharides	Fructose utilization	Transaldolase (EC 2.2.1.2)
Carbohydrates	Di- and oligosaccharides	Maltose and Maltodextrin Utilization	Neopullulanase (EC 3.2.1.135)
Carbohydrates	Di- and oligosaccharides	Maltose and Maltodextrin Utilization	Beta-phosphoglucomutase (EC 5.4.2.6)
Carbohydrates	Di- and oligosaccharides	Maltose and Maltodextrin Utilization	Maltose phosphorylase (EC 2.4.1.8)
Carbohydrates	Di- and oligosaccharides	Maltose and Maltodextrin Utilization	Alpha-amylase (EC 3.2.1.1)
Carbohydrates	Di- and oligosaccharides	Maltose and Maltodextrin Utilization	Malto-oligosyltrehalose synthase (EC 5.4.99.15)
Carbohydrates	Di- and oligosaccharides	Maltose and Maltodextrin Utilization	4-alpha-glucanotransferase (amylomaltase) (EC 2.4.1.25)
Carbohydrates	Di- and oligosaccharides	Maltose and Maltodextrin Utilization	Glycogen phosphorylase (EC 2.4.1.1)
Carbohydrates	Di- and oligosaccharides	Maltose and Maltodextrin Utilization	Alpha-glucosidase (EC 3.2.1.20)
Carbohydrates	Di- and oligosaccharides	Maltose and Maltodextrin Utilization	Glucoamylase (EC 3.2.1.3)
Carbohydrates	Di- and oligosaccharides	Trehalose Uptake and Utilization	Trehalose phosphorylase (EC 2.4.1.64)
Carbohydrates	Di- and oligosaccharides	Trehalose Uptake and Utilization	Glucose/mannose:H+ symporter GlcP
Carbohydrates	Di- and oligosaccharides	Trehalose Uptake and Utilization	Beta-phosphoglucomutase (EC 5.4.2.6)
Carbohydrates	Di- and oligosaccharides	Trehalose Biosynthesis	Alpha-amylase (EC 3.2.1.1)
Carbohydrates	Di- and oligosaccharides	Trehalose Biosynthesis	Malto-oligosyltrehalose synthase (EC 5.4.99.15)
Carbohydrates	Di- and oligosaccharides	Trehalose Biosynthesis	1,4-alpha-glucan (glycogen) branching enzyme, GH-13-type (EC 2.4.1.18)
Carbohydrates	Di- and oligosaccharides	Trehalose Biosynthesis	Trehalose synthase (EC 5.4.99.16)
Carbohydrates	Di- and oligosaccharides	Trehalose Biosynthesis	Putative glucanase glgE (EC 3.2.1.-)
Carbohydrates	Di- and oligosaccharides	Trehalose Biosynthesis	Glucoamylase (EC 3.2.1.3)
Carbohydrates	Di- and oligosaccharides	Trehalose Biosynthesis	Trehalose-6-phosphate phosphatase (EC 3.1.3.12)
Carbohydrates	Di- and oligosaccharides	Trehalose Biosynthesis	Trehalose phosphorylase (EC 2.4.1.64)

Carbohydrates	Di- and oligosaccharides	Trehalose Biosynthesis	Malto-oligosyltrehalose trehalohydrolase (EC 3.2.1.141)
Carbohydrates	Di- and oligosaccharides	Lactose and Galactose Uptake and Utilization	UDP-glucose 4-epimerase (EC 5.1.3.2)
Carbohydrates	Di- and oligosaccharides	Lactose and Galactose Uptake and Utilization	Galactokinase (EC 2.7.1.6)
Carbohydrates	Di- and oligosaccharides	Lactose and Galactose Uptake and Utilization	Galactose-1-phosphate uridylyltransferase (EC 2.7.7.10)
Carbohydrates	Aminosugars	Chitin and N-acetylglucosamine utilization	Predicted transcriptional regulator of N-Acetylglucosamine utilization, GntR family
Carbohydrates	Aminosugars	Chitin and N-acetylglucosamine utilization	N-acetylglucosamine-6-phosphate deacetylase (EC 3.5.1.25)
Carbohydrates	Aminosugars	Chitin and N-acetylglucosamine utilization	N-acetylglucosamine kinase of eukaryotic type (EC 2.7.1.59)
Carbohydrates	Aminosugars	Chitin and N-acetylglucosamine utilization	Chitinase (EC 3.2.1.14)
Carbohydrates	Aminosugars	Chitin and N-acetylglucosamine utilization	Glucosamine-6-phosphate deaminase (EC 3.5.99.6)
Carbohydrates	Polysaccharides	Glycogen metabolism	4-alpha-glucanotransferase (amylomaltase) (EC 2.4.1.25)
Carbohydrates	Polysaccharides	Glycogen metabolism	Glycogen phosphorylase (EC 2.4.1.1)
Carbohydrates	Polysaccharides	Glycogen metabolism	Glucose-1-phosphate adenylyltransferase (EC 2.7.7.27)
Carbohydrates	Polysaccharides	Glycogen metabolism	Predicted glycogen synthase, ADP-glucose transglucosylase (EC 2.4.1.21), Actinobacterial type
Carbohydrates	Polysaccharides	Glycogen metabolism	1,4-alpha-glucan (glycogen) branching enzyme, GH-13-type (EC 2.4.1.18)
Carbohydrates	Organic acids	Glycerate metabolism	2-hydroxy-3-oxopropionate reductase (EC 1.1.1.60)
Carbohydrates	Organic acids	Glycerate metabolism	Hydroxypyruvate isomerase (EC 5.3.1.22)
Carbohydrates	Organic acids	Glycerate metabolism	Pyruvate kinase (EC 2.7.1.40)
Carbohydrates	Organic acids	Glycerate metabolism	Glyoxylate carboligase (EC 4.1.1.47)
Carbohydrates	Organic acids	Glycerate metabolism	Glycerate kinase (EC 2.7.1.31)
Carbohydrates	Organic acids	Propionate-CoA to Succinate Module	2-methylcitrate dehydratase (EC 4.2.1.79)
Carbohydrates	Organic acids	Propionate-CoA to Succinate Module	2-methylaconitate isomerase
Carbohydrates	Organic acids	Propionate-CoA to Succinate Module	Methylisocitrate lyase (EC 4.1.3.30)
Carbohydrates	Organic acids	Propionate-CoA to Succinate Module	2-methylisocitrate dehydratase (EC 4.2.1.99)
Carbohydrates	Organic acids	Propionate-CoA to Succinate Module	Aconitate hydratase (EC 4.2.1.3)
Carbohydrates	Organic acids	Propionate-CoA to Succinate Module	2-methylcitrate synthase (EC 2.3.3.5)
Carbohydrates	Organic acids	Lactate utilization	Predicted L-lactate dehydrogenase, Iron-sulfur cluster-binding subunit YkgF
Carbohydrates	Organic acids	Lactate utilization	L-lactate permease
Carbohydrates	Organic acids	Lactate utilization	Predicted L-lactate dehydrogenase, hypothetical protein subunit YkgG
Carbohydrates	Organic acids	Lactate utilization	Lactate-responsive regulator LldR in Actinobacteria, GntR family
Carbohydrates	Organic acids	Lactate utilization	Predicted D-lactate dehydrogenase, Fe-S protein, FAD/FMN-containing
Carbohydrates	Organic acids	Lactate utilization	Predicted L-lactate dehydrogenase, Fe-S oxidoreductase subunit YkgE
Carbohydrates	Organic acids	Methylcitrate cycle	2-methylcitrate synthase (EC 2.3.3.5)
Carbohydrates	Organic acids	Methylcitrate cycle	2-methylisocitrate dehydratase (EC 4.2.1.99)
Carbohydrates	Organic acids	Methylcitrate cycle	Methylisocitrate lyase (EC 4.1.3.30)
Carbohydrates	Organic acids	Methylcitrate cycle	2-methylaconitate isomerase
Carbohydrates	Organic acids	Methylcitrate cycle	2-methylcitrate dehydratase (EC 4.2.1.79)
Amino Acids and Derivatives	Alanine, serine, and glycine	Alanine biosynthesis	Branched-chain amino acid aminotransferase (EC 2.6.1.42)
Amino Acids and Derivatives	Alanine, serine, and glycine	Alanine biosynthesis	Cysteine desulfurase (EC 2.8.1.7)
Amino Acids and Derivatives	Alanine, serine, and glycine	Alanine biosynthesis	Alanine racemase (EC 5.1.1.1)

Amino Acids and Derivatives	Alanine, serine, and glycine	Alanine biosynthesis	Ferredoxin, 2Fe-2S
Amino Acids and Derivatives	Alanine, serine, and glycine	Serine Biosynthesis	Phosphoserine aminotransferase (EC 2.6.1.52)
Amino Acids and Derivatives	Alanine, serine, and glycine	Serine Biosynthesis	Serine hydroxymethyltransferase (EC 2.1.2.1)
Amino Acids and Derivatives	Alanine, serine, and glycine	Serine Biosynthesis	Phosphoserine phosphatase (EC 3.1.3.3)
Amino Acids and Derivatives	Alanine, serine, and glycine	Serine Biosynthesis	D-3-phosphoglycerate dehydrogenase (EC 1.1.1.95)
Amino Acids and Derivatives	Alanine, serine, and glycine	Glycine cleavage system	Glycine dehydrogenase [decarboxylating] (glycine cleavage system P protein) (EC 1.4.4.2)
Amino Acids and Derivatives	Alanine, serine, and glycine	Glycine cleavage system	Glycine cleavage system H protein
Amino Acids and Derivatives	Alanine, serine, and glycine	Glycine cleavage system	Aminomethyltransferase (glycine cleavage system T protein) (EC 2.1.2.10)
Amino Acids and Derivatives	Alanine, serine, and glycine	Glycine and Serine Utilization	Serine hydroxymethyltransferase (EC 2.1.2.1)
Amino Acids and Derivatives	Alanine, serine, and glycine	Glycine and Serine Utilization	L-serine dehydratase, alpha subunit (EC 4.3.1.17)
Amino Acids and Derivatives	Alanine, serine, and glycine	Glycine and Serine Utilization	Phosphoserine phosphatase (EC 3.1.3.3)
Amino Acids and Derivatives	Alanine, serine, and glycine	Glycine and Serine Utilization	Aminomethyltransferase (glycine cleavage system T protein) (EC 2.1.2.10)
Amino Acids and Derivatives	Alanine, serine, and glycine	Glycine and Serine Utilization	Phosphoserine aminotransferase (EC 2.6.1.52)
Amino Acids and Derivatives	Alanine, serine, and glycine	Glycine and Serine Utilization	Seryl-tRNA synthetase (EC 6.1.1.11)
Amino Acids and Derivatives	Alanine, serine, and glycine	Glycine and Serine Utilization	Glycine cleavage system H protein
Amino Acids and Derivatives	Alanine, serine, and glycine	Glycine and Serine Utilization	Cystathionine beta-synthase (EC 4.2.1.22)
Amino Acids and Derivatives	Alanine, serine, and glycine	Glycine and Serine Utilization	D-serine/D-alanine/glycine transporter
Amino Acids and Derivatives	Alanine, serine, and glycine	Glycine and Serine Utilization	Cystathionine gamma-lyase (EC 4.4.1.1)
Amino Acids and Derivatives	Alanine, serine, and glycine	Glycine and Serine Utilization	L-serine dehydratase, beta subunit (EC 4.3.1.17)
Amino Acids and Derivatives	Alanine, serine, and glycine	Glycine and Serine Utilization	2-amino-3-ketobutyrate coenzyme A ligase (EC 2.3.1.29)
Amino Acids and Derivatives	Alanine, serine, and glycine	Glycine and Serine Utilization	Glycerate kinase (EC 2.7.1.31)

Amino Acids and Derivatives	Alanine, serine, and glycine	Glycine and Serine Utilization	L-serine dehydratase, (PLP)-dependent (EC 4.3.1.17)
Amino Acids and Derivatives	Alanine, serine, and glycine	Glycine and Serine Utilization	D-3-phosphoglycerate dehydrogenase (EC 1.1.1.95)
Amino Acids and Derivatives	Alanine, serine, and glycine	Glycine and Serine Utilization	Threonine dehydratase, catabolic (EC 4.3.1.19)
Amino Acids and Derivatives	Alanine, serine, and glycine	Glycine and Serine Utilization	Glycine dehydrogenase [decarboxylating] (glycine cleavage system P protein) (EC 1.4.4.2)
Amino Acids and Derivatives	Alanine, serine, and glycine	Glycine Biosynthesis	L-threonine 3-dehydrogenase (EC 1.1.1.103)
Amino Acids and Derivatives	Alanine, serine, and glycine	Glycine Biosynthesis	2-amino-3-ketobutyrate coenzyme A ligase (EC 2.3.1.29)
Amino Acids and Derivatives	Alanine, serine, and glycine	Glycine Biosynthesis	Serine hydroxymethyltransferase (EC 2.1.2.1)
Amino Acids and Derivatives	Arginine; urea cycle, polyamines	Urease subunits	Urease accessory protein UreE
Amino Acids and Derivatives	Arginine; urea cycle, polyamines	Urease subunits	Urease accessory protein UreF
Amino Acids and Derivatives	Arginine; urea cycle, polyamines	Urease subunits	Urease beta subunit (EC 3.5.1.5)
Amino Acids and Derivatives	Arginine; urea cycle, polyamines	Urease subunits	Urease alpha subunit (EC 3.5.1.5)
Amino Acids and Derivatives	Arginine; urea cycle, polyamines	Urease subunits	Urease gamma subunit (EC 3.5.1.5)
Amino Acids and Derivatives	Arginine; urea cycle, polyamines	Urease subunits	Urease accessory protein UreG
Amino Acids and Derivatives	Arginine; urea cycle, polyamines	Urease subunits	Urease accessory protein UreD
Amino Acids and Derivatives	Arginine; urea cycle, polyamines	Arginine Deiminase Pathway	Arginine pathway regulatory protein ArgR, repressor of arg regulon
Amino Acids and Derivatives	Arginine; urea cycle, polyamines	Arginine Deiminase Pathway	Arginine/ornithine antiporter ArcD
Amino Acids and Derivatives	Arginine; urea cycle, polyamines	Arginine Deiminase Pathway	Ornithine carbamoyltransferase (EC 2.1.3.3)
Amino Acids and Derivatives	Arginine; urea cycle, polyamines	Urea decomposition	Urease gamma subunit (EC 3.5.1.5)
Amino Acids and Derivatives	Arginine; urea cycle, polyamines	Urea decomposition	Urease accessory protein UreG
Amino Acids and Derivatives	Arginine; urea cycle, polyamines	Urea decomposition	Urea carboxylase-related amino acid permease
Amino Acids and Derivatives	Arginine; urea cycle, polyamines	Urea decomposition	Urease accessory protein UreE

Amino Acids and Derivatives	Arginine; urea cycle, polyamines	Urea decomposition	Urease accessory protein UreF
Amino Acids and Derivatives	Arginine; urea cycle, polyamines	Urea decomposition	Urease beta subunit (EC 3.5.1.5)
Amino Acids and Derivatives	Arginine; urea cycle, polyamines	Urea decomposition	Urease alpha subunit (EC 3.5.1.5)
Amino Acids and Derivatives	Arginine; urea cycle, polyamines	Urea decomposition	Urease accessory protein UreD
Amino Acids and Derivatives	Arginine; urea cycle, polyamines	Arginine and Ornithine Degradation	Delta-1-pyrroline-5-carboxylate dehydrogenase (EC 1.2.1.88)
Amino Acids and Derivatives	Arginine; urea cycle, polyamines	Arginine and Ornithine Degradation	NADP-specific glutamate dehydrogenase (EC 1.4.1.4)
Amino Acids and Derivatives	Arginine; urea cycle, polyamines	Arginine and Ornithine Degradation	Ornithine cyclodeaminase (EC 4.3.1.12)
Amino Acids and Derivatives	Arginine; urea cycle, polyamines	Arginine and Ornithine Degradation	Arginine pathway regulatory protein ArgR, repressor of arg regulon
Amino Acids and Derivatives	Arginine; urea cycle, polyamines	Arginine and Ornithine Degradation	Arginine/ornithine antiporter ArcD
Amino Acids and Derivatives	Arginine; urea cycle, polyamines	Arginine and Ornithine Degradation	Ornithine carbamoyltransferase (EC 2.1.3.3)
Amino Acids and Derivatives	Arginine; urea cycle, polyamines	Polyamine Metabolism	Spermidine synthase (EC 2.5.1.16)
Amino Acids and Derivatives	Arginine; urea cycle, polyamines	Polyamine Metabolism	Arginine/ornithine antiporter ArcD
Amino Acids and Derivatives	Arginine; urea cycle, polyamines	Polyamine Metabolism	4-aminobutyraldehyde dehydrogenase (EC 1.2.1.19)
Amino Acids and Derivatives	Arginine; urea cycle, polyamines	Putrescine utilization pathways	4-aminobutyraldehyde dehydrogenase (EC 1.2.1.19)
Amino Acids and Derivatives	Arginine; urea cycle, polyamines	Putrescine utilization pathways	Gamma-aminobutyrate:alpha-ketoglutarate aminotransferase (EC 2.6.1.19)
Amino Acids and Derivatives	Branched-chain amino acids	Valine degradation	3-hydroxyisobutyrate dehydrogenase (EC 1.1.1.31)
Amino Acids and Derivatives	Branched-chain amino acids	Valine degradation	Branched-chain alpha-keto acid dehydrogenase, E1 component, beta subunit (EC 1.2.4.4)
Amino Acids and Derivatives	Branched-chain amino acids	Valine degradation	Enoyl-CoA hydratase (EC 4.2.1.17)
Amino Acids and Derivatives	Branched-chain amino acids	Valine degradation	Branched-chain alpha-keto acid dehydrogenase, E1 component, alpha subunit (EC 1.2.4.4)
Amino Acids and Derivatives	Branched-chain amino acids	Valine degradation	Methylmalonate-semialdehyde dehydrogenase (EC 1.2.1.27)
Amino Acids and Derivatives	Branched-chain amino acids	Valine degradation	Branched-chain amino acid aminotransferase (EC 2.6.1.42)

Amino Acids and Derivatives	Branched-chain amino acids	Valine degradation	Dihydrolipoamide acyltransferase component of branched-chain alpha-keto acid dehydrogenase complex (EC 2.3.1.168)
Amino Acids and Derivatives	Branched-chain amino acids	Valine degradation	3-hydroxyacyl-CoA dehydrogenase (EC 1.1.1.35)
Amino Acids and Derivatives	Branched-chain amino acids	Valine degradation	3-hydroxyisobutyryl-CoA hydrolase (EC 3.1.2.4)
Amino Acids and Derivatives	Branched-chain amino acids	Branched-Chain Amino Acid Biosynthesis	Acetolactate synthase small subunit (EC 2.2.1.6)
Amino Acids and Derivatives	Branched-chain amino acids	Branched-Chain Amino Acid Biosynthesis	3-isopropylmalate dehydrogenase (EC 1.1.1.85)
Amino Acids and Derivatives	Branched-chain amino acids	Branched-Chain Amino Acid Biosynthesis	3-isopropylmalate dehydratase large subunit (EC 4.2.1.33)
Amino Acids and Derivatives	Branched-chain amino acids	Branched-Chain Amino Acid Biosynthesis	3-isopropylmalate dehydratase small subunit (EC 4.2.1.33)
Amino Acids and Derivatives	Branched-chain amino acids	Branched-Chain Amino Acid Biosynthesis	Dihydroxy-acid dehydratase (EC 4.2.1.9)
Amino Acids and Derivatives	Branched-chain amino acids	Branched-Chain Amino Acid Biosynthesis	Branched-chain amino acid aminotransferase (EC 2.6.1.42)
Amino Acids and Derivatives	Branched-chain amino acids	Branched-Chain Amino Acid Biosynthesis	Leucine-responsive regulatory protein, regulator for leucine (or lrp) regulon and high-affinity branched-chain amino acid transport system
Amino Acids and Derivatives	Branched-chain amino acids	Branched-Chain Amino Acid Biosynthesis	Acetolactate synthase large subunit (EC 2.2.1.6)
Amino Acids and Derivatives	Branched-chain amino acids	Branched-Chain Amino Acid Biosynthesis	Threonine dehydratase, catabolic (EC 4.3.1.19)
Amino Acids and Derivatives	Branched-chain amino acids	Branched-Chain Amino Acid Biosynthesis	2-isopropylmalate synthase (EC 2.3.3.13)
Amino Acids and Derivatives	Branched-chain amino acids	Leucine Biosynthesis	3-isopropylmalate dehydratase large subunit (EC 4.2.1.33)
Amino Acids and Derivatives	Branched-chain amino acids	Leucine Biosynthesis	2-isopropylmalate synthase (EC 2.3.3.13)
Amino Acids and Derivatives	Branched-chain amino acids	Leucine Biosynthesis	Branched-chain amino acid aminotransferase (EC 2.6.1.42)
Amino Acids and Derivatives	Branched-chain amino acids	Leucine Biosynthesis	3-isopropylmalate dehydratase small subunit (EC 4.2.1.33)
Amino Acids and Derivatives	Branched-chain amino acids	Leucine Biosynthesis	3-isopropylmalate dehydrogenase (EC 1.1.1.85)
Amino Acids and Derivatives	Branched-chain amino acids	Isoleucine degradation	Enoyl-CoA hydratase (EC 4.2.1.17)
Amino Acids and Derivatives	Branched-chain amino acids	Isoleucine degradation	Branched-chain alpha-keto acid dehydrogenase, E1 component, beta subunit (EC 1.2.4.4)
Amino Acids and Derivatives	Branched-chain amino acids	Isoleucine degradation	Branched-chain alpha-keto acid dehydrogenase, E1 component, alpha subunit (EC 1.2.4.4)

Amino Acids and Derivatives	Branched-chain amino acids	Isoleucine degradation	Branched-chain amino acid aminotransferase (EC 2.6.1.42)
Amino Acids and Derivatives	Branched-chain amino acids	Isoleucine degradation	3-ketoacyl-CoA thiolase (EC 2.3.1.16)
Amino Acids and Derivatives	Branched-chain amino acids	Isoleucine degradation	Dihydrolipoamide acyltransferase component of branched-chain alpha-keto acid dehydrogenase complex (EC 2.3.1.168)
Amino Acids and Derivatives	Branched-chain amino acids	Isoleucine degradation	3-hydroxyacyl-CoA dehydrogenase (EC 1.1.1.35)
Amino Acids and Derivatives	Amino Acids and Derivatives - no subcategory	Creatine and Creatinine Degradation	Creatinase (EC 3.5.3.3)
Amino Acids and Derivatives	Amino Acids and Derivatives - no subcategory	Creatine and Creatinine Degradation	Creatinine amidohydrolase (EC 3.5.2.10)
Amino Acids and Derivatives	Amino Acids and Derivatives - no subcategory	Creatine and Creatinine Degradation	Cytosine deaminase (EC 3.5.4.1)
Amino Acids and Derivatives	Lysine, threonine, methionine, and cysteine	Methionine Biosynthesis	5,10-methylenetetrahydrofolate reductase (EC 1.5.1.20)
Amino Acids and Derivatives	Lysine, threonine, methionine, and cysteine	Methionine Biosynthesis	Cystathionine beta-synthase (EC 4.2.1.22)
Amino Acids and Derivatives	Lysine, threonine, methionine, and cysteine	Methionine Biosynthesis	Homoserine dehydrogenase (EC 1.1.1.3)
Amino Acids and Derivatives	Lysine, threonine, methionine, and cysteine	Methionine Biosynthesis	Adenosylhomocysteinase (EC 3.3.1.1)
Amino Acids and Derivatives	Lysine, threonine, methionine, and cysteine	Methionine Biosynthesis	Cystathionine gamma-lyase (EC 4.4.1.1)
Amino Acids and Derivatives	Lysine, threonine, methionine, and cysteine	Methionine Biosynthesis	Cystathionine gamma-synthase (EC 2.5.1.48)
Amino Acids and Derivatives	Lysine, threonine, methionine, and cysteine	Methionine Biosynthesis	O-acetylhomoserine sulfhydrylase (EC 2.5.1.49)
Amino Acids and Derivatives	Lysine, threonine, methionine, and cysteine	Methionine Biosynthesis	S-adenosylmethionine synthetase (EC 2.5.1.6)
Amino Acids and Derivatives	Lysine, threonine, methionine, and cysteine	Methionine Biosynthesis	Serine acetyltransferase (EC 2.3.1.30)
Amino Acids and Derivatives	Lysine, threonine, methionine, and cysteine	Methionine Biosynthesis	Methionine ABC transporter substrate-binding protein
Amino Acids and Derivatives	Lysine, threonine, methionine, and cysteine	Methionine Biosynthesis	Methionine ABC transporter ATP-binding protein
Amino Acids and Derivatives	Lysine, threonine, methionine, and cysteine	Methionine Biosynthesis	Homoserine O-acetyltransferase (EC 2.3.1.31)
Amino Acids and Derivatives	Lysine, threonine, methionine, and cysteine	Methionine Biosynthesis	5-methyltetrahydrofolate--homocysteine methyltransferase (EC 2.1.1.13)
Amino Acids and Derivatives	Lysine, threonine, methionine, and cysteine	Methionine Biosynthesis	Homoserine kinase (EC 2.7.1.39)

Amino Acids and Derivatives	Lysine, threonine, methionine, and cysteine	Methionine Biosynthesis	O-succinylhomoserine sulfhydrylase (EC 2.5.1.48)
Amino Acids and Derivatives	Lysine, threonine, methionine, and cysteine	Methionine Biosynthesis	Methionine ABC transporter permease protein
Amino Acids and Derivatives	Lysine, threonine, methionine, and cysteine	Methionine Biosynthesis	5-methyltetrahydropteroyltriglutamate--homocysteine methyltransferase (EC 2.1.1.14)
Amino Acids and Derivatives	Lysine, threonine, methionine, and cysteine	Methionine Biosynthesis	Cysteine synthase (EC 2.5.1.47)
Amino Acids and Derivatives	Lysine, threonine, methionine, and cysteine	Lysine Biosynthesis DAP Pathway, GJO scratch	Diaminopimelate epimerase (EC 5.1.1.7)
Amino Acids and Derivatives	Lysine, threonine, methionine, and cysteine	Lysine Biosynthesis DAP Pathway, GJO scratch	Diaminopimelate decarboxylase (EC 4.1.1.20)
Amino Acids and Derivatives	Lysine, threonine, methionine, and cysteine	Lysine Biosynthesis DAP Pathway, GJO scratch	4-hydroxy-tetrahydrodipicolinate reductase (EC 1.17.1.8)
Amino Acids and Derivatives	Lysine, threonine, methionine, and cysteine	Lysine Biosynthesis DAP Pathway, GJO scratch	Aspartokinase (EC 2.7.2.4)
Amino Acids and Derivatives	Lysine, threonine, methionine, and cysteine	Lysine Biosynthesis DAP Pathway, GJO scratch	2,3,4,5-tetrahydropyridine-2,6-dicarboxylate N-succinyltransferase (EC 2.3.1.117)
Amino Acids and Derivatives	Lysine, threonine, methionine, and cysteine	Lysine Biosynthesis DAP Pathway, GJO scratch	Aspartate-semialdehyde dehydrogenase (EC 1.2.1.11)
Amino Acids and Derivatives	Lysine, threonine, methionine, and cysteine	Lysine Biosynthesis DAP Pathway, GJO scratch	4-hydroxy-tetrahydrodipicolinate synthase (EC 4.3.3.7)
Amino Acids and Derivatives	Lysine, threonine, methionine, and cysteine	Lysine Biosynthesis DAP Pathway, GJO scratch	N-succinyl-L,L-diaminopimelate desuccinylase (EC 3.5.1.18)
Amino Acids and Derivatives	Lysine, threonine, methionine, and cysteine	Threonine degradation	Threonine dehydrogenase and related Zn-dependent dehydrogenases
Amino Acids and Derivatives	Lysine, threonine, methionine, and cysteine	Threonine degradation	L-threonine 3-dehydrogenase (EC 1.1.1.103)
Amino Acids and Derivatives	Lysine, threonine, methionine, and cysteine	Threonine degradation	Threonine dehydratase, catabolic (EC 4.3.1.19)
Amino Acids and Derivatives	Lysine, threonine, methionine, and cysteine	Cysteine Biosynthesis	Serine acetyltransferase (EC 2.3.1.30)
Amino Acids and Derivatives	Lysine, threonine, methionine, and cysteine	Cysteine Biosynthesis	Cysteine synthase (EC 2.5.1.47)
Amino Acids and Derivatives	Lysine, threonine, methionine, and cysteine	Cysteine Biosynthesis	Sulfate adenylyltransferase subunit 1 (EC 2.7.7.4)
Amino Acids and Derivatives	Lysine, threonine, methionine, and cysteine	Cysteine Biosynthesis	Phosphoadenylyl-sulfate reductase [thioredoxin] (EC 1.8.4.8)
Amino Acids and Derivatives	Lysine, threonine, methionine, and cysteine	Cysteine Biosynthesis	Sulfate adenylyltransferase subunit 2 (EC 2.7.7.4)
Amino Acids and Derivatives	Lysine, threonine, methionine, and cysteine	Cysteine Biosynthesis	Cystathionine beta-synthase (EC 4.2.1.22)

Amino Acids and Derivatives	Lysine, threonine, methionine, and cysteine	Cysteine Biosynthesis	Cystathionine gamma-lyase (EC 4.4.1.1)
Amino Acids and Derivatives	Lysine, threonine, methionine, and cysteine	Cysteine Biosynthesis	Sulfate permease
Amino Acids and Derivatives	Lysine, threonine, methionine, and cysteine	Threonine and Homoserine Biosynthesis	Homoserine dehydrogenase (EC 1.1.1.3)
Amino Acids and Derivatives	Lysine, threonine, methionine, and cysteine	Threonine and Homoserine Biosynthesis	Aspartate aminotransferase (EC 2.6.1.1)
Amino Acids and Derivatives	Lysine, threonine, methionine, and cysteine	Threonine and Homoserine Biosynthesis	Threonine synthase (EC 4.2.3.1)
Amino Acids and Derivatives	Lysine, threonine, methionine, and cysteine	Threonine and Homoserine Biosynthesis	Aspartokinase (EC 2.7.2.4)
Amino Acids and Derivatives	Lysine, threonine, methionine, and cysteine	Threonine and Homoserine Biosynthesis	Homoserine kinase (EC 2.7.1.39)
Amino Acids and Derivatives	Lysine, threonine, methionine, and cysteine	Threonine and Homoserine Biosynthesis	Aspartate-semialdehyde dehydrogenase (EC 1.2.1.11)
Amino Acids and Derivatives	Lysine, threonine, methionine, and cysteine	Lysine Biosynthesis DAP Pathway	Aspartokinase (EC 2.7.2.4)
Amino Acids and Derivatives	Lysine, threonine, methionine, and cysteine	Lysine Biosynthesis DAP Pathway	4-hydroxy-tetrahydrodipicolinate reductase (EC 1.17.1.8)
Amino Acids and Derivatives	Lysine, threonine, methionine, and cysteine	Lysine Biosynthesis DAP Pathway	Diaminopimelate decarboxylase (EC 4.1.1.20)
Amino Acids and Derivatives	Lysine, threonine, methionine, and cysteine	Lysine Biosynthesis DAP Pathway	Diaminopimelate epimerase (EC 5.1.1.7)
Amino Acids and Derivatives	Lysine, threonine, methionine, and cysteine	Lysine Biosynthesis DAP Pathway	N-succinyl-L,L-diaminopimelate desuccinylase (EC 3.5.1.18)
Amino Acids and Derivatives	Lysine, threonine, methionine, and cysteine	Lysine Biosynthesis DAP Pathway	4-hydroxy-tetrahydrodipicolinate synthase (EC 4.3.3.7)
Amino Acids and Derivatives	Lysine, threonine, methionine, and cysteine	Lysine Biosynthesis DAP Pathway	Aspartate-semialdehyde dehydrogenase (EC 1.2.1.11)
Amino Acids and Derivatives	Lysine, threonine, methionine, and cysteine	Lysine Biosynthesis DAP Pathway	2,3,4,5-tetrahydropyridine-2,6-dicarboxylate N-succinyltransferase (EC 2.3.1.117)
Amino Acids and Derivatives	Glutamine, glutamate, aspartate, asparagine; ammonia assimilation	Glutamine, Glutamate, Aspartate and Asparagine Biosynthesis	Aspartate aminotransferase (EC 2.6.1.1)
Amino Acids and Derivatives	Glutamine, glutamate, aspartate, asparagine; ammonia assimilation	Glutamine, Glutamate, Aspartate and Asparagine Biosynthesis	NAD-specific glutamate dehydrogenase (EC 1.4.1.2), large form
Amino Acids and Derivatives	Glutamine, glutamate, aspartate, asparagine; ammonia assimilation	Glutamine, Glutamate, Aspartate and Asparagine Biosynthesis	Glutamate synthase [NADPH] small chain (EC 1.4.1.13)
Amino Acids and Derivatives	Glutamine, glutamate, aspartate, asparagine; ammonia assimilation	Glutamine, Glutamate, Aspartate and Asparagine Biosynthesis	Glutamate racemase (EC 5.1.1.3)
Amino Acids and Derivatives	Glutamine, glutamate, aspartate, asparagine; ammonia assimilation	Glutamine, Glutamate, Aspartate and Asparagine Biosynthesis	NADP-specific glutamate dehydrogenase (EC 1.4.1.4)

Amino Acids and Derivatives	Glutamine, glutamate, aspartate, asparagine; ammonia assimilation	Glutamine, Glutamate, Aspartate and Asparagine Biosynthesis	Glutamine synthetase type I (EC 6.3.1.2)
Amino Acids and Derivatives	Glutamine, glutamate, aspartate, asparagine; ammonia assimilation	Glutamine, Glutamate, Aspartate and Asparagine Biosynthesis	Leucine-responsive regulatory protein, regulator for leucine (or lrp) regulon and high-affinity branched-chain amino acid transport system
Amino Acids and Derivatives	Glutamine, glutamate, aspartate, asparagine; ammonia assimilation	Glutamine, Glutamate, Aspartate and Asparagine Biosynthesis	Aspartate ammonia-lyase (EC 4.3.1.1)
Amino Acids and Derivatives	Glutamine, glutamate, aspartate, asparagine; ammonia assimilation	Glutamine, Glutamate, Aspartate and Asparagine Biosynthesis	Glutamate synthase [NADPH] large chain (EC 1.4.1.13)
Amino Acids and Derivatives	Glutamine, glutamate, aspartate, asparagine; ammonia assimilation	Glutamate dehydrogenases	NAD-specific glutamate dehydrogenase (EC 1.4.1.2), large form
Amino Acids and Derivatives	Glutamine, glutamate, aspartate, asparagine; ammonia assimilation	Glutamate dehydrogenases	NADP-specific glutamate dehydrogenase (EC 1.4.1.4)
Amino Acids and Derivatives	Glutamine, glutamate, aspartate, asparagine; ammonia assimilation	Glutamine synthetases	Glutamine synthetase type I (EC 6.3.1.2)
Amino Acids and Derivatives	Histidine Metabolism	Histidine Degradation	Urocanate hydratase (EC 4.2.1.49)
Amino Acids and Derivatives	Histidine Metabolism	Histidine Degradation	Imidazolonepropionase (EC 3.5.2.7)
Amino Acids and Derivatives	Histidine Metabolism	Histidine Degradation	Histidine transport protein (permease)
Amino Acids and Derivatives	Histidine Metabolism	Histidine Degradation	Histidine ammonia-lyase (EC 4.3.1.3)
Amino Acids and Derivatives	Histidine Metabolism	Histidine Degradation	Formiminoglutamase (EC 3.5.3.8)
Amino Acids and Derivatives	Histidine Metabolism	Histidine Biosynthesis	Imidazoleglycerol-phosphate dehydratase (EC 4.2.1.19)
Amino Acids and Derivatives	Histidine Metabolism	Histidine Biosynthesis	Histidinol-phosphatase (EC 3.1.3.15)
Amino Acids and Derivatives	Histidine Metabolism	Histidine Biosynthesis	Phosphoribosylformimino-5-aminoimidazole carboxamide ribotide isomerase (EC 5.3.1.16)
Amino Acids and Derivatives	Histidine Metabolism	Histidine Biosynthesis	Histidinol dehydrogenase (EC 1.1.1.23)
Amino Acids and Derivatives	Histidine Metabolism	Histidine Biosynthesis	Phosphoribosyl-AMP cyclohydrolase (EC 3.5.4.19)
Amino Acids and Derivatives	Histidine Metabolism	Histidine Biosynthesis	Phosphoribosyl-ATP pyrophosphatase (EC 3.6.1.31)
Amino Acids and Derivatives	Histidine Metabolism	Histidine Biosynthesis	Histidinol-phosphatase [alternative form] (EC 3.1.3.15)
Amino Acids and Derivatives	Histidine Metabolism	Histidine Biosynthesis	Histidinol-phosphate aminotransferase (EC 2.6.1.9)
Amino Acids and Derivatives	Aromatic amino acids and derivatives	Phenylalanine and Tyrosine Branches from Chorismate	Biosynthetic Aromatic amino acid aminotransferase beta (EC 2.6.1.57)

Amino Acids and Derivatives	Aromatic amino acids and derivatives	Phenylalanine and Tyrosine Branches from Chorismate	Chorismate mutase I (EC 5.4.99.5)
Amino Acids and Derivatives	Aromatic amino acids and derivatives	Phenylalanine and Tyrosine Branches from Chorismate	Prephenate dehydratase (EC 4.2.1.51)
Amino Acids and Derivatives	Aromatic amino acids and derivatives	Phenylalanine and Tyrosine Branches from Chorismate	Arogenate dehydrogenase (EC 1.3.1.43)
Amino Acids and Derivatives	Aromatic amino acids and derivatives	Aromatic amino acid degradation	2-hydroxyhepta-2,4-diene-1,7-dioate isomerase (EC 5.3.3.-)
Amino Acids and Derivatives	Aromatic amino acids and derivatives	Aromatic amino acid degradation	5-carboxymethyl-2-hydroxymuconate semialdehyde dehydrogenase (EC 1.2.1.60)
Amino Acids and Derivatives	Aromatic amino acids and derivatives	Aromatic amino acid degradation	5-carboxymethyl-2-oxo-hex-3- ene-1,7-dioate decarboxylase (EC 4.1.1.68)
Amino Acids and Derivatives	Aromatic amino acids and derivatives	Aromatic amino acid degradation	Tryptophan 2,3-dioxygenase (EC 1.13.11.11)
Amino Acids and Derivatives	Aromatic amino acids and derivatives	Aromatic amino acid degradation	Aromatic-L-amino-acid decarboxylase (EC 4.1.1.28)
Amino Acids and Derivatives	Aromatic amino acids and derivatives	Aromatic amino acid degradation	5-carboxymethyl-2-hydroxymuconate delta-isomerase (EC 5.3.3.10)
Amino Acids and Derivatives	Aromatic amino acids and derivatives	Aromatic amino acid degradation	2-oxo-hepta-3-ene-1,7-dioic acid hydratase (EC 4.2.-.-)
Amino Acids and Derivatives	Aromatic amino acids and derivatives	Aromatic amino acid degradation	4-hydroxyphenylpyruvate dioxygenase (EC 1.13.11.27)
Amino Acids and Derivatives	Aromatic amino acids and derivatives	Common Pathway For Synthesis of Aromatic Compounds (DAHP synthase to chorismate)	Shikimate 5-dehydrogenase I alpha (EC 1.1.1.25)
Amino Acids and Derivatives	Aromatic amino acids and derivatives	Common Pathway For Synthesis of Aromatic Compounds (DAHP synthase to chorismate)	3-dehydroquinate dehydratase II (EC 4.2.1.10)
Amino Acids and Derivatives	Aromatic amino acids and derivatives	Common Pathway For Synthesis of Aromatic Compounds (DAHP synthase to chorismate)	2-keto-3-deoxy-D-arabino-heptulosonate-7-phosphate synthase I alpha (EC 2.5.1.54)
Amino Acids and Derivatives	Aromatic amino acids and derivatives	Common Pathway For Synthesis of Aromatic Compounds (DAHP synthase to chorismate)	Shikimate kinase I (EC 2.7.1.71)
Amino Acids and Derivatives	Aromatic amino acids and derivatives	Common Pathway For Synthesis of Aromatic Compounds (DAHP synthase to chorismate)	2-keto-3-deoxy-D-arabino-heptulosonate-7-phosphate synthase II (EC 2.5.1.54)
Amino Acids and Derivatives	Aromatic amino acids and derivatives	Common Pathway For Synthesis of Aromatic Compounds (DAHP synthase to chorismate)	Quinate/shikimate 5-dehydrogenase I delta (EC 1.1.1.25)
Amino Acids and Derivatives	Aromatic amino acids and derivatives	Common Pathway For Synthesis of Aromatic Compounds (DAHP synthase to chorismate)	Chorismate synthase (EC 4.2.3.5)
Amino Acids and Derivatives	Aromatic amino acids and derivatives	Common Pathway For Synthesis of Aromatic Compounds (DAHP synthase to chorismate)	Shikimate 5-dehydrogenase I gamma (EC 1.1.1.25)
Amino Acids and Derivatives	Aromatic amino acids and derivatives	Common Pathway For Synthesis of Aromatic Compounds (DAHP synthase to chorismate)	3-dehydroquinate synthase (EC 4.2.3.4)
Amino Acids and Derivatives	Aromatic amino acids and derivatives	Tryptophan synthesis	Aminodeoxychorismate lyase (EC 4.1.3.38)

Amino Acids and Derivatives	Aromatic amino acids and derivatives	Tryptophan synthesis	Anthranilate synthase, aminase component (EC 4.1.3.27)
Amino Acids and Derivatives	Aromatic amino acids and derivatives	Tryptophan synthesis	Para-aminobenzoate synthase, amidotransferase component (EC 2.6.1.85)
Amino Acids and Derivatives	Aromatic amino acids and derivatives	Tryptophan synthesis	Anthranilate phosphoribosyltransferase (EC 2.4.2.18)
Amino Acids and Derivatives	Aromatic amino acids and derivatives	Tryptophan synthesis	Indole-3-glycerol phosphate synthase (EC 4.1.1.48)
Amino Acids and Derivatives	Aromatic amino acids and derivatives	Tryptophan synthesis	Tryptophan synthase alpha chain (EC 4.2.1.20)
Amino Acids and Derivatives	Aromatic amino acids and derivatives	Tryptophan synthesis	Anthranilate synthase, amidotransferase component (EC 4.1.3.27)
Amino Acids and Derivatives	Aromatic amino acids and derivatives	Tryptophan synthesis	Phosphoribosylanthranilate isomerase (EC 5.3.1.24)
Amino Acids and Derivatives	Aromatic amino acids and derivatives	Tryptophan synthesis	Tryptophan-associated membrane protein
Amino Acids and Derivatives	Aromatic amino acids and derivatives	Tryptophan synthesis	Para-aminobenzoate synthase, aminase component (EC 2.6.1.85)
Amino Acids and Derivatives	Aromatic amino acids and derivatives	Tryptophan synthesis	Tryptophan synthase beta chain (EC 4.2.1.20)
Amino Acids and Derivatives	Aromatic amino acids and derivatives	Tryptophan synthesis	Acting phosphoribosylanthranilate isomerase (EC 5.3.1.24)
Amino Acids and Derivatives	Aromatic amino acids and derivatives	Chorismate: Intermediate for synthesis of Tryptophan, PAPA antibiotics, PABA, 3-hydroxyanthranilate and more.	Anthranilate synthase, aminase component (EC 4.1.3.27)
Amino Acids and Derivatives	Aromatic amino acids and derivatives	Chorismate: Intermediate for synthesis of Tryptophan, PAPA antibiotics, PABA, 3-hydroxyanthranilate and more.	Aminodeoxychorismate lyase (EC 4.1.3.38)
Amino Acids and Derivatives	Aromatic amino acids and derivatives	Chorismate: Intermediate for synthesis of Tryptophan, PAPA antibiotics, PABA, 3-hydroxyanthranilate and more.	Phosphoribosylformimino-5-aminoimidazole carboxamide ribotide isomerase (EC 5.3.1.16)
Amino Acids and Derivatives	Aromatic amino acids and derivatives	Chorismate: Intermediate for synthesis of Tryptophan, PAPA antibiotics, PABA, 3-hydroxyanthranilate and more.	Para-aminobenzoate synthase, amidotransferase component (EC 2.6.1.85)
Amino Acids and Derivatives	Aromatic amino acids and derivatives	Chorismate: Intermediate for synthesis of Tryptophan, PAPA antibiotics, PABA, 3-hydroxyanthranilate and more.	Anthranilate phosphoribosyltransferase (EC 2.4.2.18)
Amino Acids and Derivatives	Aromatic amino acids and derivatives	Chorismate: Intermediate for synthesis of Tryptophan, PAPA antibiotics, PABA, 3-hydroxyanthranilate and more.	Phosphoribosylanthranilate isomerase (EC 5.3.1.24)
Amino Acids and Derivatives	Aromatic amino acids and derivatives	Chorismate: Intermediate for synthesis of Tryptophan, PAPA antibiotics, PABA, 3-hydroxyanthranilate and more.	Anthranilate synthase, amidotransferase component (EC 4.1.3.27)

Amino Acids and Derivatives	Aromatic amino acids and derivatives	Chorismate: Intermediate for synthesis of Tryptophan, PAPA antibiotics, PABA, 3-hydroxyanthranilate and more.	Tryptophan synthase alpha chain (EC 4.2.1.20)
Amino Acids and Derivatives	Aromatic amino acids and derivatives	Chorismate: Intermediate for synthesis of Tryptophan, PAPA antibiotics, PABA, 3-hydroxyanthranilate and more.	Indole-3-glycerol phosphate synthase (EC 4.1.1.48)
Amino Acids and Derivatives	Aromatic amino acids and derivatives	Chorismate: Intermediate for synthesis of Tryptophan, PAPA antibiotics, PABA, 3-hydroxyanthranilate and more.	Isochorismate synthase (EC 5.4.4.2)
Amino Acids and Derivatives	Aromatic amino acids and derivatives	Chorismate: Intermediate for synthesis of Tryptophan, PAPA antibiotics, PABA, 3-hydroxyanthranilate and more.	Tryptophan synthase beta chain (EC 4.2.1.20)
Amino Acids and Derivatives	Aromatic amino acids and derivatives	Chorismate: Intermediate for synthesis of Tryptophan, PAPA antibiotics, PABA, 3-hydroxyanthranilate and more.	Para-aminobenzoate synthase, aminase component (EC 2.6.1.85)
Amino Acids and Derivatives	Proline and 4-hydroxyproline	Proline Synthesis	NADP-specific glutamate dehydrogenase (EC 1.4.1.4)
Amino Acids and Derivatives	Proline and 4-hydroxyproline	Proline Synthesis	Glutamate 5-kinase (EC 2.7.2.11)
Amino Acids and Derivatives	Proline and 4-hydroxyproline	Proline Synthesis	RNA-binding C-terminal domain PUA
Amino Acids and Derivatives	Proline and 4-hydroxyproline	Proline Synthesis	Pyrroline-5-carboxylate reductase (EC 1.5.1.2)
Amino Acids and Derivatives	Proline and 4-hydroxyproline	Proline Synthesis	Gamma-glutamyl phosphate reductase (EC 1.2.1.41)
Amino Acids and Derivatives	Proline and 4-hydroxyproline	Proline, 4-hydroxyproline uptake and utilization	Proline iminopeptidase (EC 3.4.11.5)
Amino Acids and Derivatives	Proline and 4-hydroxyproline	Proline, 4-hydroxyproline uptake and utilization	D-amino-acid oxidase (EC 1.4.3.3)
Amino Acids and Derivatives	Proline and 4-hydroxyproline	Proline, 4-hydroxyproline uptake and utilization	1-pyrroline-4-hydroxy-2-carboxylate deaminase (EC 3.5.4.22)
Amino Acids and Derivatives	Proline and 4-hydroxyproline	Proline, 4-hydroxyproline uptake and utilization	Delta-1-pyrroline-5-carboxylate dehydrogenase (EC 1.2.1.88)
Amino Acids and Derivatives	Proline and 4-hydroxyproline	Proline, 4-hydroxyproline uptake and utilization	L-Proline/Glycine betaine transporter ProP
Amino Acids and Derivatives	Proline and 4-hydroxyproline	Proline, 4-hydroxyproline uptake and utilization	4-hydroxyproline epimerase (EC 5.1.1.8)
Amino Acids and Derivatives	Proline and 4-hydroxyproline	Proline, 4-hydroxyproline uptake and utilization	Putative oxidoreductase in 4-hydroxyproline catabolic gene cluster
Metabolism of Aromatic Compounds	Peripheral pathways for catabolism of aromatic compounds	Benzoate degradation	Benzoate 1,2-dioxygenase, ferredoxin reductase component
Metabolism of Aromatic Compounds	Peripheral pathways for catabolism of aromatic compounds	Benzoate degradation	Benzoate transport protein

Metabolism of Aromatic Compounds	Peripheral pathways for catabolism of aromatic compounds	Benzoate degradation	Benzoate 1,2-dioxygenase beta subunit (EC 1.14.12.10)
Metabolism of Aromatic Compounds	Peripheral pathways for catabolism of aromatic compounds	Benzoate degradation	1,2-dihydroxycyclohexa-3,5-diene-1-carboxylate dehydrogenase (EC 1.3.1.25)
Metabolism of Aromatic Compounds	Peripheral pathways for catabolism of aromatic compounds	Benzoate degradation	benzoate MFS transporter BenK
Metabolism of Aromatic Compounds	Peripheral pathways for catabolism of aromatic compounds	Benzoate degradation	Benzoate 1,2-dioxygenase alpha subunit (EC 1.14.12.10)
Metabolism of Aromatic Compounds	Peripheral pathways for catabolism of aromatic compounds	p-Hydroxybenzoate degradation	P-hydroxybenzoate hydroxylase (EC 1.14.13.2)
Metabolism of Aromatic Compounds	Peripheral pathways for catabolism of aromatic compounds	p-Hydroxybenzoate degradation	4-hydroxybenzoate transporter
Metabolism of Aromatic Compounds	Peripheral pathways for catabolism of aromatic compounds	Quinate degradation	3-dehydroquinate dehydratase II (EC 4.2.1.10)
Metabolism of Aromatic Compounds	Metabolism of central aromatic intermediates	Catechol branch of beta-ketoadipate pathway	Catechol 1,2-dioxygenase (EC 1.13.11.1)
Metabolism of Aromatic Compounds	Metabolism of central aromatic intermediates	Catechol branch of beta-ketoadipate pathway	3-oxoadipate CoA-transferase subunit A (EC 2.8.3.6)
Metabolism of Aromatic Compounds	Metabolism of central aromatic intermediates	Catechol branch of beta-ketoadipate pathway	Catechol 1,2-dioxygenase 1 (EC 1.13.11.1)
Metabolism of Aromatic Compounds	Metabolism of central aromatic intermediates	Catechol branch of beta-ketoadipate pathway	Muconolactone isomerase (EC 5.3.3.4)
Metabolism of Aromatic Compounds	Metabolism of central aromatic intermediates	Catechol branch of beta-ketoadipate pathway	Succinyl-CoA:3-ketoacid-coenzyme A transferase subunit A (EC 2.8.3.5)
Metabolism of Aromatic Compounds	Metabolism of central aromatic intermediates	Catechol branch of beta-ketoadipate pathway	3-oxoadipate CoA-transferase subunit B (EC 2.8.3.6)
Metabolism of Aromatic Compounds	Metabolism of central aromatic intermediates	Catechol branch of beta-ketoadipate pathway	Beta-ketoadipate enol-lactone hydrolase (EC 3.1.1.24)
Metabolism of Aromatic Compounds	Metabolism of central aromatic intermediates	Catechol branch of beta-ketoadipate pathway	Muconate cycloisomerase (EC 5.5.1.1)
Metabolism of Aromatic Compounds	Metabolism of central aromatic intermediates	Catechol branch of beta-ketoadipate pathway	Succinyl-CoA:3-ketoacid-coenzyme A transferase subunit B (EC 2.8.3.5)
Metabolism of Aromatic Compounds	Metabolism of central aromatic intermediates	Salicylate and gentisate catabolism	Putative n-hydroxybenzoate hydroxylase
Metabolism of Aromatic Compounds	Metabolism of central aromatic intermediates	Salicylate and gentisate catabolism	Fumarylacetoacetate hydrolase family protein
Metabolism of Aromatic Compounds	Metabolism of central aromatic intermediates	Salicylate and gentisate catabolism	4-hydroxybenzoate transporter
Metabolism of Aromatic Compounds	Metabolism of central aromatic intermediates	Salicylate and gentisate catabolism	Gentisate 1,2-dioxygenase (EC 1.13.11.4)
Metabolism of Aromatic Compounds	Metabolism of central aromatic intermediates	Central meta-cleavage pathway of aromatic compound degradation	Catechol 2,3-dioxygenase (EC 1.13.11.2)

Metabolism of Aromatic Compounds	Metabolism of central aromatic intermediates	Central meta-cleavage pathway of aromatic compound degradation	5-carboxymethyl-2-hydroxymuconate semialdehyde dehydrogenase (EC 1.2.1.60)
Metabolism of Aromatic Compounds	Metabolism of central aromatic intermediates	Central meta-cleavage pathway of aromatic compound degradation	5-carboxymethyl-2-hydroxymuconate delta-isomerase (EC 5.3.3.10)
Metabolism of Aromatic Compounds	Metabolism of central aromatic intermediates	Central meta-cleavage pathway of aromatic compound degradation	Protocatechuate 4,5-dioxygenase beta chain (EC 1.13.11.8)
Metabolism of Aromatic Compounds	Metabolism of central aromatic intermediates	Central meta-cleavage pathway of aromatic compound degradation	2-oxo-hepta-3-ene-1,7-dioic acid hydratase (EC 4.2.-.-)
Metabolism of Aromatic Compounds	Metabolism of central aromatic intermediates	Protocatechuate branch of beta-ketoadipate pathway	Succinyl-CoA:3-ketoacid-coenzyme A transferase subunit B (EC 2.8.3.5)
Metabolism of Aromatic Compounds	Metabolism of central aromatic intermediates	Protocatechuate branch of beta-ketoadipate pathway	3-carboxy-cis,cis-muconate cycloisomerase (EC 5.5.1.2)
Metabolism of Aromatic Compounds	Metabolism of central aromatic intermediates	Protocatechuate branch of beta-ketoadipate pathway	4-carboxymuconolactone decarboxylase (EC 4.1.1.44)
Metabolism of Aromatic Compounds	Metabolism of central aromatic intermediates	Protocatechuate branch of beta-ketoadipate pathway	Succinyl-CoA:3-ketoacid-coenzyme A transferase subunit A (EC 2.8.3.5)
Metabolism of Aromatic Compounds	Metabolism of central aromatic intermediates	Protocatechuate branch of beta-ketoadipate pathway	Pca regulon regulatory protein PcaR
Metabolism of Aromatic Compounds	Metabolism of central aromatic intermediates	Protocatechuate branch of beta-ketoadipate pathway	Protocatechuate 3,4-dioxygenase alpha chain (EC 1.13.11.3)
Metabolism of Aromatic Compounds	Metabolism of central aromatic intermediates	Protocatechuate branch of beta-ketoadipate pathway	Beta-ketoadipate enol-lactone hydrolase (EC 3.1.1.24)
Metabolism of Aromatic Compounds	Metabolism of central aromatic intermediates	Protocatechuate branch of beta-ketoadipate pathway	3-oxoadipate CoA-transferase subunit B (EC 2.8.3.6)
Metabolism of Aromatic Compounds	Metabolism of central aromatic intermediates	Protocatechuate branch of beta-ketoadipate pathway	3-oxoadipate CoA-transferase subunit A (EC 2.8.3.6)
Metabolism of Aromatic Compounds	Metabolism of central aromatic intermediates	Protocatechuate branch of beta-ketoadipate pathway	Protocatechuate 3,4-dioxygenase beta chain (EC 1.13.11.3)
Metabolism of Aromatic Compounds	Metabolism of Aromatic Compounds - no subcategory	Gentisate degradation	Gentisate 1,2-dioxygenase (EC 1.13.11.4)
Metabolism of Aromatic Compounds	Metabolism of Aromatic Compounds - no subcategory	Gentisate degradation	Fumarylacetoacetate hydrolase family protein
Metabolism of Aromatic Compounds	Metabolism of Aromatic Compounds - no subcategory	Gentisate degradation	4-hydroxybenzoate transporter
Metabolism of Aromatic Compounds	Metabolism of Aromatic Compounds - no subcategory	Gentisate degradation	Putative n-hydroxybenzoate hydroxylase
Resistance to antibiotics and toxic compounds	Resistance to antibiotics	Beta-lactamase	Metal-dependent hydrolases of the beta-lactamase superfamily III
Resistance to antibiotics and toxic compounds	Resistance to antibiotics	Resistance to fluoroquinolones	DNA gyrase subunit A (EC 5.99.1.3)
Resistance to antibiotics and toxic compounds	Resistance to antibiotics	Resistance to fluoroquinolones	DNA gyrase subunit B (EC 5.99.1.3)

Resistance to antibiotics and toxic compounds	Resistance to heavy metals	Copper homeostasis	Copper resistance protein CopD
Resistance to antibiotics and toxic compounds	Resistance to heavy metals	Copper homeostasis	Copper-translocating P-type ATPase (EC 3.6.3.4)
Resistance to antibiotics and toxic compounds	Resistance to heavy metals	Copper homeostasis	Copper resistance protein CopC
Resistance to antibiotics and toxic compounds	Resistance to heavy metals	Copper homeostasis	Multidrug resistance transporter, Bcr/CflA family
Resistance to antibiotics and toxic compounds	Resistance to heavy metals	Cobalt-zinc-cadmium resistance	Transcriptional regulator, MerR family
Resistance to antibiotics and toxic compounds	Resistance to heavy metals	Cobalt-zinc-cadmium resistance	Cobalt-zinc-cadmium resistance protein CzcD
Resistance to antibiotics and toxic compounds	Resistance to heavy metals	Mercuric reductase	PF00070 family, FAD-dependent NAD(P)-disulphide oxidoreductase

The annotated genes from the genome of strain H3SJ34-1^T involving in metabolizaton of carbohydrates, amino acids, and aromatic compounds and resistance to antibiotics and heavy metals.

Category	Subcategory	Subsystem	Annotated genes and its roles
Carbohydrates	Central carbohydrate metabolism	TCA Cycle	Fumarate hydratase class I (EC 4.2.1.2)
Carbohydrates	Central carbohydrate metabolism	TCA Cycle	Isocitrate dehydrogenase [NADP] (EC 1.1.1.42)
Carbohydrates	Central carbohydrate metabolism	TCA Cycle	2-oxoglutarate dehydrogenase E1 component (EC 1.2.4.2)
Carbohydrates	Central carbohydrate metabolism	TCA Cycle	Dihydrolipoamide dehydrogenase of 2-oxoglutarate dehydrogenase (EC 1.8.1.4)
Carbohydrates	Central carbohydrate metabolism	TCA Cycle	Fumarate hydratase class II (EC 4.2.1.2)
Carbohydrates	Central carbohydrate metabolism	TCA Cycle	Aconitate hydratase (EC 4.2.1.3)
Carbohydrates	Central carbohydrate metabolism	TCA Cycle	Dihydrolipoamide succinyltransferase component (E2) of 2-oxoglutarate dehydrogenase complex (EC 2.3.1.61)
Carbohydrates	Central carbohydrate metabolism	TCA Cycle	Citrate synthase (si) (EC 2.3.3.1)
Carbohydrates	Central carbohydrate metabolism	TCA Cycle	Succinyl-CoA ligase [ADP-forming] beta chain (EC 6.2.1.5)
Carbohydrates	Central carbohydrate metabolism	TCA Cycle	Dihydrolipoamide dehydrogenase of pyruvate dehydrogenase complex (EC 1.8.1.4)
Carbohydrates	Central carbohydrate metabolism	TCA Cycle	hypothetical protein that often co-occurs with aconitase
Carbohydrates	Central carbohydrate metabolism	TCA Cycle	Succinyl-CoA ligase [ADP-forming] alpha chain (EC 6.2.1.5)
Carbohydrates	Central carbohydrate metabolism	TCA Cycle	Malate dehydrogenase (EC 1.1.1.37)
Carbohydrates	Central carbohydrate metabolism	Pyruvate metabolism II: acetyl-CoA, acetogenesis from pyruvate	Pyruvate dehydrogenase E1 component alpha subunit (EC 1.2.4.1)
Carbohydrates	Central carbohydrate metabolism	Pyruvate metabolism II: acetyl-CoA, acetogenesis from pyruvate	Acetate permease ActP (cation/acetate symporter)
Carbohydrates	Central carbohydrate metabolism	Pyruvate metabolism II: acetyl-CoA, acetogenesis from pyruvate	Dihydrolipoamide acetyltransferase component of pyruvate dehydrogenase complex (EC 2.3.1.12)
Carbohydrates	Central carbohydrate metabolism	Pyruvate metabolism II: acetyl-CoA, acetogenesis from pyruvate	Aldehyde dehydrogenase (EC 1.2.1.3)
Carbohydrates	Central carbohydrate metabolism	Pyruvate metabolism II: acetyl-CoA, acetogenesis from pyruvate	Acetate kinase (EC 2.7.2.1)
Carbohydrates	Central carbohydrate metabolism	Pyruvate metabolism II: acetyl-CoA, acetogenesis from pyruvate	Pyruvate dehydrogenase E1 component beta subunit (EC 1.2.4.1)
Carbohydrates	Central carbohydrate metabolism	Pyruvate metabolism II: acetyl-CoA, acetogenesis from pyruvate	Dihydrolipoamide dehydrogenase of pyruvate dehydrogenase complex (EC 1.8.1.4)
Carbohydrates	Central carbohydrate metabolism	Pyruvate metabolism II: acetyl-CoA, acetogenesis from pyruvate	NAD-dependent protein deacetylase of SIR2 family
Carbohydrates	Central carbohydrate metabolism	Pyruvate metabolism II: acetyl-CoA, acetogenesis from pyruvate	Phosphate acetyltransferase (EC 2.3.1.8)
Carbohydrates	Central carbohydrate metabolism	Pyruvate metabolism II: acetyl-CoA, acetogenesis from pyruvate	Acetyl-CoA synthetase (ADP-forming) alpha and beta chains, putative
Carbohydrates	Central carbohydrate metabolism	Methylglyoxal Metabolism	Aldehyde dehydrogenase (EC 1.2.1.3)
Carbohydrates	Central carbohydrate metabolism	Methylglyoxal Metabolism	Hydroxyacylglutathione hydrolase (EC 3.1.2.6)
Carbohydrates	Central carbohydrate metabolism	Methylglyoxal Metabolism	Lactoylglutathione lyase (EC 4.4.1.5)
Carbohydrates	Central carbohydrate metabolism	Glycolysis and Gluconeogenesis	Glucose-6-phosphate isomerase (EC 5.3.1.9)
Carbohydrates	Central carbohydrate metabolism	Glycolysis and Gluconeogenesis	Fructose-1,6-bisphosphatase, GlpX type (EC 3.1.3.11)
Carbohydrates	Central carbohydrate metabolism	Glycolysis and Gluconeogenesis	Fructose-1,6-bisphosphatase, type I (EC 3.1.3.11)
Carbohydrates	Central carbohydrate metabolism	Glycolysis and Gluconeogenesis	Fructose-bisphosphate aldolase class II (EC 4.1.2.13)
Carbohydrates	Central carbohydrate metabolism	Glycolysis and Gluconeogenesis	Glucokinase (EC 2.7.1.2)
Carbohydrates	Central carbohydrate metabolism	Glycolysis and Gluconeogenesis	Phosphoglycerate kinase (EC 2.7.2.3)
Carbohydrates	Central carbohydrate metabolism	Glycolysis and Gluconeogenesis	Enolase (EC 4.2.1.11)
Carbohydrates	Central carbohydrate metabolism	Glycolysis and Gluconeogenesis	Pyruvate,phosphate dikinase (EC 2.7.9.1)
Carbohydrates	Central carbohydrate metabolism	Glycolysis and Gluconeogenesis	Pyruvate kinase (EC 2.7.1.40)
Carbohydrates	Central carbohydrate metabolism	Glycolysis and Gluconeogenesis	NAD-dependent glyceraldehyde-3-phosphate dehydrogenase (EC 1.2.1.12)
Carbohydrates	Central carbohydrate metabolism	Glycolysis and Gluconeogenesis	Triosephosphate isomerase (EC 5.3.1.1)
Carbohydrates	Central carbohydrate metabolism	Glycolysis and Gluconeogenesis	Fructose-bisphosphate aldolase class I (EC 4.1.2.13)
Carbohydrates	Central carbohydrate metabolism	Glycolysis and Gluconeogenesis	Polyphosphate glucokinase (EC 2.7.1.63)
Carbohydrates	Central carbohydrate metabolism	Glycolate, glyoxylate interconversions	D-Lactate dehydrogenase, cytochrome c-dependent (EC 1.1.2.4)
Carbohydrates	Central carbohydrate metabolism	Glycolate, glyoxylate interconversions	Glycolate dehydrogenase (EC 1.1.99.14), subunit GlcD
Carbohydrates	Central carbohydrate metabolism	Glycolate, glyoxylate interconversions	Glyoxylate reductase (EC 1.1.1.79)
Carbohydrates	Central carbohydrate metabolism	Glycolate, glyoxylate interconversions	Glycolate dehydrogenase (EC 1.1.99.14), iron-sulfur subunit GlcF
Carbohydrates	Central carbohydrate metabolism	Glycolate, glyoxylate interconversions	Phosphoglycolate phosphatase (EC 3.1.3.18)
Carbohydrates	Central carbohydrate metabolism	Glycolate, glyoxylate interconversions	Hydroxypyruvate reductase (EC 1.1.1.81)
Carbohydrates	Central carbohydrate metabolism	Glycolate, glyoxylate interconversions	Glyoxylate reductase (EC 1.1.1.26)
Carbohydrates	Central carbohydrate metabolism	Glycolate, glyoxylate interconversions	Glycolate dehydrogenase (EC 1.1.99.14), FAD-binding subunit GlcE
Carbohydrates	Central carbohydrate metabolism	Dehydrogenase complexes	2-oxoglutarate dehydrogenase E1 component (EC 1.2.4.2)
Carbohydrates	Central carbohydrate metabolism	Dehydrogenase complexes	Leucine-, isoleucine-, valine-, threonine-, and alanine-binding protein

Carbohydrates	Central carbohydrate metabolism	Dehydrogenase complexes	Dihydrolipoamide acetyltransferase component of pyruvate dehydrogenase complex (EC 2.3.1.12)
Carbohydrates	Central carbohydrate metabolism	Dehydrogenase complexes	Pyruvate dehydrogenase E1 component alpha subunit (EC 1.2.4.1)
Carbohydrates	Central carbohydrate metabolism	Dehydrogenase complexes	Pyruvate dehydrogenase E1 component beta subunit (EC 1.2.4.1)
Carbohydrates	Central carbohydrate metabolism	Dehydrogenase complexes	Dihydrolipoamide dehydrogenase of 2-oxoglutarate dehydrogenase (EC 1.8.1.4)
Carbohydrates	Central carbohydrate metabolism	Dehydrogenase complexes	Cytosol aminopeptidase PepA (EC 3.4.11.1)
Carbohydrates	Central carbohydrate metabolism	Dehydrogenase complexes	Dihydrolipoamide succinyltransferase component (E2) of 2-oxoglutarate dehydrogenase complex (EC 2.3.1.61)
Carbohydrates	Central carbohydrate metabolism	Pyruvate Alanine Serine Interconversions	Omega-amino acid--pyruvate aminotransferase (EC 2.6.1.18)
Carbohydrates	Central carbohydrate metabolism	Pyruvate Alanine Serine Interconversions	Branched-chain amino acid aminotransferase (EC 2.6.1.42)
Carbohydrates	Central carbohydrate metabolism	Pyruvate Alanine Serine Interconversions	Alanine dehydrogenase (EC 1.4.1.1)
Carbohydrates	Central carbohydrate metabolism	Pyruvate Alanine Serine Interconversions	Valine--pyruvate aminotransferase (EC 2.6.1.66)
Carbohydrates	Central carbohydrate metabolism	Pyruvate Alanine Serine Interconversions	Alanine racemase (EC 5.1.1.1)
Carbohydrates	Central carbohydrate metabolism	Pentose phosphate pathway	Ribulose-phosphate 3-epimerase (EC 5.1.3.1)
Carbohydrates	Central carbohydrate metabolism	Pentose phosphate pathway	Transaldolase (EC 2.2.1.2)
Carbohydrates	Central carbohydrate metabolism	Pentose phosphate pathway	6-phosphogluconate dehydrogenase, decarboxylating (EC 1.1.1.44)
Carbohydrates	Central carbohydrate metabolism	Pentose phosphate pathway	Xylulose-5-phosphate phosphoketolase (EC 4.1.2.9)
Carbohydrates	Central carbohydrate metabolism	Pentose phosphate pathway	Ribose-phosphate pyrophosphokinase (EC 2.7.6.1)
Carbohydrates	Central carbohydrate metabolism	Pentose phosphate pathway	Fructose-6-phosphate phosphoketolase (EC 4.1.2.22)
Carbohydrates	Central carbohydrate metabolism	Pentose phosphate pathway	Transketolase (EC 2.2.1.1)
Carbohydrates	Central carbohydrate metabolism	Pentose phosphate pathway	Glucose-6-phosphate 1-dehydrogenase (EC 1.1.1.49)
Carbohydrates	Central carbohydrate metabolism	Pentose phosphate pathway	6-phosphogluconolactonase (EC 3.1.1.31), eukaryotic type
Carbohydrates	Central carbohydrate metabolism	Glyoxylate bypass	Malate synthase G (EC 2.3.3.9)
Carbohydrates	Central carbohydrate metabolism	Glyoxylate bypass	Aconitate hydratase (EC 4.2.1.3)
Carbohydrates	Central carbohydrate metabolism	Glyoxylate bypass	Citrate synthase (si) (EC 2.3.3.1)
Carbohydrates	Central carbohydrate metabolism	Glyoxylate bypass	Malate dehydrogenase (EC 1.1.1.37)
Carbohydrates	Central carbohydrate metabolism	Glyoxylate bypass	Isocitrate lyase (EC 4.1.3.1)
Carbohydrates	Central carbohydrate metabolism	Pyruvate metabolism I: anaplerotic reactions, PEP	Pyruvate kinase (EC 2.7.1.40)
Carbohydrates	Central carbohydrate metabolism	Pyruvate metabolism I: anaplerotic reactions, PEP	Pyruvate,phosphate dikinase (EC 2.7.9.1)
Carbohydrates	Central carbohydrate metabolism	Pyruvate metabolism I: anaplerotic reactions, PEP	Phosphoenolpyruvate carboxykinase [ATP] (EC 4.1.1.49)
Carbohydrates	Central carbohydrate metabolism	Pyruvate metabolism I: anaplerotic reactions, PEP	NADP-dependent malic enzyme (EC 1.1.1.40)
Carbohydrates	Monosaccharides	Mannose Metabolism	Phosphomannomutase (EC 5.4.2.8)
Carbohydrates	Monosaccharides	Mannose Metabolism	Mannose-6-phosphate isomerase (EC 5.3.1.8)
Carbohydrates	Monosaccharides	Mannose Metabolism	Beta-mannosidase (EC 3.2.1.25)
Carbohydrates	Monosaccharides	D-gluconate and ketogluconates metabolism	Gluconate 2-dehydrogenase (EC 1.1.99.3), membrane-bound, flavoprotein
Carbohydrates	Monosaccharides	D-gluconate and ketogluconates metabolism	Gluconate dehydratase (EC 4.2.1.39)
Carbohydrates	Monosaccharides	D-gluconate and ketogluconates metabolism	6-phosphogluconate dehydrogenase, decarboxylating (EC 1.1.1.44)
Carbohydrates	Monosaccharides	D-gluconate and ketogluconates metabolism	Gluconokinase (EC 2.7.1.12)
Carbohydrates	Monosaccharides	D-gluconate and ketogluconates metabolism	Gluconate 2-dehydrogenase (EC 1.1.99.3), membrane-bound, gamma subunit
Carbohydrates	Di- and oligosaccharides	Trehalose Uptake and Utilization	Beta-phosphoglucomutase (EC 5.4.2.6)
Carbohydrates	Di- and oligosaccharides	Trehalose Uptake and Utilization	Trehalose phosphorylase (EC 2.4.1.64)
Carbohydrates	Di- and oligosaccharides	Trehalose Biosynthesis	Malto-oligosyltrehalose trehalohydrolase (EC 3.2.1.141)
Carbohydrates	Di- and oligosaccharides	Trehalose Biosynthesis	Trehalose phosphorylase (EC 2.4.1.64)
Carbohydrates	Di- and oligosaccharides	Trehalose Biosynthesis	Alpha,alpha-trehalose-phosphate synthase [UDP-forming] (EC 2.4.1.15)
Carbohydrates	Di- and oligosaccharides	Trehalose Biosynthesis	Glucoamylase (EC 3.2.1.3)
Carbohydrates	Di- and oligosaccharides	Trehalose Biosynthesis	Trehalose synthase (EC 5.4.99.16)
Carbohydrates	Di- and oligosaccharides	Trehalose Biosynthesis	Trehalose-6-phosphate phosphatase (EC 3.1.3.12)
Carbohydrates	Di- and oligosaccharides	Trehalose Biosynthesis	Malto-oligosyltrehalose synthase (EC 5.4.99.15)
Carbohydrates	Di- and oligosaccharides	Trehalose Biosynthesis	Alpha-amylase (EC 3.2.1.1)
Carbohydrates	Di- and oligosaccharides	Trehalose Biosynthesis	1,4-alpha-glucan (glycogen) branching enzyme, GH-13-type (EC 2.4.1.18)
Carbohydrates	Di- and oligosaccharides	Maltose and Maltodextrin Utilization	Alpha-amylase (EC 3.2.1.1)
Carbohydrates	Di- and oligosaccharides	Maltose and Maltodextrin Utilization	Malto-oligosyltrehalose synthase (EC 5.4.99.15)
Carbohydrates	Di- and oligosaccharides	Maltose and Maltodextrin Utilization	Maltose O-acetyltransferase (EC 2.3.1.79)
Carbohydrates	Di- and oligosaccharides	Maltose and Maltodextrin Utilization	Maltose phosphorylase (EC 2.4.1.8)

Carbohydrates	Di- and oligosaccharides	Maltose and Maltodextrin Utilization	Beta-phosphoglucomutase (EC 5.4.2.6)
Carbohydrates	Di- and oligosaccharides	Maltose and Maltodextrin Utilization	Glucoamylase (EC 3.2.1.3)
Carbohydrates	Di- and oligosaccharides	Maltose and Maltodextrin Utilization	4-alpha-glucanotransferase (amylomaltase) (EC 2.4.1.25)
Carbohydrates	Di- and oligosaccharides	Maltose and Maltodextrin Utilization	Maltodextrin glucosidase (EC 3.2.1.20)
Carbohydrates	Di- and oligosaccharides	Maltose and Maltodextrin Utilization	Glycogen phosphorylase (EC 2.4.1.1)
Carbohydrates	Polysaccharides	Glycogen metabolism	1,4-alpha-glucan (glycogen) branching enzyme, GH-13-type (EC 2.4.1.18)
Carbohydrates	Polysaccharides	Glycogen metabolism	Glycogen synthase, ADP-glucose transglucosylase (EC 2.4.1.21)
Carbohydrates	Polysaccharides	Glycogen metabolism	Glycogen phosphorylase (EC 2.4.1.1)
Carbohydrates	Polysaccharides	Glycogen metabolism	4-alpha-glucanotransferase (amylomaltase) (EC 2.4.1.25)
Carbohydrates	Organic acids	Methylcitrate cycle	2-methylcitrate synthase (EC 2.3.3.5)
Carbohydrates	Organic acids	Methylcitrate cycle	Methylisocitrate lyase (EC 4.1.3.30)
Carbohydrates	Organic acids	Methylcitrate cycle	2-methylcitrate dehydratase (EC 4.2.1.79)
Carbohydrates	Organic acids	Lactate utilization	L-lactate dehydrogenase (EC 1.1.2.3)
Carbohydrates	Organic acids	Lactate utilization	Predicted L-lactate dehydrogenase, hypothetical protein subunit YkgG
Carbohydrates	Organic acids	Lactate utilization	Predicted L-lactate dehydrogenase, Iron-sulfur cluster-binding subunit YkgF
Carbohydrates	Organic acids	Lactate utilization	Predicted L-lactate dehydrogenase, Fe-S oxidoreductase subunit YkgE
Amino Acids and Derivatives	Lysine, threonine, methionine, and cysteine	Lysine Biosynthesis DAP Pathway, GJO scratch	4-hydroxy-tetrahydrodipicolinate reductase (EC 1.17.1.8)
Amino Acids and Derivatives	Lysine, threonine, methionine, and cysteine	Lysine Biosynthesis DAP Pathway, GJO scratch	N-acetyl-L,L-diaminopimelate deacetylase (EC 3.5.1.47)
Amino Acids and Derivatives	Lysine, threonine, methionine, and cysteine	Lysine Biosynthesis DAP Pathway, GJO scratch	Aspartokinase (EC 2.7.2.4)
Amino Acids and Derivatives	Lysine, threonine, methionine, and cysteine	Lysine Biosynthesis DAP Pathway, GJO scratch	Diaminopimelate epimerase (EC 5.1.1.7)
Amino Acids and Derivatives	Lysine, threonine, methionine, and cysteine	Lysine Biosynthesis DAP Pathway, GJO scratch	Diaminopimelate decarboxylase (EC 4.1.1.20)
Amino Acids and Derivatives	Lysine, threonine, methionine, and cysteine	Lysine Biosynthesis DAP Pathway, GJO scratch	4-hydroxy-tetrahydrodipicolinate synthase (EC 4.3.3.7)
Amino Acids and Derivatives	Lysine, threonine, methionine, and cysteine	Lysine Biosynthesis DAP Pathway, GJO scratch	N-succinyl-L,L-diaminopimelate desuccinylase (EC 3.5.1.18)
Amino Acids and Derivatives	Lysine, threonine, methionine, and cysteine	Lysine Biosynthesis DAP Pathway, GJO scratch	2,3,4,5-tetrahydropyridine-2,6-dicarboxylate N-succinyltransferase (EC 2.3.1.117)
Amino Acids and Derivatives	Lysine, threonine, methionine, and cysteine	Lysine Biosynthesis DAP Pathway, GJO scratch	Aspartate-semialdehyde dehydrogenase (EC 1.2.1.11)
Amino Acids and Derivatives	Lysine, threonine, methionine, and cysteine	Methionine Biosynthesis	S-adenosylmethionine synthetase (EC 2.5.1.6)
Amino Acids and Derivatives	Lysine, threonine, methionine, and cysteine	Methionine Biosynthesis	Transcriptional activator MetR
Amino Acids and Derivatives	Lysine, threonine, methionine, and cysteine	Methionine Biosynthesis	Serine acetyltransferase (EC 2.3.1.30)
Amino Acids and Derivatives	Lysine, threonine, methionine, and cysteine	Methionine Biosynthesis	Cysteine synthase (EC 2.5.1.47)
Amino Acids and Derivatives	Lysine, threonine, methionine, and cysteine	Methionine Biosynthesis	Cystathionine beta-lyase (EC 4.4.1.8)
Amino Acids and Derivatives	Lysine, threonine, methionine, and cysteine	Methionine Biosynthesis	5-methyltetrahydropteroyltriglutamate--homocysteine methyltransferase (EC 2.1.1.14)
Amino Acids and Derivatives	Lysine, threonine, methionine, and cysteine	Methionine Biosynthesis	Homoserine kinase (EC 2.7.1.39)
Amino Acids and Derivatives	Lysine, threonine, methionine, and cysteine	Methionine Biosynthesis	O-succinylhomoserine sulfhydrylase (EC 2.5.1.48)

Amino Acids and Derivatives	Lysine, threonine, methionine, and cysteine	Methionine Biosynthesis	Cystathionine gamma-lyase (EC 4.4.1.1)
Amino Acids and Derivatives	Lysine, threonine, methionine, and cysteine	Methionine Biosynthesis	O-acetylhomoserine sulfhydrylase (EC 2.5.1.49)
Amino Acids and Derivatives	Lysine, threonine, methionine, and cysteine	Methionine Biosynthesis	5,10-methylenetetrahydrofolate reductase (EC 1.5.1.20)
Amino Acids and Derivatives	Lysine, threonine, methionine, and cysteine	Methionine Biosynthesis	Cystathionine beta-synthase (EC 4.2.1.22)
Amino Acids and Derivatives	Lysine, threonine, methionine, and cysteine	Methionine Biosynthesis	Homoserine dehydrogenase (EC 1.1.1.3)
Amino Acids and Derivatives	Lysine, threonine, methionine, and cysteine	Methionine Biosynthesis	Homoserine O-acetyltransferase (EC 2.3.1.31)
Amino Acids and Derivatives	Lysine, threonine, methionine, and cysteine	Methionine Biosynthesis	5-methyltetrahydrofolate--homocysteine methyltransferase (EC 2.1.1.13)
Amino Acids and Derivatives	Lysine, threonine, methionine, and cysteine	Methionine Biosynthesis	Adenosylhomocysteinase (EC 3.3.1.1)
Amino Acids and Derivatives	Lysine, threonine, methionine, and cysteine	Threonine degradation	Threonine dehydrogenase and related Zn-dependent dehydrogenases
Amino Acids and Derivatives	Lysine, threonine, methionine, and cysteine	Threonine degradation	FIG003492: Threonine dehydrogenase and related Zn-dependent dehydrogenases
Amino Acids and Derivatives	Lysine, threonine, methionine, and cysteine	Lysine Biosynthesis DAP Pathway	Diaminopimelate decarboxylase (EC 4.1.1.20)
Amino Acids and Derivatives	Lysine, threonine, methionine, and cysteine	Lysine Biosynthesis DAP Pathway	Diaminopimelate epimerase (EC 5.1.1.7)
Amino Acids and Derivatives	Lysine, threonine, methionine, and cysteine	Lysine Biosynthesis DAP Pathway	Aspartokinase (EC 2.7.2.4)
Amino Acids and Derivatives	Lysine, threonine, methionine, and cysteine	Lysine Biosynthesis DAP Pathway	N-acetyl-L,L-diaminopimelate deacetylase (EC 3.5.1.47)
Amino Acids and Derivatives	Lysine, threonine, methionine, and cysteine	Lysine Biosynthesis DAP Pathway	4-hydroxy-tetrahydrodipicolinate reductase (EC 1.17.1.8)
Amino Acids and Derivatives	Lysine, threonine, methionine, and cysteine	Lysine Biosynthesis DAP Pathway	Aspartate-semialdehyde dehydrogenase (EC 1.2.1.11)
Amino Acids and Derivatives	Lysine, threonine, methionine, and cysteine	Lysine Biosynthesis DAP Pathway	2,3,4,5-tetrahydropyridine-2,6-dicarboxylate N-succinyltransferase (EC 2.3.1.117)
Amino Acids and Derivatives	Lysine, threonine, methionine, and cysteine	Lysine Biosynthesis DAP Pathway	4-hydroxy-tetrahydrodipicolinate synthase (EC 4.3.3.7)
Amino Acids and Derivatives	Lysine, threonine, methionine, and cysteine	Lysine Biosynthesis DAP Pathway	N-succinyl-L,L-diaminopimelate desuccinylase (EC 3.5.1.18)
Amino Acids and Derivatives	Lysine, threonine, methionine, and cysteine	Threonine and Homoserine Biosynthesis	Aspartokinase (EC 2.7.2.4)
Amino Acids and Derivatives	Lysine, threonine, methionine, and cysteine	Threonine and Homoserine Biosynthesis	Threonine synthase (EC 4.2.3.1)
Amino Acids and Derivatives	Lysine, threonine, methionine, and cysteine	Threonine and Homoserine Biosynthesis	Aspartate aminotransferase (EC 2.6.1.1)
Amino Acids and Derivatives	Lysine, threonine, methionine, and cysteine	Threonine and Homoserine Biosynthesis	Homoserine dehydrogenase (EC 1.1.1.3)
Amino Acids and Derivatives	Lysine, threonine, methionine, and cysteine	Threonine and Homoserine Biosynthesis	Aspartate-semialdehyde dehydrogenase (EC 1.2.1.11)
Amino Acids and Derivatives	Lysine, threonine, methionine, and cysteine	Threonine and Homoserine Biosynthesis	Homoserine kinase (EC 2.7.1.39)

Amino Acids and Derivatives	Lysine, threonine, methionine, and cysteine	Cysteine Biosynthesis	Sulfate adenylyltransferase subunit 1 (EC 2.7.7.4)
Amino Acids and Derivatives	Lysine, threonine, methionine, and cysteine	Cysteine Biosynthesis	Serine acetyltransferase (EC 2.3.1.30)
Amino Acids and Derivatives	Lysine, threonine, methionine, and cysteine	Cysteine Biosynthesis	Sulfate and thiosulfate import ATP-binding protein CysA (EC 3.6.3.25)
Amino Acids and Derivatives	Lysine, threonine, methionine, and cysteine	Cysteine Biosynthesis	Cystathionine gamma-lyase (EC 4.4.1.1)
Amino Acids and Derivatives	Lysine, threonine, methionine, and cysteine	Cysteine Biosynthesis	Cystathionine beta-synthase (EC 4.2.1.22)
Amino Acids and Derivatives	Lysine, threonine, methionine, and cysteine	Cysteine Biosynthesis	Sulfate transport system permease protein CysW
Amino Acids and Derivatives	Lysine, threonine, methionine, and cysteine	Cysteine Biosynthesis	Sulfite reductase [NADPH] hemoprotein beta-component (EC 1.8.1.2)
Amino Acids and Derivatives	Lysine, threonine, methionine, and cysteine	Cysteine Biosynthesis	Sulfate adenylyltransferase subunit 2 (EC 2.7.7.4)
Amino Acids and Derivatives	Lysine, threonine, methionine, and cysteine	Cysteine Biosynthesis	Cysteine synthase (EC 2.5.1.47)
Amino Acids and Derivatives	Lysine, threonine, methionine, and cysteine	Cysteine Biosynthesis	Sulfate permease
Amino Acids and Derivatives	Lysine, threonine, methionine, and cysteine	Cysteine Biosynthesis	Adenylylsulfate kinase (EC 2.7.1.25)
Amino Acids and Derivatives	Lysine, threonine, methionine, and cysteine	Cysteine Biosynthesis	Sulfate transport system permease protein CysT
Amino Acids and Derivatives	Lysine, threonine, methionine, and cysteine	Cysteine Biosynthesis	Sulfate and thiosulfate binding protein CysP
Amino Acids and Derivatives	Branched-chain amino acids	Leucine Biosynthesis	3-isopropylmalate dehydratase small subunit (EC 4.2.1.33)
Amino Acids and Derivatives	Branched-chain amino acids	Leucine Biosynthesis	Branched-chain amino acid aminotransferase (EC 2.6.1.42)
Amino Acids and Derivatives	Branched-chain amino acids	Leucine Biosynthesis	3-isopropylmalate dehydrogenase (EC 1.1.1.85)
Amino Acids and Derivatives	Branched-chain amino acids	Leucine Biosynthesis	3-isopropylmalate dehydratase large subunit (EC 4.2.1.33)
Amino Acids and Derivatives	Branched-chain amino acids	Leucine Biosynthesis	2-isopropylmalate synthase (EC 2.3.3.13)
Amino Acids and Derivatives	Branched-chain amino acids	Branched-Chain Amino Acid Biosynthesis	(R)-citramalate synthase (EC 2.3.1.182)
Amino Acids and Derivatives	Branched-chain amino acids	Branched-Chain Amino Acid Biosynthesis	Threonine dehydratase biosynthetic (EC 4.3.1.19)
Amino Acids and Derivatives	Branched-chain amino acids	Branched-Chain Amino Acid Biosynthesis	Valine--pyruvate aminotransferase (EC 2.6.1.66)
Amino Acids and Derivatives	Branched-chain amino acids	Branched-Chain Amino Acid Biosynthesis	2-isopropylmalate synthase (EC 2.3.3.13)
Amino Acids and Derivatives	Branched-chain amino acids	Branched-Chain Amino Acid Biosynthesis	Branched-chain amino acid aminotransferase (EC 2.6.1.42)
Amino Acids and Derivatives	Branched-chain amino acids	Branched-Chain Amino Acid Biosynthesis	Dihydroxy-acid dehydratase (EC 4.2.1.9)
Amino Acids and Derivatives	Branched-chain amino acids	Branched-Chain Amino Acid Biosynthesis	3-isopropylmalate dehydratase small subunit (EC 4.2.1.33)

Amino Acids and Derivatives	Branched-chain amino acids	Branched-Chain Amino Acid Biosynthesis	Acetolactate synthase large subunit (EC 2.2.1.6)
Amino Acids and Derivatives	Branched-chain amino acids	Branched-Chain Amino Acid Biosynthesis	3-isopropylmalate dehydratase large subunit (EC 4.2.1.33)
Amino Acids and Derivatives	Branched-chain amino acids	Branched-Chain Amino Acid Biosynthesis	Acetolactate synthase small subunit (EC 2.2.1.6)
Amino Acids and Derivatives	Branched-chain amino acids	Branched-Chain Amino Acid Biosynthesis	3-isopropylmalate dehydrogenase (EC 1.1.1.85)
Amino Acids and Derivatives	Branched-chain amino acids	Leucine Degradation and HMG-CoA Metabolism	Methylcrotonyl-CoA carboxylase carboxyl transferase subunit (EC 6.4.1.4)
Amino Acids and Derivatives	Branched-chain amino acids	Leucine Degradation and HMG-CoA Metabolism	Isovaleryl-CoA dehydrogenase (EC 1.3.8.4)
Amino Acids and Derivatives	Branched-chain amino acids	Leucine Degradation and HMG-CoA Metabolism	Hydroxymethylglutaryl-CoA lyase (EC 4.1.3.4)
Amino Acids and Derivatives	Branched-chain amino acids	Leucine Degradation and HMG-CoA Metabolism	Branched-chain amino acid aminotransferase (EC 2.6.1.42)
Amino Acids and Derivatives	Branched-chain amino acids	Leucine Degradation and HMG-CoA Metabolism	Methylcrotonyl-CoA carboxylase biotin-containing subunit (EC 6.4.1.4)
Amino Acids and Derivatives	Branched-chain amino acids	Leucine Degradation and HMG-CoA Metabolism	Succinyl-CoA:3-ketoacid-coenzyme A transferase subunit A (EC 2.8.3.5)
Amino Acids and Derivatives	Branched-chain amino acids	Leucine Degradation and HMG-CoA Metabolism	Succinyl-CoA:3-ketoacid-coenzyme A transferase subunit B (EC 2.8.3.5)
Amino Acids and Derivatives	Branched-chain amino acids	Leucine Degradation and HMG-CoA Metabolism	Methylglutaconyl-CoA hydratase (EC 4.2.1.18)
Amino Acids and Derivatives	Branched-chain amino acids	Leucine Degradation and HMG-CoA Metabolism	Branched-chain acyl-CoA dehydrogenase (EC 1.3.99.12)
Amino Acids and Derivatives	Branched-chain amino acids	Valine degradation	Branched-chain amino acid aminotransferase (EC 2.6.1.42)
Amino Acids and Derivatives	Branched-chain amino acids	Valine degradation	Methylmalonate-semialdehyde dehydrogenase (EC 1.2.1.27)
Amino Acids and Derivatives	Branched-chain amino acids	Valine degradation	3-hydroxyisobutyryl-CoA hydrolase (EC 3.1.2.4)
Amino Acids and Derivatives	Branched-chain amino acids	Valine degradation	3-hydroxyacyl-CoA dehydrogenase (EC 1.1.1.35)
Amino Acids and Derivatives	Branched-chain amino acids	Valine degradation	3-hydroxyisobutyrate dehydrogenase (EC 1.1.1.31)
Amino Acids and Derivatives	Branched-chain amino acids	Valine degradation	Branched-chain acyl-CoA dehydrogenase (EC 1.3.99.12)
Amino Acids and Derivatives	Branched-chain amino acids	Valine degradation	Enoyl-CoA hydratase (EC 4.2.1.17)
Amino Acids and Derivatives	Branched-chain amino acids	HMG CoA Synthesis	Methylcrotonyl-CoA carboxylase carboxyl transferase subunit (EC 6.4.1.4)
Amino Acids and Derivatives	Branched-chain amino acids	HMG CoA Synthesis	Isovaleryl-CoA dehydrogenase (EC 1.3.8.4)
Amino Acids and Derivatives	Branched-chain amino acids	HMG CoA Synthesis	Methylcrotonyl-CoA carboxylase biotin-containing subunit (EC 6.4.1.4)
Amino Acids and Derivatives	Branched-chain amino acids	HMG CoA Synthesis	Methylglutaconyl-CoA hydratase (EC 4.2.1.18)
Amino Acids and Derivatives	Branched-chain amino acids	HMG CoA Synthesis	Hydroxymethylglutaryl-CoA lyase (EC 4.1.3.4)

Amino Acids and Derivatives	Amino Acids and Derivatives - no subcategory	Creatine and Creatinine Degradation	N-carbamoylsarcosine amidase (EC 3.5.1.59)
Amino Acids and Derivatives	Amino Acids and Derivatives - no subcategory	Creatine and Creatinine Degradation	N-methylhydantoinase B (EC 3.5.2.14)
Amino Acids and Derivatives	Amino Acids and Derivatives - no subcategory	Creatine and Creatinine Degradation	N-methylhydantoinase A (EC 3.5.2.14)
Amino Acids and Derivatives	Amino Acids and Derivatives - no subcategory	Creatine and Creatinine Degradation	Creatinine amidohydrolase (EC 3.5.2.10)
Amino Acids and Derivatives	Amino Acids and Derivatives - no subcategory	Creatine and Creatinine Degradation	Cytosine deaminase (EC 3.5.4.1)
Amino Acids and Derivatives	Arginine; urea cycle, polyamines	Urease subunits	Urease accessory protein UreD
Amino Acids and Derivatives	Arginine; urea cycle, polyamines	Urease subunits	Urease accessory protein UreF
Amino Acids and Derivatives	Arginine; urea cycle, polyamines	Urease subunits	Urease accessory protein UreE
Amino Acids and Derivatives	Arginine; urea cycle, polyamines	Urease subunits	Urease beta subunit (EC 3.5.1.5)
Amino Acids and Derivatives	Arginine; urea cycle, polyamines	Urease subunits	Urease alpha subunit (EC 3.5.1.5)
Amino Acids and Derivatives	Arginine; urea cycle, polyamines	Urease subunits	Urease gamma subunit (EC 3.5.1.5)
Amino Acids and Derivatives	Arginine; urea cycle, polyamines	Urease subunits	Urease accessory protein UreG
Amino Acids and Derivatives	Arginine; urea cycle, polyamines	Urea carboxylase and Allophanate hydrolase cluster	Allophanate hydrolase (EC 3.5.1.54)
Amino Acids and Derivatives	Arginine; urea cycle, polyamines	Urea carboxylase and Allophanate hydrolase cluster	Biotin carboxylase (EC 6.3.4.14)
Amino Acids and Derivatives	Arginine; urea cycle, polyamines	Urea carboxylase and Allophanate hydrolase cluster	Biotin carboxyl carrier protein
Amino Acids and Derivatives	Arginine; urea cycle, polyamines	Urea carboxylase and Allophanate hydrolase cluster	Urea carboxylase (EC 6.3.4.6)
Amino Acids and Derivatives	Arginine; urea cycle, polyamines	Urea decomposition	Urea ABC transporter, ATPase protein UrtD
Amino Acids and Derivatives	Arginine; urea cycle, polyamines	Urea decomposition	Allophanate hydrolase (EC 3.5.1.54)
Amino Acids and Derivatives	Arginine; urea cycle, polyamines	Urea decomposition	Urease gamma subunit (EC 3.5.1.5)
Amino Acids and Derivatives	Arginine; urea cycle, polyamines	Urea decomposition	Urease accessory protein UreG
Amino Acids and Derivatives	Arginine; urea cycle, polyamines	Urea decomposition	Urea ABC transporter, permease protein UrtC
Amino Acids and Derivatives	Arginine; urea cycle, polyamines	Urea decomposition	Urease beta subunit (EC 3.5.1.5)
Amino Acids and Derivatives	Arginine; urea cycle, polyamines	Urea decomposition	Urea ABC transporter, ATPase protein UrtE
Amino Acids and Derivatives	Arginine; urea cycle, polyamines	Urea decomposition	Urease accessory protein UreD
Amino Acids and Derivatives	Arginine; urea cycle, polyamines	Urea decomposition	Urea carboxylase-related ABC transporter, permease protein

Amino Acids and Derivatives	Arginine; urea cycle, polyamines	Urea decomposition	Urea carboxylase (EC 6.3.4.6)
Amino Acids and Derivatives	Arginine; urea cycle, polyamines	Urea decomposition	Urea ABC transporter, permease protein UrtB
Amino Acids and Derivatives	Arginine; urea cycle, polyamines	Urea decomposition	Urease accessory protein UreE
Amino Acids and Derivatives	Arginine; urea cycle, polyamines	Urea decomposition	Urease accessory protein UreF
Amino Acids and Derivatives	Arginine; urea cycle, polyamines	Urea decomposition	Urease alpha subunit (EC 3.5.1.5)
Amino Acids and Derivatives	Arginine; urea cycle, polyamines	Polyamine Metabolism	ABC transporter, periplasmic spermidine putrescine-binding protein PotD (TC 3.A.1.11.1)
Amino Acids and Derivatives	Arginine; urea cycle, polyamines	Polyamine Metabolism	Putrescine transport system permease protein PotH (TC 3.A.1.11.2)
Amino Acids and Derivatives	Arginine; urea cycle, polyamines	Polyamine Metabolism	Spermidine synthase (EC 2.5.1.16)
Amino Acids and Derivatives	Arginine; urea cycle, polyamines	Polyamine Metabolism	Putrescine transport ATP-binding protein PotG (TC 3.A.1.11.2)
Amino Acids and Derivatives	Arginine; urea cycle, polyamines	Polyamine Metabolism	Putrescine transport ATP-binding protein PotA (TC 3.A.1.11.1)
Amino Acids and Derivatives	Arginine; urea cycle, polyamines	Polyamine Metabolism	Putrescine transport system permease protein PotI (TC 3.A.1.11.2)
Amino Acids and Derivatives	Arginine; urea cycle, polyamines	Polyamine Metabolism	Putrescine ABC transporter putrescine-binding protein PotF (TC 3.A.1.11.2)
Amino Acids and Derivatives	Arginine; urea cycle, polyamines	Polyamine Metabolism	Ornithine decarboxylase (EC 4.1.1.17)
Amino Acids and Derivatives	Arginine; urea cycle, polyamines	Arginine and Ornithine Degradation	Ornithine carbamoyltransferase (EC 2.1.3.3)
Amino Acids and Derivatives	Arginine; urea cycle, polyamines	Arginine and Ornithine Degradation	Arginase (EC 3.5.3.1)
Amino Acids and Derivatives	Arginine; urea cycle, polyamines	Arginine and Ornithine Degradation	Delta-1-pyrroline-5-carboxylate dehydrogenase (EC 1.2.1.88)
Amino Acids and Derivatives	Arginine; urea cycle, polyamines	Arginine and Ornithine Degradation	Ornithine decarboxylase (EC 4.1.1.17)
Amino Acids and Derivatives	Arginine; urea cycle, polyamines	Arginine and Ornithine Degradation	NADP-specific glutamate dehydrogenase (EC 1.4.1.4)
Amino Acids and Derivatives	Arginine; urea cycle, polyamines	Arginine and Ornithine Degradation	Ornithine cyclodeaminase (EC 4.3.1.12)
Amino Acids and Derivatives	Alanine, serine, and glycine	Glycine and Serine Utilization	Glycine dehydrogenase [decarboxylating] (glycine cleavage system P2 protein) (EC 1.4.4.2)
Amino Acids and Derivatives	Alanine, serine, and glycine	Glycine and Serine Utilization	D-3-phosphoglycerate dehydrogenase (EC 1.1.1.95)
Amino Acids and Derivatives	Alanine, serine, and glycine	Glycine and Serine Utilization	Cystathionine gamma-lyase (EC 4.4.1.1)
Amino Acids and Derivatives	Alanine, serine, and glycine	Glycine and Serine Utilization	Aminomethyltransferase (glycine cleavage system T protein) (EC 2.1.2.10)
Amino Acids and Derivatives	Alanine, serine, and glycine	Glycine and Serine Utilization	Phosphoserine aminotransferase (EC 2.6.1.52)
Amino Acids and Derivatives	Alanine, serine, and glycine	Glycine and Serine Utilization	Glycine cleavage system H protein

Amino Acids and Derivatives	Alanine, serine, and glycine	Glycine and Serine Utilization	Seryl-tRNA synthetase (EC 6.1.1.11)
Amino Acids and Derivatives	Alanine, serine, and glycine	Glycine and Serine Utilization	Cystathionine beta-synthase (EC 4.2.1.22)
Amino Acids and Derivatives	Alanine, serine, and glycine	Glycine and Serine Utilization	Glycine dehydrogenase [decarboxylating] (glycine cleavage system P1 protein) (EC 1.4.4.2)
Amino Acids and Derivatives	Alanine, serine, and glycine	Glycine and Serine Utilization	Serine hydroxymethyltransferase (EC 2.1.2.1)
Amino Acids and Derivatives	Alanine, serine, and glycine	Glycine and Serine Utilization	Phosphoserine phosphatase (EC 3.1.3.3)
Amino Acids and Derivatives	Alanine, serine, and glycine	Glycine Biosynthesis	Serine hydroxymethyltransferase (EC 2.1.2.1)
Amino Acids and Derivatives	Alanine, serine, and glycine	Serine Biosynthesis	Phosphoserine phosphatase (EC 3.1.3.3)
Amino Acids and Derivatives	Alanine, serine, and glycine	Serine Biosynthesis	D-3-phosphoglycerate dehydrogenase (EC 1.1.1.95)
Amino Acids and Derivatives	Alanine, serine, and glycine	Serine Biosynthesis	Serine hydroxymethyltransferase (EC 2.1.2.1)
Amino Acids and Derivatives	Alanine, serine, and glycine	Serine Biosynthesis	Phosphoserine aminotransferase (EC 2.6.1.52)
Amino Acids and Derivatives	Alanine, serine, and glycine	Glycine cleavage system	Glycine cleavage system H protein
Amino Acids and Derivatives	Alanine, serine, and glycine	Glycine cleavage system	Aminomethyltransferase (glycine cleavage system T protein) (EC 2.1.2.10)
Amino Acids and Derivatives	Alanine, serine, and glycine	Glycine cleavage system	Glycine dehydrogenase [decarboxylating] (glycine cleavage system P2 protein) (EC 1.4.4.2)
Amino Acids and Derivatives	Alanine, serine, and glycine	Glycine cleavage system	Glycine dehydrogenase [decarboxylating] (glycine cleavage system P1 protein) (EC 1.4.4.2)
Amino Acids and Derivatives	Alanine, serine, and glycine	Alanine biosynthesis	Cysteine desulfurase (EC 2.8.1.7)
Amino Acids and Derivatives	Alanine, serine, and glycine	Alanine biosynthesis	Ferredoxin, 2Fe-2S
Amino Acids and Derivatives	Alanine, serine, and glycine	Alanine biosynthesis	Valine--pyruvate aminotransferase (EC 2.6.1.66)
Amino Acids and Derivatives	Alanine, serine, and glycine	Alanine biosynthesis	Alanine racemase (EC 5.1.1.1)
Amino Acids and Derivatives	Alanine, serine, and glycine	Alanine biosynthesis	Branched-chain amino acid aminotransferase (EC 2.6.1.42)
Amino Acids and Derivatives	Proline and 4-hydroxyproline	Proline Synthesis	Gamma-glutamyl phosphate reductase (EC 1.2.1.41)
Amino Acids and Derivatives	Proline and 4-hydroxyproline	Proline Synthesis	Pyrroline-5-carboxylate reductase (EC 1.5.1.2)
Amino Acids and Derivatives	Proline and 4-hydroxyproline	Proline Synthesis	RNA-binding C-terminal domain PUA
Amino Acids and Derivatives	Proline and 4-hydroxyproline	Proline Synthesis	NADP-specific glutamate dehydrogenase (EC 1.4.1.4)
Amino Acids and Derivatives	Proline and 4-hydroxyproline	Proline Synthesis	Glutamate 5-kinase (EC 2.7.2.11)
Amino Acids and Derivatives	Proline and 4-hydroxyproline	Proline, 4-hydroxyproline uptake and utilization	Delta-1-pyrroline-5-carboxylate dehydrogenase (EC 1.2.1.88)

Amino Acids and Derivatives	Proline and 4-hydroxyproline	Proline, 4-hydroxyproline uptake and utilization	Proline iminopeptidase (EC 3.4.11.5)
Amino Acids and Derivatives	Histidine Metabolism	Histidine Biosynthesis	Phosphoribosyl-ATP pyrophosphatase (EC 3.6.1.31)
Amino Acids and Derivatives	Histidine Metabolism	Histidine Biosynthesis	Imidazoleglycerol-phosphate dehydratase (EC 4.2.1.19)
Amino Acids and Derivatives	Histidine Metabolism	Histidine Biosynthesis	Phosphoribosyl-AMP cyclohydrolase (EC 3.5.4.19)
Amino Acids and Derivatives	Histidine Metabolism	Histidine Biosynthesis	Histidinol dehydrogenase (EC 1.1.1.23)
Amino Acids and Derivatives	Histidine Metabolism	Histidine Biosynthesis	Phosphoribosylformimino-5-aminoimidazole carboxamide ribotide isomerase (EC 5.3.1.16)
Amino Acids and Derivatives	Histidine Metabolism	Histidine Biosynthesis	Histidinol-phosphatase [alternative form] (EC 3.1.3.15)
Amino Acids and Derivatives	Aromatic amino acids and derivatives	Common Pathway For Synthesis of Aromatic Compounds (DAHP synthase to chorismate)	Chorismate synthase (EC 4.2.3.5)
Amino Acids and Derivatives	Aromatic amino acids and derivatives	Common Pathway For Synthesis of Aromatic Compounds (DAHP synthase to chorismate)	3-dehydroquinate synthase (EC 4.2.3.4)
Amino Acids and Derivatives	Aromatic amino acids and derivatives	Common Pathway For Synthesis of Aromatic Compounds (DAHP synthase to chorismate)	2-keto-3-deoxy-D-arabino-heptulosonate-7-phosphate synthase II (EC 2.5.1.54)
Amino Acids and Derivatives	Aromatic amino acids and derivatives	Common Pathway For Synthesis of Aromatic Compounds (DAHP synthase to chorismate)	Shikimate 5-dehydrogenase I alpha (EC 1.1.1.25)
Amino Acids and Derivatives	Aromatic amino acids and derivatives	Common Pathway For Synthesis of Aromatic Compounds (DAHP synthase to chorismate)	3-dehydroquinate dehydratase II (EC 4.2.1.10)
Amino Acids and Derivatives	Aromatic amino acids and derivatives	Chorismate: Intermediate for synthesis of Tryptophan, PAPA antibiotics, PABA, 3-hydroxyanthranilate and more.	Tryptophan synthase beta chain (EC 4.2.1.20)
Amino Acids and Derivatives	Aromatic amino acids and derivatives	Chorismate: Intermediate for synthesis of Tryptophan, PAPA antibiotics, PABA, 3-hydroxyanthranilate and more.	Para-aminobenzoate synthase, aminase component (EC 2.6.1.85)
Amino Acids and Derivatives	Aromatic amino acids and derivatives	Chorismate: Intermediate for synthesis of Tryptophan, PAPA antibiotics, PABA, 3-hydroxyanthranilate and more.	Indole-3-glycerol phosphate synthase (EC 4.1.1.48)
Amino Acids and Derivatives	Aromatic amino acids and derivatives	Chorismate: Intermediate for synthesis of Tryptophan, PAPA antibiotics, PABA, 3-hydroxyanthranilate and more.	Anthranilate synthase, amidotransferase component (EC 4.1.3.27)
Amino Acids and Derivatives	Aromatic amino acids and derivatives	Chorismate: Intermediate for synthesis of Tryptophan, PAPA antibiotics, PABA, 3-hydroxyanthranilate and more.	Tryptophan synthase alpha chain (EC 4.2.1.20)
Amino Acids and Derivatives	Aromatic amino acids and derivatives	Chorismate: Intermediate for synthesis of Tryptophan, PAPA antibiotics, PABA, 3-hydroxyanthranilate and more.	Phosphoribosylanthranilate isomerase (EC 5.3.1.24)
Amino Acids and Derivatives	Aromatic amino acids and derivatives	Chorismate: Intermediate for synthesis of Tryptophan, PAPA antibiotics, PABA, 3-hydroxyanthranilate and more.	Anthranilate phosphoribosyltransferase (EC 2.4.2.18)
Amino Acids and Derivatives	Aromatic amino acids and derivatives	Chorismate: Intermediate for synthesis of Tryptophan, PAPA antibiotics, PABA, 3-hydroxyanthranilate and more.	Para-aminobenzoate synthase, amidotransferase component (EC 2.6.1.85)
Amino Acids and Derivatives	Aromatic amino acids and derivatives	Chorismate: Intermediate for synthesis of Tryptophan, PAPA antibiotics, PABA, 3-hydroxyanthranilate and more.	Phosphoribosylformimino-5-aminoimidazole carboxamide ribotide isomerase (EC 5.3.1.16)
Amino Acids and Derivatives	Aromatic amino acids and derivatives	Chorismate: Intermediate for synthesis of Tryptophan, PAPA antibiotics, PABA, 3-hydroxyanthranilate and more.	Aminodeoxychorismate lyase (EC 4.1.3.38)
Amino Acids and Derivatives	Aromatic amino acids and derivatives	Chorismate: Intermediate for synthesis of Tryptophan, PAPA antibiotics, PABA, 3-hydroxyanthranilate and more.	Anthranilate synthase, aminase component (EC 4.1.3.27)
Amino Acids and Derivatives	Aromatic amino acids and derivatives	Chorismate: Intermediate for synthesis of Tryptophan, PAPA antibiotics, PABA, 3-hydroxyanthranilate and more.	Isochorismatase (EC 3.3.2.1)
Amino Acids and Derivatives	Aromatic amino acids and derivatives	Tryptophan synthesis	Aminodeoxychorismate lyase (EC 4.1.3.38)

Amino Acids and Derivatives	Aromatic amino acids and derivatives	Tryptophan synthesis	Indole-3-glycerol phosphate synthase (EC 4.1.1.48)
Amino Acids and Derivatives	Aromatic amino acids and derivatives	Tryptophan synthesis	Tryptophan synthase alpha chain (EC 4.2.1.20)
Amino Acids and Derivatives	Aromatic amino acids and derivatives	Tryptophan synthesis	Anthranilate synthase, amidotransferase component (EC 4.1.3.27)
Amino Acids and Derivatives	Aromatic amino acids and derivatives	Tryptophan synthesis	Phosphoribosylanthranilate isomerase (EC 5.3.1.24)
Amino Acids and Derivatives	Aromatic amino acids and derivatives	Tryptophan synthesis	Anthranilate synthase, aminase component (EC 4.1.3.27)
Amino Acids and Derivatives	Aromatic amino acids and derivatives	Tryptophan synthesis	Anthranilate phosphoribosyltransferase (EC 2.4.2.18)
Amino Acids and Derivatives	Aromatic amino acids and derivatives	Tryptophan synthesis	Tryptophan synthase beta chain (EC 4.2.1.20)
Amino Acids and Derivatives	Aromatic amino acids and derivatives	Tryptophan synthesis	Para-aminobenzoate synthase, aminase component (EC 2.6.1.85)
Amino Acids and Derivatives	Aromatic amino acids and derivatives	Tryptophan synthesis	Para-aminobenzoate synthase, amidotransferase component (EC 2.6.1.85)
Amino Acids and Derivatives	Aromatic amino acids and derivatives	Aromatic amino acid degradation	2-hydroxyhepta-2,4-diene-1,7-dioate isomerase (EC 5.3.3.-)
Amino Acids and Derivatives	Aromatic amino acids and derivatives	Aromatic amino acid degradation	3,4-dihydroxyphenylacetate 2,3-dioxygenase (EC 1.13.11.15)
Amino Acids and Derivatives	Aromatic amino acids and derivatives	Aromatic amino acid degradation	Predicted 2-keto-4-pentenoate hydratase/2-oxohepta-3-ene-1,7-dioic acid hydratase
Amino Acids and Derivatives	Aromatic amino acids and derivatives	Aromatic amino acid degradation	5-carboxymethyl-2-oxo-hex-3- ene-1,7-dioate decarboxylase (EC 4.1.1.68)
Amino Acids and Derivatives	Aromatic amino acids and derivatives	Aromatic amino acid degradation	Kynurenine formamidase, bacterial (EC 3.5.1.9)
Amino Acids and Derivatives	Aromatic amino acids and derivatives	Aromatic amino acid degradation	Phenylalanine-4-hydroxylase (EC 1.14.16.1)
Amino Acids and Derivatives	Aromatic amino acids and derivatives	Aromatic amino acid degradation	Putative fumarylacetoacetate (FAA) hydrolase
Amino Acids and Derivatives	Aromatic amino acids and derivatives	Aromatic amino acid degradation	4-hydroxyphenylpyruvate dioxygenase (EC 1.13.11.27)
Amino Acids and Derivatives	Aromatic amino acids and derivatives	Aromatic amino acid degradation	2-oxo-hepta-3-ene-1,7-dioic acid hydratase (EC 4.2.-.-)
Amino Acids and Derivatives	Aromatic amino acids and derivatives	Phenylalanine and Tyrosine Branches from Chorismate	Prephenate and/or arogenate dehydrogenase (unknown specificity) (EC 1.3.1.12)(EC 1.3.1.43)
Amino Acids and Derivatives	Aromatic amino acids and derivatives	Phenylalanine and Tyrosine Branches from Chorismate	Biosynthetic Aromatic amino acid aminotransferase beta (EC 2.6.1.57)
Amino Acids and Derivatives	Aromatic amino acids and derivatives	Phenylalanine and Tyrosine Branches from Chorismate	Chorismate mutase I (EC 5.4.99.5)
Amino Acids and Derivatives	Aromatic amino acids and derivatives	Phenylalanine and Tyrosine Branches from Chorismate	Prephenate dehydratase (EC 4.2.1.51)
Amino Acids and Derivatives	Aromatic amino acids and derivatives	Indole-pyruvate oxidoreductase complex	Indolepyruvate oxidoreductase subunit IorB II (EC 1.2.7.8)
Amino Acids and Derivatives	Aromatic amino acids and derivatives	Indole-pyruvate oxidoreductase complex	Indolepyruvate oxidoreductase subunit IorA (EC 1.2.7.8)
Amino Acids and Derivatives	Aromatic amino acids and derivatives	Aromatic amino acid interconversions with aryl acids	Indolepyruvate ferredoxin oxidoreductase, alpha and beta subunits

Amino Acids and Derivatives	Aromatic amino acids and derivatives	Aromatic amino acid interconversions with aryl acids	Indolepyruvate oxidoreductase subunit IorA (EC 1.2.7.8)
Amino Acids and Derivatives	Aromatic amino acids and derivatives	Aromatic amino acid interconversions with aryl acids	Indolepyruvate oxidoreductase subunit IorB II (EC 1.2.7.8)
Amino Acids and Derivatives	Glutamine, glutamate, aspartate, asparagine; ammonia assimilation	Glutamate dehydrogenases	NADP-specific glutamate dehydrogenase (EC 1.4.1.4)
Amino Acids and Derivatives	Glutamine, glutamate, aspartate, asparagine; ammonia assimilation	Glutamine synthetases	Glutamine synthetase type I (EC 6.3.1.2)
Amino Acids and Derivatives	Glutamine, glutamate, aspartate, asparagine; ammonia assimilation	Glutamine synthetases	Glutamine synthetase type II, eukaryotic (EC 6.3.1.2)
Amino Acids and Derivatives	Glutamine, glutamate, aspartate, asparagine; ammonia assimilation	Glutamine, Glutamate, Aspartate and Asparagine Biosynthesis	NADP-specific glutamate dehydrogenase (EC 1.4.1.4)
Amino Acids and Derivatives	Glutamine, glutamate, aspartate, asparagine; ammonia assimilation	Glutamine, Glutamate, Aspartate and Asparagine Biosynthesis	Glutamine synthetase type I (EC 6.3.1.2)
Amino Acids and Derivatives	Glutamine, glutamate, aspartate, asparagine; ammonia assimilation	Glutamine, Glutamate, Aspartate and Asparagine Biosynthesis	Glutamine synthetase type II, eukaryotic (EC 6.3.1.2)
Amino Acids and Derivatives	Glutamine, glutamate, aspartate, asparagine; ammonia assimilation	Glutamine, Glutamate, Aspartate and Asparagine Biosynthesis	Aspartate ammonia-lyase (EC 4.3.1.1)
Amino Acids and Derivatives	Glutamine, glutamate, aspartate, asparagine; ammonia assimilation	Glutamine, Glutamate, Aspartate and Asparagine Biosynthesis	Ferredoxin-dependent glutamate synthase (EC 1.4.7.1)
Amino Acids and Derivatives	Glutamine, glutamate, aspartate, asparagine; ammonia assimilation	Glutamine, Glutamate, Aspartate and Asparagine Biosynthesis	Glutamate synthase [NADPH] large chain (EC 1.4.1.13)
Amino Acids and Derivatives	Glutamine, glutamate, aspartate, asparagine; ammonia assimilation	Glutamine, Glutamate, Aspartate and Asparagine Biosynthesis	Aspartate aminotransferase (EC 2.6.1.1)
Amino Acids and Derivatives	Glutamine, glutamate, aspartate, asparagine; ammonia assimilation	Glutamine, Glutamate, Aspartate and Asparagine Biosynthesis	Glutaminase (EC 3.5.1.2)
Amino Acids and Derivatives	Glutamine, glutamate, aspartate, asparagine; ammonia assimilation	Glutamine, Glutamate, Aspartate and Asparagine Biosynthesis	Glutamate synthase [NADPH] small chain (EC 1.4.1.13)
Amino Acids and Derivatives	Glutamine, glutamate, aspartate, asparagine; ammonia assimilation	Glutamine, Glutamate, Aspartate and Asparagine Biosynthesis	Asparagine synthetase [glutamine-hydrolyzing] (EC 6.3.5.4)
Metabolism of Aromatic Compounds	Metabolism of Aromatic Compounds - no subcategory	Aromatic Amin Catabolism	3,4-dihydroxyphenylacetate 2,3-dioxygenase (EC 1.13.11.15)
Metabolism of Aromatic Compounds	Metabolism of Aromatic Compounds - no subcategory	Aromatic Amin Catabolism	Nitrilotriacetate monooxygenase component B (EC 1.14.13.-)
Metabolism of Aromatic Compounds	Metabolism of Aromatic Compounds - no subcategory	Gentisate degradation	Putative n-hydroxybenzoate hydroxylase
Metabolism of Aromatic Compounds	Metabolism of Aromatic Compounds - no subcategory	Gentisate degradation	Maleylacetoacetate isomerase (EC 5.2.1.2)
Metabolism of Aromatic Compounds	Metabolism of Aromatic Compounds - no subcategory	Gentisate degradation	4-hydroxybenzoate transporter
Metabolism of Aromatic Compounds	Metabolism of Aromatic Compounds - no subcategory	Gentisate degradation	Fumarylacetoacetate hydrolase family protein
Metabolism of Aromatic Compounds	Metabolism of Aromatic Compounds - no subcategory	Gentisate degradation	Gentisate 1,2-dioxygenase (EC 1.13.11.4)
Metabolism of Aromatic Compounds	Metabolism of Aromatic Compounds - no subcategory	Gentisate degradation	putative 4-hydroxybenzoyl-CoA thioesterase
Metabolism of Aromatic Compounds	Metabolism of central aromatic intermediates	Catechol branch of beta-ketoadipate pathway	Succinyl-CoA:3-ketoacid-coenzyme A transferase subunit B (EC 2.8.3.5)
Metabolism of Aromatic Compounds	Metabolism of central aromatic intermediates	Catechol branch of beta-ketoadipate pathway	Succinyl-CoA:3-ketoacid-coenzyme A transferase subunit A (EC 2.8.3.5)

Metabolism of Aromatic Compounds	Metabolism of central aromatic intermediates	Catechol branch of beta-ketoadipate pathway	Catechol 1,2-dioxygenase (EC 1.13.11.1)
Metabolism of Aromatic Compounds	Metabolism of central aromatic intermediates	Catechol branch of beta-ketoadipate pathway	Catechol 1,2-dioxygenase 1 (EC 1.13.11.1)
Metabolism of Aromatic Compounds	Metabolism of central aromatic intermediates	Catechol branch of beta-ketoadipate pathway	Beta-ketoadipate enol-lactone hydrolase (EC 3.1.1.24)
Metabolism of Aromatic Compounds	Metabolism of central aromatic intermediates	Catechol branch of beta-ketoadipate pathway	3-oxoadipate CoA-transferase subunit B (EC 2.8.3.6)
Metabolism of Aromatic Compounds	Metabolism of central aromatic intermediates	Homogentisate pathway of aromatic compound degradation	Transcriptional regulator, IclR family
Metabolism of Aromatic Compounds	Metabolism of central aromatic intermediates	Homogentisate pathway of aromatic compound degradation	Homogentisate 1,2-dioxygenase (EC 1.13.11.5)
Metabolism of Aromatic Compounds	Metabolism of central aromatic intermediates	Homogentisate pathway of aromatic compound degradation	Fumarylacetoacetase (EC 3.7.1.2)
Metabolism of Aromatic Compounds	Metabolism of central aromatic intermediates	Homogentisate pathway of aromatic compound degradation	Maleylacetoacetate isomerase (EC 5.2.1.2)
Metabolism of Aromatic Compounds	Metabolism of central aromatic intermediates	Homogentisate pathway of aromatic compound degradation	4-hydroxyphenylpyruvate dioxygenase (EC 1.13.11.27)
Metabolism of Aromatic Compounds	Metabolism of central aromatic intermediates	Salicylate and gentisate catabolism	Putative n-hydroxybenzoate hydroxylase
Metabolism of Aromatic Compounds	Metabolism of central aromatic intermediates	Salicylate and gentisate catabolism	Fumarylacetoacetase (EC 3.7.1.2)
Metabolism of Aromatic Compounds	Metabolism of central aromatic intermediates	Salicylate and gentisate catabolism	Maleylacetoacetate isomerase (EC 5.2.1.2)
Metabolism of Aromatic Compounds	Metabolism of central aromatic intermediates	Salicylate and gentisate catabolism	salicylate esterase
Metabolism of Aromatic Compounds	Metabolism of central aromatic intermediates	Salicylate and gentisate catabolism	Gentisate 1,2-dioxygenase (EC 1.13.11.4)
Metabolism of Aromatic Compounds	Metabolism of central aromatic intermediates	Salicylate and gentisate catabolism	Salicylate hydroxylase (EC 1.14.13.1)
Metabolism of Aromatic Compounds	Metabolism of central aromatic intermediates	Salicylate and gentisate catabolism	4-hydroxybenzoate transporter
Metabolism of Aromatic Compounds	Metabolism of central aromatic intermediates	Salicylate and gentisate catabolism	Fumarylacetoacetate hydrolase family protein
Metabolism of Aromatic Compounds	Peripheral pathways for catabolism of aromatic compounds	Salicylate ester degradation	Salicylate hydroxylase (EC 1.14.13.1)
Metabolism of Aromatic Compounds	Peripheral pathways for catabolism of aromatic compounds	Salicylate ester degradation	salicylate esterase
Metabolism of Aromatic Compounds	Peripheral pathways for catabolism of aromatic compounds	Quinate degradation	3-dehydroquinate dehydratase II (EC 4.2.1.10)
Metabolism of Aromatic Compounds	Peripheral pathways for catabolism of aromatic compounds	p-Hydroxybenzoate degradation	P-hydroxybenzoate hydroxylase (EC 1.14.13.2)
Metabolism of Aromatic Compounds	Peripheral pathways for catabolism of aromatic compounds	p-Hydroxybenzoate degradation	4-hydroxybenzoate transporter
Metabolism of Aromatic Compounds	Peripheral pathways for catabolism of aromatic compounds	Benzoate degradation	Benzoate 1,2-dioxygenase alpha subunit (EC 1.14.12.10)
Metabolism of Aromatic Compounds	Peripheral pathways for catabolism of aromatic compounds	Benzoate degradation	Benzoylformate decarboxylase (EC 4.1.1.7)
Metabolism of Aromatic Compounds	Peripheral pathways for catabolism of aromatic compounds	Benzoate degradation	Ortho-halobenzoate 1,2-dioxygenase beta-ISP protein OhbA

Metabolism of Aromatic Compounds	Peripheral pathways for catabolism of aromatic compounds	Benzoate degradation	Ortho-halobenzoate 1,2-dioxygenase alpha-ISP protein OhbB
Resistance to antibiotics and toxic compounds	Resistance to antibiotics	Beta-lactamase	Metal-dependent hydrolases of the beta-lactamase superfamily I
Resistance to antibiotics and toxic compounds	Resistance to antibiotics	Resistance to fluoroquinolones	DNA gyrase subunit A (EC 5.99.1.3)
Resistance to antibiotics and toxic compounds	Resistance to antibiotics	Resistance to fluoroquinolones	Efflux pump Lde
Resistance to antibiotics and toxic compounds	Resistance to antibiotics	Resistance to fluoroquinolones	DNA gyrase subunit B (EC 5.99.1.3)
Resistance to antibiotics and toxic compounds	Resistance to antibiotics	Tolerance to colicin E2	Conserved uncharacterized protein CreA
Resistance to antibiotics and toxic compounds	Resistance to heavy metals	Copper homeostasis	Copper tolerance protein
Resistance to antibiotics and toxic compounds	Resistance to heavy metals	Copper homeostasis	Cytochrome c heme lyase subunit CcmF
Resistance to antibiotics and toxic compounds	Resistance to heavy metals	Copper homeostasis	Multidrug resistance transporter, Bcr/CflA family
Resistance to antibiotics and toxic compounds	Resistance to heavy metals	Copper homeostasis	Multicopper oxidase
Resistance to antibiotics and toxic compounds	Resistance to heavy metals	Copper homeostasis	Copper-translocating P-type ATPase (EC 3.6.3.4)
Resistance to antibiotics and toxic compounds	Resistance to heavy metals	Copper homeostasis	Cytochrome c heme lyase subunit CcmH
Resistance to antibiotics and toxic compounds	Resistance to heavy metals	Copper homeostasis	CopG protein
Resistance to antibiotics and toxic compounds	Resistance to heavy metals	Copper homeostasis: copper tolerance	Copper homeostasis protein CutE
Resistance to antibiotics and toxic compounds	Resistance to heavy metals	Arsenic resistance	Arsenical-resistance protein ACR3
Resistance to antibiotics and toxic compounds	Resistance to heavy metals	Arsenic resistance	Arsenic resistance protein ArsH
Resistance to antibiotics and toxic compounds	Resistance to heavy metals	Arsenic resistance	Arsenical resistance operon repressor
Resistance to antibiotics and toxic compounds	Resistance to heavy metals	Arsenic resistance	Arsenate reductase (EC 1.20.4.1)
Resistance to antibiotics and toxic compounds	Resistance to heavy metals	Mercuric reductase	PF00070 family, FAD-dependent NAD(P)-disulphide oxidoreductase
Resistance to antibiotics and toxic compounds	Resistance to heavy metals	Mercuric reductase	Mercuric ion reductase (EC 1.16.1.1)
Resistance to antibiotics and toxic compounds	Resistance to heavy metals	Resistance to chromium compounds	Chromate transport protein ChrA
Resistance to antibiotics and toxic compounds	Resistance to heavy metals	Mercury resistance operon	Mercuric transport protein, MerT
Resistance to antibiotics and toxic compounds	Resistance to heavy metals	Mercury resistance operon	Mercuric ion reductase (EC 1.16.1.1)
Resistance to antibiotics and toxic compounds	Resistance to heavy metals	Cobalt-zinc-cadmium resistance	Transcriptional regulator, MerR family
Resistance to antibiotics and toxic compounds	Resistance to heavy metals	Cobalt-zinc-cadmium resistance	Cobalt-zinc-cadmium resistance protein

Resistance to antibiotics and toxic compounds	Resistance to heavy metals	Cobalt-zinc-cadmium resistance	Probable Co/Zn/Cd efflux system membrane fusion protein
Resistance to antibiotics and toxic compounds	Resistance to heavy metals	Cobalt-zinc-cadmium resistance	Hypothetical protein involved in heavy metal export

The annotated genes from the genome of strain H3M7-6^T involving in metabolizaton of carbohydrates, amino acids, and aromatic compounds and resistance to antibiotics and heavy metals.

Category	Subcategory	Subsystem	Annotated genes and its roles
Carbohydrates	Central carbohydrate metabolism	Glyoxylate bypass	Citrate synthase (si) (EC 2.3.3.1)
Carbohydrates	Central carbohydrate metabolism	Glyoxylate bypass	Malate dehydrogenase (EC 1.1.1.37)
Carbohydrates	Central carbohydrate metabolism	Glyoxylate bypass	Isocitrate lyase (EC 4.1.3.1)
Carbohydrates	Central carbohydrate metabolism	Glyoxylate bypass	Aconitate hydratase 2 (EC 4.2.1.3)
Carbohydrates	Central carbohydrate metabolism	Glyoxylate bypass	Malate synthase G (EC 2.3.3.9)
Carbohydrates	Central carbohydrate metabolism	Glyoxylate bypass	Aconitate hydratase (EC 4.2.1.3)
Carbohydrates	Central carbohydrate metabolism	Glyoxylate bypass	Isocitrate dehydrogenase phosphatase (EC 2.7.11.5)/kinase (EC 3.1.3.-)
Carbohydrates	Central carbohydrate metabolism	Pyruvate metabolism I: anaplerotic reactions, PEP	Pyruvate,phosphate dikinase (EC 2.7.9.1)
Carbohydrates	Central carbohydrate metabolism	Pyruvate metabolism I: anaplerotic reactions, PEP	Pyruvate kinase (EC 2.7.1.40)
Carbohydrates	Central carbohydrate metabolism	Pyruvate metabolism I: anaplerotic reactions, PEP	Phosphoenolpyruvate carboxylase (EC 4.1.1.31)
Carbohydrates	Central carbohydrate metabolism	Pyruvate metabolism I: anaplerotic reactions, PEP	NADP-dependent malic enzyme (EC 1.1.1.40)
Carbohydrates	Central carbohydrate metabolism	Pyruvate metabolism I: anaplerotic reactions, PEP	Phosphoenolpyruvate synthase (EC 2.7.9.2)
Carbohydrates	Central carbohydrate metabolism	Pyruvate metabolism I: anaplerotic reactions, PEP	Phosphoenolpyruvate carboxykinase [GTP] (EC 4.1.1.32)
Carbohydrates	Central carbohydrate metabolism	Pyruvate Alanine Serine Interconversions	Valine--pyruvate aminotransferase (EC 2.6.1.66)
Carbohydrates	Central carbohydrate metabolism	Pyruvate Alanine Serine Interconversions	Alanine racemase (EC 5.1.1.1)
Carbohydrates	Central carbohydrate metabolism	Pyruvate Alanine Serine Interconversions	Branched-chain amino acid aminotransferase (EC 2.6.1.42)
Carbohydrates	Central carbohydrate metabolism	Pyruvate Alanine Serine Interconversions	Omega-amino acid--pyruvate aminotransferase (EC 2.6.1.18)
Carbohydrates	Central carbohydrate metabolism	TCA Cycle	Succinyl-CoA ligase [ADP-forming] alpha chain (EC 6.2.1.5)
Carbohydrates	Central carbohydrate metabolism	TCA Cycle	Malate dehydrogenase (EC 1.1.1.37)
Carbohydrates	Central carbohydrate metabolism	TCA Cycle	Succinyl-CoA ligase [ADP-forming] beta chain (EC 6.2.1.5)
Carbohydrates	Central carbohydrate metabolism	TCA Cycle	Fumarate hydratase class I, aerobic (EC 4.2.1.2)
Carbohydrates	Central carbohydrate metabolism	TCA Cycle	Dihydrolipoamide dehydrogenase of pyruvate dehydrogenase complex (EC 1.8.1.4)
Carbohydrates	Central carbohydrate metabolism	TCA Cycle	Dihydrolipoamide dehydrogenase of 2-oxoglutarate dehydrogenase (EC 1.8.1.4)
Carbohydrates	Central carbohydrate metabolism	TCA Cycle	Dihydrolipoamide succinyltransferase component (E2) of 2-oxoglutarate dehydrogenase complex (EC 2.3.1.61)
Carbohydrates	Central carbohydrate metabolism	TCA Cycle	Aconitate hydratase (EC 4.2.1.3)
Carbohydrates	Central carbohydrate metabolism	TCA Cycle	Citrate synthase (si) (EC 2.3.3.1)
Carbohydrates	Central carbohydrate metabolism	TCA Cycle	Isocitrate dehydrogenase [NADP] (EC 1.1.1.42)
Carbohydrates	Central carbohydrate metabolism	TCA Cycle	Aconitate hydratase 2 (EC 4.2.1.3)
Carbohydrates	Central carbohydrate metabolism	TCA Cycle	2-oxoglutarate dehydrogenase E1 component (EC 1.2.4.2)
Carbohydrates	Central carbohydrate metabolism	Pyruvate metabolism II: acetyl-CoA, acetogenesis from pyruvate	Phosphate acetyltransferase (EC 2.3.1.8)
Carbohydrates	Central carbohydrate metabolism	Pyruvate metabolism II: acetyl-CoA, acetogenesis from pyruvate	Acetyl-CoA synthetase (ADP-forming) alpha and beta chains, putative
Carbohydrates	Central carbohydrate metabolism	Pyruvate metabolism II: acetyl-CoA, acetogenesis from pyruvate	NAD-dependent protein deacetylase of SIR2 family
Carbohydrates	Central carbohydrate metabolism	Pyruvate metabolism II: acetyl-CoA, acetogenesis from pyruvate	Dihydrolipoamide dehydrogenase of pyruvate dehydrogenase complex (EC 1.8.1.4)
Carbohydrates	Central carbohydrate metabolism	Pyruvate metabolism II: acetyl-CoA, acetogenesis from pyruvate	Pyruvate dehydrogenase E1 component (EC 1.2.4.1)
Carbohydrates	Central carbohydrate metabolism	Pyruvate metabolism II: acetyl-CoA, acetogenesis from pyruvate	Acetate kinase (EC 2.7.2.1)
Carbohydrates	Central carbohydrate metabolism	Pyruvate metabolism II: acetyl-CoA, acetogenesis from pyruvate	Aldehyde dehydrogenase (EC 1.2.1.3)
Carbohydrates	Central carbohydrate metabolism	Pyruvate metabolism II: acetyl-CoA, acetogenesis from pyruvate	Acetate permease ActP (cation/acetate symporter)
Carbohydrates	Central carbohydrate metabolism	Pyruvate metabolism II: acetyl-CoA, acetogenesis from pyruvate	Dihydrolipoamide acetyltransferase component of pyruvate dehydrogenase complex (EC 2.3.1.12)
Carbohydrates	Central carbohydrate metabolism	Glycolysis and Gluconeogenesis	Pyruvate,phosphate dikinase (EC 2.7.9.1)
Carbohydrates	Central carbohydrate metabolism	Glycolysis and Gluconeogenesis	Pyruvate kinase (EC 2.7.1.40)

Carbohydrates	Central carbohydrate metabolism	Glycolysis and Gluconeogenesis	Phosphoglycerate kinase (EC 2.7.2.3)
Carbohydrates	Central carbohydrate metabolism	Glycolysis and Gluconeogenesis	Enolase (EC 4.2.1.11)
Carbohydrates	Central carbohydrate metabolism	Glycolysis and Gluconeogenesis	Glucokinase (EC 2.7.1.2)
Carbohydrates	Central carbohydrate metabolism	Glycolysis and Gluconeogenesis	Fructose-1,6-bisphosphatase, type I (EC 3.1.3.11)
Carbohydrates	Central carbohydrate metabolism	Glycolysis and Gluconeogenesis	Fructose-bisphosphate aldolase class II (EC 4.1.2.13)
Carbohydrates	Central carbohydrate metabolism	Glycolysis and Gluconeogenesis	Phosphoenolpyruvate synthase (EC 2.7.9.2)
Carbohydrates	Central carbohydrate metabolism	Glycolysis and Gluconeogenesis	Triosephosphate isomerase (EC 5.3.1.1)
Carbohydrates	Central carbohydrate metabolism	Glycolysis and Gluconeogenesis	NAD-dependent glyceraldehyde-3-phosphate dehydrogenase (EC 1.2.1.12)
Carbohydrates	Central carbohydrate metabolism	Glycolysis and Gluconeogenesis	Glucose-6-phosphate isomerase (EC 5.3.1.9)
Carbohydrates	Central carbohydrate metabolism	Methylglyoxal Metabolism	Aldehyde dehydrogenase (EC 1.2.1.3)
Carbohydrates	Central carbohydrate metabolism	Methylglyoxal Metabolism	Hydroxyacylglutathione hydrolase (EC 3.1.2.6)
Carbohydrates	Central carbohydrate metabolism	Methylglyoxal Metabolism	Lactoylglutathione lyase (EC 4.4.1.5)
Carbohydrates	Central carbohydrate metabolism	Glycolate, glyoxylate interconversions	D-Lactate dehydrogenase, cytochrome c-dependent (EC 1.1.2.4)
Carbohydrates	Central carbohydrate metabolism	Glycolate, glyoxylate interconversions	Glycolate dehydrogenase (EC 1.1.99.14), subunit GlcD
Carbohydrates	Central carbohydrate metabolism	Glycolate, glyoxylate interconversions	Glyoxylate reductase (EC 1.1.1.79)
Carbohydrates	Central carbohydrate metabolism	Glycolate, glyoxylate interconversions	Glycolate dehydrogenase (EC 1.1.99.14), iron-sulfur subunit GlcF
Carbohydrates	Central carbohydrate metabolism	Glycolate, glyoxylate interconversions	Phosphoglycolate phosphatase (EC 3.1.3.18)
Carbohydrates	Central carbohydrate metabolism	Glycolate, glyoxylate interconversions	Hydroxypyruvate reductase (EC 1.1.1.81)
Carbohydrates	Central carbohydrate metabolism	Glycolate, glyoxylate interconversions	Glycolate dehydrogenase (EC 1.1.99.14), FAD-binding subunit GlcE
Carbohydrates	Central carbohydrate metabolism	Glycolate, glyoxylate interconversions	Glyoxylate reductase (EC 1.1.1.26)
Carbohydrates	Central carbohydrate metabolism	Dehydrogenase complexes	2-oxoglutarate dehydrogenase E1 component (EC 1.2.4.2)
Carbohydrates	Central carbohydrate metabolism	Dehydrogenase complexes	Cytosol aminopeptidase PepA (EC 3.4.11.1)
Carbohydrates	Central carbohydrate metabolism	Dehydrogenase complexes	Dihydrolipoamide dehydrogenase of 2-oxoglutarate dehydrogenase (EC 1.8.1.4)
Carbohydrates	Central carbohydrate metabolism	Dehydrogenase complexes	Dihydrolipoamide succinyltransferase component (E2) of 2-oxoglutarate dehydrogenase complex (EC 2.3.1.61)
Carbohydrates	Central carbohydrate metabolism	Dehydrogenase complexes	Pyruvate dehydrogenase E1 component (EC 1.2.4.1)
Carbohydrates	Central carbohydrate metabolism	Dehydrogenase complexes	Dihydrolipoamide acetyltransferase component of pyruvate dehydrogenase complex (EC 2.3.1.12)
Carbohydrates	Central carbohydrate metabolism	Dehydrogenase complexes	Leucine-, isoleucine-, valine-, threonine-, and alanine-binding protein
Carbohydrates	Monosaccharides	Mannose Metabolism	Phosphomannomutase (EC 5.4.2.8)
Carbohydrates	Monosaccharides	Mannose Metabolism	Mannose-6-phosphate isomerase (EC 5.3.1.8)
Carbohydrates	Di- and oligosaccharides	Maltose and Maltodextrin Utilization	4-alpha-glucanotransferase (amylomaltase) (EC 2.4.1.25)
Carbohydrates	Di- and oligosaccharides	Maltose and Maltodextrin Utilization	Glycogen phosphorylase (EC 2.4.1.1)
Carbohydrates	Polysaccharides	Glycogen metabolism	Glycogen synthase, ADP-glucose transglucosylase (EC 2.4.1.21)
Carbohydrates	Polysaccharides	Glycogen metabolism	Glycogen phosphorylase (EC 2.4.1.1)
Carbohydrates	Polysaccharides	Glycogen metabolism	4-alpha-glucanotransferase (amylomaltase) (EC 2.4.1.25)
Carbohydrates	Polysaccharides	Glycogen metabolism	1,4-alpha-glucan (glycogen) branching enzyme, GH-13-type (EC 2.4.1.18)
Carbohydrates	Polysaccharides	Glycogen metabolism	Glucose-1-phosphate adenylyltransferase (EC 2.7.7.27)
Carbohydrates	Organic acids	Propionyl-CoA to Succinyl-CoA Module	Propionyl-CoA carboxylase biotin-containing subunit (EC 6.4.1.3)
Carbohydrates	Organic acids	Propionyl-CoA to Succinyl-CoA Module	Methylmalonyl-CoA epimerase (EC 5.1.99.1)
Carbohydrates	Organic acids	Propionyl-CoA to Succinyl-CoA Module	B12 binding domain of Methylmalonyl-CoA mutase (EC 5.4.99.2)
Carbohydrates	Organic acids	Propionyl-CoA to Succinyl-CoA Module	Methylmalonyl-CoA mutase (EC 5.4.99.2)
Carbohydrates	Organic acids	Propionyl-CoA to Succinyl-CoA Module	Propionyl-CoA carboxylase carboxyl transferase subunit (EC 6.4.1.3)
Carbohydrates	Organic acids	Glycerate metabolism	Hydroxypyruvate reductase (EC 1.1.1.81)
Carbohydrates	Organic acids	Glycerate metabolism	2-hydroxy-3-oxopropionate reductase (EC 1.1.1.60)
Carbohydrates	Organic acids	Glycerate metabolism	Hydroxypyruvate isomerase (EC 5.3.1.22)
Carbohydrates	Organic acids	Glycerate metabolism	Pyruvate kinase (EC 2.7.1.40)
Carbohydrates	Organic acids	Glycerate metabolism	Glycerate kinase (EC 2.7.1.31)
Amino Acids and Derivatives	Lysine, threonine, methionine, and cysteine	Methionine Degradation	Pyruvate dehydrogenase E1 component (EC 1.2.4.1)
Amino Acids and Derivatives	Lysine, threonine, methionine, and cysteine	Methionine Degradation	S-adenosylmethionine synthetase (EC 2.5.1.6)
Amino Acids and Derivatives	Lysine, threonine, methionine, and cysteine	Methionine Degradation	Adenosylhomocysteinase (EC 3.3.1.1)

Amino Acids and Derivatives	Lysine, threonine, methionine, and cysteine	Methionine Degradation	S-adenosylhomocysteine nucleosidase (EC 3.2.2.9)
Amino Acids and Derivatives	Lysine, threonine, methionine, and cysteine	Lysine degradation	Lysine decarboxylase (EC 4.1.1.18)
Amino Acids and Derivatives	Lysine, threonine, methionine, and cysteine	Lysine degradation	L-lysine permease
Amino Acids and Derivatives	Lysine, threonine, methionine, and cysteine	Cysteine Biosynthesis	Sulfate adenylyltransferase subunit 2 (EC 2.7.7.4)
Amino Acids and Derivatives	Lysine, threonine, methionine, and cysteine	Cysteine Biosynthesis	Sulfite reductase [NADPH] hemoprotein beta-component (EC 1.8.1.2)
Amino Acids and Derivatives	Lysine, threonine, methionine, and cysteine	Cysteine Biosynthesis	Cys regulon transcriptional activator CysB
Amino Acids and Derivatives	Lysine, threonine, methionine, and cysteine	Cysteine Biosynthesis	Serine acetyltransferase (EC 2.3.1.30)
Amino Acids and Derivatives	Lysine, threonine, methionine, and cysteine	Cysteine Biosynthesis	Cysteine synthase (EC 2.5.1.47)
Amino Acids and Derivatives	Lysine, threonine, methionine, and cysteine	Cysteine Biosynthesis	Sulfate adenylyltransferase subunit 1 (EC 2.7.7.4)
Amino Acids and Derivatives	Lysine, threonine, methionine, and cysteine	Cysteine Biosynthesis	Sulfate and thiosulfate import ATP-binding protein CysA (EC 3.6.3.25)
Amino Acids and Derivatives	Lysine, threonine, methionine, and cysteine	Cysteine Biosynthesis	Sulfate transport system permease protein CysT
Amino Acids and Derivatives	Lysine, threonine, methionine, and cysteine	Cysteine Biosynthesis	Sulfate transport system permease protein CysW
Amino Acids and Derivatives	Lysine, threonine, methionine, and cysteine	Cysteine Biosynthesis	Cysteine synthase B (EC 2.5.1.47)
Amino Acids and Derivatives	Lysine, threonine, methionine, and cysteine	Cysteine Biosynthesis	Sulfate and thiosulfate binding protein CysP
Amino Acids and Derivatives	Lysine, threonine, methionine, and cysteine	Lysine Biosynthesis DAP Pathway, GJO scratch	Aspartokinase (EC 2.7.2.4)
Amino Acids and Derivatives	Lysine, threonine, methionine, and cysteine	Lysine Biosynthesis DAP Pathway, GJO scratch	4-hydroxy-tetrahydrodipicolinate reductase (EC 1.17.1.8)
Amino Acids and Derivatives	Lysine, threonine, methionine, and cysteine	Lysine Biosynthesis DAP Pathway, GJO scratch	Diaminopimelate decarboxylase (EC 4.1.1.20)
Amino Acids and Derivatives	Lysine, threonine, methionine, and cysteine	Lysine Biosynthesis DAP Pathway, GJO scratch	Diaminopimelate epimerase (EC 5.1.1.7)
Amino Acids and Derivatives	Lysine, threonine, methionine, and cysteine	Lysine Biosynthesis DAP Pathway, GJO scratch	4-hydroxy-tetrahydrodipicolinate synthase (EC 4.3.3.7)
Amino Acids and Derivatives	Lysine, threonine, methionine, and cysteine	Lysine Biosynthesis DAP Pathway, GJO scratch	N-succinyl-L,L-diaminopimelate desuccinylase (EC 3.5.1.18)
Amino Acids and Derivatives	Lysine, threonine, methionine, and cysteine	Lysine Biosynthesis DAP Pathway, GJO scratch	Aspartate-semialdehyde dehydrogenase (EC 1.2.1.11)
Amino Acids and Derivatives	Lysine, threonine, methionine, and cysteine	Lysine Biosynthesis DAP Pathway, GJO scratch	2,3,4,5-tetrahydropyridine-2,6-dicarboxylate N-succinyltransferase (EC 2.3.1.117)
Amino Acids and Derivatives	Lysine, threonine, methionine, and cysteine	Methionine Biosynthesis	Homoserine dehydrogenase (EC 1.1.1.3)
Amino Acids and Derivatives	Lysine, threonine, methionine, and cysteine	Methionine Biosynthesis	Adenosylhomocysteinase (EC 3.3.1.1)
Amino Acids and Derivatives	Lysine, threonine, methionine, and cysteine	Methionine Biosynthesis	S-adenosylhomocysteine nucleosidase (EC 3.2.2.9)

Amino Acids and Derivatives	Lysine, threonine, methionine, and cysteine	Methionine Biosynthesis	5,10-methylenetetrahydrofolate reductase (EC 1.5.1.20)
Amino Acids and Derivatives	Lysine, threonine, methionine, and cysteine	Methionine Biosynthesis	O-acetylhomoserine sulfhydrylase (EC 2.5.1.49)
Amino Acids and Derivatives	Lysine, threonine, methionine, and cysteine	Methionine Biosynthesis	Serine acetyltransferase (EC 2.3.1.30)
Amino Acids and Derivatives	Lysine, threonine, methionine, and cysteine	Methionine Biosynthesis	S-adenosylmethionine synthetase (EC 2.5.1.6)
Amino Acids and Derivatives	Lysine, threonine, methionine, and cysteine	Methionine Biosynthesis	5-methyltetrahydrofolate--homocysteine methyltransferase (EC 2.1.1.13)
Amino Acids and Derivatives	Lysine, threonine, methionine, and cysteine	Methionine Biosynthesis	Homoserine O-acetyltransferase (EC 2.3.1.31)
Amino Acids and Derivatives	Lysine, threonine, methionine, and cysteine	Methionine Biosynthesis	Homoserine kinase (EC 2.7.1.39)
Amino Acids and Derivatives	Lysine, threonine, methionine, and cysteine	Methionine Biosynthesis	O-succinylhomoserine sulfhydrylase (EC 2.5.1.48)
Amino Acids and Derivatives	Lysine, threonine, methionine, and cysteine	Methionine Biosynthesis	Cysteine synthase (EC 2.5.1.47)
Amino Acids and Derivatives	Lysine, threonine, methionine, and cysteine	Methionine Biosynthesis	Cystathionine beta-lyase (EC 4.4.1.8)
Amino Acids and Derivatives	Lysine, threonine, methionine, and cysteine	Lysine Biosynthesis DAP Pathway	4-hydroxy-tetrahydrodipicolinate synthase (EC 4.3.3.7)
Amino Acids and Derivatives	Lysine, threonine, methionine, and cysteine	Lysine Biosynthesis DAP Pathway	N-succinyl-L,L-diaminopimelate desuccinylase (EC 3.5.1.18)
Amino Acids and Derivatives	Lysine, threonine, methionine, and cysteine	Lysine Biosynthesis DAP Pathway	Aspartate-semialdehyde dehydrogenase (EC 1.2.1.11)
Amino Acids and Derivatives	Lysine, threonine, methionine, and cysteine	Lysine Biosynthesis DAP Pathway	2,3,4,5-tetrahydropyridine-2,6-dicarboxylate N-succinyltransferase (EC 2.3.1.117)
Amino Acids and Derivatives	Lysine, threonine, methionine, and cysteine	Lysine Biosynthesis DAP Pathway	Aspartokinase (EC 2.7.2.4)
Amino Acids and Derivatives	Lysine, threonine, methionine, and cysteine	Lysine Biosynthesis DAP Pathway	4-hydroxy-tetrahydrodipicolinate reductase (EC 1.17.1.8)
Amino Acids and Derivatives	Lysine, threonine, methionine, and cysteine	Lysine Biosynthesis DAP Pathway	Diaminopimelate decarboxylase (EC 4.1.1.20)
Amino Acids and Derivatives	Lysine, threonine, methionine, and cysteine	Lysine Biosynthesis DAP Pathway	Diaminopimelate epimerase (EC 5.1.1.7)
Amino Acids and Derivatives	Lysine, threonine, methionine, and cysteine	Threonine and Homoserine Biosynthesis	Homoserine kinase (EC 2.7.1.39)
Amino Acids and Derivatives	Lysine, threonine, methionine, and cysteine	Threonine and Homoserine Biosynthesis	Aspartate-semialdehyde dehydrogenase (EC 1.2.1.11)
Amino Acids and Derivatives	Lysine, threonine, methionine, and cysteine	Threonine and Homoserine Biosynthesis	Threonine synthase (EC 4.2.3.1)
Amino Acids and Derivatives	Lysine, threonine, methionine, and cysteine	Threonine and Homoserine Biosynthesis	Aspartate aminotransferase (EC 2.6.1.1)
Amino Acids and Derivatives	Lysine, threonine, methionine, and cysteine	Threonine and Homoserine Biosynthesis	Homoserine dehydrogenase (EC 1.1.1.3)
Amino Acids and Derivatives	Lysine, threonine, methionine, and cysteine	Threonine and Homoserine Biosynthesis	Aspartokinase (EC 2.7.2.4)
Amino Acids and Derivatives	Amino Acids and Derivatives - no subcategory	Creatine and Creatinine Degradation	Creatinine amidohydrolase (EC 3.5.2.10)

Amino Acids and Derivatives	Amino Acids and Derivatives - no subcategory	Creatine and Creatinine Degradation	Cytosine deaminase (EC 3.5.4.1)
Amino Acids and Derivatives	Branched-chain amino acids	HMG CoA Synthesis	Acetoacetyl-CoA synthetase [leucine] (EC 6.2.1.16)
Amino Acids and Derivatives	Branched-chain amino acids	HMG CoA Synthesis	Methylcrotonyl-CoA carboxylase carboxyl transferase subunit (EC 6.4.1.4)
Amino Acids and Derivatives	Branched-chain amino acids	HMG CoA Synthesis	Isovaleryl-CoA dehydrogenase (EC 1.3.8.4)
Amino Acids and Derivatives	Branched-chain amino acids	HMG CoA Synthesis	Hydroxymethylglutaryl-CoA lyase (EC 4.1.3.4)
Amino Acids and Derivatives	Branched-chain amino acids	HMG CoA Synthesis	Methylcrotonyl-CoA carboxylase biotin-containing subunit (EC 6.4.1.4)
Amino Acids and Derivatives	Branched-chain amino acids	HMG CoA Synthesis	Acetoacetyl-CoA synthetase (EC 6.2.1.16)
Amino Acids and Derivatives	Branched-chain amino acids	HMG CoA Synthesis	Methylglutaconyl-CoA hydratase (EC 4.2.1.18)
Amino Acids and Derivatives	Branched-chain amino acids	Leucine Degradation and HMG-CoA Metabolism	Acetoacetyl-CoA synthetase [leucine] (EC 6.2.1.16)
Amino Acids and Derivatives	Branched-chain amino acids	Leucine Degradation and HMG-CoA Metabolism	Succinyl-CoA:3-ketoacid-coenzyme A transferase subunit A (EC 2.8.3.5)
Amino Acids and Derivatives	Branched-chain amino acids	Leucine Degradation and HMG-CoA Metabolism	Methylcrotonyl-CoA carboxylase carboxyl transferase subunit (EC 6.4.1.4)
Amino Acids and Derivatives	Branched-chain amino acids	Leucine Degradation and HMG-CoA Metabolism	Isovaleryl-CoA dehydrogenase (EC 1.3.8.4)
Amino Acids and Derivatives	Branched-chain amino acids	Leucine Degradation and HMG-CoA Metabolism	Branched-chain amino acid aminotransferase (EC 2.6.1.42)
Amino Acids and Derivatives	Branched-chain amino acids	Leucine Degradation and HMG-CoA Metabolism	Hydroxymethylglutaryl-CoA lyase (EC 4.1.3.4)
Amino Acids and Derivatives	Branched-chain amino acids	Leucine Degradation and HMG-CoA Metabolism	Methylcrotonyl-CoA carboxylase biotin-containing subunit (EC 6.4.1.4)
Amino Acids and Derivatives	Branched-chain amino acids	Leucine Degradation and HMG-CoA Metabolism	Succinyl-CoA:3-ketoacid-coenzyme A transferase subunit B (EC 2.8.3.5)
Amino Acids and Derivatives	Branched-chain amino acids	Leucine Degradation and HMG-CoA Metabolism	Methylglutaconyl-CoA hydratase (EC 4.2.1.18)
Amino Acids and Derivatives	Branched-chain amino acids	Leucine Degradation and HMG-CoA Metabolism	Branched-chain acyl-CoA dehydrogenase (EC 1.3.99.12)
Amino Acids and Derivatives	Branched-chain amino acids	Leucine Degradation and HMG-CoA Metabolism	Acetoacetyl-CoA synthetase (EC 6.2.1.16)
Amino Acids and Derivatives	Branched-chain amino acids	Branched-Chain Amino Acid Biosynthesis	Acetolactate synthase small subunit (EC 2.2.1.6)
Amino Acids and Derivatives	Branched-chain amino acids	Branched-Chain Amino Acid Biosynthesis	3-isopropylmalate dehydrogenase (EC 1.1.1.85)
Amino Acids and Derivatives	Branched-chain amino acids	Branched-Chain Amino Acid Biosynthesis	3-isopropylmalate dehydratase large subunit (EC 4.2.1.33)
Amino Acids and Derivatives	Branched-chain amino acids	Branched-Chain Amino Acid Biosynthesis	3-isopropylmalate dehydratase small subunit (EC 4.2.1.33)
Amino Acids and Derivatives	Branched-chain amino acids	Branched-Chain Amino Acid Biosynthesis	Dihydroxy-acid dehydratase (EC 4.2.1.9)
Amino Acids and Derivatives	Branched-chain amino acids	Branched-Chain Amino Acid Biosynthesis	Branched-chain amino acid aminotransferase (EC 2.6.1.42)

Amino Acids and Derivatives	Branched-chain amino acids	Branched-Chain Amino Acid Biosynthesis	Acetolactate synthase large subunit (EC 2.2.1.6)
Amino Acids and Derivatives	Branched-chain amino acids	Branched-Chain Amino Acid Biosynthesis	Leucine-responsive regulatory protein, regulator for leucine (or lrp) regulon and high-affinity branched-chain amino acid transport system
Amino Acids and Derivatives	Branched-chain amino acids	Branched-Chain Amino Acid Biosynthesis	Valine--pyruvate aminotransferase (EC 2.6.1.66)
Amino Acids and Derivatives	Branched-chain amino acids	Branched-Chain Amino Acid Biosynthesis	Threonine dehydratase biosynthetic (EC 4.3.1.19)
Amino Acids and Derivatives	Branched-chain amino acids	Branched-Chain Amino Acid Biosynthesis	2-isopropylmalate synthase (EC 2.3.3.13)
Amino Acids and Derivatives	Branched-chain amino acids	Leucine Biosynthesis	2-isopropylmalate synthase (EC 2.3.3.13)
Amino Acids and Derivatives	Branched-chain amino acids	Leucine Biosynthesis	3-isopropylmalate dehydratase large subunit (EC 4.2.1.33)
Amino Acids and Derivatives	Branched-chain amino acids	Leucine Biosynthesis	Branched-chain amino acid aminotransferase (EC 2.6.1.42)
Amino Acids and Derivatives	Branched-chain amino acids	Leucine Biosynthesis	3-isopropylmalate dehydratase small subunit (EC 4.2.1.33)
Amino Acids and Derivatives	Branched-chain amino acids	Leucine Biosynthesis	3-isopropylmalate dehydrogenase (EC 1.1.1.85)
Amino Acids and Derivatives	Arginine; urea cycle, polyamines	Putrescine utilization pathways	Uncharacterized protein in putrescine utilization cluster
Amino Acids and Derivatives	Arginine; urea cycle, polyamines	Putrescine utilization pathways	Gamma-glutamyl-putrescine synthetase (EC 6.3.1.11)
Amino Acids and Derivatives	Arginine; urea cycle, polyamines	Putrescine utilization pathways	Gamma-glutamyl-GABA hydrolase (EC 3.5.1.94)
Amino Acids and Derivatives	Arginine; urea cycle, polyamines	Putrescine utilization pathways	Gamma-glutamyl-putrescine oxidase (EC1.4.3.-)
Amino Acids and Derivatives	Arginine; urea cycle, polyamines	Putrescine utilization pathways	Gamma-aminobutyrate:alpha-ketoglutarate aminotransferase (EC 2.6.1.19)
Amino Acids and Derivatives	Arginine; urea cycle, polyamines	Arginine and Ornithine Degradation	Delta-1-pyrroline-5-carboxylate dehydrogenase (EC 1.2.1.88)
Amino Acids and Derivatives	Arginine; urea cycle, polyamines	Arginine and Ornithine Degradation	Arginine decarboxylase (EC 4.1.1.19)
Amino Acids and Derivatives	Arginine; urea cycle, polyamines	Arginine and Ornithine Degradation	NADP-specific glutamate dehydrogenase (EC 1.4.1.4)
Amino Acids and Derivatives	Arginine; urea cycle, polyamines	Arginine and Ornithine Degradation	Ornithine decarboxylase (EC 4.1.1.17)
Amino Acids and Derivatives	Arginine; urea cycle, polyamines	Arginine and Ornithine Degradation	Agmatinase (EC 3.5.3.11)
Amino Acids and Derivatives	Arginine; urea cycle, polyamines	Arginine and Ornithine Degradation	Ornithine carbamoyltransferase (EC 2.1.3.3)
Amino Acids and Derivatives	Arginine; urea cycle, polyamines	Polyamine Metabolism	Putrescine ABC transporter putrescine-binding protein PotF (TC 3.A.1.11.2)
Amino Acids and Derivatives	Arginine; urea cycle, polyamines	Polyamine Metabolism	Probable two-component sensor, near polyamine transporter
Amino Acids and Derivatives	Arginine; urea cycle, polyamines	Polyamine Metabolism	Ornithine decarboxylase (EC 4.1.1.17)
Amino Acids and Derivatives	Arginine; urea cycle, polyamines	Polyamine Metabolism	5'-methylthioadenosine nucleosidase (EC 3.2.2.16)

Amino Acids and Derivatives	Arginine; urea cycle, polyamines	Polyamine Metabolism	S-adenosylmethionine decarboxylase proenzyme (EC 4.1.1.50), prokaryotic class 1B
Amino Acids and Derivatives	Arginine; urea cycle, polyamines	Polyamine Metabolism	Agmatinase (EC 3.5.3.11)
Amino Acids and Derivatives	Arginine; urea cycle, polyamines	Polyamine Metabolism	Putrescine transport ATP-binding protein PotG (TC 3.A.1.11.2)
Amino Acids and Derivatives	Arginine; urea cycle, polyamines	Polyamine Metabolism	Putrescine transport system permease protein PotI (TC 3.A.1.11.2)
Amino Acids and Derivatives	Arginine; urea cycle, polyamines	Polyamine Metabolism	Spermidine synthase (EC 2.5.1.16)
Amino Acids and Derivatives	Arginine; urea cycle, polyamines	Polyamine Metabolism	Arginine decarboxylase (EC 4.1.1.19)
Amino Acids and Derivatives	Arginine; urea cycle, polyamines	Polyamine Metabolism	Putrescine transport system permease protein PotH (TC 3.A.1.11.2)
Amino Acids and Derivatives	Arginine; urea cycle, polyamines	Urea decomposition	Allophanate hydrolase (EC 3.5.1.54)
Amino Acids and Derivatives	Arginine; urea cycle, polyamines	Urea decomposition	Urea ABC transporter, ATPase protein UrtD
Amino Acids and Derivatives	Arginine; urea cycle, polyamines	Urea decomposition	Urease beta subunit (EC 3.5.1.5)
Amino Acids and Derivatives	Arginine; urea cycle, polyamines	Urea decomposition	Urea ABC transporter, permease protein UrtC
Amino Acids and Derivatives	Arginine; urea cycle, polyamines	Urea decomposition	Urease gamma subunit (EC 3.5.1.5)
Amino Acids and Derivatives	Arginine; urea cycle, polyamines	Urea decomposition	Urease accessory protein UreG
Amino Acids and Derivatives	Arginine; urea cycle, polyamines	Urea decomposition	Urease accessory protein UreD
Amino Acids and Derivatives	Arginine; urea cycle, polyamines	Urea decomposition	Urea ABC transporter, ATPase protein UrtE
Amino Acids and Derivatives	Arginine; urea cycle, polyamines	Urea decomposition	Urease accessory protein UreF
Amino Acids and Derivatives	Arginine; urea cycle, polyamines	Urea decomposition	Urease accessory protein UreE
Amino Acids and Derivatives	Arginine; urea cycle, polyamines	Urea decomposition	Urea ABC transporter, permease protein UrtB
Amino Acids and Derivatives	Arginine; urea cycle, polyamines	Urea decomposition	Urease alpha subunit (EC 3.5.1.5)
Amino Acids and Derivatives	Arginine; urea cycle, polyamines	Cyanophycin Metabolism	Cyanophycin synthase (EC 6.3.2.29)(EC 6.3.2.30)
Amino Acids and Derivatives	Arginine; urea cycle, polyamines	Cyanophycin Metabolism	Asparagine synthetase [glutamine-hydrolyzing] (EC 6.3.5.4)
Amino Acids and Derivatives	Arginine; urea cycle, polyamines	Urea carboxylase and Allophanate hydrolase cluster	Allophanate hydrolase (EC 3.5.1.54)
Amino Acids and Derivatives	Arginine; urea cycle, polyamines	Urease subunits	Urease accessory protein UreD
Amino Acids and Derivatives	Arginine; urea cycle, polyamines	Urease subunits	Urease gamma subunit (EC 3.5.1.5)
Amino Acids and Derivatives	Arginine; urea cycle, polyamines	Urease subunits	Urease accessory protein UreG

Amino Acids and Derivatives	Arginine; urea cycle, polyamines	Urease subunits	Urease accessory protein UreE
Amino Acids and Derivatives	Arginine; urea cycle, polyamines	Urease subunits	Urease accessory protein UreF
Amino Acids and Derivatives	Arginine; urea cycle, polyamines	Urease subunits	Urease alpha subunit (EC 3.5.1.5)
Amino Acids and Derivatives	Arginine; urea cycle, polyamines	Urease subunits	Urease beta subunit (EC 3.5.1.5)
Amino Acids and Derivatives	Alanine, serine, and glycine	Glycine and Serine Utilization	Glycine cleavage system H protein
Amino Acids and Derivatives	Alanine, serine, and glycine	Glycine and Serine Utilization	Seryl-tRNA synthetase (EC 6.1.1.11)
Amino Acids and Derivatives	Alanine, serine, and glycine	Glycine and Serine Utilization	Aminomethyltransferase (glycine cleavage system T protein) (EC 2.1.2.10)
Amino Acids and Derivatives	Alanine, serine, and glycine	Glycine and Serine Utilization	Glycerate kinase (EC 2.7.1.31)
Amino Acids and Derivatives	Alanine, serine, and glycine	Glycine and Serine Utilization	Phosphoserine aminotransferase (EC 2.6.1.52)
Amino Acids and Derivatives	Alanine, serine, and glycine	Glycine and Serine Utilization	D-3-phosphoglycerate dehydrogenase (EC 1.1.1.95)
Amino Acids and Derivatives	Alanine, serine, and glycine	Glycine and Serine Utilization	Glycine dehydrogenase [decarboxylating] (glycine cleavage system P protein) (EC 1.4.4.2)
Amino Acids and Derivatives	Alanine, serine, and glycine	Glycine and Serine Utilization	Serine hydroxymethyltransferase (EC 2.1.2.1)
Amino Acids and Derivatives	Alanine, serine, and glycine	Glycine and Serine Utilization	Phosphoserine phosphatase (EC 3.1.3.3)
Amino Acids and Derivatives	Alanine, serine, and glycine	Glycine Biosynthesis	Serine hydroxymethyltransferase (EC 2.1.2.1)
Amino Acids and Derivatives	Alanine, serine, and glycine	Glycine cleavage system	Aminomethyltransferase (glycine cleavage system T protein) (EC 2.1.2.10)
Amino Acids and Derivatives	Alanine, serine, and glycine	Glycine cleavage system	Glycine cleavage system H protein
Amino Acids and Derivatives	Alanine, serine, and glycine	Glycine cleavage system	Glycine dehydrogenase [decarboxylating] (glycine cleavage system P protein) (EC 1.4.4.2)
Amino Acids and Derivatives	Alanine, serine, and glycine	Serine Biosynthesis	D-3-phosphoglycerate dehydrogenase (EC 1.1.1.95)
Amino Acids and Derivatives	Alanine, serine, and glycine	Serine Biosynthesis	Phosphoserine phosphatase (EC 3.1.3.3)
Amino Acids and Derivatives	Alanine, serine, and glycine	Serine Biosynthesis	Serine hydroxymethyltransferase (EC 2.1.2.1)
Amino Acids and Derivatives	Alanine, serine, and glycine	Serine Biosynthesis	Phosphoserine aminotransferase (EC 2.6.1.52)
Amino Acids and Derivatives	Alanine, serine, and glycine	Alanine biosynthesis	Valine--pyruvate aminotransferase (EC 2.6.1.66)
Amino Acids and Derivatives	Alanine, serine, and glycine	Alanine biosynthesis	Ferredoxin, 2Fe-2S
Amino Acids and Derivatives	Alanine, serine, and glycine	Alanine biosynthesis	Iron-sulfur cluster assembly scaffold protein IscU
Amino Acids and Derivatives	Alanine, serine, and glycine	Alanine biosynthesis	Alanine racemase (EC 5.1.1.1)

Amino Acids and Derivatives	Alanine, serine, and glycine	Alanine biosynthesis	Cysteine desulfurase (EC 2.8.1.7)
Amino Acids and Derivatives	Alanine, serine, and glycine	Alanine biosynthesis	Chaperone protein HscA
Amino Acids and Derivatives	Alanine, serine, and glycine	Alanine biosynthesis	Branched-chain amino acid aminotransferase (EC 2.6.1.42)
Amino Acids and Derivatives	Alanine, serine, and glycine	Alanine biosynthesis	Iron-sulfur cluster regulator IscR
Amino Acids and Derivatives	Proline and 4-hydroxyproline	Proline, 4-hydroxyproline uptake and utilization	Proline iminopeptidase (EC 3.4.11.5)
Amino Acids and Derivatives	Proline and 4-hydroxyproline	Proline, 4-hydroxyproline uptake and utilization	Similar to eukaryotic Peptidyl prolyl 4-hydroxylase, alpha subunit (EC 1.14.11.2)
Amino Acids and Derivatives	Proline and 4-hydroxyproline	Proline, 4-hydroxyproline uptake and utilization	ProQ: influences osmotic activation of compatible solute ProP
Amino Acids and Derivatives	Proline and 4-hydroxyproline	Proline, 4-hydroxyproline uptake and utilization	Delta-1-pyrroline-5-carboxylate dehydrogenase (EC 1.2.1.88)
Amino Acids and Derivatives	Proline and 4-hydroxyproline	Proline Synthesis	Glutamate 5-kinase (EC 2.7.2.11)
Amino Acids and Derivatives	Proline and 4-hydroxyproline	Proline Synthesis	NADP-specific glutamate dehydrogenase (EC 1.4.1.4)
Amino Acids and Derivatives	Proline and 4-hydroxyproline	Proline Synthesis	RNA-binding C-terminal domain PUA
Amino Acids and Derivatives	Proline and 4-hydroxyproline	Proline Synthesis	Gamma-glutamyl phosphate reductase (EC 1.2.1.41)
Amino Acids and Derivatives	Proline and 4-hydroxyproline	Proline Synthesis	Pyrroline-5-carboxylate reductase (EC 1.5.1.2)
Amino Acids and Derivatives	Histidine Metabolism	Histidine Biosynthesis	Phosphoribosylformimino-5-aminoimidazole carboxamide ribotide isomerase (EC 5.3.1.16)
Amino Acids and Derivatives	Histidine Metabolism	Histidine Biosynthesis	Histidinol-phosphatase (EC 3.1.3.15)
Amino Acids and Derivatives	Histidine Metabolism	Histidine Biosynthesis	Imidazoleglycerol-phosphate dehydratase (EC 4.2.1.19)
Amino Acids and Derivatives	Histidine Metabolism	Histidine Biosynthesis	ATP phosphoribosyltransferase regulatory subunit (EC 2.4.2.17)
Amino Acids and Derivatives	Histidine Metabolism	Histidine Biosynthesis	Histidinol-phosphate aminotransferase (EC 2.6.1.9)
Amino Acids and Derivatives	Histidine Metabolism	Histidine Biosynthesis	Histidinol dehydrogenase (EC 1.1.1.23)
Amino Acids and Derivatives	Histidine Metabolism	Histidine Biosynthesis	Phosphoribosyl-AMP cyclohydrolase (EC 3.5.4.19)
Amino Acids and Derivatives	Histidine Metabolism	Histidine Biosynthesis	Phosphoribosyl-ATP pyrophosphatase (EC 3.6.1.31)
Amino Acids and Derivatives	Aromatic amino acids and derivatives	Aromatic amino acid interconversions with aryl acids	Indolepyruvate ferredoxin oxidoreductase, alpha and beta subunits
Amino Acids and Derivatives	Aromatic amino acids and derivatives	Chorismate: Intermediate for synthesis of Tryptophan, PAPA antibiotics, PABA, 3-hydroxyanthranilate and more.	Isochorismatase (EC 3.3.2.1)
Amino Acids and Derivatives	Aromatic amino acids and derivatives	Chorismate: Intermediate for synthesis of Tryptophan, PAPA antibiotics, PABA, 3-hydroxyanthranilate and more.	Anthranilate synthase, aminase component (EC 4.1.3.27)
Amino Acids and Derivatives	Aromatic amino acids and derivatives	Chorismate: Intermediate for synthesis of Tryptophan, PAPA antibiotics, PABA, 3-hydroxyanthranilate and more.	Aminodeoxychorismate lyase (EC 4.1.3.38)

Amino Acids and Derivatives	Aromatic amino acids and derivatives	Chorismate: Intermediate for synthesis of Tryptophan, PAPA antibiotics, PABA, 3-hydroxyanthranilate and more.	Phosphoribosylformimino-5-aminoimidazole carboxamide ribotide isomerase (EC 5.3.1.16)
Amino Acids and Derivatives	Aromatic amino acids and derivatives	Chorismate: Intermediate for synthesis of Tryptophan, PAPA antibiotics, PABA, 3-hydroxyanthranilate and more.	Para-aminobenzoate synthase, amidotransferase component (EC 2.6.1.85)
Amino Acids and Derivatives	Aromatic amino acids and derivatives	Chorismate: Intermediate for synthesis of Tryptophan, PAPA antibiotics, PABA, 3-hydroxyanthranilate and more.	Anthranilate phosphoribosyltransferase (EC 2.4.2.18)
Amino Acids and Derivatives	Aromatic amino acids and derivatives	Chorismate: Intermediate for synthesis of Tryptophan, PAPA antibiotics, PABA, 3-hydroxyanthranilate and more.	Phosphoribosylanthranilate isomerase (EC 5.3.1.24)
Amino Acids and Derivatives	Aromatic amino acids and derivatives	Chorismate: Intermediate for synthesis of Tryptophan, PAPA antibiotics, PABA, 3-hydroxyanthranilate and more.	Tryptophan synthase alpha chain (EC 4.2.1.20)
Amino Acids and Derivatives	Aromatic amino acids and derivatives	Chorismate: Intermediate for synthesis of Tryptophan, PAPA antibiotics, PABA, 3-hydroxyanthranilate and more.	Anthranilate synthase, amidotransferase component (EC 4.1.3.27)
Amino Acids and Derivatives	Aromatic amino acids and derivatives	Chorismate: Intermediate for synthesis of Tryptophan, PAPA antibiotics, PABA, 3-hydroxyanthranilate and more.	Indole-3-glycerol phosphate synthase (EC 4.1.1.48)
Amino Acids and Derivatives	Aromatic amino acids and derivatives	Chorismate: Intermediate for synthesis of Tryptophan, PAPA antibiotics, PABA, 3-hydroxyanthranilate and more.	Tryptophan synthase beta chain (EC 4.2.1.20)
Amino Acids and Derivatives	Aromatic amino acids and derivatives	Chorismate: Intermediate for synthesis of Tryptophan, PAPA antibiotics, PABA, 3-hydroxyanthranilate and more.	Para-aminobenzoate synthase, aminase component (EC 2.6.1.85)
Amino Acids and Derivatives	Aromatic amino acids and derivatives	Common Pathway For Synthesis of Aromatic Compounds (DAHP synthase to chorismate)	Shikimate kinase I (EC 2.7.1.71)
Amino Acids and Derivatives	Aromatic amino acids and derivatives	Common Pathway For Synthesis of Aromatic Compounds (DAHP synthase to chorismate)	Chorismate synthase (EC 4.2.3.5)
Amino Acids and Derivatives	Aromatic amino acids and derivatives	Common Pathway For Synthesis of Aromatic Compounds (DAHP synthase to chorismate)	3-dehydroquinate synthase (EC 4.2.3.4)
Amino Acids and Derivatives	Aromatic amino acids and derivatives	Common Pathway For Synthesis of Aromatic Compounds (DAHP synthase to chorismate)	Shikimate 5-dehydrogenase I alpha (EC 1.1.1.25)
Amino Acids and Derivatives	Aromatic amino acids and derivatives	Common Pathway For Synthesis of Aromatic Compounds (DAHP synthase to chorismate)	3-dehydroquinate dehydratase II (EC 4.2.1.10)
Amino Acids and Derivatives	Aromatic amino acids and derivatives	Common Pathway For Synthesis of Aromatic Compounds (DAHP synthase to chorismate)	2-keto-3-deoxy-D-arabino-heptulosonate-7-phosphate synthase I alpha (EC 2.5.1.54)
Amino Acids and Derivatives	Aromatic amino acids and derivatives	Tryptophan synthesis	Para-aminobenzoate synthase, aminase component (EC 2.6.1.85)
Amino Acids and Derivatives	Aromatic amino acids and derivatives	Tryptophan synthesis	Tryptophan synthase beta chain (EC 4.2.1.20)
Amino Acids and Derivatives	Aromatic amino acids and derivatives	Tryptophan synthesis	Para-aminobenzoate synthase, amidotransferase component (EC 2.6.1.85)
Amino Acids and Derivatives	Aromatic amino acids and derivatives	Tryptophan synthesis	Anthranilate phosphoribosyltransferase (EC 2.4.2.18)
Amino Acids and Derivatives	Aromatic amino acids and derivatives	Tryptophan synthesis	Anthranilate synthase, amidotransferase component (EC 4.1.3.27)
Amino Acids and Derivatives	Aromatic amino acids and derivatives	Tryptophan synthesis	Tryptophan synthase alpha chain (EC 4.2.1.20)
Amino Acids and Derivatives	Aromatic amino acids and derivatives	Tryptophan synthesis	Aminodeoxychorismate lyase (EC 4.1.3.38)
Amino Acids and Derivatives	Aromatic amino acids and derivatives	Tryptophan synthesis	Indole-3-glycerol phosphate synthase (EC 4.1.1.48)
Amino Acids and Derivatives	Aromatic amino acids and derivatives	Tryptophan synthesis	Anthranilate synthase, aminase component (EC 4.1.3.27)
Amino Acids and Derivatives	Aromatic amino acids and derivatives	Tryptophan synthesis	Phosphoribosylanthranilate isomerase (EC 5.3.1.24)

Amino Acids and Derivatives	Aromatic amino acids and derivatives	Phenylalanine and Tyrosine Branches from Chorismate	Prephenate dehydratase (EC 4.2.1.51)
Amino Acids and Derivatives	Aromatic amino acids and derivatives	Phenylalanine and Tyrosine Branches from Chorismate	Chorismate mutase I (EC 5.4.99.5)
Amino Acids and Derivatives	Aromatic amino acids and derivatives	Phenylalanine and Tyrosine Branches from Chorismate	Biosynthetic Aromatic amino acid aminotransferase alpha (EC 2.6.1.57)
Amino Acids and Derivatives	Aromatic amino acids and derivatives	Phenylalanine and Tyrosine Branches from Chorismate	Cyclohexadienyl dehydrogenase (EC 1.3.1.12)(EC 1.3.1.43)
Amino Acids and Derivatives	Glutamine, glutamate, aspartate, asparagine; ammonia assimilation	Glutamine, Glutamate, Aspartate and Asparagine Biosynthesis	Glutamate synthase [NADPH] small chain (EC 1.4.1.13)
Amino Acids and Derivatives	Glutamine, glutamate, aspartate, asparagine; ammonia assimilation	Glutamine, Glutamate, Aspartate and Asparagine Biosynthesis	Glutamate racemase (EC 5.1.1.3)
Amino Acids and Derivatives	Glutamine, glutamate, aspartate, asparagine; ammonia assimilation	Glutamine, Glutamate, Aspartate and Asparagine Biosynthesis	Asparagine synthetase [glutamine-hydrolyzing] (EC 6.3.5.4)
Amino Acids and Derivatives	Glutamine, glutamate, aspartate, asparagine; ammonia assimilation	Glutamine, Glutamate, Aspartate and Asparagine Biosynthesis	L-asparaginase (EC 3.5.1.1)
Amino Acids and Derivatives	Glutamine, glutamate, aspartate, asparagine; ammonia assimilation	Glutamine, Glutamate, Aspartate and Asparagine Biosynthesis	Aspartate aminotransferase (EC 2.6.1.1)
Amino Acids and Derivatives	Glutamine, glutamate, aspartate, asparagine; ammonia assimilation	Glutamine, Glutamate, Aspartate and Asparagine Biosynthesis	Glutamate synthase [NADPH] large chain (EC 1.4.1.13)
Amino Acids and Derivatives	Glutamine, glutamate, aspartate, asparagine; ammonia assimilation	Glutamine, Glutamate, Aspartate and Asparagine Biosynthesis	Ferredoxin-dependent glutamate synthase (EC 1.4.7.1)
Amino Acids and Derivatives	Glutamine, glutamate, aspartate, asparagine; ammonia assimilation	Glutamine, Glutamate, Aspartate and Asparagine Biosynthesis	Leucine-responsive regulatory protein, regulator for leucine (or lrp) regulon and high-affinity branched-chain amino acid transport system
Amino Acids and Derivatives	Glutamine, glutamate, aspartate, asparagine; ammonia assimilation	Glutamine, Glutamate, Aspartate and Asparagine Biosynthesis	Glutamine synthetase type I (EC 6.3.1.2)
Amino Acids and Derivatives	Glutamine, glutamate, aspartate, asparagine; ammonia assimilation	Glutamine, Glutamate, Aspartate and Asparagine Biosynthesis	NADP-specific glutamate dehydrogenase (EC 1.4.1.4)
Amino Acids and Derivatives	Glutamine, glutamate, aspartate, asparagine; ammonia assimilation	Glutamine synthetases	Glutamine synthetase type I (EC 6.3.1.2)
Amino Acids and Derivatives	Glutamine, glutamate, aspartate, asparagine; ammonia assimilation	Glutamate dehydrogenases	NADP-specific glutamate dehydrogenase (EC 1.4.1.4)
Metabolism of Aromatic Compounds	Peripheral pathways for catabolism of aromatic compounds	Benzoate degradation	Benzoate 1,2-dioxygenase alpha subunit (EC 1.14.12.10)
Metabolism of Aromatic Compounds	Peripheral pathways for catabolism of aromatic compounds	Benzoate degradation	Benzoate transport protein
Metabolism of Aromatic Compounds	Peripheral pathways for catabolism of aromatic compounds	Benzoate degradation	Benzoate 1,2-dioxygenase beta subunit (EC 1.14.12.10)
Metabolism of Aromatic Compounds	Peripheral pathways for catabolism of aromatic compounds	Benzoate degradation	Ortho-halobenzoate 1,2-dioxygenase alpha-ISP protein OhbB
Metabolism of Aromatic Compounds	Peripheral pathways for catabolism of aromatic compounds	Benzoate degradation	Ortho-halobenzoate 1,2-dioxygenase beta-ISP protein OhbA
Metabolism of Aromatic Compounds	Peripheral pathways for catabolism of aromatic compounds	Quinate degradation	3-dehydroquinate dehydratase II (EC 4.2.1.10)
Metabolism of Aromatic Compounds	Peripheral pathways for catabolism of aromatic compounds	Biphenyl Degradation	4-hydroxy-2-oxovalerate aldolase (EC 4.1.3.39)
Metabolism of Aromatic Compounds	Peripheral pathways for catabolism of aromatic compounds	Biphenyl Degradation	Large subunit naph/bph dioxygenase
Metabolism of Aromatic Compounds	Peripheral pathways for catabolism of aromatic compounds	Biphenyl Degradation	Biphenyl dioxygenase system ferredoxin component

Metabolism of Aromatic Compounds	Peripheral pathways for catabolism of aromatic compounds	Biphenyl Degradation	Biphenyl dioxygenase beta subunit (EC 1.14.12.18)
Metabolism of Aromatic Compounds	Peripheral pathways for catabolism of aromatic compounds	Biphenyl Degradation	2-keto-4-pentenoate hydratase (EC 4.2.1.80)
Metabolism of Aromatic Compounds	Peripheral pathways for catabolism of aromatic compounds	Biphenyl Degradation	2,3-dihydroxybiphenyl 1,2-dioxygenase (EC 1.13.11.39)
Metabolism of Aromatic Compounds	Peripheral pathways for catabolism of aromatic compounds	Biphenyl Degradation	Acetaldehyde dehydrogenase, acetylating, (EC 1.2.1.10) in gene cluster for degradation of phenols, cresols, catechol
Metabolism of Aromatic Compounds	Peripheral pathways for catabolism of aromatic compounds	Biphenyl Degradation	2-hydroxy-6-oxo-6-phenylhexa-2,4-dienoate hydrolase (EC 3.7.1.-)
Metabolism of Aromatic Compounds	Peripheral pathways for catabolism of aromatic compounds	Biphenyl Degradation	Biphenyl dioxygenase alpha subunit (EC 1.14.12.18)
Metabolism of Aromatic Compounds	Peripheral pathways for catabolism of aromatic compounds	Biphenyl Degradation	Dihydrodiol dehydrogenase (EC 1.3.1.56)
Metabolism of Aromatic Compounds	Peripheral pathways for catabolism of aromatic compounds	Salicylate ester degradation	putative facilitator of salicylate uptake
Metabolism of Aromatic Compounds	Metabolism of Aromatic Compounds - no subcategory	Gentisate degradation	Putative n-hydroxybenzoate hydroxylase
Metabolism of Aromatic Compounds	Metabolism of Aromatic Compounds - no subcategory	Gentisate degradation	Fumarylacetoacetate hydrolase family protein
Metabolism of Aromatic Compounds	Metabolism of Aromatic Compounds - no subcategory	Gentisate degradation	Gentisate 1,2-dioxygenase (EC 1.13.11.4)
Metabolism of Aromatic Compounds	Metabolism of Aromatic Compounds - no subcategory	Gentisate degradation	putative 4-hydroxybenzoyl-CoA thioesterase
Metabolism of Aromatic Compounds	Metabolism of central aromatic intermediates	Salicylate and gentisate catabolism	Gentisate 1,2-dioxygenase (EC 1.13.11.4)
Metabolism of Aromatic Compounds	Metabolism of central aromatic intermediates	Salicylate and gentisate catabolism	Fumarylacetoacetate hydrolase family protein
Metabolism of Aromatic Compounds	Metabolism of central aromatic intermediates	Salicylate and gentisate catabolism	putative facilitator of salicylate uptake
Metabolism of Aromatic Compounds	Metabolism of central aromatic intermediates	Salicylate and gentisate catabolism	Putative n-hydroxybenzoate hydroxylase
Metabolism of Aromatic Compounds	Metabolism of central aromatic intermediates	Catechol branch of beta-ketoadipate pathway	Succinyl-CoA:3-ketoacid-coenzyme A transferase subunit A (EC 2.8.3.5)
Metabolism of Aromatic Compounds	Metabolism of central aromatic intermediates	Catechol branch of beta-ketoadipate pathway	Succinyl-CoA:3-ketoacid-coenzyme A transferase subunit B (EC 2.8.3.5)
Resistance to antibiotics and toxic compounds	Resistance to antibiotics	Tolerance to colicin E2	Conserved uncharacterized protein CreA
Resistance to antibiotics and toxic compounds	Resistance to antibiotics	Resistance to fluoroquinolones	DNA gyrase subunit B (EC 5.99.1.3)
Resistance to antibiotics and toxic compounds	Resistance to antibiotics	Resistance to fluoroquinolones	DNA gyrase subunit A (EC 5.99.1.3)
Resistance to antibiotics and toxic compounds	Resistance to heavy metals	Cobalt-zinc-cadmium resistance	Heavy metal sensor histidine kinase
Resistance to antibiotics and toxic compounds	Resistance to heavy metals	Cobalt-zinc-cadmium resistance	Cobalt-zinc-cadmium resistance protein
Resistance to antibiotics and toxic compounds	Resistance to heavy metals	Cobalt-zinc-cadmium resistance	Transcriptional regulator, MerR family
Resistance to antibiotics and toxic compounds	Resistance to heavy metals	Cobalt-zinc-cadmium resistance	Copper-sensing two-component system response regulator CusR

Resistance to antibiotics and toxic compounds	Resistance to heavy metals	Resistance to chromium compounds	Chromate transport protein ChrA
Resistance to antibiotics and toxic compounds	Resistance to heavy metals	Copper homeostasis	Copper tolerance protein
Resistance to antibiotics and toxic compounds	Resistance to heavy metals	Copper homeostasis	Multidrug resistance transporter, Bcr/CflA family
Resistance to antibiotics and toxic compounds	Resistance to heavy metals	Copper homeostasis	Multicopper oxidase
Resistance to antibiotics and toxic compounds	Resistance to heavy metals	Copper homeostasis	Copper resistance protein B
Resistance to antibiotics and toxic compounds	Resistance to heavy metals	Copper homeostasis	Copper chaperone
Resistance to antibiotics and toxic compounds	Resistance to heavy metals	Copper homeostasis	Copper-translocating P-type ATPase (EC 3.6.3.4)
Resistance to antibiotics and toxic compounds	Resistance to heavy metals	Copper homeostasis	Copper resistance protein D
Resistance to antibiotics and toxic compounds	Resistance to heavy metals	Copper homeostasis	CopG protein
Resistance to antibiotics and toxic compounds	Resistance to heavy metals	Copper homeostasis	Copper-sensing two-component system response regulator CusR
Resistance to antibiotics and toxic compounds	Resistance to heavy metals	Copper homeostasis: copper tolerance	Magnesium and cobalt efflux protein CorC
Resistance to antibiotics and toxic compounds	Resistance to heavy metals	Copper homeostasis: copper tolerance	Copper homeostasis protein CutE

The annotated genes from the genome of strain H39-3-26^{T} involving in metabolizaton of carbohydrates, amino acids, and aromatic compounds and resistance to antibiotics and heavy metals.

Category	Subcategory	Subsystem	Annotated genes and its roles
Carbohydrates	Central carbohydrate metabolism	TCA Cycle	Malate dehydrogenase (EC 1.1.1.37)
Carbohydrates	Central carbohydrate metabolism	TCA Cycle	Succinyl-CoA ligase [ADP-forming] alpha chain (EC 6.2.1.5)
Carbohydrates	Central carbohydrate metabolism	TCA Cycle	Dihydrolipoamide dehydrogenase of pyruvate dehydrogenase complex (EC 1.8.1.4)
Carbohydrates	Central carbohydrate metabolism	TCA Cycle	Succinyl-CoA ligase [ADP-forming] beta chain (EC 6.2.1.5)
Carbohydrates	Central carbohydrate metabolism	TCA Cycle	Fumarate hydratase class I, aerobic (EC 4.2.1.2)
Carbohydrates	Central carbohydrate metabolism	TCA Cycle	Citrate synthase (si) (EC 2.3.3.1)
Carbohydrates	Central carbohydrate metabolism	TCA Cycle	Aconitate hydratase (EC 4.2.1.3)
Carbohydrates	Central carbohydrate metabolism	TCA Cycle	Dihydrolipoamide succinyltransferase component (E2) of 2-oxoglutarate dehydrogenase complex (EC 2.3.1.61)
Carbohydrates	Central carbohydrate metabolism	TCA Cycle	Dihydrolipoamide dehydrogenase of 2-oxoglutarate dehydrogenase (EC 1.8.1.4)
Carbohydrates	Central carbohydrate metabolism	TCA Cycle	Fumarate hydratase class II (EC 4.2.1.2)
Carbohydrates	Central carbohydrate metabolism	TCA Cycle	Aconitate hydratase 2 (EC 4.2.1.3)
Carbohydrates	Central carbohydrate metabolism	TCA Cycle	2-oxoglutarate dehydrogenase E1 component (EC 1.2.4.2)
Carbohydrates	Central carbohydrate metabolism	TCA Cycle	Isocitrate dehydrogenase [NADP] (EC 1.1.1.42)
Carbohydrates	Central carbohydrate metabolism	Pyruvate metabolism II: acetyl-CoA, acetogenesis from pyruvate	NAD-dependent protein deacetylase of SIR2 family
Carbohydrates	Central carbohydrate metabolism	Pyruvate metabolism II: acetyl-CoA, acetogenesis from pyruvate	Phosphate acetyltransferase (EC 2.3.1.8)
Carbohydrates	Central carbohydrate metabolism	Pyruvate metabolism II: acetyl-CoA, acetogenesis from pyruvate	Dihydrolipoamide dehydrogenase of pyruvate dehydrogenase complex (EC 1.8.1.4)
Carbohydrates	Central carbohydrate metabolism	Pyruvate metabolism II: acetyl-CoA, acetogenesis from pyruvate	Pyruvate dehydrogenase E1 component (EC 1.2.4.1)
Carbohydrates	Central carbohydrate metabolism	Pyruvate metabolism II: acetyl-CoA, acetogenesis from pyruvate	Dihydrolipoamide acetyltransferase component of pyruvate dehydrogenase complex (EC 2.3.1.12)
Carbohydrates	Central carbohydrate metabolism	Pyruvate metabolism II: acetyl-CoA, acetogenesis from pyruvate	Acetate permease ActP (cation/acetate symporter)
Carbohydrates	Central carbohydrate metabolism	Pyruvate metabolism II: acetyl-CoA, acetogenesis from pyruvate	Acetate kinase (EC 2.7.2.1)
Carbohydrates	Central carbohydrate metabolism	Pyruvate metabolism II: acetyl-CoA, acetogenesis from pyruvate	Aldehyde dehydrogenase (EC 1.2.1.3)
Carbohydrates	Central carbohydrate metabolism	Glycolysis and Gluconeogenesis	Phosphoenolpyruvate synthase (EC 2.7.9.2)
Carbohydrates	Central carbohydrate metabolism	Glycolysis and Gluconeogenesis	Fructose-bisphosphate aldolase class II (EC 4.1.2.13)
Carbohydrates	Central carbohydrate metabolism	Glycolysis and Gluconeogenesis	Fructose-1,6-bisphosphatase, type I (EC 3.1.3.11)
Carbohydrates	Central carbohydrate metabolism	Glycolysis and Gluconeogenesis	Enolase (EC 4.2.1.11)
Carbohydrates	Central carbohydrate metabolism	Glycolysis and Gluconeogenesis	Phosphoglycerate kinase (EC 2.7.2.3)
Carbohydrates	Central carbohydrate metabolism	Glycolysis and Gluconeogenesis	Pyruvate kinase (EC 2.7.1.40)
Carbohydrates	Central carbohydrate metabolism	Glycolysis and Gluconeogenesis	Pyruvate,phosphate dikinase (EC 2.7.9.1)

Carbohydrates	Central carbohydrate metabolism	Glycolysis and Gluconeogenesis	Glucose-6-phosphate isomerase (EC 5.3.1.9)
Carbohydrates	Central carbohydrate metabolism	Glycolysis and Gluconeogenesis	NAD-dependent glyceraldehyde-3-phosphate dehydrogenase (EC 1.2.1.12)
Carbohydrates	Central carbohydrate metabolism	Glycolysis and Gluconeogenesis	Triosephosphate isomerase (EC 5.3.1.1)
Carbohydrates	Central carbohydrate metabolism	Methylglyoxal Metabolism	Aldehyde dehydrogenase (EC 1.2.1.3)
Carbohydrates	Central carbohydrate metabolism	Methylglyoxal Metabolism	Hydroxyacylglutathione hydrolase (EC 3.1.2.6)
Carbohydrates	Central carbohydrate metabolism	Methylglyoxal Metabolism	Lactoylglutathione lyase (EC 4.4.1.5)
Carbohydrates	Central carbohydrate metabolism	Glycolate, glyoxylate interconversions	D-Lactate dehydrogenase, cytochrome c-dependent (EC 1.1.2.4)
Carbohydrates	Central carbohydrate metabolism	Glycolate, glyoxylate interconversions	Glycolate dehydrogenase (EC 1.1.99.14), subunit GlcD
Carbohydrates	Central carbohydrate metabolism	Glycolate, glyoxylate interconversions	Glyoxylate reductase (EC 1.1.1.79)
Carbohydrates	Central carbohydrate metabolism	Glycolate, glyoxylate interconversions	Glycolate dehydrogenase (EC 1.1.99.14), iron-sulfur subunit GlcF
Carbohydrates	Central carbohydrate metabolism	Glycolate, glyoxylate interconversions	Phosphoglycolate phosphatase (EC 3.1.3.18)
Carbohydrates	Central carbohydrate metabolism	Glycolate, glyoxylate interconversions	Hydroxypyruvate reductase (EC 1.1.1.81)
Carbohydrates	Central carbohydrate metabolism	Glycolate, glyoxylate interconversions	Glyoxylate reductase (EC 1.1.1.26)
Carbohydrates	Central carbohydrate metabolism	Glycolate, glyoxylate interconversions	Glycolate dehydrogenase (EC 1.1.99.14), FAD-binding subunit GlcE
Carbohydrates	Central carbohydrate metabolism	Dehydrogenase complexes	2-oxoglutarate dehydrogenase E1 component (EC 1.2.4.2)
Carbohydrates	Central carbohydrate metabolism	Dehydrogenase complexes	Dihydrolipoamide acyltransferase component of branched-chain alpha-keto acid dehydrogenase complex (EC 2.3.1.168)
Carbohydrates	Central carbohydrate metabolism	Dehydrogenase complexes	Dihydrolipoamide acetyltransferase component of pyruvate dehydrogenase complex (EC 2.3.1.12)
Carbohydrates	Central carbohydrate metabolism	Dehydrogenase complexes	Pyruvate dehydrogenase E1 component (EC 1.2.4.1)
Carbohydrates	Central carbohydrate metabolism	Dehydrogenase complexes	Dihydrolipoamide dehydrogenase of 2-oxoglutarate dehydrogenase (EC 1.8.1.4)
Carbohydrates	Central carbohydrate metabolism	Dehydrogenase complexes	Cytosol aminopeptidase PepA (EC 3.4.11.1)
Carbohydrates	Central carbohydrate metabolism	Dehydrogenase complexes	Dihydrolipoamide succinyltransferase component (E2) of 2-oxoglutarate dehydrogenase complex (EC 2.3.1.61)
Carbohydrates	Central carbohydrate metabolism	Pyruvate metabolism I: anaplerotic reactions, PEP	Phosphoenolpyruvate carboxykinase [GTP] (EC 4.1.1.32)
Carbohydrates	Central carbohydrate metabolism	Pyruvate metabolism I: anaplerotic reactions, PEP	Phosphoenolpyruvate synthase (EC 2.7.9.2)
Carbohydrates	Central carbohydrate metabolism	Pyruvate metabolism I: anaplerotic reactions, PEP	NADP-dependent malic enzyme (EC 1.1.1.40)
Carbohydrates	Central carbohydrate metabolism	Pyruvate metabolism I: anaplerotic reactions, PEP	Pyruvate,phosphate dikinase (EC 2.7.9.1)
Carbohydrates	Central carbohydrate metabolism	Pyruvate metabolism I: anaplerotic reactions, PEP	Pyruvate kinase (EC 2.7.1.40)
Carbohydrates	Central carbohydrate metabolism	Glyoxylate bypass	Malate synthase G (EC 2.3.3.9)
Carbohydrates	Central carbohydrate metabolism	Glyoxylate bypass	Isocitrate dehydrogenase phosphatase (EC 2.7.11.5)/kinase (EC 3.1.3.-)
Carbohydrates	Central carbohydrate metabolism	Glyoxylate bypass	Aconitate hydratase (EC 4.2.1.3)
Carbohydrates	Central carbohydrate metabolism	Glyoxylate bypass	Malate dehydrogenase (EC 1.1.1.37)

Carbohydrates	Central carbohydrate metabolism	Glyoxylate bypass	Citrate synthase (si) (EC 2.3.3.1)
Carbohydrates	Central carbohydrate metabolism	Glyoxylate bypass	Isocitrate lyase (EC 4.1.3.1)
Carbohydrates	Central carbohydrate metabolism	Glyoxylate bypass	Aconitate hydratase 2 (EC 4.2.1.3)
Carbohydrates	Central carbohydrate metabolism	Pyruvate Alanine Serine Interconversions	Alanine racemase (EC 5.1.1.1)
Carbohydrates	Central carbohydrate metabolism	Pyruvate Alanine Serine Interconversions	Valine--pyruvate aminotransferase (EC 2.6.1.66)
Carbohydrates	Central carbohydrate metabolism	Pyruvate Alanine Serine Interconversions	Branched-chain amino acid aminotransferase (EC 2.6.1.42)
Carbohydrates	Central carbohydrate metabolism	Pyruvate Alanine Serine Interconversions	D-alanine aminotransferase (EC 2.6.1.21)
Carbohydrates	Central carbohydrate metabolism	Pyruvate Alanine Serine Interconversions	Omega-amino acid--pyruvate aminotransferase (EC 2.6.1.18)
Carbohydrates	Monosaccharides	Mannose Metabolism	Mannose-6-phosphate isomerase (EC 5.3.1.8)
Carbohydrates	Monosaccharides	Mannose Metabolism	Phosphomannomutase (EC 5.4.2.8)
Carbohydrates	Di- and oligosaccharides	Trehalose Biosynthesis	Alpha,alpha-trehalose-phosphate synthase [UDP-forming] (EC 2.4.1.15)
Carbohydrates	Di- and oligosaccharides	Trehalose Biosynthesis	Trehalose-6-phosphate phosphatase (EC 3.1.3.12)
Carbohydrates	Di- and oligosaccharides	Trehalose Biosynthesis	Glucoamylase (EC 3.2.1.3)
Carbohydrates	Organic acids	Propionyl-CoA to Succinyl-CoA Module	Propionyl-CoA carboxylase carboxyl transferase subunit (EC 6.4.1.3)
Carbohydrates	Organic acids	Propionyl-CoA to Succinyl-CoA Module	Methylmalonyl-CoA epimerase (EC 5.1.99.1)
Carbohydrates	Organic acids	Propionyl-CoA to Succinyl-CoA Module	Propionyl-CoA carboxylase biotin-containing subunit (EC 6.4.1.3)
Carbohydrates	Organic acids	Propionyl-CoA to Succinyl-CoA Module	B12 binding domain of Methylmalonyl-CoA mutase (EC 5.4.99.2)
Carbohydrates	Organic acids	Propionyl-CoA to Succinyl-CoA Module	Methylmalonyl-CoA mutase (EC 5.4.99.2)
Amino Acids and Derivatives	Glutamine, glutamate, aspartate, asparagine; ammonia assimilation	Glutamine, Glutamate, Aspartate and Asparagine Biosynthesis	L-asparaginase (EC 3.5.1.1)
Amino Acids and Derivatives	Glutamine, glutamate, aspartate, asparagine; ammonia assimilation	Glutamine, Glutamate, Aspartate and Asparagine Biosynthesis	Glutaminase (EC 3.5.1.2)
Amino Acids and Derivatives	Glutamine, glutamate, aspartate, asparagine; ammonia assimilation	Glutamine, Glutamate, Aspartate and Asparagine Biosynthesis	Glutamate synthase [NADPH] small chain (EC 1.4.1.13)
Amino Acids and Derivatives	Glutamine, glutamate, aspartate, asparagine; ammonia assimilation	Glutamine, Glutamate, Aspartate and Asparagine Biosynthesis	Glutamate racemase (EC 5.1.1.3)
Amino Acids and Derivatives	Glutamine, glutamate, aspartate, asparagine; ammonia assimilation	Glutamine, Glutamate, Aspartate and Asparagine Biosynthesis	Aspartate aminotransferase (EC 2.6.1.1)
Amino Acids and Derivatives	Glutamine, glutamate, aspartate, asparagine; ammonia assimilation	Glutamine, Glutamate, Aspartate and Asparagine Biosynthesis	NAD-specific glutamate dehydrogenase (EC 1.4.1.2)
Amino Acids and Derivatives	Glutamine, glutamate, aspartate, asparagine; ammonia assimilation	Glutamine, Glutamate, Aspartate and Asparagine Biosynthesis	Glutamine synthetase type I (EC 6.3.1.2)
Amino Acids and Derivatives	Glutamine, glutamate, aspartate, asparagine; ammonia assimilation	Glutamine, Glutamate, Aspartate and Asparagine Biosynthesis	Leucine-responsive regulatory protein, regulator for leucine (or lrp) regulon and high-affinity branched-chain amino acid transport system
Amino Acids and Derivatives	Glutamine, glutamate, aspartate, asparagine; ammonia assimilation	Glutamine, Glutamate, Aspartate and Asparagine Biosynthesis	Ferredoxin-dependent glutamate synthase (EC 1.4.7.1)
Amino Acids and Derivatives	Glutamine, glutamate, aspartate, asparagine; ammonia assimilation	Glutamine, Glutamate, Aspartate and Asparagine Biosynthesis	Glutamate synthase [NADPH] large chain (EC 1.4.1.13)

Amino Acids and Derivatives	Glutamine, glutamate, aspartate, asparagine; ammonia assimilation	Glutamine, Glutamate, Aspartate and Asparagine Biosynthesis	NADP-specific glutamate dehydrogenase (EC 1.4.1.4)
Amino Acids and Derivatives	Glutamine, glutamate, aspartate, asparagine; ammonia assimilation	Glutamine synthetases	Glutamine synthetase type I (EC 6.3.1.2)
Amino Acids and Derivatives	Glutamine, glutamate, aspartate, asparagine; ammonia assimilation	Glutamate dehydrogenases	NAD-specific glutamate dehydrogenase (EC 1.4.1.2)
Amino Acids and Derivatives	Glutamine, glutamate, aspartate, asparagine; ammonia assimilation	Glutamate dehydrogenases	NADP-specific glutamate dehydrogenase (EC 1.4.1.4)
Amino Acids and Derivatives	Aromatic amino acids and derivatives	Chorismate: Intermediate for synthesis of Tryptophan, PAPA antibiotics, PABA, 3-hydroxyanthranilate and more.	Tryptophan synthase beta chain (EC 4.2.1.20)
Amino Acids and Derivatives	Aromatic amino acids and derivatives	Chorismate: Intermediate for synthesis of Tryptophan, PAPA antibiotics, PABA, 3-hydroxyanthranilate and more.	Para-aminobenzoate synthase, amidotransferase component (EC 2.6.1.85)
Amino Acids and Derivatives	Aromatic amino acids and derivatives	Chorismate: Intermediate for synthesis of Tryptophan, PAPA antibiotics, PABA, 3-hydroxyanthranilate and more.	Phosphoribosylformimino-5-aminoimidazole carboxamide ribotide isomerase (EC 5.3.1.16)
Amino Acids and Derivatives	Aromatic amino acids and derivatives	Chorismate: Intermediate for synthesis of Tryptophan, PAPA antibiotics, PABA, 3-hydroxyanthranilate and more.	Indole-3-glycerol phosphate synthase (EC 4.1.1.48)
Amino Acids and Derivatives	Aromatic amino acids and derivatives	Chorismate: Intermediate for synthesis of Tryptophan, PAPA antibiotics, PABA, 3-hydroxyanthranilate and more.	Anthranilate synthase, amidotransferase component (EC 4.1.3.27)
Amino Acids and Derivatives	Aromatic amino acids and derivatives	Chorismate: Intermediate for synthesis of Tryptophan, PAPA antibiotics, PABA, 3-hydroxyanthranilate and more.	Tryptophan synthase alpha chain (EC 4.2.1.20)
Amino Acids and Derivatives	Aromatic amino acids and derivatives	Chorismate: Intermediate for synthesis of Tryptophan, PAPA antibiotics, PABA, 3-hydroxyanthranilate and more.	Phosphoribosylanthranilate isomerase (EC 5.3.1.24)
Amino Acids and Derivatives	Aromatic amino acids and derivatives	Chorismate: Intermediate for synthesis of Tryptophan, PAPA antibiotics, PABA, 3-hydroxyanthranilate and more.	Anthranilate synthase, aminase component (EC 4.1.3.27)
Amino Acids and Derivatives	Aromatic amino acids and derivatives	Chorismate: Intermediate for synthesis of Tryptophan, PAPA antibiotics, PABA, 3-hydroxyanthranilate and more.	Anthranilate phosphoribosyltransferase (EC 2.4.2.18)
Amino Acids and Derivatives	Aromatic amino acids and derivatives	Chorismate: Intermediate for synthesis of Tryptophan, PAPA antibiotics, PABA, 3-hydroxyanthranilate and more.	Isochorismatase (EC 3.3.2.1)
Amino Acids and Derivatives	Aromatic amino acids and derivatives	Aromatic amino acid interconversions with aryl acids	Indolepyruvate ferredoxin oxidoreductase, alpha and beta subunits
Amino Acids and Derivatives	Aromatic amino acids and derivatives	Tryptophan synthesis	Anthranilate synthase, aminase component (EC 4.1.3.27)
Amino Acids and Derivatives	Aromatic amino acids and derivatives	Tryptophan synthesis	Phosphoribosylanthranilate isomerase (EC 5.3.1.24)
Amino Acids and Derivatives	Aromatic amino acids and derivatives	Tryptophan synthesis	Anthranilate synthase, amidotransferase component (EC 4.1.3.27)
Amino Acids and Derivatives	Aromatic amino acids and derivatives	Tryptophan synthesis	Tryptophan synthase alpha chain (EC 4.2.1.20)
Amino Acids and Derivatives	Aromatic amino acids and derivatives	Tryptophan synthesis	Indole-3-glycerol phosphate synthase (EC 4.1.1.48)
Amino Acids and Derivatives	Aromatic amino acids and derivatives	Tryptophan synthesis	Anthranilate phosphoribosyltransferase (EC 2.4.2.18)
Amino Acids and Derivatives	Aromatic amino acids and derivatives	Tryptophan synthesis	Para-aminobenzoate synthase, amidotransferase component (EC 2.6.1.85)
Amino Acids and Derivatives	Aromatic amino acids and derivatives	Tryptophan synthesis	Tryptophan synthase beta chain (EC 4.2.1.20)
Amino Acids and Derivatives	Aromatic amino acids and derivatives	Common Pathway For Synthesis of Aromatic Compounds (DAHP synthase to chorismate)	Shikimate 5-dehydrogenase I alpha (EC 1.1.1.25)
Amino Acids and Derivatives	Aromatic amino acids and derivatives	Common Pathway For Synthesis of Aromatic Compounds (DAHP synthase to chorismate)	3-dehydroquinate dehydratase II (EC 4.2.1.10)

Amino Acids and Derivatives	Aromatic amino acids and derivatives	Common Pathway For Synthesis of Aromatic Compounds (DAHP synthase to chorismate)	2-keto-3-deoxy-D-arabino-heptulosonate-7-phosphate synthase I alpha (EC 2.5.1.54)
Amino Acids and Derivatives	Aromatic amino acids and derivatives	Common Pathway For Synthesis of Aromatic Compounds (DAHP synthase to chorismate)	Shikimate kinase I (EC 2.7.1.71)
Amino Acids and Derivatives	Aromatic amino acids and derivatives	Common Pathway For Synthesis of Aromatic Compounds (DAHP synthase to chorismate)	Chorismate synthase (EC 4.2.3.5)
Amino Acids and Derivatives	Aromatic amino acids and derivatives	Common Pathway For Synthesis of Aromatic Compounds (DAHP synthase to chorismate)	3-dehydroquinate synthase (EC 4.2.3.4)
Amino Acids and Derivatives	Aromatic amino acids and derivatives	Phenylalanine and Tyrosine Branches from Chorismate	Prephenate dehydratase (EC 4.2.1.51)
Amino Acids and Derivatives	Aromatic amino acids and derivatives	Phenylalanine and Tyrosine Branches from Chorismate	Chorismate mutase I (EC 5.4.99.5)
Amino Acids and Derivatives	Aromatic amino acids and derivatives	Phenylalanine and Tyrosine Branches from Chorismate	Biosynthetic Aromatic amino acid aminotransferase alpha (EC 2.6.1.57)
Amino Acids and Derivatives	Aromatic amino acids and derivatives	Phenylalanine and Tyrosine Branches from Chorismate	Cyclohexadienyl dehydrogenase (EC 1.3.1.12)(EC 1.3.1.43)
Amino Acids and Derivatives	Aromatic amino acids and derivatives	Aromatic amino acid degradation	2-hydroxyhepta-2,4-diene-1,7-dioate isomerase (EC 5.3.3.-)
Amino Acids and Derivatives	Aromatic amino acids and derivatives	Aromatic amino acid degradation	Phenylalanine-4-hydroxylase (EC 1.14.16.1)
Amino Acids and Derivatives	Aromatic amino acids and derivatives	Aromatic amino acid degradation	4-hydroxyphenylpyruvate dioxygenase (EC 1.13.11.27)
Amino Acids and Derivatives	Aromatic amino acids and derivatives	Aromatic amino acid degradation	5-carboxymethyl-2-oxo-hex-3- ene-1,7-dioate decarboxylase (EC 4.1.1.68)
Amino Acids and Derivatives	Aromatic amino acids and derivatives	Aromatic amino acid degradation	Predicted 2-keto-4-pentenoate hydratase/2-oxohepta-3-ene-1,7-dioic acid hydratase
Amino Acids and Derivatives	Histidine Metabolism	Histidine Biosynthesis	Histidinol-phosphate aminotransferase (EC 2.6.1.9)
Amino Acids and Derivatives	Histidine Metabolism	Histidine Biosynthesis	Histidinol dehydrogenase (EC 1.1.1.23)
Amino Acids and Derivatives	Histidine Metabolism	Histidine Biosynthesis	Phosphoribosyl-AMP cyclohydrolase (EC 3.5.4.19)
Amino Acids and Derivatives	Histidine Metabolism	Histidine Biosynthesis	Phosphoribosyl-ATP pyrophosphatase (EC 3.6.1.31)
Amino Acids and Derivatives	Histidine Metabolism	Histidine Biosynthesis	Phosphoribosylformimino-5-aminoimidazole carboxamide ribotide isomerase (EC 5.3.1.16)
Amino Acids and Derivatives	Histidine Metabolism	Histidine Biosynthesis	Histidinol-phosphatase (EC 3.1.3.15)
Amino Acids and Derivatives	Histidine Metabolism	Histidine Biosynthesis	Imidazoleglycerol-phosphate dehydratase (EC 4.2.1.19)
Amino Acids and Derivatives	Histidine Metabolism	Histidine Biosynthesis	ATP phosphoribosyltransferase regulatory subunit (EC 2.4.2.17)
Amino Acids and Derivatives	Histidine Metabolism	Histidine Degradation	Urocanate hydratase (EC 4.2.1.49)
Amino Acids and Derivatives	Histidine Metabolism	Histidine Degradation	Formiminoglutamic iminohydrolase (EC 3.5.3.13)
Amino Acids and Derivatives	Histidine Metabolism	Histidine Degradation	Imidazolonepropionase (EC 3.5.2.7)
Amino Acids and Derivatives	Histidine Metabolism	Histidine Degradation	Histidine utilization repressor

Amino Acids and Derivatives	Histidine Metabolism	Histidine Degradation	N-formylglutamate deformylase (EC 3.5.1.68)
Amino Acids and Derivatives	Histidine Metabolism	Histidine Degradation	Histidine ammonia-lyase (EC 4.3.1.3)
Amino Acids and Derivatives	Proline and 4-hydroxyproline	Proline Synthesis	RNA-binding C-terminal domain PUA
Amino Acids and Derivatives	Proline and 4-hydroxyproline	Proline Synthesis	Pyrroline-5-carboxylate reductase (EC 1.5.1.2)
Amino Acids and Derivatives	Proline and 4-hydroxyproline	Proline Synthesis	Gamma-glutamyl phosphate reductase (EC 1.2.1.41)
Amino Acids and Derivatives	Proline and 4-hydroxyproline	Proline Synthesis	Glutamate 5-kinase (EC 2.7.2.11)
Amino Acids and Derivatives	Proline and 4-hydroxyproline	Proline Synthesis	NADP-specific glutamate dehydrogenase (EC 1.4.1.4)
Amino Acids and Derivatives	Proline and 4-hydroxyproline	Proline, 4-hydroxyproline uptake and utilization	Delta-1-pyrroline-5-carboxylate dehydrogenase (EC 1.2.1.88)
Amino Acids and Derivatives	Alanine, serine, and glycine	Alanine biosynthesis	Cysteine desulfurase (EC 2.8.1.7)
Amino Acids and Derivatives	Alanine, serine, and glycine	Alanine biosynthesis	Valine--pyruvate aminotransferase (EC 2.6.1.66)
Amino Acids and Derivatives	Alanine, serine, and glycine	Alanine biosynthesis	Iron-sulfur cluster assembly scaffold protein IscU
Amino Acids and Derivatives	Alanine, serine, and glycine	Alanine biosynthesis	Alanine racemase (EC 5.1.1.1)
Amino Acids and Derivatives	Alanine, serine, and glycine	Alanine biosynthesis	Ferredoxin, 2Fe-2S
Amino Acids and Derivatives	Alanine, serine, and glycine	Alanine biosynthesis	Branched-chain amino acid aminotransferase (EC 2.6.1.42)
Amino Acids and Derivatives	Alanine, serine, and glycine	Alanine biosynthesis	Iron-sulfur cluster regulator IscR
Amino Acids and Derivatives	Alanine, serine, and glycine	Alanine biosynthesis	Chaperone protein HscA
Amino Acids and Derivatives	Alanine, serine, and glycine	Glycine and Serine Utilization	Phosphoserine aminotransferase (EC 2.6.1.52)
Amino Acids and Derivatives	Alanine, serine, and glycine	Glycine and Serine Utilization	Glycine cleavage system H protein
Amino Acids and Derivatives	Alanine, serine, and glycine	Glycine and Serine Utilization	Seryl-tRNA synthetase (EC 6.1.1.11)
Amino Acids and Derivatives	Alanine, serine, and glycine	Glycine and Serine Utilization	L-serine dehydratase, (PLP)-dependent (EC 4.3.1.17)
Amino Acids and Derivatives	Alanine, serine, and glycine	Glycine and Serine Utilization	D-3-phosphoglycerate dehydrogenase (EC 1.1.1.95)
Amino Acids and Derivatives	Alanine, serine, and glycine	Glycine and Serine Utilization	Threonine dehydratase, catabolic (EC 4.3.1.19)
Amino Acids and Derivatives	Alanine, serine, and glycine	Glycine and Serine Utilization	Serine hydroxymethyltransferase (EC 2.1.2.1)
Amino Acids and Derivatives	Alanine, serine, and glycine	Glycine and Serine Utilization	Phosphoserine phosphatase (EC 3.1.3.3)
Amino Acids and Derivatives	Alanine, serine, and glycine	Glycine Biosynthesis	Serine hydroxymethyltransferase (EC 2.1.2.1)

Amino Acids and Derivatives	Alanine, serine, and glycine	Serine Biosynthesis	D-3-phosphoglycerate dehydrogenase (EC 1.1.1.95)
Amino Acids and Derivatives	Alanine, serine, and glycine	Serine Biosynthesis	Phosphoserine phosphatase (EC 3.1.3.3)
Amino Acids and Derivatives	Alanine, serine, and glycine	Serine Biosynthesis	Phosphoserine aminotransferase (EC 2.6.1.52)
Amino Acids and Derivatives	Alanine, serine, and glycine	Serine Biosynthesis	Serine hydroxymethyltransferase (EC 2.1.2.1)
Amino Acids and Derivatives	Arginine; urea cycle, polyamines	Arginine and Ornithine Degradation	Ornithine cyclodeaminase (EC 4.3.1.12)
Amino Acids and Derivatives	Arginine; urea cycle, polyamines	Arginine and Ornithine Degradation	NADP-specific glutamate dehydrogenase (EC 1.4.1.4)
Amino Acids and Derivatives	Arginine; urea cycle, polyamines	Arginine and Ornithine Degradation	Arginase (EC 3.5.3.1)
Amino Acids and Derivatives	Arginine; urea cycle, polyamines	Arginine and Ornithine Degradation	Delta-1-pyrroline-5-carboxylate dehydrogenase (EC 1.2.1.88)
Amino Acids and Derivatives	Arginine; urea cycle, polyamines	Arginine and Ornithine Degradation	Arginine decarboxylase (EC 4.1.1.19)
Amino Acids and Derivatives	Arginine; urea cycle, polyamines	Arginine and Ornithine Degradation	Ornithine decarboxylase (EC 4.1.1.17)
Amino Acids and Derivatives	Arginine; urea cycle, polyamines	Arginine and Ornithine Degradation	Ornithine carbamoyltransferase (EC 2.1.3.3)
Amino Acids and Derivatives	Arginine; urea cycle, polyamines	Polyamine Metabolism	Arginine decarboxylase (EC 4.1.1.19)
Amino Acids and Derivatives	Arginine; urea cycle, polyamines	Polyamine Metabolism	Putrescine transport system permease protein PotH (TC 3.A.1.11.2)
Amino Acids and Derivatives	Arginine; urea cycle, polyamines	Polyamine Metabolism	Spermidine synthase (EC 2.5.1.16)
Amino Acids and Derivatives	Arginine; urea cycle, polyamines	Polyamine Metabolism	Putrescine ABC transporter putrescine-binding protein PotF (TC 3.A.1.11.2)
Amino Acids and Derivatives	Arginine; urea cycle, polyamines	Polyamine Metabolism	Probable two-component sensor, near polyamine transporter
Amino Acids and Derivatives	Arginine; urea cycle, polyamines	Polyamine Metabolism	Ornithine decarboxylase (EC 4.1.1.17)
Amino Acids and Derivatives	Arginine; urea cycle, polyamines	Polyamine Metabolism	5'-methylthioadenosine nucleosidase (EC 3.2.2.16)
Amino Acids and Derivatives	Arginine; urea cycle, polyamines	Polyamine Metabolism	Putrescine transport ATP-binding protein PotG (TC 3.A.1.11.2)
Amino Acids and Derivatives	Arginine; urea cycle, polyamines	Polyamine Metabolism	S-adenosylmethionine decarboxylase proenzyme (EC 4.1.1.50), prokaryotic class 1B
Amino Acids and Derivatives	Arginine; urea cycle, polyamines	Polyamine Metabolism	Putrescine transport system permease protein PotI (TC 3.A.1.11.2)
Amino Acids and Derivatives	Arginine; urea cycle, polyamines	Urease subunits	Urease accessory protein UreD
Amino Acids and Derivatives	Arginine; urea cycle, polyamines	Urease subunits	Nickel-binding accessory protein UreJ-HupE
Amino Acids and Derivatives	Arginine; urea cycle, polyamines	Urease subunits	Urease accessory protein UreG
Amino Acids and Derivatives	Arginine; urea cycle, polyamines	Urease subunits	Urease gamma subunit (EC 3.5.1.5)

Amino Acids and Derivatives	Arginine; urea cycle, polyamines	Urease subunits	Urease alpha subunit (EC 3.5.1.5)
Amino Acids and Derivatives	Arginine; urea cycle, polyamines	Urease subunits	Urease beta subunit (EC 3.5.1.5)
Amino Acids and Derivatives	Arginine; urea cycle, polyamines	Urease subunits	Urease accessory protein UreF
Amino Acids and Derivatives	Arginine; urea cycle, polyamines	Urease subunits	Urease accessory protein UreE
Amino Acids and Derivatives	Arginine; urea cycle, polyamines	Urea carboxylase and Allophanate hydrolase cluster	Allophanate hydrolase (EC 3.5.1.54)
Amino Acids and Derivatives	Arginine; urea cycle, polyamines	Urea decomposition	Urea ABC transporter, ATPase protein UrtE
Amino Acids and Derivatives	Arginine; urea cycle, polyamines	Urea decomposition	Urease accessory protein UreD
Amino Acids and Derivatives	Arginine; urea cycle, polyamines	Urea decomposition	Urea ABC transporter, permease protein UrtB
Amino Acids and Derivatives	Arginine; urea cycle, polyamines	Urea decomposition	Urease accessory protein UreE
Amino Acids and Derivatives	Arginine; urea cycle, polyamines	Urea decomposition	Urease accessory protein UreF
Amino Acids and Derivatives	Arginine; urea cycle, polyamines	Urea decomposition	Urease alpha subunit (EC 3.5.1.5)
Amino Acids and Derivatives	Arginine; urea cycle, polyamines	Urea decomposition	Urea ABC transporter, ATPase protein UrtD
Amino Acids and Derivatives	Arginine; urea cycle, polyamines	Urea decomposition	Allophanate hydrolase (EC 3.5.1.54)
Amino Acids and Derivatives	Arginine; urea cycle, polyamines	Urea decomposition	Urease gamma subunit (EC 3.5.1.5)
Amino Acids and Derivatives	Arginine; urea cycle, polyamines	Urea decomposition	Urease accessory protein UreG
Amino Acids and Derivatives	Arginine; urea cycle, polyamines	Urea decomposition	Urea ABC transporter, permease protein UrtC
Amino Acids and Derivatives	Arginine; urea cycle, polyamines	Urea decomposition	Urease beta subunit (EC 3.5.1.5)
Amino Acids and Derivatives	Amino Acids and Derivatives - no subcategory	Creatine and Creatinine Degradation	Creatinine amidohydrolase (EC 3.5.2.10)
Amino Acids and Derivatives	Amino Acids and Derivatives - no subcategory	Creatine and Creatinine Degradation	Cytosine deaminase (EC 3.5.4.1)
Amino Acids and Derivatives	Branched-chain amino acids	Branched-Chain Amino Acid Biosynthesis	3-isopropylmalate dehydrogenase (EC 1.1.1.85)
Amino Acids and Derivatives	Branched-chain amino acids	Branched-Chain Amino Acid Biosynthesis	Threonine dehydratase biosynthetic (EC 4.3.1.19)
Amino Acids and Derivatives	Branched-chain amino acids	Branched-Chain Amino Acid Biosynthesis	Threonine dehydratase, catabolic (EC 4.3.1.19)
Amino Acids and Derivatives	Branched-chain amino acids	Branched-Chain Amino Acid Biosynthesis	Valine--pyruvate aminotransferase (EC 2.6.1.66)
Amino Acids and Derivatives	Branched-chain amino acids	Branched-Chain Amino Acid Biosynthesis	2-isopropylmalate synthase (EC 2.3.3.13)
Amino Acids and Derivatives	Branched-chain amino acids	Branched-Chain Amino Acid Biosynthesis	Dihydroxy-acid dehydratase (EC 4.2.1.9)

Amino Acids and Derivatives	Branched-chain amino acids	Branched-Chain Amino Acid Biosynthesis	Acetolactate synthase large subunit (EC 2.2.1.6)
Amino Acids and Derivatives	Branched-chain amino acids	Branched-Chain Amino Acid Biosynthesis	Leucine-responsive regulatory protein, regulator for leucine (or lrp) regulon and high-affinity branched-chain amino acid transport system
Amino Acids and Derivatives	Branched-chain amino acids	Branched-Chain Amino Acid Biosynthesis	3-isopropylmalate dehydratase large subunit (EC 4.2.1.33)
Amino Acids and Derivatives	Branched-chain amino acids	Branched-Chain Amino Acid Biosynthesis	Acetolactate synthase small subunit (EC 2.2.1.6)
Amino Acids and Derivatives	Branched-chain amino acids	Branched-Chain Amino Acid Biosynthesis	Branched-chain amino acid aminotransferase (EC 2.6.1.42)
Amino Acids and Derivatives	Branched-chain amino acids	Branched-Chain Amino Acid Biosynthesis	3-isopropylmalate dehydratase small subunit (EC 4.2.1.33)
Amino Acids and Derivatives	Branched-chain amino acids	Leucine Biosynthesis	3-isopropylmalate dehydrogenase (EC 1.1.1.85)
Amino Acids and Derivatives	Branched-chain amino acids	Leucine Biosynthesis	Branched-chain amino acid aminotransferase (EC 2.6.1.42)
Amino Acids and Derivatives	Branched-chain amino acids	Leucine Biosynthesis	3-isopropylmalate dehydratase small subunit (EC 4.2.1.33)
Amino Acids and Derivatives	Branched-chain amino acids	Leucine Biosynthesis	3-isopropylmalate dehydratase large subunit (EC 4.2.1.33)
Amino Acids and Derivatives	Branched-chain amino acids	Leucine Biosynthesis	2-isopropylmalate synthase (EC 2.3.3.13)
Amino Acids and Derivatives	Branched-chain amino acids	HMG CoA Synthesis	Hydroxymethylglutaryl-CoA lyase (EC 4.1.3.4)
Amino Acids and Derivatives	Branched-chain amino acids	HMG CoA Synthesis	Methylglutaconyl-CoA hydratase (EC 4.2.1.18)
Amino Acids and Derivatives	Branched-chain amino acids	HMG CoA Synthesis	Acetoacetyl-CoA synthetase [leucine] (EC 6.2.1.16)
Amino Acids and Derivatives	Branched-chain amino acids	HMG CoA Synthesis	Isovaleryl-CoA dehydrogenase (EC 1.3.8.4)
Amino Acids and Derivatives	Branched-chain amino acids	HMG CoA Synthesis	Methylcrotonyl-CoA carboxylase carboxyl transferase subunit (EC 6.4.1.4)
Amino Acids and Derivatives	Branched-chain amino acids	HMG CoA Synthesis	Methylcrotonyl-CoA carboxylase biotin-containing subunit (EC 6.4.1.4)
Amino Acids and Derivatives	Branched-chain amino acids	Leucine Degradation and HMG-CoA Metabolism	Acetoacetyl-CoA synthetase [leucine] (EC 6.2.1.16)
Amino Acids and Derivatives	Branched-chain amino acids	Leucine Degradation and HMG-CoA Metabolism	Succinyl-CoA:3-ketoacid-coenzyme A transferase subunit A (EC 2.8.3.5)
Amino Acids and Derivatives	Branched-chain amino acids	Leucine Degradation and HMG-CoA Metabolism	Methylcrotonyl-CoA carboxylase carboxyl transferase subunit (EC 6.4.1.4)
Amino Acids and Derivatives	Branched-chain amino acids	Leucine Degradation and HMG-CoA Metabolism	Isovaleryl-CoA dehydrogenase (EC 1.3.8.4)
Amino Acids and Derivatives	Branched-chain amino acids	Leucine Degradation and HMG-CoA Metabolism	Dihydrolipoamide acyltransferase component of branched-chain alpha-keto acid dehydrogenase complex (EC 2.3.1.168)
Amino Acids and Derivatives	Branched-chain amino acids	Leucine Degradation and HMG-CoA Metabolism	Branched-chain amino acid aminotransferase (EC 2.6.1.42)
Amino Acids and Derivatives	Branched-chain amino acids	Leucine Degradation and HMG-CoA Metabolism	Hydroxymethylglutaryl-CoA lyase (EC 4.1.3.4)
Amino Acids and Derivatives	Branched-chain amino acids	Leucine Degradation and HMG-CoA Metabolism	Methylcrotonyl-CoA carboxylase biotin-containing subunit (EC 6.4.1.4)

Amino Acids and Derivatives	Branched-chain amino acids	Leucine Degradation and HMG-CoA Metabolism	Branched-chain acyl-CoA dehydrogenase (EC 1.3.99.12)
Amino Acids and Derivatives	Branched-chain amino acids	Leucine Degradation and HMG-CoA Metabolism	Succinyl-CoA:3-ketoacid-coenzyme A transferase subunit B (EC 2.8.3.5)
Amino Acids and Derivatives	Branched-chain amino acids	Leucine Degradation and HMG-CoA Metabolism	Methylglutaconyl-CoA hydratase (EC 4.2.1.18)
Amino Acids and Derivatives	Lysine, threonine, methionine, and cysteine	Lysine Biosynthesis DAP Pathway	Aspartate-semialdehyde dehydrogenase (EC 1.2.1.11)
Amino Acids and Derivatives	Lysine, threonine, methionine, and cysteine	Lysine Biosynthesis DAP Pathway	2,3,4,5-tetrahydropyridine-2,6-dicarboxylate N-succinyltransferase (EC 2.3.1.117)
Amino Acids and Derivatives	Lysine, threonine, methionine, and cysteine	Lysine Biosynthesis DAP Pathway	N-succinyl-L,L-diaminopimelate desuccinylase (EC 3.5.1.18)
Amino Acids and Derivatives	Lysine, threonine, methionine, and cysteine	Lysine Biosynthesis DAP Pathway	4-hydroxy-tetrahydrodipicolinate synthase (EC 4.3.3.7)
Amino Acids and Derivatives	Lysine, threonine, methionine, and cysteine	Lysine Biosynthesis DAP Pathway	Diaminopimelate decarboxylase (EC 4.1.1.20)
Amino Acids and Derivatives	Lysine, threonine, methionine, and cysteine	Lysine Biosynthesis DAP Pathway	Diaminopimelate epimerase (EC 5.1.1.7)
Amino Acids and Derivatives	Lysine, threonine, methionine, and cysteine	Lysine Biosynthesis DAP Pathway	Aspartokinase (EC 2.7.2.4)
Amino Acids and Derivatives	Lysine, threonine, methionine, and cysteine	Lysine Biosynthesis DAP Pathway	4-hydroxy-tetrahydrodipicolinate reductase (EC 1.17.1.8)
Amino Acids and Derivatives	Lysine, threonine, methionine, and cysteine	Threonine and Homoserine Biosynthesis	Aspartate aminotransferase (EC 2.6.1.1)
Amino Acids and Derivatives	Lysine, threonine, methionine, and cysteine	Threonine and Homoserine Biosynthesis	Homoserine dehydrogenase (EC 1.1.1.3)
Amino Acids and Derivatives	Lysine, threonine, methionine, and cysteine	Threonine and Homoserine Biosynthesis	Threonine synthase (EC 4.2.3.1)
Amino Acids and Derivatives	Lysine, threonine, methionine, and cysteine	Threonine and Homoserine Biosynthesis	Aspartokinase (EC 2.7.2.4)
Amino Acids and Derivatives	Lysine, threonine, methionine, and cysteine	Threonine and Homoserine Biosynthesis	Aspartate-semialdehyde dehydrogenase (EC 1.2.1.11)
Amino Acids and Derivatives	Lysine, threonine, methionine, and cysteine	Threonine and Homoserine Biosynthesis	Homoserine kinase (EC 2.7.1.39)
Amino Acids and Derivatives	Lysine, threonine, methionine, and cysteine	Methionine Biosynthesis	O-succinylhomoserine sulfhydrylase (EC 2.5.1.48)
Amino Acids and Derivatives	Lysine, threonine, methionine, and cysteine	Methionine Biosynthesis	Homoserine kinase (EC 2.7.1.39)
Amino Acids and Derivatives	Lysine, threonine, methionine, and cysteine	Methionine Biosynthesis	O-acetylhomoserine sulfhydrylase (EC 2.5.1.49)
Amino Acids and Derivatives	Lysine, threonine, methionine, and cysteine	Methionine Biosynthesis	5,10-methylenetetrahydrofolate reductase (EC 1.5.1.20)
Amino Acids and Derivatives	Lysine, threonine, methionine, and cysteine	Methionine Biosynthesis	Adenosylhomocysteinase (EC 3.3.1.1)
Amino Acids and Derivatives	Lysine, threonine, methionine, and cysteine	Methionine Biosynthesis	Homoserine O-acetyltransferase (EC 2.3.1.31)
Amino Acids and Derivatives	Lysine, threonine, methionine, and cysteine	Methionine Biosynthesis	S-adenosylhomocysteine nucleosidase (EC 3.2.2.9)
Amino Acids and Derivatives	Lysine, threonine, methionine, and cysteine	Methionine Biosynthesis	Homoserine dehydrogenase (EC 1.1.1.3)

Amino Acids and Derivatives	Lysine, threonine, methionine, and cysteine	Methionine Biosynthesis	S-adenosylmethionine synthetase (EC 2.5.1.6)
Amino Acids and Derivatives	Lysine, threonine, methionine, and cysteine	Methionine Biosynthesis	Cysteine synthase (EC 2.5.1.47)
Amino Acids and Derivatives	Lysine, threonine, methionine, and cysteine	Methionine Biosynthesis	Cystathionine beta-lyase (EC 4.4.1.8)
Amino Acids and Derivatives	Lysine, threonine, methionine, and cysteine	Methionine Biosynthesis	Serine acetyltransferase (EC 2.3.1.30)
Amino Acids and Derivatives	Lysine, threonine, methionine, and cysteine	Lysine Biosynthesis DAP Pathway, GJO scratch	2,3,4,5-tetrahydropyridine-2,6-dicarboxylate N-succinyltransferase (EC 2.3.1.117)
Amino Acids and Derivatives	Lysine, threonine, methionine, and cysteine	Lysine Biosynthesis DAP Pathway, GJO scratch	Aspartate-semialdehyde dehydrogenase (EC 1.2.1.11)
Amino Acids and Derivatives	Lysine, threonine, methionine, and cysteine	Lysine Biosynthesis DAP Pathway, GJO scratch	4-hydroxy-tetrahydrodipicolinate synthase (EC 4.3.3.7)
Amino Acids and Derivatives	Lysine, threonine, methionine, and cysteine	Lysine Biosynthesis DAP Pathway, GJO scratch	N-succinyl-L,L-diaminopimelate desuccinylase (EC 3.5.1.18)
Amino Acids and Derivatives	Lysine, threonine, methionine, and cysteine	Lysine Biosynthesis DAP Pathway, GJO scratch	Diaminopimelate epimerase (EC 5.1.1.7)
Amino Acids and Derivatives	Lysine, threonine, methionine, and cysteine	Lysine Biosynthesis DAP Pathway, GJO scratch	Diaminopimelate decarboxylase (EC 4.1.1.20)
Amino Acids and Derivatives	Lysine, threonine, methionine, and cysteine	Lysine Biosynthesis DAP Pathway, GJO scratch	4-hydroxy-tetrahydrodipicolinate reductase (EC 1.17.1.8)
Amino Acids and Derivatives	Lysine, threonine, methionine, and cysteine	Lysine Biosynthesis DAP Pathway, GJO scratch	Aspartokinase (EC 2.7.2.4)
Amino Acids and Derivatives	Lysine, threonine, methionine, and cysteine	Methionine Degradation	S-adenosylhomocysteine nucleosidase (EC 3.2.2.9)
Amino Acids and Derivatives	Lysine, threonine, methionine, and cysteine	Methionine Degradation	Adenosylhomocysteinase (EC 3.3.1.1)
Amino Acids and Derivatives	Lysine, threonine, methionine, and cysteine	Methionine Degradation	S-adenosylmethionine synthetase (EC 2.5.1.6)
Amino Acids and Derivatives	Lysine, threonine, methionine, and cysteine	Methionine Degradation	2-Oxobutyrate oxidase, putative
Amino Acids and Derivatives	Lysine, threonine, methionine, and cysteine	Methionine Degradation	Pyruvate dehydrogenase E1 component (EC 1.2.4.1)
Amino Acids and Derivatives	Lysine, threonine, methionine, and cysteine	Cysteine Biosynthesis	Sulfite reductase [NADPH] hemoprotein beta-component (EC 1.8.1.2)
Amino Acids and Derivatives	Lysine, threonine, methionine, and cysteine	Cysteine Biosynthesis	Sulfate adenylyltransferase subunit 2 (EC 2.7.7.4)
Amino Acids and Derivatives	Lysine, threonine, methionine, and cysteine	Cysteine Biosynthesis	Sulfate adenylyltransferase subunit 1 (EC 2.7.7.4)
Amino Acids and Derivatives	Lysine, threonine, methionine, and cysteine	Cysteine Biosynthesis	Cysteine synthase (EC 2.5.1.47)
Amino Acids and Derivatives	Lysine, threonine, methionine, and cysteine	Cysteine Biosynthesis	Serine acetyltransferase (EC 2.3.1.30)
Amino Acids and Derivatives	Lysine, threonine, methionine, and cysteine	Cysteine Biosynthesis	Cys regulon transcriptional activator CysB
Amino Acids and Derivatives	Lysine, threonine, methionine, and cysteine	Cysteine Biosynthesis	Sulfate transport system permease protein CysT
Amino Acids and Derivatives	Lysine, threonine, methionine, and cysteine	Cysteine Biosynthesis	Sulfate and thiosulfate import ATP-binding protein CysA (EC 3.6.3.25)

Amino Acids and Derivatives	Lysine, threonine, methionine, and cysteine	Cysteine Biosynthesis	Sulfate and thiosulfate binding protein CysP
Amino Acids and Derivatives	Lysine, threonine, methionine, and cysteine	Cysteine Biosynthesis	Cysteine synthase B (EC 2.5.1.47)
Amino Acids and Derivatives	Lysine, threonine, methionine, and cysteine	Cysteine Biosynthesis	Sulfate transport system permease protein CysW
Amino Acids and Derivatives	Lysine, threonine, methionine, and cysteine	Lysine degradation	Lysine decarboxylase (EC 4.1.1.18)
Amino Acids and Derivatives	Lysine, threonine, methionine, and cysteine	Threonine degradation	Threonine dehydratase, catabolic (EC 4.3.1.19)
Amino Acids and Derivatives	Lysine, threonine, methionine, and cysteine	Threonine degradation	low-specificity D-threonine aldolase
Metabolism of Aromatic Compounds	Peripheral pathways for catabolism of aromatic compounds	Quinate degradation	3-dehydroquinate dehydratase II (EC 4.2.1.10)
Metabolism of Aromatic Compounds	Peripheral pathways for catabolism of aromatic compounds	Benzoate degradation	Ortho-halobenzoate 1,2-dioxygenase alpha-ISP protein OhbB
Metabolism of Aromatic Compounds	Peripheral pathways for catabolism of aromatic compounds	Benzoate degradation	Ortho-halobenzoate 1,2-dioxygenase beta-ISP protein OhbA
Metabolism of Aromatic Compounds	Metabolism of central aromatic intermediates	Catechol branch of beta-ketoadipate pathway	Succinyl-CoA:3-ketoacid-coenzyme A transferase subunit B (EC 2.8.3.5)
Metabolism of Aromatic Compounds	Metabolism of central aromatic intermediates	Catechol branch of beta-ketoadipate pathway	3-oxoadipate CoA-transferase subunit B (EC 2.8.3.6)
Metabolism of Aromatic Compounds	Metabolism of central aromatic intermediates	Catechol branch of beta-ketoadipate pathway	Succinyl-CoA:3-ketoacid-coenzyme A transferase subunit A (EC 2.8.3.5)
Metabolism of Aromatic Compounds	Metabolism of central aromatic intermediates	Catechol branch of beta-ketoadipate pathway	3-oxoadipate CoA-transferase subunit A (EC 2.8.3.6)
Metabolism of Aromatic Compounds	Metabolism of central aromatic intermediates	Salicylate and gentisate catabolism	Fumarylacetoacetate hydrolase family protein
Metabolism of Aromatic Compounds	Metabolism of central aromatic intermediates	Salicylate and gentisate catabolism	Gentisate 1,2-dioxygenase (EC 1.13.11.4)
Metabolism of Aromatic Compounds	Metabolism of central aromatic intermediates	Salicylate and gentisate catabolism	Putative n-hydroxybenzoate hydroxylase
Metabolism of Aromatic Compounds	Metabolism of central aromatic intermediates	Salicylate and gentisate catabolism	Maleylacetoacetate isomerase (EC 5.2.1.2)
Metabolism of Aromatic Compounds	Metabolism of Aromatic Compounds - no subcategory	Gentisate degradation	Maleylacetoacetate isomerase (EC 5.2.1.2)
Metabolism of Aromatic Compounds	Metabolism of Aromatic Compounds - no subcategory	Gentisate degradation	Putative n-hydroxybenzoate hydroxylase
Metabolism of Aromatic Compounds	Metabolism of Aromatic Compounds - no subcategory	Gentisate degradation	putative 4-hydroxybenzoyl-CoA thioesterase
Metabolism of Aromatic Compounds	Metabolism of Aromatic Compounds - no subcategory	Gentisate degradation	Gentisate 1,2-dioxygenase (EC 1.13.11.4)
Metabolism of Aromatic Compounds	Metabolism of Aromatic Compounds - no subcategory	Gentisate degradation	Fumarylacetoacetate hydrolase family protein
Resistance to antibiotics and toxic compounds	Resistance to antibiotics	Beta-lactamase	Metal-dependent hydrolases of the beta-lactamase superfamily I
Resistance to antibiotics and toxic compounds	Resistance to antibiotics	Resistance to fluoroquinolones	DNA gyrase subunit A (EC 5.99.1.3)
Resistance to antibiotics and toxic compounds	Resistance to antibiotics	Resistance to fluoroquinolones	DNA gyrase subunit B (EC 5.99.1.3)

Resistance to antibiotics and toxic compounds	Resistance to heavy metals	Copper homeostasis	Copper-sensing two-component system response regulator CusR
Resistance to antibiotics and toxic compounds	Resistance to heavy metals	Copper homeostasis	Copper-translocating P-type ATPase (EC 3.6.3.4)
Resistance to antibiotics and toxic compounds	Resistance to heavy metals	Copper homeostasis	Copper chaperone
Resistance to antibiotics and toxic compounds	Resistance to heavy metals	Copper homeostasis	Copper resistance protein B
Resistance to antibiotics and toxic compounds	Resistance to heavy metals	Copper homeostasis	Multidrug resistance transporter, Bcr/CflA family
Resistance to antibiotics and toxic compounds	Resistance to heavy metals	Copper homeostasis	Copper sensory histidine kinase CusS
Resistance to antibiotics and toxic compounds	Resistance to heavy metals	Copper homeostasis	Multicopper oxidase
Resistance to antibiotics and toxic compounds	Resistance to heavy metals	Copper homeostasis	Cu(I)-responsive transcriptional regulator
Resistance to antibiotics and toxic compounds	Resistance to heavy metals	Copper homeostasis	Copper tolerance protein
Resistance to antibiotics and toxic compounds	Resistance to heavy metals	Copper homeostasis: copper tolerance	Magnesium and cobalt efflux protein CorC
Resistance to antibiotics and toxic compounds	Resistance to heavy metals	Copper homeostasis: copper tolerance	Copper homeostasis protein CutE
Resistance to antibiotics and toxic compounds	Resistance to heavy metals	Cobalt-zinc-cadmium resistance	DNA-binding heavy metal response regulator
Resistance to antibiotics and toxic compounds	Resistance to heavy metals	Cobalt-zinc-cadmium resistance	Copper sensory histidine kinase CusS
Resistance to antibiotics and toxic compounds	Resistance to heavy metals	Cobalt-zinc-cadmium resistance	Transcriptional regulator, MerR family
Resistance to antibiotics and toxic compounds	Resistance to heavy metals	Cobalt-zinc-cadmium resistance	Cobalt-zinc-cadmium resistance protein
Resistance to antibiotics and toxic compounds	Resistance to heavy metals	Cobalt-zinc-cadmium resistance	Copper-sensing two-component system response regulator CusR
Resistance to antibiotics and toxic compounds	Resistance to heavy metals	Resistance to chromium compounds	Chromate transport protein ChrA

The predicted prophage-related proteins from the genome of strain H39-3-26^T by PHAEST program.

Number	Category	CDS Position	BLAST Hit
1	Head protein	1849438..1849728	PP_01780;putative head-tail adaptor;phage;-;PHAGE_Geobac_E2_NC_009552
2	Head protein	1849752..1850030	PP_01781;head-tail connector family protein;phage;-;PHAGE_Pseudo_phi2_NC_030931
3	Head protein	1854068..1854490	PP_01786;putative prohead protease;phage;-;PHAGE_Clostr_phi3626_NC_003524
4	Head protein	1854615..1855841	PP_01787;major capsid protein;phage;-;PHAGE_Synech_S_CBS3_NC_015465
5	Head protein	3269829..3270722	PP_03157;Mu-like prophage major head subunit gpT;phage;-;PHAGE_Pseudo_JBD25_NC_027992
6	Hypothetical protein	1852297..1852854	PP_01784;hypothetical protein;phage;-;PHAGE_Paraco_Shpa_NC_041868
7	Hypothetical protein	1857812..1858015	PP_01789;hypothetical protein;phage;-;PHAGE_Burkho_BcepIL02_NC_012743
8	Hypothetical protein	3267661..3268077	PP_03154;hypothetical protein;phage;-;PHAGE_Bacill_BalMu_1_NC_030945
9	Hypothetical protein	3269390..3269800	PP_03156;hypothetical protein;phage;-;PHAGE_Pseudo_JBD25_NC_027992
10	Hypothetical protein	3272422..3273339	PP_03162;hypothetical protein;phage;-;PHAGE_Rhodob_RcapMu_NC_016165
11	Integrase	1859432..1860469	PP_01792;putative integrase;phage;-;PHAGE_Pseudo_H66_NC_042342
12	Phage-like protein	1856935..1857768	PP_01788;putative DNA topoisomerase;phage;-;PHAGE_Pseudo_PaMx25_NC_041953
13	Phage-like protein	3267213..3267659	PP_03153;gp02;phage;-;PHAGE_Burkho_BcepMu_NC_005882
14	Phage-like protein	3270752..3271048	PP_03158;Mu phage uncharacterized protein;phage;-;PHAGE_Ralsto_RS138_NC_029107
15	Phage-like protein	3271192..3271728	PP_03159;PF07030 family protein;phage;-;PHAGE_Pseudo_JBD25_NC_027992
16	Portal protein	1850234..1851733	PP_01782;portal protein;phage;-;PHAGE_Escher_ECP1_NC_049926
17	Portal protein	1852863..1854035	PP_01785;portal-like protein;phage;-;PHAGE_Paraco_Shpa_NC_041868
18	Protease	3268198..3269364	PP_03155;protease (I) and scaffold (Z) protein;phage;-;PHAGE_Pseudo_JBD93_NC_030918
19	Tail protein	3274301..3279232	PP_03166;putative tail component protein;phage;-;PHAGE_Pseudo_MP48_NC_024782
20	Terminase	1851887..1852219	PP_01783;terminase small subunit;phage;-;PHAGE_Entero_mEp390_NC_019721

The predicted prophage-related proteins from the genome of strain H3SJ34-1^{T} by PHAEST program.

Number	Category	CDS Position	BLAST Hit
1	Fiber protein	2294894..2297308	PP_02294;tail hyaluronidase/tail fiber;phage;-;PHAGE_Bacter_crAss001_NC_049977
2	Head protein	2278875..2280014	PP_01781;head-tail connector family protein;phage;-;PHAGE_Pseudo_phi2_NC_030931
3	Head protein	2280016..2280639	PP_02272;head decoration protein D;phage;-;PHAGE_Aurant_AmM_1_NC_027334
4	Head protein	2280755..2281780	PP_02273;major capsid;phage;-;PHAGE_Aurant_AmM_1_NC_027334
5	Hypothetical protein	3269829..3270722	PP_02284;hypothetical protein;phage;-;PHAGE_Rhizob_RHEph04_NC_041908
6	Hypothetical protein	2288881..2289129	PP_02285;hypothetical protein;phage;-;PHAGE_Rhizob_RHEph04_NC_041908
7	Hypothetical protein	2289146..2290030	PP_02286;hypothetical protein;phage;-;PHAGE_Rhizob_RHEph04_NC_041908
8	Hypothetical protein	2292144..2292962	PP_02290;hypothetical protein;phage;-;PHAGE_Rhizob_RHEph04_NC_041908
9	Hypothetical protein	2299236..2300276	PP_02299;hypothetical protein;phage;-;PHAGE_Bacill_BM5_NC_029069
10	Phage-like protein	2281935..2282339	PP_02274;putative structural protein;phage;-;PHAGE_Rhizob_RHEph04_NC_041908
11	Phage-like protein	2284778..2285206	PP_02278;putative structural protein;phage;-;PHAGE_Rhizob_RHEph04_NC_041908
12	Phage-like protein	2297437..2298036	PP_02295;putative endolysin;phage;-;PHAGE_Acinet_phiAC_1_NC_028995
13	Plate protein	2290087..2290662	PP_02287;putative baseplate assembly protein;phage;-;PHAGE_Rhizob_RHEph04_NC_041908
14	Plate protein	2290851..2291213	PP_02288;putative baseplate component;phage;-;PHAGE_Pantoe_vB_PagM_AAM37_NC_048766
15	Plate protein	2291232..2292140	PP_02289;putative baseplate assembly protein;phage;-;PHAGE_Rhizob_RHEph04_NC_041908
16	Tail protein	2283323..2284753	PP_02277;tail collar domain;phage;-;PHAGE_Sinorh_phiLM21_NC_029046
17	Tail protein	2292985..2293770	PP_02291;putative tail collar domain-containing protein;phage;-;PHAGE_Pseudo_PPpW_3_NC_023006

The predicted prophage-related proteins from the genome of strain H3M7-6^T by PHAEST program.

Number	Category	CDS Position	BLAST Hit
1	Head protein	2580096..2581073	PP_02402;major capsid protein;phage;-;PHAGE_Rheinh_vB_RspM_Barba18A_NC_048189
2	Head protein	2581133..2581501	PP_01781;head-tail connector family protein;phage;-;PHAGE_Pseudo_phi2_NC_030931
3	Head protein	2581529..2582767	PP_02404;putative capsid assembly protein/protease;phage;-;PHAGE_Burkho_phiE125_NC_003309
4	Head protein	2584901..2585392	PP_02408;capsid-related protein;phage;-;PHAGE_Sinorh_phiM7_NC_041929
5	Head protein	3269829..3270722	PP_02418;RuvC-like resolvase;phage;-;PHAGE_Rhodoc_Sleepyhead_NC_048782
6	Hypothetical protein	2563782..2564072	PP_02386;hypothetical protein;phage;-;PHAGE_Rhizob_vB_RleS_L338C_NC_023502
7	Hypothetical protein	2564149..2564307	PP_02387;hypothetical protein;phage;-;PHAGE_Ralsto_RS138_NC_029107
8	Hypothetical protein	2566777..2567862	PP_02390;hypothetical protein;phage;-;PHAGE_Ralsto_RS138_NC_029107
9	Hypothetical protein	2572720..2573061	PP_02393;hypothetical protein;phage;-;PHAGE_Ralsto_RS138_NC_029107
10	Hypothetical protein	2579346..2579792	PP_02400;hypothetical protein;phage;-;PHAGE_Synech_S_CBS3_NC_015465
11	Hypothetical protein	2584509..2584874	PP_02407;hypothetical protein;phage;-;PHAGE_Rhizob_vB_RleM_PPF1_NC_025427
12	Hypothetical protein	2588034..2588231	PP_02411;hypothetical protein;phage;-;PHAGE_Agroba_Atu_ph07_NC_042013
13	Hypothetical protein	2593900..2596086	PP_02419;hypothetical protein;phage;-;PHAGE_Bacill_phi105_NC_004167
14	Hypothetical protein	2598210..2599064	PP_02423;hypothetical protein;phage;-;PHAGE_Pseudo_YMC11/02/R656_NC_028657
15	Hypothetical protein	2601062..2601373	PP_02427;hypothetical protein;phage;-;PHAGE_Pseudo_PPpW_3_NC_023006
16	Hypothetical protein	2604122..2604676	PP_02432;hypothetical protein;phage;-;PHAGE_Mycoba_Adler_NC_023591
17	Hypothetical protein	2604727..2604960	PP_02433;hypothetical protein;phage;-;PHAGE_Entero_mEp390_NC_019721
18	Integrase	2602005..2603252	PP_02430;site-specific integrase;phage;-;PHAGE_Entero_EFC_1_NC_025453
19	Phage-like protein	2562845..2563303	PP_02384;p15;phage;-;PHAGE_Bacill_Nf_NC_049976
20	Phage-like protein	2589672..2590847	PP_02414;DNA modification methylase;phage;-;PHAGE_Bacill_vB_BtS_B83_NC_048762
21	Phage-like protein	2590940..2592445	PP_02415;DNA methylase;phage;-;PHAGE_Psychr_pOW20_A_NC_020841
22	Phage-like protein	2596184..2596780	PP_02420;RNA polymerase sigma factor;phage;-;PHAGE_Klebsi_ST13_OXA48phi12.1_NC_049453
23	Phage-like protein	2596982..2597653	PP_02421;putative nuclease;phage;-;PHAGE_Entero_CAjan_NC_028776
24	Phage-like protein	2599723..2600553	PP_02425;ATPase;phage;-;PHAGE_Escher_Seurat_NC_027378
25	Portal protein	2582777..2584201	PP_02405;portal protein;phage;-;PHAGE_Vibrio_vB_VpaM_MAR_NC_019722
26	Regulatory protein	2560735..2561421	PP_02382;hybrid sensor histidine kinase - response regulator;phage;-;PHAGE_Altero_vB_AmeM_PT11_V22_NC_048847
27	Tail protein	2564363..2566381	PP_02388;putative tail protein c;phage;-;PHAGE_Stenot_S1_NC_011589
28	Tail protein	2567866..2569131	PP_02391;tail assembly protein;phage;-;PHAGE_Stenot_vB_SmaS_DLP_5_NC_042082
29	Tail protein	2569155..2572718	PP_02392;putative tail protein b;phage;-;PHAGE_Stenot_S1_NC_011589
30	Tail protein	2573072..2577115	PP_02394;phage tail tape measure protein;phage;-;PHAGE_Cronob_ENT39118_NC_019934
31	Tail protein	2578397..2579077	PP_02398;putative tail sheath protein;phage;-;PHAGE_Pseudo_vB_PaeS_PM105_NC_028667
32	Terminase	2585410..2587269	PP_02409;large terminase subunit;phage;-;PHAGE_Synech_S_CBS3_NC_015465

The predicted plasimid sequences from the genome of strain H39-3-26$^{T.}$

Sample name	Contig name	Probability (%)	Contig length (bp)	GC content (%)	Acc num	Acc plasmid name	Identity (%)
H39_3_26_GCF_029211125	NZ_JARGEN010000088.1	-	642	56.23	-	-	-
H39_3_26_GCF_029211125	NZ_JARGEN010000092.1	75.52	7528	63.67	-	-	-
H39_3_26_GCF_029211125	NZ_JARGEN010000004.1	81.79	3661	59.85	-	-	-
H39_3_26_GCF_029211125	NZ_JARGEN010000060.1	81.31	14768	61.79	NZ_LR594670.1	5	99.746
H39_3_26_GCF_029211125	NZ_JARGEN010000090.1	-	564	61.17	NZ_CP045303.1	unnamed1	80.226
H39_3_26_GCF_029211125	NZ_JARGEN010000020.1	-	4412	57.77	-	-	-
H39_3_26_GCF_029211125	NZ_JARGEN010000099.1	-	801	47.44	-	-	-
H39_3_26_GCF_029211125	NZ_JARGEN010000072.1	97.42	1192	56.96	NZ_CP027671.1	unnamed2	89.567
H39_3_26_GCF_029211125	NZ_JARGEN010000066.1	75.7	3351	60.73	-	-	-
H39_3_26_GCF_029211125	NZ_JARGEN010000078.1	98.44	12536	57.68	NZ_KR106190.1	pHS87a	85.106
H39_3_26_GCF_029211125	NZ_JARGEN010000044.1	96.63	1494	62.38	NC_007974.2		78.906
H39_3_26_GCF_029211125	NZ_JARGEN010000041.1	-	3933	56.34	-	-	-
H39_3_26_GCF_029211125	NZ_JARGEN010000098.1	89.04	753	58.83	NZ_CP019241.1	unnamed1	85.557
H39_3_26_GCF_029211125	NZ_JARGEN010000086.1	71.81	13968	63.6	-	-	-
H39_3_26_GCF_029211125	NZ_JARGEN010000081.1	-	1440	56.11	-	-	-

The predicted plasimid sequences from the genome of strain H3SJ34-1[T.]

Sample name	Contig name	Probability (%)	Contig length (bp)	GC content (%)	Acc num	Acc plasmid name	Identity (%)
H3SJ34_1_GCA_029222685	JARHVB010000068.1	98.33	4918	62.91	NZ_CP023451.1	p2	75.385
H3SJ34_1_GCA_029222685	JARHVB010000078.1	72.13	883	62.4	NZ_CP034088.1	pGW6_2	77.477
H3SJ34_1_GCA_029222685	JARHVB010000051.1	90.16	16505	59.93	NZ_CP034088.1	pGW6_2	76.566
H3SJ34_1_GCA_029222685	JARHVB010000079.1	77.93	43284	60.25	NZ_CP044544.1	pBbPL7HG1	84.925
H3SJ34_1_GCA_029222685	JARHVB010000080.1	98.4	551	67.7	NC_011987.1	pAtK84c	77.315
H3SJ34_1_GCA_029222685	JARHVB010000066.1	99.74	1624	63.49	NZ_AP014687.1	pNK6c	95.135
H3SJ34_1_GCA_029222685	JARHVB010000052.1	94.25	23059	62.88	NZ_CP032695.1	pRCCGE525c	75.897
H3SJ34_1_GCA_029222685	JARHVB010000053.1	79.98	30371	57.98	NZ_CP047896.1	pC33	79.757
H3SJ34_1_GCA_029222685	JARHVB010000056.1	87.24	1418	64.32	NZ_CP049700.1	pB323S2a	90.909
H3SJ34_1_GCA_029222685	JARHVB010000016.1	92.54	42156	57.45	NZ_CP044330.1	unnamed2	79.615
H3SJ34_1_GCA_029222685	JARHVB010000073.1	96.63	625	58.88	-	-	-
H3SJ34_1_GCA_029222685	JARHVB010000077.1	96.79	79861	61.01	NZ_CP025114.1	unnamed	89.647
H3SJ34_1_GCA_029222685	JARHVB010000047.1	98.74	20137	62.09	NZ_CP015743.1	pShin-07	75.68
H3SJ34_1_GCA_029222685	JARHVB010000007.1	97.17	54405	60.52	NC_009621.1	pSMED02	77.262
H3SJ34_1_GCA_029222685	JARHVB010000074.1	98.73	1255	60.16	-	-	-
H3SJ34_1_GCA_029222685	JARHVB010000075.1	73.86	506	63.24	-	-	-
H3SJ34_1_GCA_029222685	JARHVB010000009.1	87.61	29083	58.54	NZ_AP014659.1	pNK6a	92.875
H3SJ34_1_GCA_029222685	JARHVB010000027.1	95.62	168407	62.81	NZ_CP033508.1	pMJ700743a	82.484
H3SJ34_1_GCA_029222685	JARHVB010000029.1	70.26	124507	58.19	NC_008242.1	1	79.049
H3SJ34_1_GCA_029222685	JARHVB010000026.1	95.27	25733	62.56	NZ_CP044544.1	pBbPL7HG1	87.473
H3SJ34_1_GCA_029222685	JARHVB010000070.1	97.4	6191	61.09	NZ_LR134450.1	8	80.189
H3SJ34_1_GCA_029222685	JARHVB010000041.1	76.95	74830	63.32	NC_014007.1	pCHQ1	81.501
H3SJ34_1_GCA_029222685	JARHVB010000069.1	98.77	7358	60.7	NZ_CP032827.1	unnamed2	81.026
H3SJ34_1_GCA_029222685	JARHVB010000045.1	96.41	20561	60.99	NZ_AP014705.1	pMaq22A_1p	82.713
H3SJ34_1_GCA_029222685	JARHVB010000034.1	79.49	544	58.09	NZ_CP006990.1	pRetIE4771d	72.753
H3SJ34_1_GCA_029222685	JARHVB010000064.1	85.01	7271	55.93	NZ_CP049700.1	pB323S2a	99.01
H3SJ34_1_GCA_029222685	JARHVB010000022.1	71.42	17852	62.47	NZ_AP014705.1	pMaq22A_1p	77.9
H3SJ34_1_GCA_029222685	JARHVB010000048.1	95.98	16530	60.16	NC_017966.1	pTM2	72.572
H3SJ34_1_GCA_029222685	JARHVB010000015.1	91.27	59225	62.18	NZ_AP014579.1	p1 DNA	76.081
H3SJ34_1_GCA_029222685	JARHVB010000024.1	96.41	142045	63.41	NC_017536.1	pHCG3B	79.981
H3SJ34_1_GCA_029222685	JARHVB010000062.1	99.73	2452	59.34	NZ_AP014659.1	pNK6a	91.346

The predicted plasimid sequences from the genome of strain H3M7-6^T.

Sample name	Contig name	Probability (%)	Contig length (bp)	GC content (%)	Acc num	Acc plasmid name	Identity (%)
H3M7_6_GCF_029211265	NZ_JARGEL010000040.1	92.89	924	70.02	NZ_CP011839.1	pSg1-NDM	97.817
H3M7_6_GCF_029211265	NZ_JARGEL010000045.1	91.26	2919	61.22	CP045288.2	pCffl	81.139
H3M7_6_GCF_029211265	NZ_JARGEL010000017.1	71.24	139989	59.73	NC_006823.1	1	97.397
H3M7_6_GCF_029211265	NZ_JARGEL010000035.1	84.28	566	61.66	-	-	-
H3M7_6_GCF_029211265	NZ_JARGEL010000049.1	89.6	9271	56.59	NZ_CP040718.1	pHX3	79.641
H3M7_6_GCF_029211265	NZ_JARGEL010000009.1	80.9	61432	65.41	NZ_CP039632.3	unnamed	96.571

The predicted plasimid sequences from the genome of strain H3Y2-7^T.

Sample name	Contig name	Probability (%)	Contig length (bp)	GC content (%)	Acc num	Acc plasmid name	Identity (%)
H3Y2_7_GCA_029076445	JARESG010000086.1	79.38	570	68.77	-	-	-
H3Y2_7_GCA_029076445	JARESG010000069.1	93.65	1855	64.31	NC_015147.1	pASPHE302	85.314
H3Y2_7_GCA_029076445	JARESG010000034.1	94.9	23121	62.75	NZ_CP047900.1	unnamed2	90.44
H3Y2_7_GCA_029076445	JARESG010000089.1	-	523	60.8	NZ_CP029643.1	pDCT5	97.08
H3Y2_7_GCA_029076445	JARESG010000084.1	73.77	659	63.88	NZ_CP005191.1	pMI2	99.697
H3Y2_7_GCA_029076445	JARESG010000085.1	79.52	630	61.9	-	-	-
H3Y2_7_GCA_029076445	JARESG010000016.1	86.49	104249	63	NZ_KJ410765.1	p2MP	94.817
H3Y2_7_GCA_029076445	JARESG010000049.1	-	6416	61.07	NZ_CP017422.1	pZXY21	88.673
H3Y2_7_GCA_029076445	JARESG010000050.1	82.82	6231	63.97	NZ_KJ410765.1	p2MP	89.917
H3Y2_7_GCA_029076445	JARESG010000056.1	83.25	4959	64.73	NC_019339.1	pJ349-116	95.691
H3Y2_7_GCA_029076445	JARESG010000087.1	82.29	564	70.57	-	-	-
H3Y2_7_GCA_029076445	JARESG010000080.1	-	900	64.22	NZ_CP046569.1	pII	76.496
H3Y2_7_GCA_029076445	JARESG010000037.1	-	20565	59.73	NZ_KJ410765.1	p2MP	91.742
H3Y2_7_GCA_029076445	JARESG010000026.1	88.49	41126	61.91	NC_019339.1	pJ349-116	91.548
H3Y2_7_GCA_029076445	JARESG010000090.1	79.32	512	70.7	-	-	-
H3Y2_7_GCA_029076445	JARESG010000076.1	-	1415	62.83	-	-	-
H3Y2_7_GCA_029076445	JARESG010000048.1	97.79	7232	60.25	NZ_CP017426.1	pZXY25	80.66
H3Y2_7_GCA_029076445	JARESG010000054.1	-	5053	58.26	-	-	-
H3Y2_7_GCA_029076445	JARESG010000045.1	-	10326	61.89	NZ_CP029643.1	pDCT5	89.593
H3Y2_7_GCA_029076445	JARESG010000067.1	-	1964	65.22	NZ_CP029643.1	pDCT5	97.659
H3Y2_7_GCA_029076445	JARESG010000057.1	89.53	4780	64.23	NC_015146.1	pASPHE301	78.956
H3Y2_7_GCA_029076445	JARESG010000070.1	-	1823	66.43	NZ_KJ410765.1	p2MP	86.207
H3Y2_7_GCA_029076445	JARESG010000032.1	96.53	26862	61.44	NC_011881.1	pACHL02	85.167
H3Y2_7_GCA_029076445	JARESG010000042.1	77.79	12178	62.89	NC_019332.1	pJ353-116	94.223
H3Y2_7_GCA_029076445	JARESG010000043.1	74.55	12087	62.89	NC_008712.1	pTC1	86.764
H3Y2_7_GCA_029076445	JARESG010000060.1	-	3080	59.42	NZ_LR134469.1	27	76.78
